# Bone marrow-derived mesenchymal stem cells combined with intramuscular injection promote the healing of diabetic foot ulcers: a systematic review and meta-analysis

**DOI:** 10.3389/fendo.2026.1763071

**Published:** 2026-03-10

**Authors:** Xuliu Zhang, Guobin Liu, Yang You, Huimin Lu, Weijing Fan, Weiran Li

**Affiliations:** Department of Vascular Surgery, Shuguang Hospital Affiliated to Shanghai University of Traditional Chinese Medicine, Shanghai, China

**Keywords:** amputation, diabetic foot, diabetic foot ulcer, mesenchymal stem cell, meta-analysis, neovascularization, stem cell transplantation, wound healing

## Abstract

**Background:**

Diabetic foot ulcers (DFUs) represent a debilitating complication of diabetes characterized by high rates of morbidity and mortality, yet conventional management often yields limited efficacy. Although cell-based therapies offer a promising therapeutic avenue, their clinical efficacy and optimal target populations remain to be definitively established.

**Methods:**

We searched databases including PubMed, Embase, the Cochrane Library, Web of Science, and the China National Knowledge Infrastructure from inception to November 2025 for randomized controlled trials (RCTs). Study quality was assessed using the Revised Cochrane risk-of-bias tool for randomized trials. Meta-analysis, subgroup analysis, and heterogeneity analysis were performed using R software (version 4.3.3). Sensitivity analysis was conducted via the leave-one-out method.

**Results:**

Thirty-two RCTs involving 2059 patients were included, assessing nine core outcomes including ulcer healing rate and amputation rate. Pooled analysis demonstrated that cell therapy significantly improved the ulcer healing rate (OR = 4.64, 95% CI: 3.11 to 6.90), reduced the amputation rate (OR = 0.29, 95% CI: 0.18 to 0.49), enhanced limb perfusion (ABI MD = 0.14, 95% CI: 0.05 to 0.22; TcPO_2_ MD = 11.58, 95% CI: 5.36 to 17.80), alleviated pain (resting pain score MD = -1.04, 95% CI: -1.49 to -0.59), reduced ulcer area (MD = -2.15, 95% CI: -3.74 to -0.56), and shortened healing time (MD = -16.83 days, 95% CI: -27.93 to -5.74). Subgroup analyses revealed: 1) Cell Type: Autologous bone marrow-derived mesenchymal stem cells (BMMSCs) yielded the most favorable outcomes (ulcer healing OR = 8.33, *P* < 0.01). 2) Administration Protocol: A medium-to-high dose range combined with intramuscular injection was identified as optimal. Specifically, high and very-high doses demonstrated the strongest efficacy in critical limb salvage outcomes (amputation and healing rates), while the medium-dose regimen exhibited the most robust statistical consistency across all secondary metrics (e.g., ulcer area reduction). 3) Target Population: Patients with shorter diabetes duration (<10 years), larger ulcer area (≥10 cm²), or prolonged non-healing ulcers (>200 days) derived more significant benefit.

**Conclusion:**

Cell therapy shows significant potential as an adjuvant treatment for DFUs. Current evidence suggests that a protocol utilizing autologous BMMSCs within a medium-to-high dose range (targeting 1×10^7^ to 1.2×10^9^ cells) via intramuscular injection may optimize therapeutic efficacy while ensuring clinical feasibility. Exploratory findings indicate that this strategy might be particularly suitable for patients with large, refractory DFUs and short diabetes duration, though these observations require further validation in large-scale trials.

**Systematic review registration:**

https://www.crd.york.ac.uk/PROSPERO/, identifier CRD420251247289.

## 1 Background

Diabetic foot (DF), one of the most severe chronic complications of diabetes mellitus, arises from a pathological triad of diabetes-associated peripheral neuropathy, peripheral arterial disease, and localized infection. This culminates in foot ulceration (DFU), tissue ischemia, necrosis, and ultimately, limb amputation (1). Globally, the prevalence of foot ulcers among diabetic patients ranges from 19% to 34%, with 15%-24% of these patients facing the risk of lower extremity amputation (2). Compared to non-diabetic individuals, the risk of lower limb amputation is 30–40 times higher in patients with diabetes. Post-amputation, the one-year mortality rate can reach 20%-50%, imposing a substantial burden on patients, families, and healthcare systems (3). Current conventional management of DF relies on a multidisciplinary framework, encompassing glycemic and lipid control, anti-infective therapy, wound debridement, offloading, and revascularization procedures. Notably, recent advances in orthopedic surgical management, such as minimally invasive metatarsal osteotomies, have demonstrated significant efficacy in correcting biomechanical deformities and promoting the healing of plantar pre-ulcerative and ulcerative lesions (4). However, despite these comprehensive vascular and orthopedic limb-salvage strategies, the clinical efficacy remains limited for a subset of patients (5). For patients with severe lower extremity arterial occlusive disease who are ineligible for or refractory to surgical revascularization and standard care, amputation often becomes the final therapeutic option (6). Studies indicate that approximately 20%-30% of DF patients, due to diffuse vascular disease, distal occlusion, or multi-organ comorbidities, fail to benefit from conventional therapies, highlighting an urgent need for novel interventions (7, 8).

Cell-based therapy, a cornerstone of regenerative medicine, offers a novel paradigm for DF treatment. Despite their diverse origins, these cell-based therapeutic strategies consistently promote the extracellular secretion of bioactive factors. The core mechanism involves the transplantation of stem/progenitor cells with angiogenic potential (e.g., mesenchymal stem cells [MSCs], peripheral blood mononuclear cells). These cells promote local angiogenesis, secrete pro-healing factors such as vascular endothelial growth factor (VEGF) and basic fibroblast growth factor (bFGF), ameliorate the ischemic microenvironment, and accelerate granulation tissue formation and re-epithelialization within the ulcer bed (9, 10). In recent years, numerous clinical studies have substantiated the potential of cell therapy in improving limb ischemia (as evidenced by increases in the ankle-brachial index [ABI] and transcutaneous oxygen pressure [TcPO_2_]), enhancing DFU healing rates, and reducing amputation risk (11–13).

Nevertheless, significant heterogeneity exists among current clinical trials regarding critical parameters such as cell type, delivery method, and treatment regimen. Many studies are also limited by small sample sizes, leading to inconsistent conclusions regarding the efficacy and prognostic impact of cell therapy for DF (14). For instance, there is a lack of standardized criteria to evaluate the angiogenic capacity and ulcer-healing efficacy of different cell types. Therefore, there is a pressing need for a systematic review and meta-analysis to synthesize data from existing randomized controlled trials. This approach is essential to comprehensively evaluate the integrated efficacy of cell therapy on DFU healing, amputation risk, and improvement of limb ischemia, thereby providing high-quality evidence for clinical decision-making. While previous systematic reviews, notably by Sun et al. (15) and Guo et al. (16), have substantiated the general efficacy of cell therapy, they often lack granular guidance on the optimal treatment regimen due to the aggregation of heterogeneous cell types and delivery methods. Building upon these foundational studies, the present meta-analysis incorporates 32 high-quality RCTs to further refine the understanding of “dose-effect” and “route-effect” relationships. Complementing earlier broad-spectrum reviews, we aimed to systematically evaluate the efficacy of autologous bone marrow-derived mesenchymal stem cells (BMMSCs) combined with intramuscular injection and explore the optimal cell dosage. Furthermore, this review provides a comprehensive stratification of patient populations, assessing how baseline characteristics—such as diabetes duration (<10 years), ulcer size (≥10 cm²), and chronicity (>200 days)—influence therapeutic outcomes, thereby offering more personalized evidence for clinical practice. Consequently, this meta-analysis was conducted to elucidate the clinical value and application prospects of cell therapy in the management of diabetic foot.

## 2 Methods

### 2.1 Study design and registration

This study is a systematic review and meta-analysis of randomized controlled trials (RCTs), designed and reported in strict accordance with the Preferred Reporting Items for Systematic Reviews and Meta-Analyses (PRISMA 2020) statement (17). The study protocol was registered on the PROSPERO international prospective register of systematic reviews on December 5, 2025 (Registration No.: CRD420251247289), ensuring transparency and methodological reproducibility.

### 2.2 Literature search strategy

A systematic search was conducted across multiple databases, including PubMed, Embase, the Cochrane Library, Web of Science, and the China National Knowledge Infrastructure (CNKI). The search covered the period from the inception of each database to November 15, 2025. Language restrictions were applied to include studies published in English and Chinese. The search employed a combination of Medical Subject Headings (MeSH) terms and free-text words. Core English search terms included “diabetic foot,” “cell therapy,” “stem cell,” and “bone marrow,” while core Chinese search terms included “diabetic foot,” “cell therapy,” “stem cells,” and “bone marrow mononuclear cells.” The detailed search strategy is provided in **Supplementary Table 1**.

### 2.3 Inclusion and exclusion criteria

The research objective and eligibility criteria were structured based on the PICOS framework: Population (patients with DFU), Intervention (cell-based therapies), Comparator (standard of care or placebo), Outcomes (ulcer healing, amputation, etc.), and Study design (parallel-group RCTs).

Specific inclusion criteria were as follows: (1) Study design: RCTs with a parallel-group design, regardless of blinding status. (2) Population: Patients aged 18 years or older with a clinical diagnosis of diabetic foot ulcer (DFU) secondary to type 1 or type 2 diabetes mellitus, with no restrictions based on race or gender. (3) Intervention: The experimental group received any form of cell-based therapy, including but not limited to autologous or allogeneic bone marrow mononuclear cells (BMMNCs), BMMSCs, peripheral blood progenitor cells, or adipose-derived stem cells, administered via local injection, topical application, or intravascular infusion. (4) Comparator: The control group received either placebo (e.g., saline), conventional standard care (e.g., debridement, dressings, offloading), or other non-cellular therapies. (5) Outcomes: Studies must have reported at least one of the following primary or secondary outcomes. Primary outcomes: (1) complete ulcer healing rate; (2) amputation rate; (3) improvement in ABI or TcPO_2_. Secondary outcomes: (1) ulcer healing time; (2) resting pain score; (3) pain-free walking distance; (4) post-treatment ulcer area.

Exclusion criteria were: (1) Crossover trials were excluded due to the nature of the endpoint (ulcer healing) and potential carryover effects of cell therapy. (2) Non randomized study designs, such as case reports, case series, observational studies, animal experiments, or *in vitro* studies. (3) Studies involving patients with non-diabetic ulcers, non-foot ulcers, or those complicated by active malignancy or severe systemic infection. (4) Interventions involving non-cellular biological therapies, such as growth factor treatment, platelet-rich plasma, or tissue-engineered skin. (5) Full texts that were unavailable or contained incomplete/unclear data, even after contacting the authors.

### 2.4 Data extraction and management

Two investigators independently extracted information using a pre-designed, standardized data collection form. The extracted content encompassed basic study characteristics (author, publication year, trial design), patient baseline data (sample size, age, diabetes duration, DFU grade), intervention details (cell type, dose, route of administration, and frequency), outcome data (event counts for dichotomous outcomes; means and standard deviations for continuous outcomes), and information on loss to follow-up. Any discrepancies between the two investigators were resolved through joint re-examination of the original articles or consultation with a third researcher. For studies with incomplete outcome data, attempts were made to contact the original authors to obtain the raw data to ensure the accuracy of the analysis. To avoid unit-of-analysis errors and potential carry-over effects inherent in crossover designs, we exclusively extracted and analyzed data from the first intervention period, treating these trials as parallel-group studies.

### 2.5 Risk of bias assessment

The Cochrane Collaboration’s Revised Cochrane risk-of-bias tool for randomized trials (RoB 2.0) was used to independently assess the risk of bias in the included RCTs. The core domains evaluated included the randomization process, allocation concealment, blinding of participants and personnel, blinding of outcome assessment, completeness of outcome data, selective reporting, and other potential biases (e.g., baseline imbalance, conflict of interest). The overall risk of bias for each study was categorized as low, unclear, or high. Disagreements in quality assessment were resolved in the same manner as during data extraction.

### 2.6 Outcome measures and standardization

To minimize heterogeneity due to inconsistent outcome definitions, all primary endpoint data were standardized during extraction. Ulcer area was recorded in cm², with analysis focusing on the change from baseline to follow-up endpoint, provided no statistically significant difference in baseline area existed between groups. Follow-up duration was uniformly converted to months and time to ulcer healing was extracted in days. Outcome definitions were applied as follows: ulcer healing rate was defined as the proportion of patients achieving complete wound epithelialization during follow-up, excluding studies reporting only significant or partial healing; amputation rate as the proportion undergoing any major (above-/below-knee) or minor (metatarsal/toe) amputation; limb perfusion parameters (ABI and TcPO_2_ in mmHg) were extracted as continuous variables, preferentially using the change from baseline or, if unavailable, the absolute post-treatment value; resting pain assessed by VAS was converted to a unified 0–10 scale; neovascularization was extracted both as a rate (proportion of patients with new vessel formation) and as a quantitative score (including metrics such as blood flow velocity in mm/s and vessel diameter), with qualitatively reported studies excluded from quantitative synthesis.

### 2.7 Rationale for subgroup stratification

For the subgroup analyses, patients were stratified based on clinical characteristics and treatment protocols. The duration of diabetes was dichotomized at a cutoff of 10 years. This threshold approximates the median disease duration reported in the included studies and is clinically recognized as a critical juncture associated with a significant increase in microvascular complications and the progression to a more chronic disease state. Ulcer area was categorized using a cutoff of 10 cm² to differentiate between medium and large ulcers. This value aligns with the median baseline ulcer size reported in most studies, which helps achieve a more balanced distribution of ulcer severity across the compared subgroups. To rigorously evaluate the dose-response relationship, the total cell number administered per patient was classified into four tiers based on the natural distribution of dosages and the clustering of therapeutic approaches in the included literature: a Low-dose group (0.5×10^6^ to 8×10^6^ cells), a Medium-dose group (1×10^7^ to 8.6×10^7^ cells), a High-dose group (3×10^8^ to 1.2×10^9^ cells), and an Ultra-high-dose group (≥2×10^9^ cells). Studies that reported only cell concentration without total volume, or where the dose was not quantifiable, were analyzed separately as a “Dose-unspecified” group.

### 2.7 Statistical analysis

All statistical analyses were performed using R software (version 4.3.3) with the meta package. For dichotomous outcomes (e.g., ulcer healing rate, amputation rate), pooled odds ratios (ORs) with 95% confidence intervals (CIs) were calculated using the Mantel-Haenszel method. For continuous outcomes (e.g., changes in ABI, TcPO_2_, ulcer healing time), mean differences (MDs) or standardized mean differences (SMDs) with 95% CIs were estimated using the Inverse Variance method. Heterogeneity among studies was assessed using the Cochran *Q* test and quantified with the *I²* statistic. Given the anticipated clinical heterogeneity regarding cell types and treatment protocols, a random-effects model was primarily employed to provide conservative estimates. A fixed-effects model was used only when heterogeneity was negligible (*I²* < 50% and *P*>0.10). Subgroup analyses were conducted to explore potential sources of heterogeneity and were evaluated using the test for subgroup differences (interaction *Q*-test). Sensitivity analyses were performed using the leave-one-out method to assess the robustness of the results. Potential publication bias was evaluated visually using funnel plots and quantitatively using Egger’s linear regression test, provided that at least 10 studies were included in the analysis.

## 3 Results

### 3.1 Literature screening process and results

**Figure 1:**
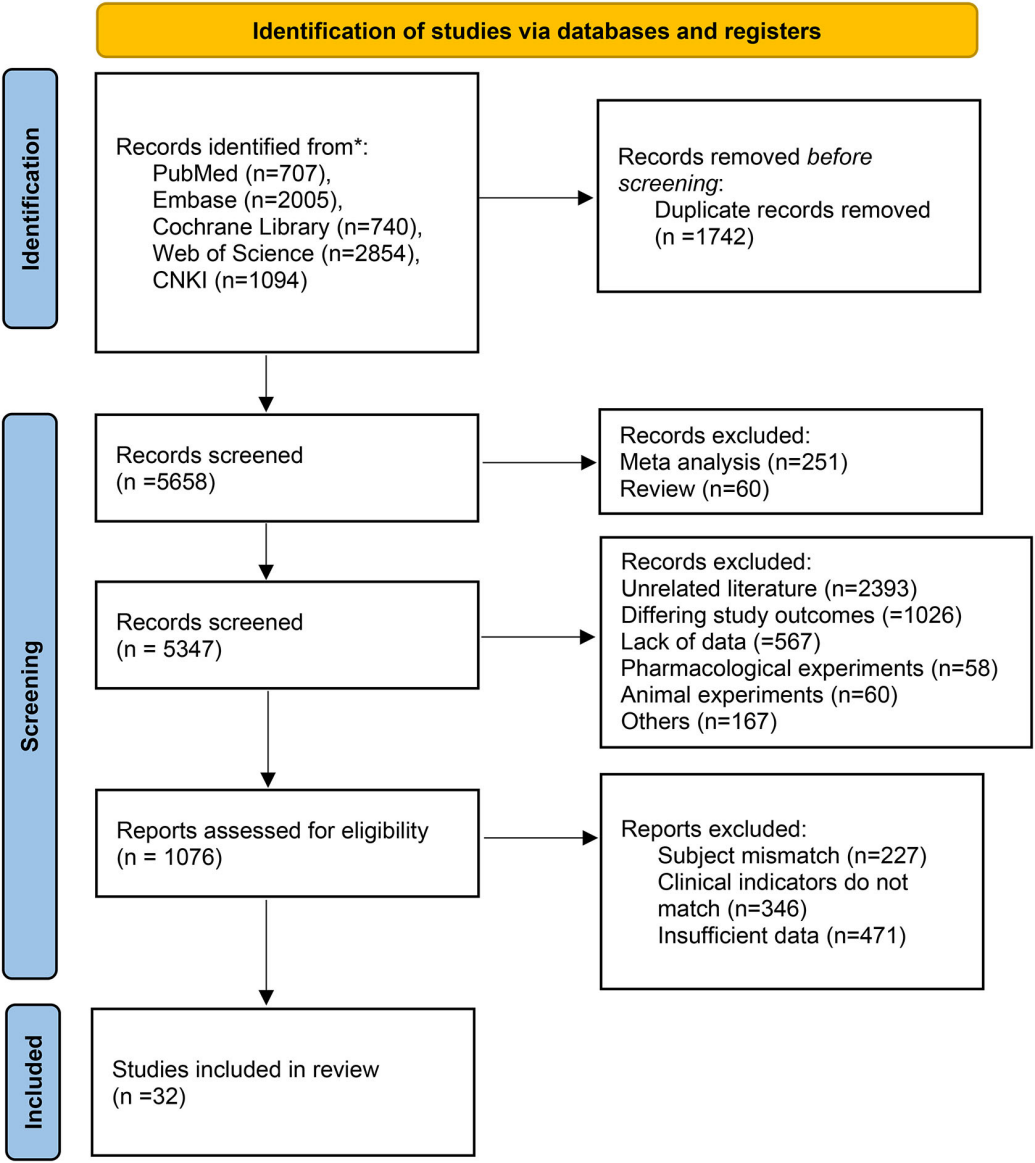
Literature screening flow diagram.

Following the PRISMA 2020 flow diagram (**Figure 1**), a total of 7,400 records were initially identified through systematic searches across the five databases: PubMed, Embase, Cochrane Library, Web of Science, and CNKI. After removing 1,742 duplicates, 5,658 records remained for title and abstract screening. During this initial screening, 4,271 records were excluded for the following reasons: irrelevant to the research objective (n = 2,393), outcomes not matching (n = 1,026), incomplete data (n = 567), pharmacological or animal studies (n = 118), and other reasons for ineligibility (n = 167). Full-text retrieval and detailed assessment were performed for the remaining 1,076 articles. Based on the pre-defined criteria, further exclusions were made: 227 studies due to mismatched study population, 346 due to non-conforming clinical evaluation metrics, and 471 due to insufficient key data for quantitative analysis. Ultimately, 32 RCTs (18–49) met all inclusion criteria and were selected for analysis. The basic characteristics of the included studies are presented in **Table 1** (additional treatment details are provided in **Supplementary Tables 2**, **3**).

**Table 1:** Basic characteristics of the included studies.

Author	Year	Age (T/C)	N (T/C)	Country	Cell type	Route	Follow-up (months)
Ozturk (26)	2012	71.9 ± 9.2	20/20	Turkey	PBMNC	IM	3
Rakowska (48)	2023	56.7 ± 11.1	23/23	Poland	ASC	Topical	1.6
Dash (19)	2009	40 ± 10	3/3	India	BMMSC	IM	3
Dubsky1 (23)	2013	60.7 ± 9.4	17/20	Czech Rep.	BMMNC	IM	6
Dubsky2 (23)	2013	63.4 ± 10.4	11/22	Czech Rep.	PBMNC	IM	6
Raposio (35)	2016	70.75	16/24	Italy	ASC	SC	18
Tanios (36)	2021	48.12 ± 14.58	50/50	Egypt	ASC	SC	6
Uzun (32)	2021	57.5 ± 8.4	10/10	Turkey	ASC	SC	26-50
Tan (49)	2017	64.0 ± 7.1	50/50	China	PBMNC	SC	6
Wang_A (42)	2024	61 (42-75)	15/15	China	PBMNC	SC	3
Qin (29)	2016	75 ± 3	28/25	China	UC-MSC	IM	3
Han (21)	2010	66.5 ± 7.5	26/26	S. Korea	ASC	Topical	2
He	2013	63.3	50/50	China	UC-MSC	IM	3
Kirana1 (25)	2012	68.5 ± 1.5	12/6	Germany	BMMNC	IM	52
Kirana2 (25)	2012	70.9 ± 1.7	10/6	Germany	BMMSC	IM	52
Moon (33)	2019	59.9 ± 13.3	22/17	S. Korea	ASC	Topical	12
Mohammadzadeh (27)	2013	63.5 ± 7.8	7/14	Iran	PBMNC	IM	3
Lu1 (21)	2011	65 ± 10	19/37	China	BMMNC	IM	6
Lu2 (21)	2011	63 ± 8	18/37	China	BMMSC	IM	6
Debin (38)	2008	66.55 ± 6.50	22/23	China	BMMSC	IM + SC	3
Dubský (24)	2014	62.7 ± 10.4	31/23	Czech/UK	BMMNC	IM	12
Smith (47)	2020	60.2 (45-78)	6/6	UK	ASC	SC	3
Liu_A (44)	2023	65.89 ± 6.17	50/50	China	BMMSC	IM	1
Liu_B (45)	2021	58.39 ± 6.42	54/51	China	BMMSC	IM	6
Sui (39)	2020	57.31 ± 5.62	43/43	China	UC-MSC	IV + SC	3
Huang (18)	2005	71.1 ± 5.9	14/14	China	PBMNC	SC + IM	3
Jain (22)	2011	54.25 (33-76)	23/24	India	BM-DC	SC + Top.	3
Pollak1 (37)	2025	58.9 ± 12.17	44/45	USA	UC-MSC	IM	24
Pollak2 (37)	2025	56.0 ± 11.82	47/45	USA	UC-MSC	IM	24
Pollak3 (37)	2025	58.1 ± 12.25	23/45	USA	UC-MSC	IM	24
Lonardi (31)	2019	69.0 ± 11.6	55/50	Italy	ASC	SC	6
Meamar1 (34)	2021	56 ± 10.5(F)/68 ± 8.1(M)	11/7	Iran	UC-MSC	Topical	16
Meamar2 (34)	2021	56 ± 10.5(F)/68 ± 8.1(M)	10/7	Iran	UC-MSC	Topical	16
Wang_B (43)	2024	63.14 ± 6.42	43/43	China	UC-MSC	SC	3
Li_A (41)	2014	57.5 ± 4.2	30/26	China	UC-MSC	IM	3
Zhang (30)	2016	71.26 ± 9.12	27/26	China	PBMNC	IA	18
Zhao (40)	2018	55.2	37/43	China	ASC	SC	1-6
Li_B (46)	2021	59.06 ± 6.13	28/28	China	BMMSC	IM	3

Data in the Age (T/C) column are presented as mean ± standard deviation (SD), mean (range), or mean alone, as reported in the original studies. Abbreviations: T, Treatment group; C, Control group; BMMSC, Bone marrow mesenchymal stem cells; BMMNC, Bone marrow mononuclear cells; PBMNC, Autologous peripheral blood mononuclear cells; ASC, Adipose-derived stem cells (or related components); UC-MSC, Umbilical cord/placental mesenchymal stem cells; BM-DC, Bone marrow-derived dendritic cells; IM, Intramuscular injection; SC, Subcutaneous injection; IV, Intravenous injection; IA, Intra-arterial injection; Top., Topical application; F, Female; M, Male; SD, Standard deviation.

### 3.2 Risk of bias assessment

**Figure 2:**
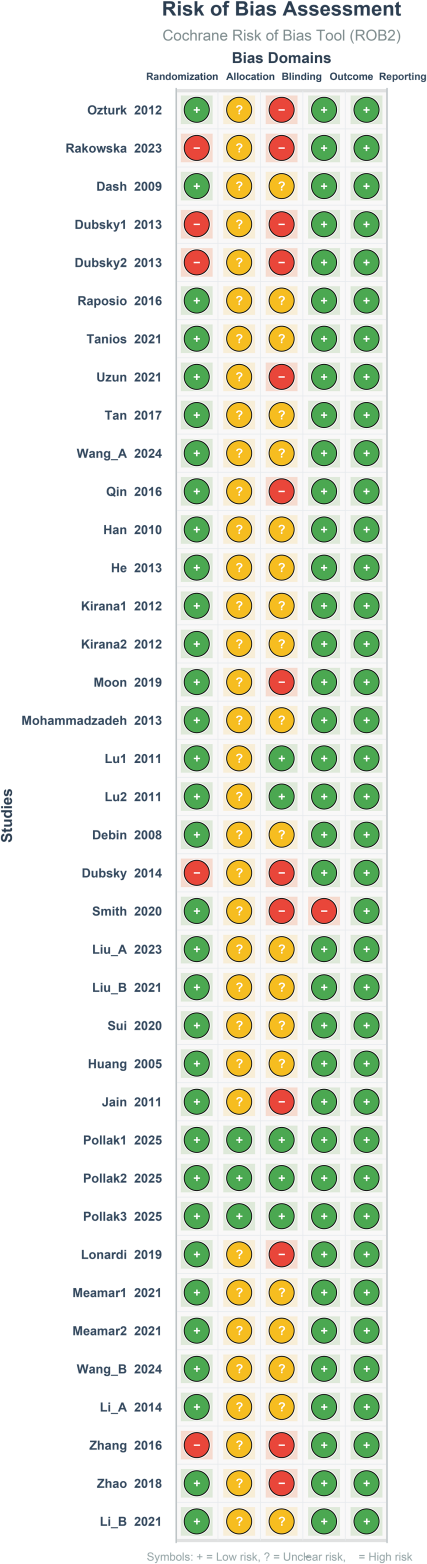
Risk of bias assessment graph.

The risk of bias assessment for the included studies demonstrated methodological heterogeneity. Regarding random sequence generation, 28 studies were judged as low risk, while 4 were rated high risk due to unclear methodological descriptions. The implementation of allocation concealment was generally poor, with only 3 studies rated low risk; the remaining 29 were of unclear risk. Blinding was the weakest domain. Only 3 studies were adequately blinded, while 15 were rated as high risk due to the lack of blinding or partial blinding, which may introduce detection bias in subjective outcome assessments. The vast majority of studies demonstrated low risk concerning the completeness of outcome data and selective reporting (see **Figure 2**; **Supplementary Figure 1**). Overall, the included studies presented a moderate risk of bias. While selection and attrition biases were generally well-controlled, substantial concerns remain regarding performance and detection biases due to the prevalence of unblinded designs and unclear allocation concealment.

### 3.3 Meta-analysis of ulcer healing rate

#### 3.3.1 Overall efficacy analysis

A total of 1,624 patients reported ulcer healing outcomes. Pooled results using a random-effects model showed a significantly higher ulcer healing rate in the cell therapy group compared to the control group, with a pooled odds ratio (OR) of 4.64 (95% CI: 3.11 to 6.90; *P* < 0.001) (**Supplementary Figure 2**). Heterogeneity testing indicated moderate statistical heterogeneity (*I²* = 53%), confirming the appropriateness of the chosen statistical model.

#### 3.3.2 Subgroup analysis

To explore sources of heterogeneity and factors influencing efficacy, pre-specified subgroup analyses were conducted, with results as follows:

##### 3.3.2.1 Geographic subgroup analysis

**Figure 3:**
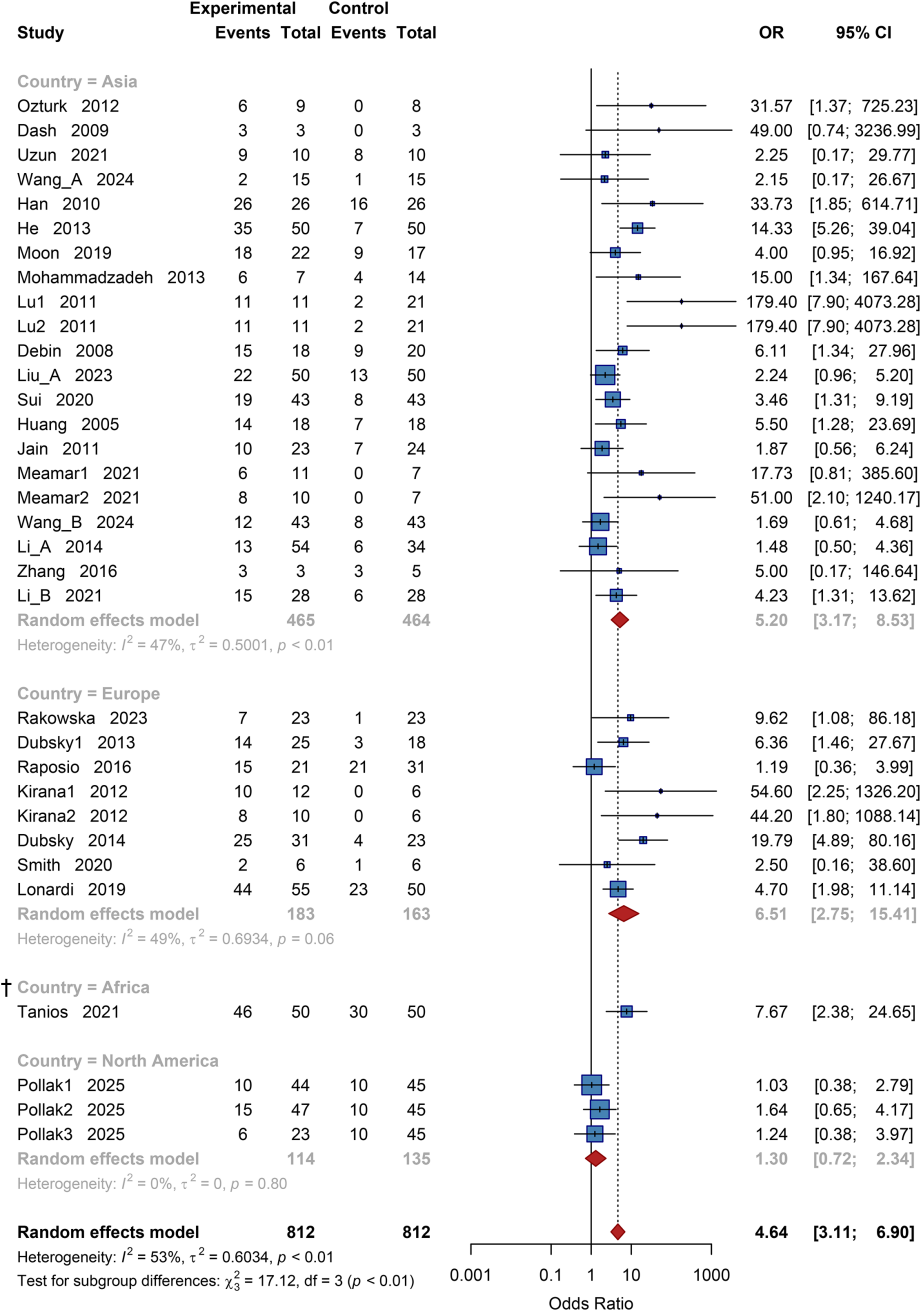
Subgroup analysis of ulcer healing rate by country. OR, Odds Ratio; CI, Confidence Interval, † Results based on sparse data (number of studies k<3) should be interpreted with caution.

Studies were stratified by region into four subgroups: Asia, Europe, Africa, and North America. The test for subgroup differences indicated significant variation (χ² = 17.12, df = 3, *P* < 0.01). The pooled OR for the Asian subgroup was 5.20 (95% CI: 3.17 to 8.53); for the European subgroup, 6.51 (95% CI: 2.75 to 15.41). The result for North America was 1.30 (95% CI: 0.72 to 2.34). These results indicated that the ulcer healing effect of cell therapy was more pronounced in Asian and European regions, while no statistically significant difference was observed in the North American subgroup (**Figure 3**).

##### 3.3.2.2 Subgroup analysis by cell type

Studies were stratified into six subgroups based on cell type: autologous peripheral blood mononuclear cells (PBMNCs), adipose-derived stem cells (ASCs), BMMSCs, BMMNCs, umbilical cord/placental mesenchymal stem cells (UC-MSCs/PL-MSCs), and autologous bone marrow dendritic cells. The difference between subgroups approached statistical significance (χ² = 11.34, df = 5, *P* = 0.05). The pooled OR for PBMNCs was 6.69 (95% CI: 2.45 to 18.30); for ASCs, 4.19 (95% CI: 2.28 to 7.69); for autologous BMMSCs, 8.33 (95% CI: 2.48 to 27.90); for autologous BMMNCs, 19.57 (95% CI: 5.81 to 65.89); for UC-MSCs/PL-MSCs, 2.73 (95% CI: 1.37 to 5.44); while in the autologous bone marrow dendritic cell subgroup, the study by Jain et al. reported an OR of 1.87 (95% CI: 0.56 to 6.24). These results suggest that autologous BMMNCs had the strongest effect, followed by autologous BMMSCs, whereas autologous bone marrow dendritic cells may be ineffective (**Supplementary Figure 3**).

##### 3.3.2.3 Subgroup analysis by cell dose

Studies were categorized by administered cell dose into low-dose, medium-dose, high-dose, very high-dose, and dose-unknown groups. A significant difference was observed between subgroups (χ² = 12.85, df = 5, *P* = 0.02). The pooled OR for the very high-dose group was 5.91 (95% CI: 2.10 to 16.66); for the high-dose group, 57.22 (95% CI: 13.41 to 244.15); for the medium-dose group, 3.41 (95% CI: 1.41 to 8.21); and for the low-dose group, 3.66 (95% CI: 1.79 to 7.48). The results suggest that high and very high cell doses are associated with more significant ulcer healing efficacy, indicating a potential dose-response relationship (**Supplementary Figure 4**).

##### 3.3.2.4 Subgroup analysis by delivery route

Studies were stratified by administration route into intramuscular injection, intra-arterial infusion, combined injection, subcutaneous injection, and local/topical therapy subgroups. The pooled OR for the intramuscular injection subgroup was 6.79 (95% CI: 3.12 to 14.79); for local therapy, 9.86 (95% CI: 3.38 to 28.77); for subcutaneous injection, 2.87 (95% CI: 1.51 to 5.46); and for combined injection, 3.51 (95% CI: 1.90 to 6.50). The results for intra-arterial infusion showed no statistically significant difference. This suggests that all administration routes except intra-arterial infusion significantly improved ulcer healing rates, with local therapy and intramuscular injection potentially offering greater advantages (**Supplementary Figure 5**).

##### 3.3.2.5 Other subgroup analyses

In the analysis stratified by follow-up duration, the subgroup with follow-up<10 months had a pooled OR of 4.99 (95% CI: 3.28 to 7.59), and the subgroup with follow-up ≥10 months had a pooled OR of 4.02 (95% CI: 1.76 to 9.21), indicating sustained efficacy through long-term follow-up (**Supplementary Figure 6**). Subgroup analysis based on baseline ulcer area showed a pooled OR of 5.78 (95% CI: 3.14 to 10.63) for ulcers<10 cm² and 5.22 (95% CI: 1.78 to 15.33) for ulcers ≥10 cm² (**Supplementary Figure 7**). Analysis by diabetes duration yielded a pooled OR of 7.68 (95% CI: 2.24 to 26.29) for a duration of 10 to 15 years, 9.30 (95% CI: 5.10 to 16.96) for >15 years, and 15.82 (95% CI: 2.10 to 119.16) for<10 years (**Supplementary Figure 8**). Subgroup analysis by ulcer duration: the subgroup with duration ≤200 days had a pooled OR of 18.20 (95% CI: 4.96 to 66.71), while the subgroup >200 days had a pooled OR of 3.87 (95% CI: 1.45 to 10.35). The difference between these subgroups approached significance (χ² = 3.46, df = 1, *P* = 0.06) (**Supplementary Figure 9**).

### 3.4 Meta-analysis of amputation rate

#### 3.4.1 Overall efficacy analysis

Amputation outcomes were reported for a total of 852 patients (397 in the intervention group, 455 in the control group). The pooled results using a fixed-effects model showed that the amputation rate was significantly lower in the cell therapy group compared to the control group, with a pooled odds ratio (OR) of 0.29 (95% CI: 0.18 to 0.49; P<0.001) (**Supplementary Figure 10**). Heterogeneity testing indicated no statistical heterogeneity (*I²* = 0%, *P* = 0.49), suggesting high reliability of the pooled estimate.

#### 3.4.2 Subgroup analysis

##### 3.4.2.1 Geographic subgroup analysis

**Figure 4:**
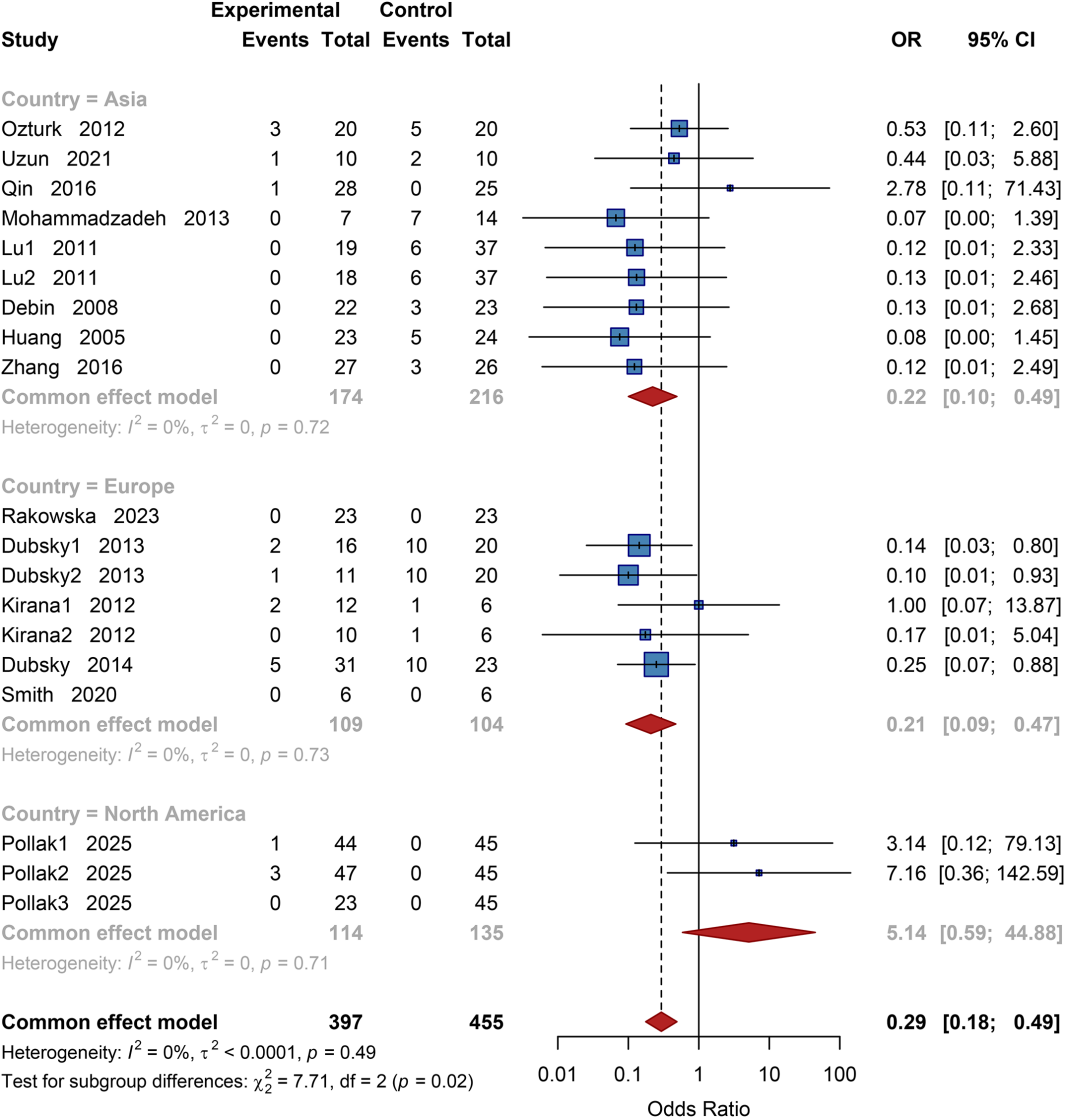
Subgroup analysis of amputation rate by country. OR, Odds Ratio, CI, Confidence Interval.

Studies were stratified by region into three subgroups: Asia, Europe, and North America. The test for subgroup differences indicated a significant variation (χ² = 7.71, df = 2, *P* = 0.02). The pooled OR for the Asian subgroup was 0.22 (95% CI: 0.10 to 0.49); for the European subgroup, 0.21 (95% CI: 0.09 to 0.47); and for the North American subgroup, 5.14 (95% CI: 0.59 to 44.88). These results indicate that cell therapy significantly reduced the amputation rate in Asian and European regions, while no statistically significant difference was observed in the North American subgroup (**Figure 4**).

##### 3.4.2.2 Subgroup analysis by cell type

Studies were stratified into five subgroups based on cell type: umbilical cord/placental mesenchymal stem cells (UC-MSCs/PL-MSCs), BMMSCs, BMMNCs, PBMNCs, and other cell types. The difference between subgroups was statistically significant (χ² = 10.97, df = 4, *P* = 0.03). The pooled OR for the autologous BMMSC subgroup was 0.14 (95% CI: 0.02 to 0.84); for the autologous BMMNC subgroup, 0.23 (95% CI: 0.09 to 0.54); for the autologous peripheral blood-derived cell subgroup, 0.17 (95% CI: 0.06 to 0.44); and for the UC-MSC/PL-MSC subgroup, 4.33 (95% CI: 0.72 to 25.97). These results suggest that autologous bone marrow-derived cells (BMMSCs and BMMNCs) significantly reduce the amputation rate, while UC-MSCs/PL-MSCs may not confer a clear benefit (**Supplementary Figure 11**).

##### 3.4.2.3 Subgroup analysis by cell dose

The analysis showed a pooled OR of 0.11 (95% CI: 0.03 to 0.38) for the very high-dose group; 0.17 (95% CI: 0.05 to 0.64) for the high-dose group; 0.58 (95% CI: 0.16 to 2.09) for the medium-dose group; and 0.99 (95% CI: 0.33 to 2.93) for the low-dose group. The results suggest that high and very high cell doses are associated with a more pronounced effect in reducing the amputation rate, indicating a potential dose-response trend (**Supplementary Figure 12**).

##### 3.4.2.4 Subgroup analysis by administration route

Studies were stratified by delivery route into intramuscular injection, subcutaneous injection, intra-arterial infusion, and other/combined methods. No statistically significant difference was found between subgroups. The pooled OR for the intramuscular injection subgroup was 0.34 (95% CI: 0.19 to 0.59); for the combined therapy subgroup, 0.10 (95% CI: 0.01 to 0.79); and for the intra-arterial infusion subgroup, 0.12 (95% CI: 0.01 to 2.49). The results suggest that various injection routes, except potentially intra-arterial infusion, can significantly lower the amputation rate, with combined therapy possibly offering greater advantage, although the between-group difference was not statistically significant (**Supplementary Figure 13**).

##### 3.4.2.5 Other subgroup analyses

In the analysis stratified by follow-up duration, the subgroup with follow-up<10 months had a pooled OR of 0.19 (95% CI: 0.09 to 0.39), while the subgroup with follow-up ≥10 months had a pooled OR of 0.53 (95% CI: 0.25 to 1.12). This suggests the effect is more pronounced in the short term, while long-term benefit requires further validation (**Supplementary Figure 14**). Subgroup analysis based on baseline ulcer area showed a pooled OR of 0.28 (95% CI: 0.15 to 0.50) for ulcers<10 cm² and 0.17 (95% CI: 0.03 to 1.07) for ulcers ≥10 cm², with no significant difference between subgroups (**Supplementary Figure 15**). Analysis by diabetes duration yielded a pooled OR of 0.10 (95% CI: 0.01 to 0.81) for duration<10 years, 0.46 (95% CI: 0.16 to 1.31) for 10 to 15 years, and 0.18 (95% CI: 0.06 to 0.48) for >15 years, with no significant difference between subgroups (**Supplementary Figure 16**). The subgroup with ulcer duration ≤200 days had a pooled OR of 0.20 (95% CI: 0.07 to 0.60), suggesting cell therapy can reduce amputation risk when ulcer duration is shorter (**Supplementary Figure 17**).

### 3.5 Meta-analysis of neovascularization rate

#### 3.5.1 Overall efficacy analysis

Seven studies included in this analysis reported outcomes related to neovascularization. The pooled results from a random-effects model demonstrated a significantly higher neovascularization rate in the cell therapy group compared to the control group, with a pooled OR of 15.36 (95% CI: 4.62 to 51.09) (**Supplementary Figure 18**). Heterogeneity testing indicated moderate statistical heterogeneity (*I²* = 50%, *P* = 0.06). As no extreme heterogeneity was present to preclude pooling, a random-effects model was used, yielding stable results.

#### 3.5.2 Subgroup analysis

##### 3.5.2.1 Geographic subgroup analysis

**Figure 5:**
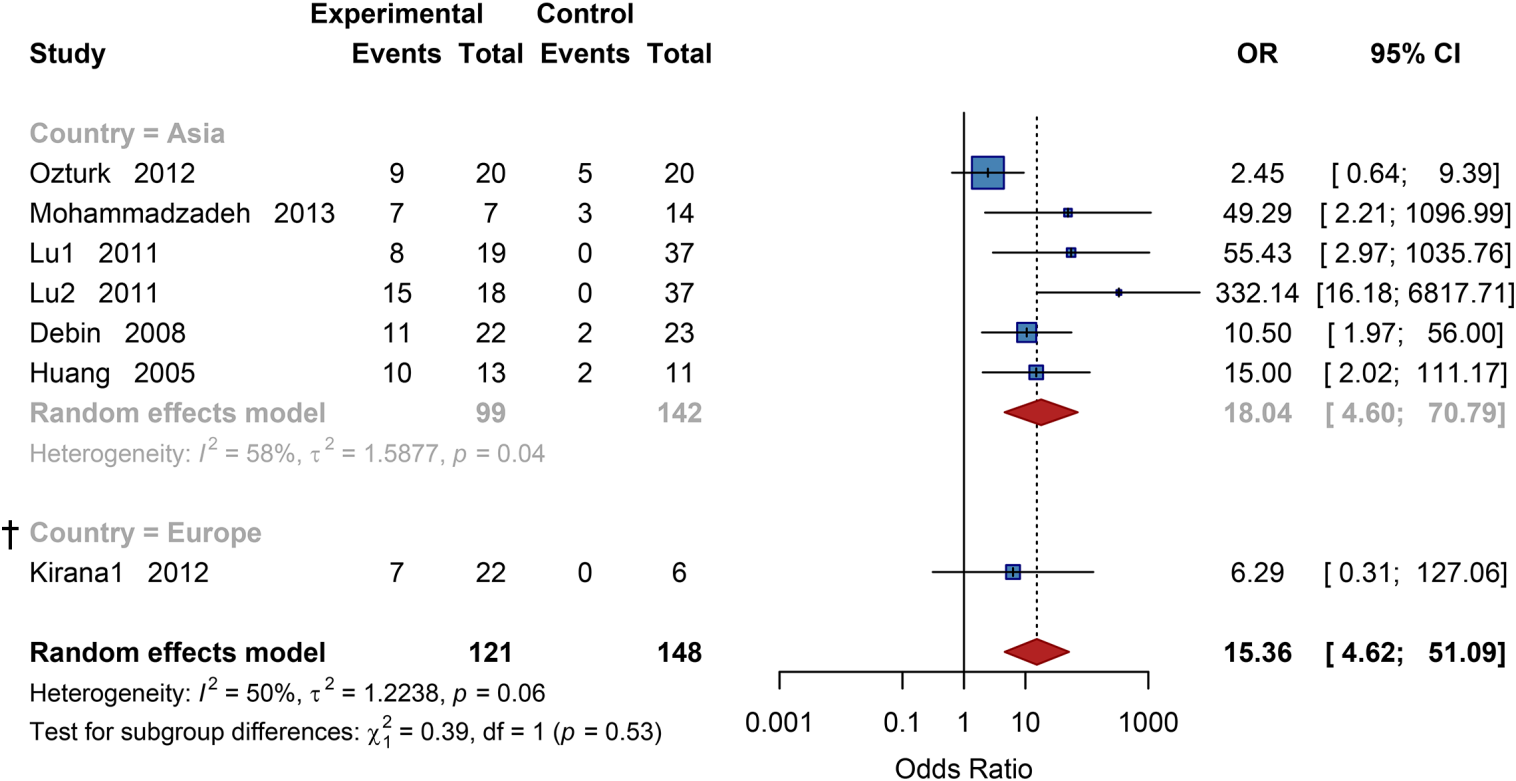
Subgroup analysis of neovascularization rate by country. OR, Odds Ratio; CI, Confidence Interval, † Results based on sparse data (number of studies k<3) should be interpreted with caution.

Studies were stratified by region into two prespecified subgroups: Asia and Europe. The test for subgroup differences showed no statistical significance. The pooled OR for the Asian subgroup was 18.04 (95% CI: 4.60 to 70.79), indicating significant efficacy. The result for the European region was OR = 6.29 (95% CI: 0.31 to 127.06), which did not reach statistical significance likely due to the limited sample size (**Figure 5**).

##### 3.5.2.2 Subgroup analysis by cell type

Studies were stratified by cell type into three subgroups: PBMNCs, BMMSCs, and BMMNCs. No statistically significant difference was observed between subgroups. The pooled OR for the autologous BMMNC subgroup was 19.20 (95% CI: 2.28 to 161.86); for the autologous peripheral blood-derived cell subgroup, 8.40 (95% CI: 1.52 to 46.54); and for the autologous BMMSC subgroup, 46.53 (95% CI: 1.63 to 1329.58). These results indicated positive outcomes for all three cell types, although the wide confidence intervals preclude determining the superiority of any specific type (**Supplementary Figure 19**).

##### 3.5.2.3 Subgroup analysis by cell dose

The analysis showed a pooled OR of 48.72 (95% CI: 9.94 to 238.85) for the high-dose group; OR = 15.00 (95% CI: 2.02 to 111.17) for the very high-dose group; OR = 10.50 (95% CI: 1.97 to 56.00) for the combined high/very high-dose group; and OR = 2.45 (95% CI: 0.64 to 9.39) for the low-dose group. The results suggest a potential dose-dependent relationship, with higher doses associated with larger effect estimates (**Supplementary Figure 20**).

##### 3.5.2.4 Subgroup analysis by administration route

Studies were categorized by delivery route into two main subgroups, with no statistical difference between them. The pooled OR for the intramuscular injection subgroup was 20.79 (95% CI: 3.22 to 134.28), and for the combined therapy subgroup, 12.16 (95% CI: 3.37 to 43.93). This suggests that intramuscular injection remains a robust delivery method for promoting neovascularization (**Supplementary Figure 21**).

##### 3.5.2.5 Other subgroup analyses

In the subgroup analysis by follow-up duration, the subgroup with follow-up <10 months had a pooled OR of 18.04 (95% CI: 4.60 to 70.79), whereas the subgroup with follow-up ≥10 months (OR = 6.29, 95% CI: 0.31 to 127.06) did not differ significantly, likely necessitating further long-term verification (**Supplementary Figure 22**). Subgroup analysis based on baseline ulcer area showed a pooled OR of 19.94 (95% CI: 7.12 to 55.89) for ulcers <10 cm² and OR = 49.29 (95% CI: 2.21 to 1069.99) for ulcers ≥10 cm², with no difference between subgroups (**Supplementary Figure 23**). Analysis by diabetes duration yielded a pooled OR of 131.21 (95% CI: 15.06 to 1143.17) for duration <10 years, compared to smaller estimates for longer durations. Although the difference was not statistically significant, patients with shorter disease duration showed a trend toward greater benefit (**Supplementary Figure 24**). For ulcer duration, the subgroup with duration <200 days had a pooled OR of 48.72 (95% CI: 9.94 to 238.85), indicating favorable outcomes for earlier intervention (**Supplementary Figure 25**). Additionally, a meta-analysis of neovascularization scores showed no statistically significant difference between the two groups (**Supplementary Figure 26**).

### 3.6 Meta-analysis of ABI

#### 3.6.1 Overall efficacy analysis

Twelve studies included in this analysis reported outcomes related to changes in ABI. The pooled results from a random-effects model demonstrated a significantly higher ABI value in the cell therapy group compared to the control group, with a pooled MD of 0.14 (95% CI: 0.05 to 0.22; *P* < 0.01) (**Supplementary Figure 27**).

#### 3.6.2 Subgroup analysis

##### 3.6.2.1 Geographic subgroup analysis

**Figure 6:**
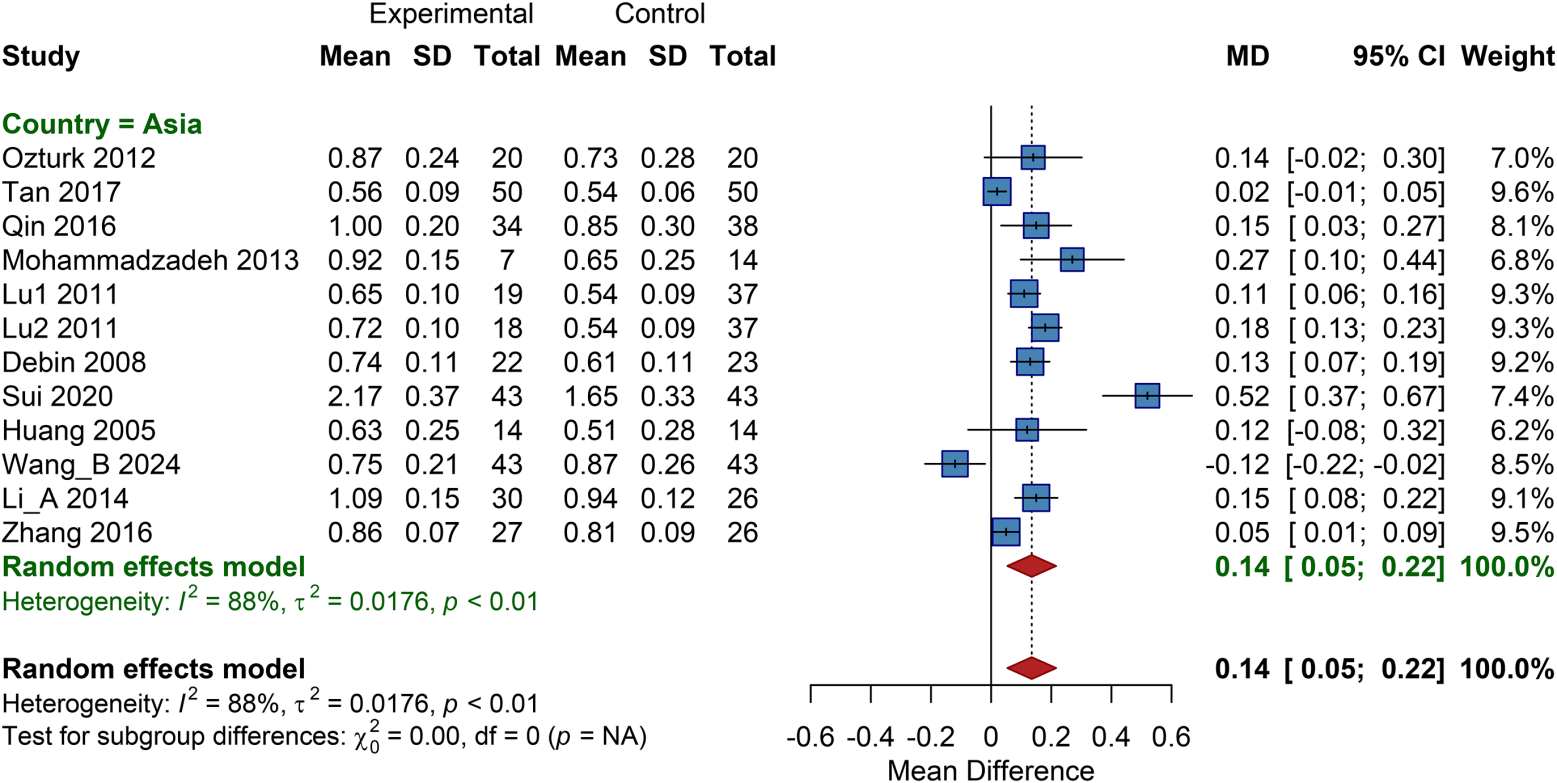
Subgroup analysis of ABI by country, SD, Standard Deviation.

All included studies for this outcome were conducted in Asian regions, therefore only a single-region analysis was performed. The pooled MD was 0.14 (95% CI: 0.05 to 0.22), indicating that cell therapy significantly improves ABI levels in patients from Asian regions (**Figure 6**).

##### 3.6.2.2 Subgroup analysis by cell type

The analysis demonstrated that the autologous BMMSC subgroup had a pooled MD of 0.16 (95% CI: 0.11 to 0.21); the autologous BMMNC subgroup, MD = 0.11 (95% CI: 0.06 to 0.16); and the autologous peripheral blood-derived cell subgroup, MD = 0.09 (95% CI: 0.01 to 0.17). This suggests that various cell types can improve ABI levels, with autologous BMMSCs showing a slightly superior effect (**Supplementary Figure 28**).

##### 3.6.2.3 Subgroup analysis by cell dose

Studies were stratified into five subgroups based on administered cell dose, revealing a significant difference between subgroups. The pooled MD for the high-dose group was 0.16 (95% CI: 0.09 to 0.23); for the very high-dose group, MD = 0.12 (95% CI: -0.08 to 0.32); for the combined high/very high-dose group, MD = 0.13 (95% CI: 0.07 to 0.19); for the low-dose group, MD = 0.33 (95% CI: -0.04 to 0.70); and for the medium-dose group, MD = 0.06 (95% CI: -0.06 to 0.18). The results indicate that high-dose cell therapy yields the optimal effect in improving ABI (**Supplementary Figure 29**).

##### 3.6.2.4 Subgroup analysis by administration route

The analysis showed that the intramuscular injection subgroup had a pooled MD of 0.15 (95% CI: 0.11 to 0.19), with low heterogeneity and significant efficacy. Analyses for the combined therapy and subcutaneous injection subgroups showed no statistically significant differences, suggesting that intramuscular injection is the more effective delivery route for improving ABI (**Supplementary Figure 30**).

##### 3.6.2.5 Other subgroup analyses

In the subgroup analysis by follow-up duration, the subgroup with follow-up<10 months had a pooled MD of 0.14 (95% CI: 0.06 to 0.23), while the subgroup with follow-up ≥10 months had an MD of 0.05 (95% CI: 0.01 to 0.09), suggesting a more pronounced improvement in ABI during short-term follow-up (**Supplementary Figure 31**). Subgroup analysis based on baseline ulcer area showed a pooled MD of 0.14 (95% CI: 0.10 to 0.18) for ulcers<10 cm² and an MD of 0.07 (95% CI: -0.31 to 0.45) for ulcers ≥10 cm² (**Supplementary Figure 32**). Analysis by diabetes duration yielded a pooled MD of 0.19 (95% CI: 0.14 to 0.24) for duration<10 years, MD = 0.19 (95% CI: -0.02 to 0.40) for 10 to 15 years, and MD = 0.13 (95% CI: 0.07 to 0.19) for >15 years, indicating a trend toward more pronounced benefit in patients with shorter disease duration (**Supplementary Figure 33**). For ulcer duration, studies with duration<200 days had a pooled MD of 0.10 (95% CI: -0.05 to 0.26), suggesting that ulcer duration does not significantly impact the improvement in ABI (**Supplementary Figure 34**).

### 3.7 Meta-analysis of TcPO_2_

#### 3.7.1 Overall efficacy analysis

Ten studies included in this analysis reported outcomes related to changes in TcPO_2_. The pooled results from a random-effects model demonstrated a significantly higher TcPO_2_ value in the cell therapy group compared to the control group, with a pooled MD of 11.58 (95% CI: 5.36 to 17.80) (**Supplementary Figure 35**).

#### 3.7.2 Subgroup analysis

##### 3.7.2.1 Geographic subgroup analysis

**Figure 7:**
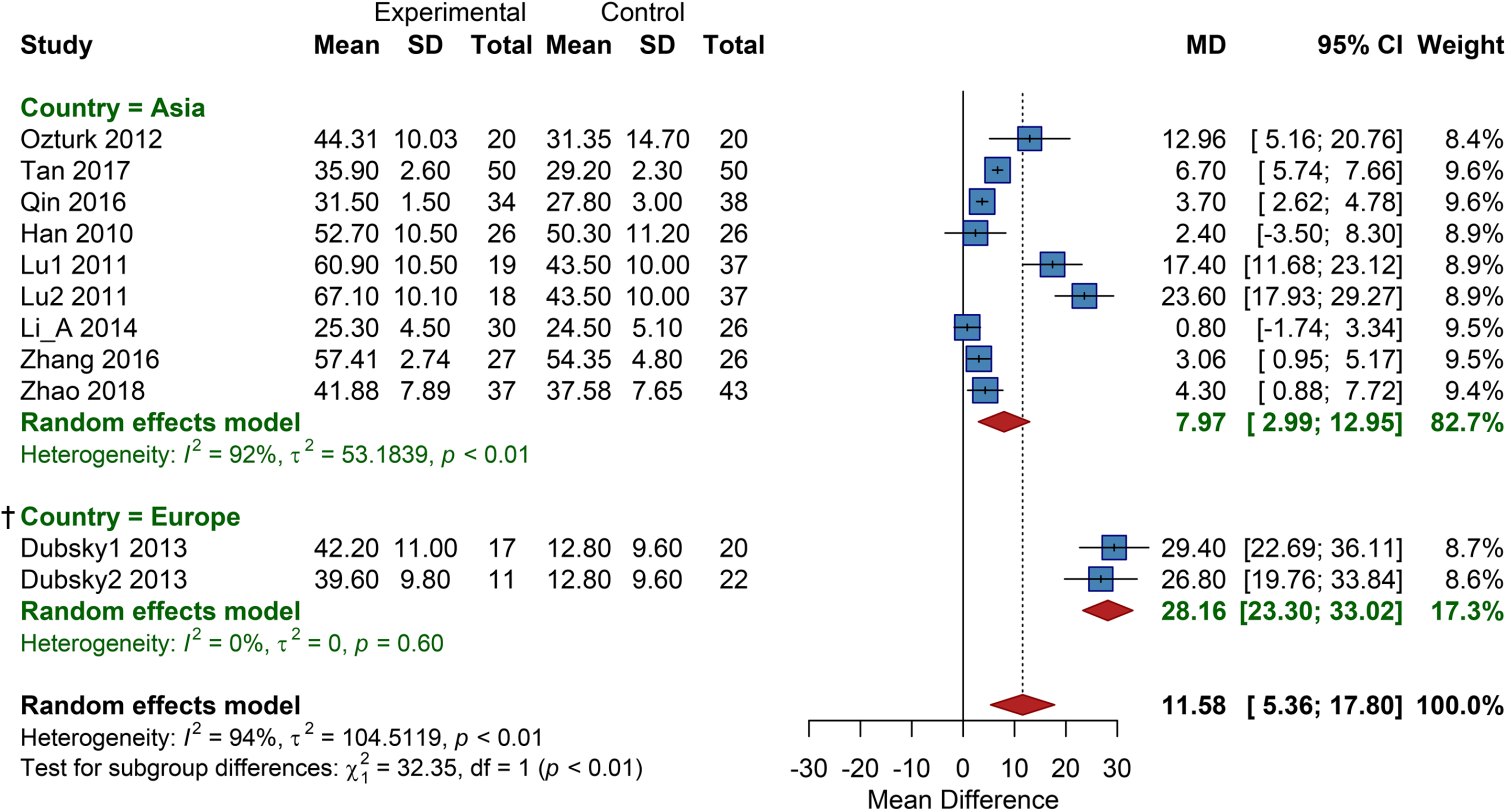
Subgroup analysis of TcPO_2_ by country, SD, Standard Deviation, † Results based on sparse data (number of studies k<3) should be interpreted with caution.

Studies were stratified by region into two subgroups: Asia and Europe. The analysis demonstrated a significant difference between subgroups (*P<*0.01). The pooled MD for the Asian subgroup was 7.97 (95% CI: 2.99 to 12.95), and for the European subgroup, MD = 28.16 (95% CI: 23.30 to 33.02). These results suggest a more pronounced effect of cell therapy on improving TcPO_2_ in European regions, while a clear benefit was also observed in Asia (**Figure 7**).

##### 3.7.2.2 Subgroup analysis by cell type

Subgroup Analysis by Cell Type demonstrated significant differences between subgroups. The autologous BMMSC subgroup showed an MD of 23.60 (95% CI: 17.93 to 29.27); the autologous BMMNC subgroup, a pooled MD of 23.27 (95% CI: 11.51 to 35.02); the autologous peripheral blood-derived cell subgroup, a pooled MD of 11.95 (95% CI: 1.83 to 22.06); the adipose-derived stem cell (ASC) subgroup, a pooled MD of 3.82 (95% CI: 0.86 to 6.78); and the umbilical cord/placental mesenchymal stem cell (UC-MSC/PL-MSC) subgroup, an MD of 2.49 (95% CI: -0.32 to 5.29). These results suggest that autologous bone marrow-derived cells (BMMSCs and BMMNCs) have a more pronounced effect on improving TcPO_2_ (**Supplementary Figure 36**).

##### 3.7.2.3 Subgroup analysis by cell dose

Subgroup Analysis by Cell Dose revealed significant differences. The very high-dose group had a pooled MD of 28.16 (95% CI: 23.30 to 33.02), showing the most significant effect; the high-dose group, a pooled MD of 20.51 (95% CI: 14.44 to 26.59); the medium-dose group, an MD of 2.84 (95% CI: 1.28 to 4.39); the low-dose group, a pooled MD of 5.83 (95% CI: 0.54 to 11.12); and the dose-unknown group, an MD of 6.70 (95% CI: 5.74 to 7.66). The results indicate a clear dose-response relationship, with higher doses leading to more significant improvements in TcPO_2_ (**Supplementary Figure 37**).

##### 3.7.2.4 Subgroup analysis by administration route

Significant differences were found between subgroups by Administration Route. The intramuscular injection subgroup had a pooled MD of 16.07 (95% CI: 7.64 to 24.49); the intra-arterial infusion subgroup, an MD of 3.06 (95% CI: 0.95 to 5.17); and the subcutaneous injection subgroup, a pooled MD of 6.08 (95% CI: 4.03 to 8.14). This suggests that intramuscular injection may be the more effective delivery route for improving TcPO_2_ (**Supplementary Figure 38**).

##### 3.7.2.5 Other subgroup analyses

In the analysis by follow-up duration, the subgroup with follow-up<10 months had a pooled MD of 12.49 (95% CI: 5.83 to 19.14), and the subgroup with follow-up ≥10 months had an MD of 3.06 (95% CI: 0.95 to 5.17). A significant difference existed between subgroups (χ² = 7, df = 1, P<0.01), indicating more significant TcPO_2_ improvement during short-term follow-up (**Supplementary Figure 39**). Subgroup analysis based on baseline ulcer area showed a pooled MD of 19.83 (95% CI: 10.38 to 29.29) for ulcers<10 cm², suggesting significant TcPO_2_ improvement when the baseline ulcer area is smaller (**Supplementary Figure 40**). Analysis by diabetes duration showed a pooled MD of 9.53 (95% CI: 3.44 to 15.63) for a duration of 10 to 15 years, with significant differences between subgroups (**Supplementary Figure 41**). In the ulcer duration subgroup analysis, the subgroup with duration ≤200 days had a pooled MD of 20.51 (95% CI: 14.44 to 26.59), while the subgroup >200 days had an MD of 4.30 (95% CI: 0.88 to 7.72). A significant difference was observed between subgroups, suggesting a more pronounced improvement in patients with shorter ulcer duration (**Supplementary Figure 42**).

### 3.8 Meta-analysis of pain-free walking distance

#### 3.8.1 Overall efficacy analysis

**Figure 8:**
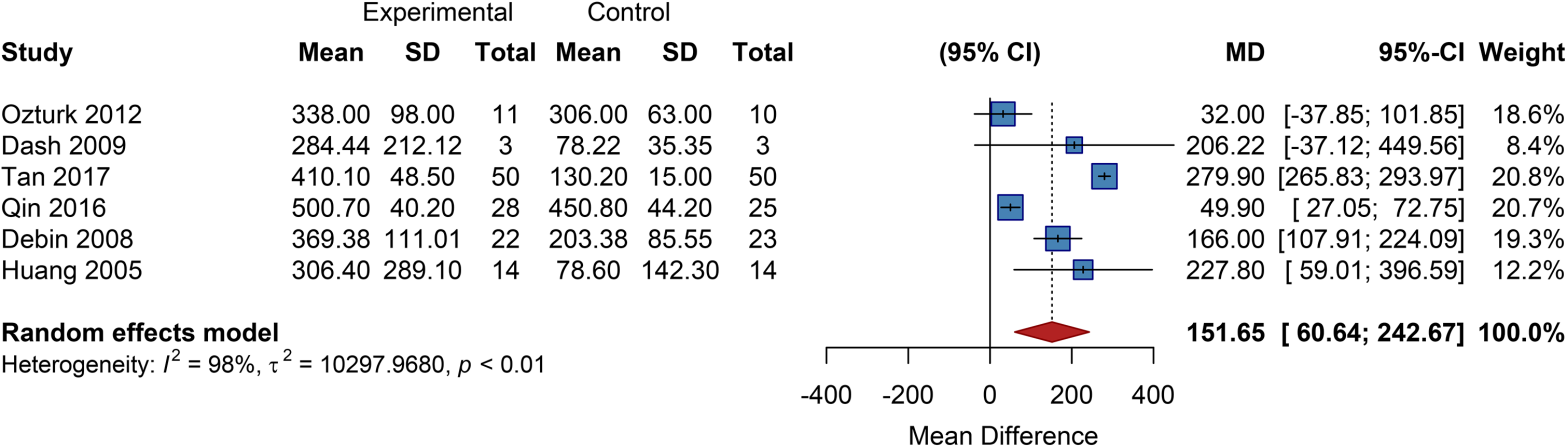
Overall analysis of pain-free walking distance, SD, Standard Deviation.

Six studies included in this analysis reported outcomes related to changes in PFWD. The pooled results from a random-effects model demonstrated a significantly longer PFWD in the cell therapy group compared to the control group, with a pooled MD of 151.65 (95% CI: 60.64 to 242.67) (**Figure 8**).

#### 3.8.2 Subgroup analysis

##### 3.8.2.1 Subgroup analysis by cell type

Subgroup Analysis by Cell Type showed that the autologous peripheral blood-derived cell subgroup had a pooled MD of 178.17 (95% CI: 19.06 to 337.27); the autologous BMMSC subgroup, a pooled MD of 168.17 (95% CI: 111.67 to 224.67); and the UC-MSC/PL-MSC subgroup, an MD of 49.90 (95% CI: 27.05 to 72.75). This suggests that autologous BMMSCs and PBMNCs have a more pronounced effect on improving PFWD, with the former showing low heterogeneity and thus more reliable results (**Supplementary Figure 43**).

##### 2.8.2.2 Subgroup analysis by cell dose

Subgroup Analysis by Cell Dose only included studies related to the medium-dose group, yielding a pooled MD of 51.27 (95% CI: 28.52 to 74.01; *I²* = 36%, *P* = 0.21), indicating no significant heterogeneity (**Supplementary Figure 44**). Due to insufficient data from other dose groups, a multi-dose comparison was not conducted, precluding an assessment of a dose-response relationship. Future studies are needed to provide additional evidence.

##### 3.8.2.3 Subgroup analysis by administration route

Subgroup Analysis by Administration Route stratified studies into intramuscular injection and combined therapy subgroups, revealing a significant difference between them. The combined therapy subgroup had a pooled MD of 172.54 (95% CI: 117.62 to 227.47), while the intramuscular injection subgroup had a pooled MD of 49.42 (95% CI: 27.79 to 71.05). This suggests that combined therapy has a more significant effect on improving PFWD, although intramuscular injection also provides a clear and stable benefit (**Supplementary Figure 45**).

##### 3.8.2.4 Subgroup analysis of diabetes disease course

Subgroup Analysis by Diabetes Duration only included studies with a duration of 10–15 years, yielding a pooled MD of 122.52 (95% CI: -35.30 to 280.35), which was not statistically significant (**Supplementary Figure 46**).

### 3.9 Meta-analysis of pain-free walking claudication score

#### 3.9.1 Overall efficacy analysis

**Figure 9:**
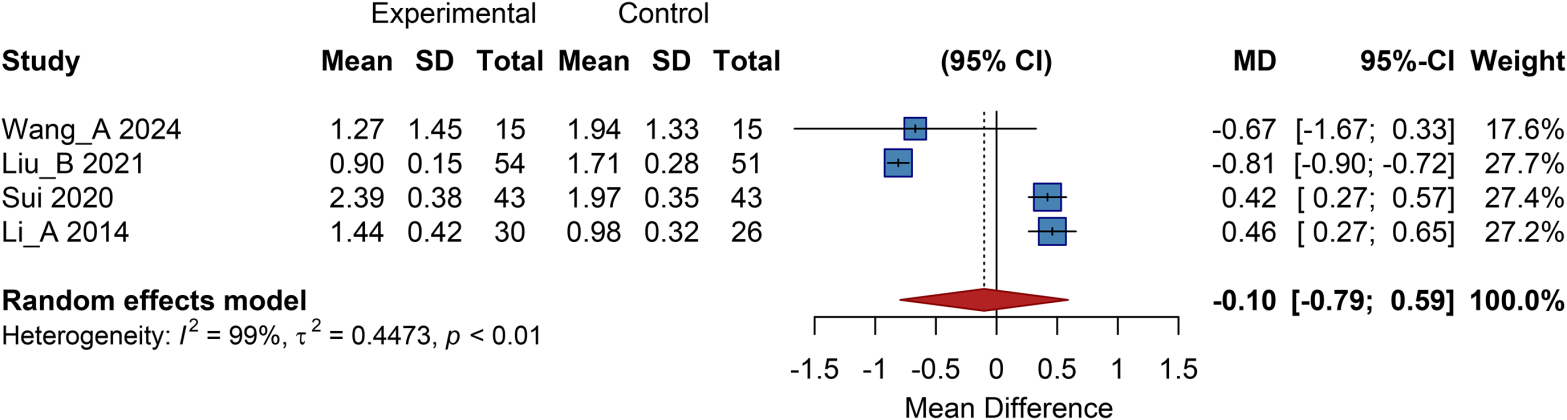
Overall analysis of pain-free walking claudication score, SD, Standard Deviation.

Four studies included in this analysis reported outcomes related to changes in the pain-free walking claudication score. The pooled results from a random-effects model showed no statistically significant difference between the cell therapy group and the control group, with a pooled MD of -0.10 (95% CI: -0.79 to 0.59) (**Figure 9**).

#### 3.9.2 Subgroup analysis

##### 3.9.2.1 Subgroup analysis by cell type

Subgroup Analysis by Cell Type showed no statistically significant difference in the autologous BMMSC subgroup. The UC-MSC/PL-MSC subgroup had a pooled MD of 0.44 (95% CI: 0.31 to 0.56), indicating a significant increase in the score. The autologous peripheral blood-derived cell subgroup had an MD of -0.67 (95% CI: -1.67 to 0.33), showing no statistical difference. This suggests that autologous BMMSCs and PBMNCs are relatively safe, while UC-MSCs/PL-MSCs may be associated with a certain risk of increased claudication scores (**Supplementary Figure 47**).

##### 3.9.2.2 Subgroup analysis by cell dose

In the Subgroup Analysis by Cell Dose, the low-dose group had an MD of 0.42 (95% CI: 0.27 to 0.57); the medium-dose group had a pooled MD of -0.18 (95% CI: -1.42 to 1.07, *P*>0.05); and the dose-unknown group had an MD of -0.67 (95% CI: -1.67 to 0.33). This suggests that low-dose intervention may lead to an increase in the claudication score, while no clear trend was observed for the medium-dose and dose-unknown groups. More data are required to verify any dose-response relationship (**Supplementary Figure 48**).

##### 3.9.2.3 Subgroup analysis by administration route

In the Subgroup Analysis by Administration Route, the combined therapy subgroup had an MD of 0.42 (95% CI: 0.27 to 0.57); the intramuscular injection subgroup had a pooled MD of -0.18 (95% CI: -1.42 to 1.07); and the subcutaneous injection subgroup had a pooled MD of -0.67 (95% CI: -1.67 to 0.33). This indicates that different administration routes have varying effects on the claudication score. Only the combined therapy group showed a significant score increase, while no clear benefit was observed in the intramuscular or subcutaneous injection groups (**Supplementary Figure 49**).

### 3.10 Meta-analysis of resting pain score

#### 3.10.1 Overall efficacy analysis

**Figure 10:**
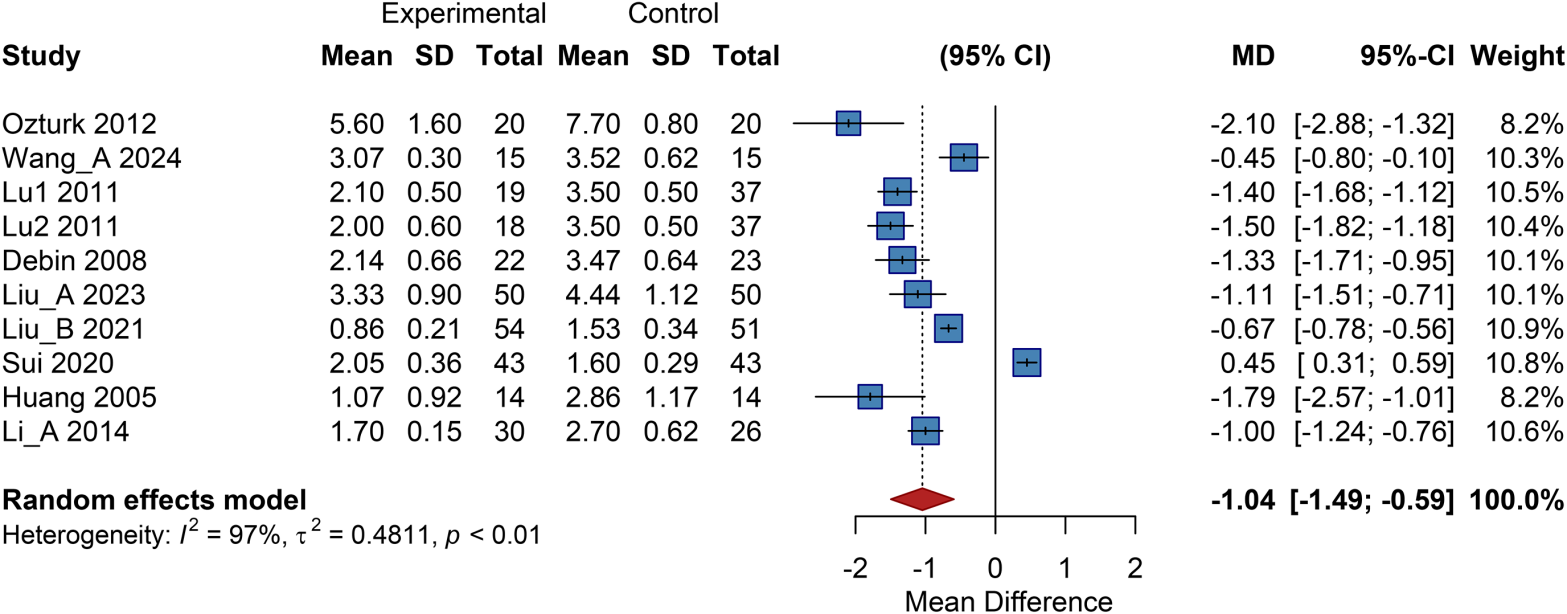
Overall analysis of resting pain score, SD, Standard Deviation.

Nine studies included in this analysis reported outcomes related to changes in resting pain score. The pooled results from a random-effects model demonstrated a significantly lower resting pain score in the cell therapy group compared to the control group, with a pooled MD of -1.04 (95% CI: -1.49 to -0.59; *P* < 0.01) (**Figure 10**).

#### 3.10.2 Subgroup analysis

##### 3.10.2.1 Subgroup analysis by cell type

The autologous peripheral blood-derived cell subgroup showed a pooled MD of -1.39 (95% CI: -2.43 to -0.36); the autologous BMMSC subgroup, MD = -1.13 (95% CI: -1.51 to -0.74); and the UC-MSC/PL-MSC subgroup, MD = -0.27 (95% CI: -1.69 to 1.15). This indicates that PBMNCs and autologous BMMSCs significantly alleviate resting pain, while UC-MSCs/PL-MSCs show no clear benefit (**Supplementary Figure 50**).

##### 3.10.2.2 Cell dose subgroup

The high-dose group demonstrated the most significant effect with low heterogeneity (MD = -1.44, 95% CI: -1.65 to -1.23). The combined high/very high-dose group had an MD of -1.33 (95% CI: -1.71 to -0.95); the medium-dose group, MD = -0.82 (95% CI: -1.14 to -0.49); and the low-dose group, MD = -0.79 (95% CI: -3.29 to 1.70; *P*>0.05). These results suggest that high-dose and combined high/very high-dose cell therapy provides more consistent pain relief, indicating a dose-response trend (**Supplementary Figure 51**).

##### 3.10.2.3 Administration route subgroup

The intramuscular injection subgroup showed a pooled MD of -1.22 (95% CI: -1.56 to -0.88); the subcutaneous injection subgroup, MD = -0.45 (95% CI: -0.80 to -0.10); and the combined therapy subgroup, MD = -0.85 (95% CI: -2.21 to 0.50; *P*>0.05). This suggests that intramuscular injection provides significant and more pronounced relief of resting pain (**Supplementary Figure 52**).

##### 3.10.2.4 Baseline ulcer area subgroup

Stratified into<10 cm² and ≥10 cm² subgroups, a significant difference was observed. The<10 cm² subgroup had a pooled MD of -1.23 (95% CI: -1.58 to -0.87), while the ≥10 cm² subgroup had an MD of -0.67 (95% CI: -0.78 to -0.56; *P* < 0.01). This indicates that patients benefit regardless of ulcer size, with a slightly greater effect observed in those with ulcers<10 cm² (**Supplementary Figure 53**).

##### 3.10.2.5 Diabetes duration subgroup

Stratified into<10 years, 10 to 15 years, and >15 years subgroups, no significant inter-subgroup difference was found. The respective pooled MD s were -1.07 (95% CI: -1.57 to -0.58), -0.98 (95% CI: -2.48 to 0.51), and -1.33 (95% CI: -1.71 to -0.95; *P<*0.01). This demonstrates significant pain relief in patients with both short and long diabetes duration (**Supplementary Figure 54**).

##### 3.10.2.6 Ulcer duration subgroup

Stratified into ≤200 days and >200 days subgroups, a significant difference was observed. The ≤200 days subgroup had a pooled MD of -1.44 (95% CI: -1.65 to -1.23), and the >200 days subgroup had an MD of -0.67 (95% CI: -0.78 to -0.56). This indicates that patients with shorter ulcer duration experience more significant pain relief (**Supplementary Figure 55**).

### 3.11 Meta-analysis of ulcer area

#### 3.11.1 Overall efficacy analysis

**Figure 11:**
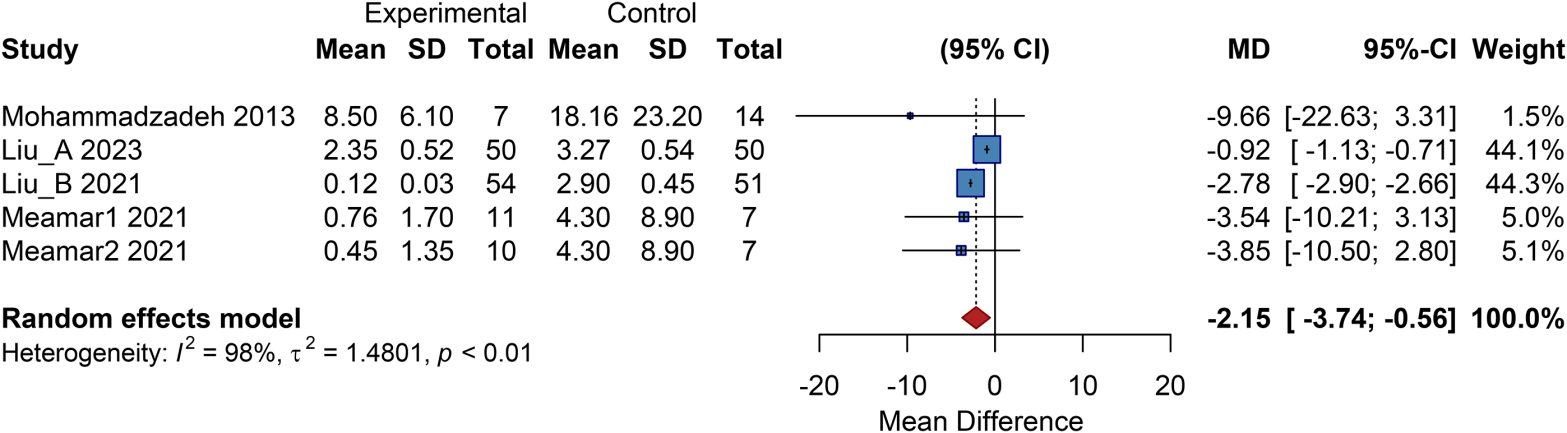
Overall analysis of ulcer area, SD, Standard Deviation.

Four studies included in this analysis reported outcomes related to changes in ulcer area. The pooled results from a random-effects model demonstrated a significantly smaller ulcer area in the cell therapy group compared to the control group, with a pooled mean difference (MD) of -2.15 (95% CI: -3.74 to -0.56; *P<*0.01) (**Figure 11**).

#### 3.11.2 Subgroup analysis

##### 3.11.2.1 Cell type subgroup:

The autologous BMMSC subgroup showed a pooled MD of -1.85 (95% CI: -3.67 to -0.03). The autologous peripheral blood-derived cell subgroup had an MD of -9.66 (95% CI: -22.63 to 3.31), and the UC-MSC/PL-MSC subgroup had a pooled MD of -3.70 (95% CI: -8.40 to 1.01; *P*>0.05). This indicates that only autologous BMMSCs significantly reduced ulcer area, while other cell types showed no clear benefit (**Supplementary Figure 56**).

##### 3.11.2.2 Cell dose subgroup

The medium-dose group had an MD of -2.78 (95% CI: -2.90 to -2.66). The dose-unknown group had an MD of -0.92 (95% CI: -1.13 to -0.71). The high-dose group had an MD of -9.66 (95% CI: -22.63 to 3.31; *P*>0.05), and the low-dose group had a pooled MD of -3.70 (95% CI: -8.40 to 1.01; *P*>0.05). These results suggest that a medium dose is effective in significantly reducing ulcer area (**Supplementary Figure 57**).

##### 3.11.2.3 Administration route subgroup

Studies were stratified into intramuscular injection and topical application subgroups, with no significant difference between them. The intramuscular injection subgroup had a pooled MD of -2.00 (95% CI: -3.82 to -0.18), while the topical application subgroup had a pooled MD of -3.70 (95% CI: -8.40 to 1.01; *P*>0.05). This indicates that intramuscular injection significantly reduces ulcer area, whereas topical application shows no clear benefit (**Supplementary Figure 58**).

##### 3.11.2.4 Follow-up duration subgroup

Stratified into<10 months and ≥10 months subgroups. The<10 months subgroup had a pooled MD of -2.00 (95% CI: -3.82 to -0.18), and the ≥10 months subgroup had a pooled MD of -3.70 (95% CI: -8.40 to 1.01). This suggests that ulcer area reduction can be observed in short-term follow-up, while long-term effects require further data for validation (**Supplementary Figure 59**).

##### 3.11.2.5 Baseline ulcer area subgroup

Stratified into <10 cm² and ≥10 cm² subgroups, a highly significant difference was observed. The <10 cm² subgroup had an MD of -0.92 (95% CI: -1.13 to -0.71; *P<*0.01), and the ≥10 cm² subgroup had a pooled MD of -2.78 (95% CI: -2.90 to -2.66; *P<*0.01). This indicates benefit for patients across ulcer sizes, with a more pronounced reduction observed in larger ulcers (≥10 cm²) (**Supplementary Figure 60**).

##### 3.11.2.6 Diabetes duration subgroup

Stratified into <10 years and 10 to 15 years subgroups. The <10 years subgroup had a pooled MD of -2.09 (95% CI: -3.78 to -0.40), and the 10 to 15 years subgroup had a pooled MD of -3.54 (95% CI: -10.21 to 3.13). This suggests a significant reduction in ulcer area for patients with short disease duration, while conclusions for the intermediate-duration group should be drawn cautiously (**Supplementary Figure 61**).

##### 3.11.2.7 Ulcer duration subgroup

Stratified into ≤200 days and >200 days subgroups, with no significant difference between them. The >200 days subgroup had an MD of -2.78 (95% CI: -2.90 to -2.66), and the ≤200 days subgroup had a pooled MD of -4.39 (95% CI: -8.82 to 0.03). This indicates a significant reduction in long-term non-healing ulcers (>200 days), with a trend toward benefit in shorter-duration ulcers approaching statistical significance (**Supplementary Figure 62**).

### 3.12 Meta-analysis of ulcer healing time

#### 3.12.1 Overall efficacy analysis

**Figure 12:**
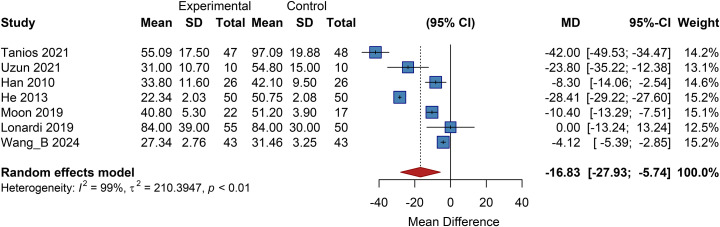
Overall analysis of ulcer healing time, SD, Standard Deviation.

Seven studies included in this analysis reported outcomes related to ulcer healing time. The pooled results from a random-effects model demonstrated a significantly shorter ulcer healing time in the cell therapy group compared to the control group, with a pooled mean difference (MD) of -16.83 (95% CI: -27.93 to -5.74; *P<*0.01) (**Figure 12**).

#### 3.12.2 Subgroup analysis

##### 3.12.2.1 Geographic subgroup analysis

Studies were stratified by region into Africa, Asia, and Europe, revealing significant inter-subgroup differences. The African subgroup demonstrated an MD of -42.00 (95% CI: -49.53 to -34.47), and the Asian subgroup had a pooled MD of -14.68 (95% CI: -24.09 to -5.28), indicating that cell therapy significantly shortens healing time in these regions (**Supplementary Figure 63**).

##### 3.12.2.2 Cell type subgroup analysis

Stratified into UC-MSCs/PL-MSCs and ASCs subgroups. The UC-MSC/PL-MSC subgroup had a pooled MD of -16.27 (95% CI: -40.07 to 7.53), while the ASC subgroup showed a pooled MD of -17.06 (95% CI: -31.37 to -2.75), indicating a significant reduction in healing time for the latter (**Supplementary Figure 64**).

##### 3.12.2.3 Cell dose subgroup analysis

The low-dose group had a pooled MD of -12.58 (95% CI: -19.97 to -5.18). The medium-dose group had a pooled MD of -16.27 (95% CI: -40.07 to 7.53; *P*>0.05), and the dose-unknown group had a pooled MD of -21.37 (95% CI: -62.52 to 19.79; *P*>0.05). This suggests a stable effect of low-dose therapy in shortening healing time (**Supplementary Figure 65**).

##### 3.12.2.4 Administration route subgroup analysis

The topical application subgroup had a pooled MD of -9.98 (95% CI: -12.56 to -7.40). The intramuscular injection subgroup showed an MD of -28.41 (95% CI: -29.22 to -27.60). The subcutaneous injection subgroup had a pooled MD of -17.61 (95% CI: -36.71 to 1.50; *P*>0.05). This indicates that both topical application and intramuscular injection significantly reduce healing time, with the latter showing a more pronounced effect (**Supplementary Figure 66**).

##### 3.12.2.5 Follow-up duration subgroup analysis

Stratified into <10 months and ≥10 months subgroups, with no significant difference between them. The <10 months subgroup had a pooled MD of -16.82 (95% CI: -32.36 to -1.29), and the ≥10 months subgroup had a pooled MD of -15.91 (95% CI: -28.84 to -2.99). This suggests that the reduction in healing time is observable across different follow-up periods and appears sustained (**Supplementary Figure 67**).

##### 3.12.2.6 Baseline ulcer area subgroup analysis

Stratified into<10 cm² and ≥10 cm² subgroups. The <10 cm² subgroup had a pooled MD of -20.05 (95% CI: -41.21 to 1.11), and the ≥10 cm² subgroup had a pooled MD of -18.49 (95% CI: -33.76 to -3.22), indicating a significant reduction in healing time for patients with larger ulcers (**Supplementary Figure 68**).

##### 3.12.2.7 Diabetes duration subgroup analysis

Stratified into 10 to 15 years and >15 years subgroups. The 10 to 15 years subgroup showed an MD of -23.80 (95% CI: -35.22 to -12.38), and the >15 years subgroup had a pooled MD of -19.46 (95% CI: -37.11 to -1.81), suggesting a significant shortening of healing time across these disease durations (**Supplementary Figure 69**).

##### 3.12.2.8 Ulcer duration subgroup analysis

Stratified into ≤200 days and >200 days subgroups. The ≤200 days subgroup had a pooled MD of -13.11 (95% CI: -32.32 to 6.11), and the >200 days subgroup had a pooled MD of -26.00 (95% CI: -56.97 to 4.96), with neither showing statistical significance (**Supplementary Figure 70**).

## 4 Analysis of bias, sensitivity, heterogeneity and meta-regression

### 4.1 Bias analysis

**Figure 13:**
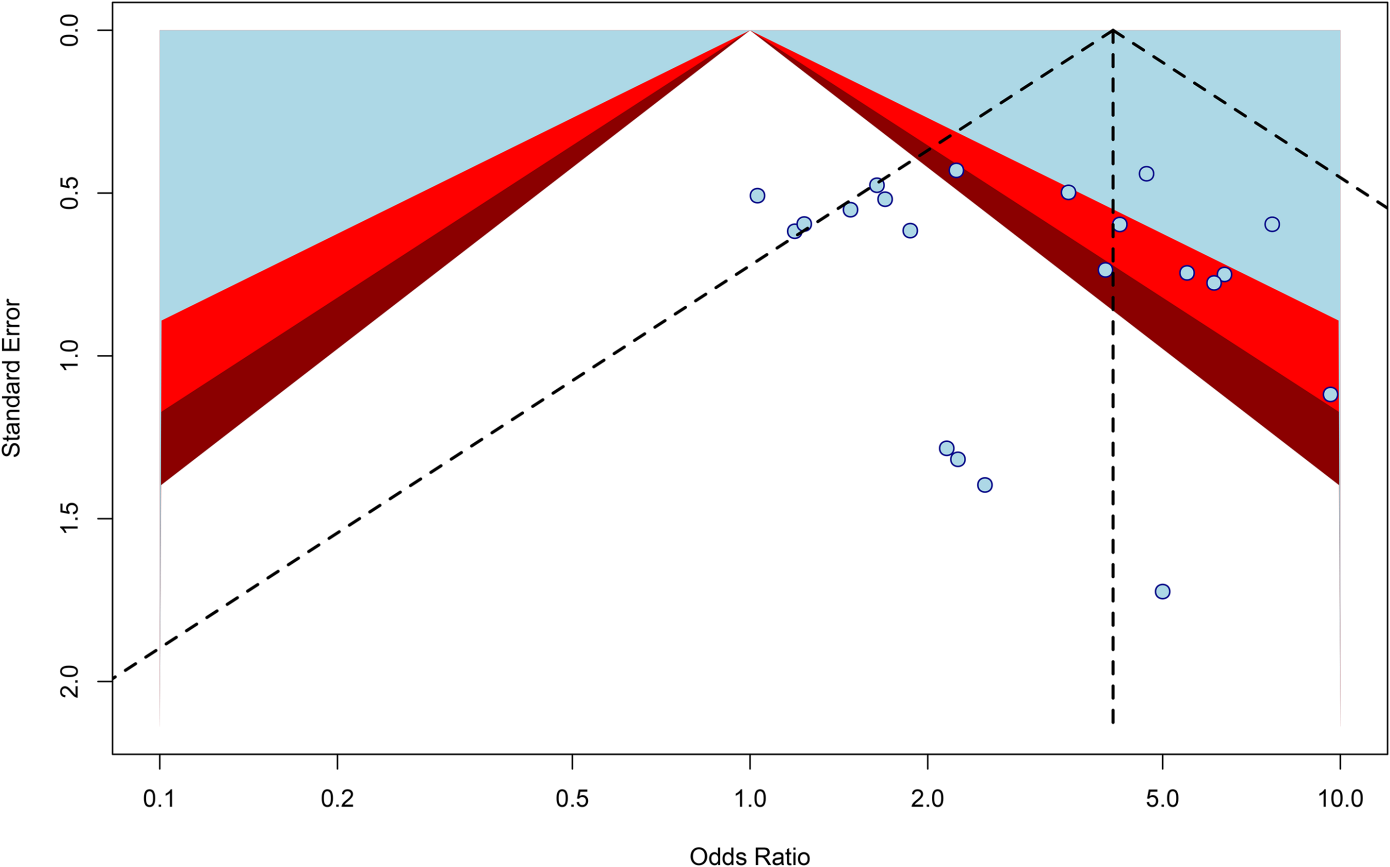
Ulcer healing rate funnel chart.

Publication bias for the core outcomes was assessed using funnel plot symmetry in conjunction with methodological quality evaluation. The overall risk of bias was deemed acceptable. Funnel plots for amputation rate, ABI, and resting pain score demonstrated no evidence of significant publication bias. However, visual inspection of the funnel plot for ulcer healing rate (**Figure 13**) revealed mild asymmetry with a deficit of smaller studies reporting negative results, suggesting the possible presence of small-study bias. For ulcer area and ulcer healing time, the limited number of included studies resulted in funnel plots exhibiting slight asymmetry, though no extreme outliers were present, indicating a moderate risk of publication bias. No significant methodological bias was identified across all outcomes. Collectively, the observed biases are unlikely to substantially compromise the credibility of the conclusions (see **Supplementary Figures 71**–**78**).

### 4.2 Meta-regression analysis

To systematically investigate potential sources of heterogeneity, we performed univariate meta-regression analyses for all outcomes with sufficient data (≥3 studies). The complete results are provided in **Supplementary Table 4**. For the ulcer healing rate, diabetes duration (*P =* 0.006) emerged as a significant moderator. Heterogeneity in the amputation rate was primarily influenced by the year of publication (*P* < 0.001), country (*P =* 0.004), and cell type (*P =* 0.037). The heterogeneity observed in TcPO_2_ outcomes was significantly associated with cell dose (*P =* 0.001) and baseline ulcer area (*P* = 0.010). Baseline ulcer area (*P<*0.001) was a significant moderator for the reduction in ulcer area, while the pain-free walking distance was influenced by the route of administration (*P* = 0.018). For outcomes with an insufficient number of studies for meta-regression (e.g., neovascularization score), we validated the robustness of the pooled effect estimates through sensitivity analyses.

### 4.3 Sensitivity analysis

**Figure 14:**
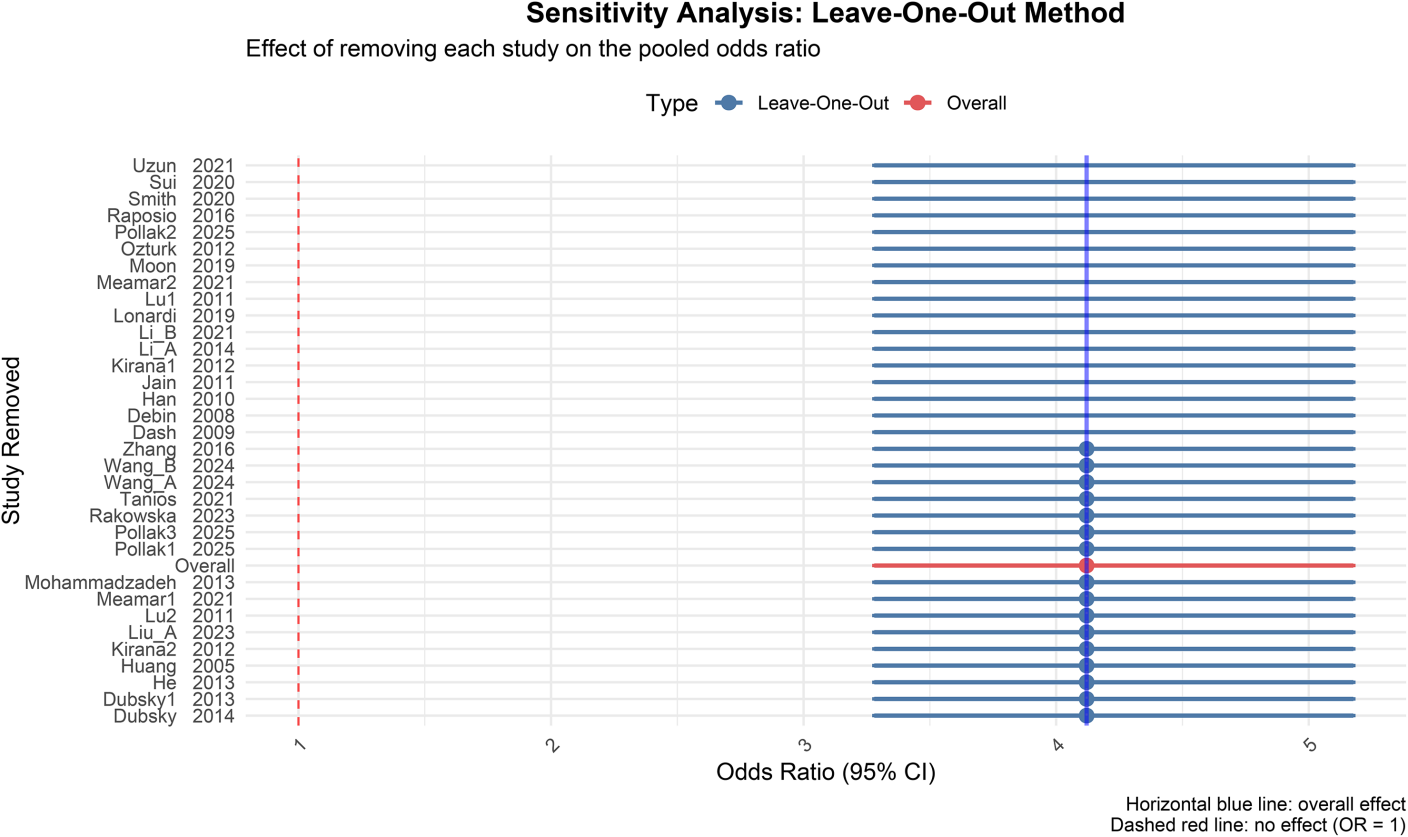
Sensitivity analysis of ulcer healing rate.

A leave-one-out sensitivity analysis was conducted to verify the robustness of the results. The pooled effect sizes for all outcomes exhibited no significant fluctuation: the pooled OR for ulcer healing rate ranged from 4.12 to 5.03 (**Figure 14**); for amputation rate, from 0.22 to 0.34; the pooled MD for ABI ranged from 0.10 to 0.18; for TcPO_2_, from 8.21 to 15.36; for resting pain score, from -1.28 to -0.81; for ulcer area, from -3.21 to -1.58; and for ulcer healing time, from -22.15 to -12.38. Furthermore, all 95% confidence intervals within these ranges excluded the null value (OR = 1 or MD = 0). No single study was found to dominantly influence the pooled effect estimates, indicating that the core conclusions of this meta-analysis are stable and not unduly influenced by any individual trial (see **Supplementary Figures 79**–**86**).

### 4.4 Heterogeneity analysis

**Figure 15:**
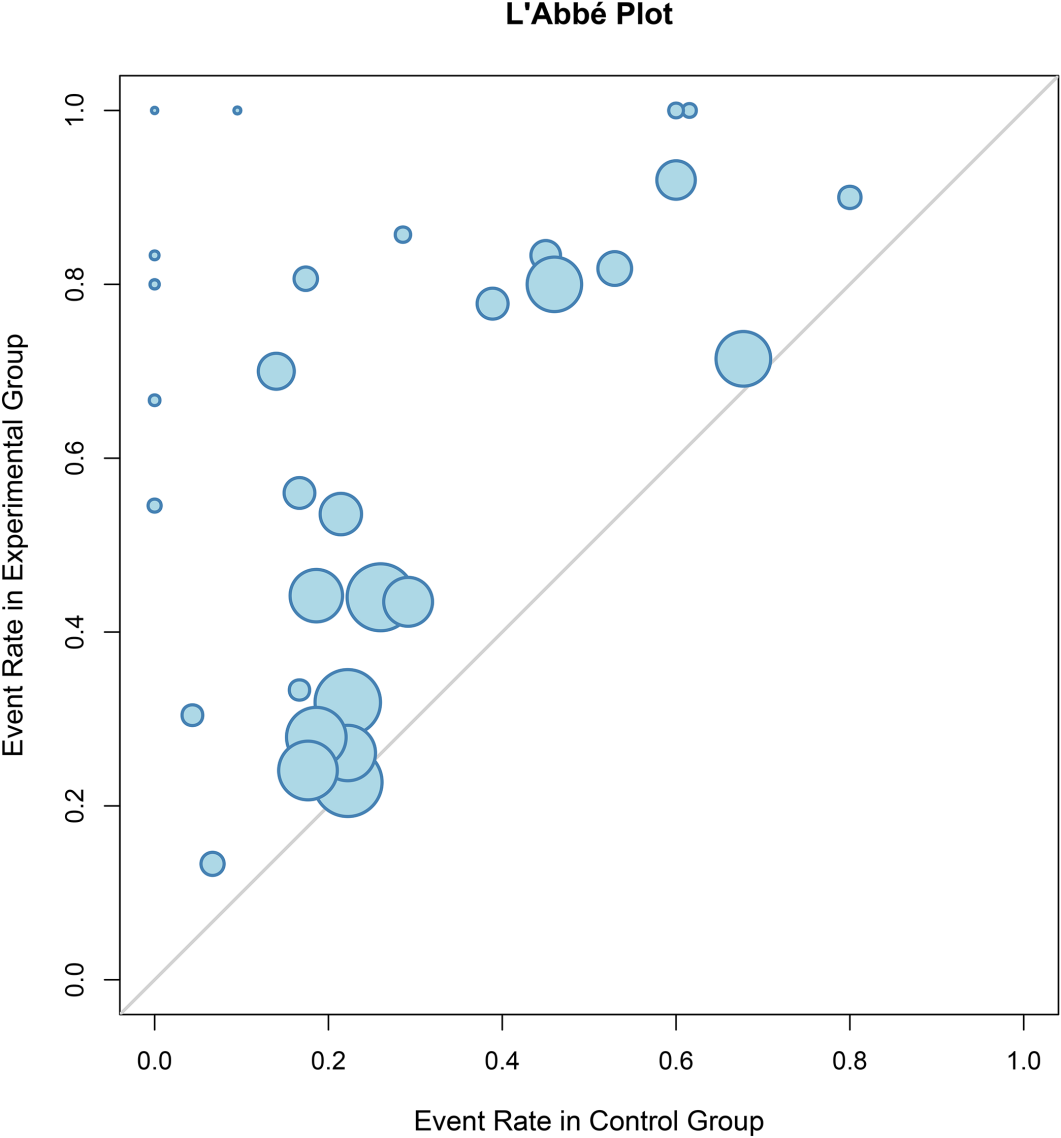
Heterogeneity test of ulcer healing rate.

The characteristics and sources of heterogeneity were delineated through a combined assessment of the *I²* statistic, Baujat plots, and subgroup analyses. The amputation rate demonstrated no statistical heterogeneity (*I²* = 0%), indicating strong consistency across studies. Moderate heterogeneity was observed for ulcer healing rate (*I²* = 53%) and neovascularization rate *(I²* = 50%), which was primarily attributed to geographic variations (non-significant effects in the North American subgroup) and cell dose stratification (**Figure 15**). High levels of heterogeneity were present for ABI (*I²* = 88%), TcPO_2_ (*I²* = 94%), pain-free walking distance (*I²* = 98%), resting pain score (*I²* = 97%), ulcer area (*I²* = 98%), and ulcer healing time (*I²* = 99%). Baujat plots identified studies such as Sui 2020 (ABI), LiA 2014 (TcPO_2_), and LiuB 2021 (pain-free walking claudication score) as major contributors. Further subgroup analysis revealed that administration route, cell dose, and baseline ulcer area were the core sources of this heterogeneity. Stratification substantially reduced within-subgroup heterogeneity. Crucially, the observed heterogeneity did not alter the core conclusion that cell therapy is superior to the control group across all outcomes, indicating that the overall heterogeneity is manageable (see **Supplementary Figures 87**–**95**).

## 5 Discussion

The incidence of diabetic foot disease is rising annually, imposing a substantial burden on the global diabetic population. It is estimated that chronic ulcers develop in approximately 30% to 50% of patients with diabetic foot, with an annual healing rate of less than 50%. This directly contributes to a 20- to 30-fold higher risk of amputation compared to the general population. Notably, the five-year mortality rate following a diabetic foot-related amputation ranges from 40% to 60%, a prognosis worse than that of many malignancies (50, 51). Although conventional comprehensive clinical management offers some benefit, it remains inadequate for refractory ulcers complicated by severe limb ischemia, neuropathy, or chronic infection, often resulting in slow healing, high recurrence rates, and poorly controlled amputation rates (52). While revascularization procedures can improve perfusion, they are only suitable for fewer than 60% of patients with adequate vascular anatomy and are associated with 30% to 40% rates of restenosis (53). Novel adjuncts like platelet-rich plasma (PRP) and negative pressure wound therapy (NPWT) may accelerate local repair but fail to reverse the distal ischemic-hypoxic microenvironment, offering limited clinical benefit (54, 55).

While cell-based therapy has introduced a novel paradigm for treating DFU, existing evidence remains subject to significant limitations. First, previous meta-analyses have predominantly focused on isolated outcomes, failing to comprehensively integrate core prognostic indicators—such as amputation rates, perfusion parameters (ABI, TcPO_2_), and pain relief—thereby obscuring the therapy’s full clinical value. Second, subgroup analyses have often been superficial, typically limited to geographic stratification, without probing the interactions between cell dosage, delivery routes, and baseline patient characteristics (e.g., ulcer size, disease duration); consequently, optimal therapeutic scenarios remain undefined. Third, prior studies have largely failed to systematically address sources of heterogeneity and often relied on small sample sizes, resulting in a low overall quality of evidence and limited generalizability. To address these deficiencies, this study synthesizes data from 32 RCTs involving 2,059 patients to systematically evaluate therapeutic efficacy, explore potential therapeutic strategies, and provide evidence-based insights to guide clinical practice and future research.

This study demonstrates that cell-based therapy increases the ulcer healing rate by 4.64-fold (OR = 4.64, P<0.001) and reduces the amputation rate by 71% (OR = 0.29, *P* < 0.001). These findings align with a meta-analysis by Sun et al. (2022), which included 14 studies with 683 patients, reporting that stem cell therapy significantly improved ulcer healing (OR = 8.20, 95% CI: 5.33 to 12.62), reduced amputation risk (OR = 0.19, 95% CI: 0.10 to 0.36), and effectively enhanced ABI, TcPO_2_, pain-free walking distance, and resting pain scores compared to conventional therapy (15). The present analysis extends the evidence by not only validating benefits in healing and amputation but also demonstrating through subgroup analyses that cell therapy significantly improves limb perfusion (ABI MD = 0.14, *P<*0.01; TcPO_2_ MD = 11.58, *P* < 0.01), shortens healing time (MD = -16.83 days, *P<*0.01), and alleviates resting pain (MD = -1.04, *P* < 0.01).

Notably, our exploratory subgroup analysis suggests that cell therapy may exhibit more pronounced efficacy for large ulcers (≥10 cm²) and prolonged non-healing ulcers (>200 days) (ulcer area MD = -2.78 vs. -0.92). This observation contrasts with some studies, such as that by Adam DJ et al., which suggested that severe ischemia or large ulcer size might impair stem cell survival and function, potentially limiting therapeutic benefit (56). This discrepancy may be attributed to heterogeneity in patient baseline characteristics. The present meta-analysis exclusively included patients with chronic ulcers, whose endogenous repair mechanisms are severely compromised and whose local microenvironment is often in a state of chronic inflammation (57). Exogenously administered stem cells, through paracrine actions, provide a rich source of cytokines such as VEGF and bFGF. Crucially, as highlighted in recent mechanistic reviews, MSCs exert potent anti-inflammatory and immunomodulatory effects that facilitate tissue repair in chronic inflammatory environments (58). This effectively modulates local immunity and promotes angiogenesis, thereby disrupting the vicious cycle of “inflammation-ischemia” (59). Furthermore, efficacy appears to be linked to cell type, dose, and delivery route. Evidence suggests that intramuscular injection directly into the ischemic zone may ensure better local cell retention and viability compared to intra-arterial infusion (60). Current data indicate that a protocol combining high dose (TcPO_2_ MD = 20.51, *P* < 0.01) with intramuscular injection (ulcer healing OR = 6.79, *P* < 0.01) shows potential for improving therapeutic outcomes, offering a promising direction for treating refractory large ulcers unresponsive to conventional therapy.

Moreover, this study suggests that cell therapy significantly promotes neovascularization (OR = 15.36, *P* < 0.001), a mechanism underpinning its long-term benefits. Although this large effect size is accompanied by a wide confidence interval (95% CI: 4.62–51.09) due to the limited number of studies, sensitivity analyses confirmed that the direction of the pro-angiogenic benefit remained consistent. By secreting factors like VEGF and Ang-1 to foster functional angiogenesis, cell therapy not only improves local perfusion but also reverses neural ischemia and hypoxia. This aligns with our findings of concurrent improvement in ABI, TcPO_2_, and pain symptoms, suggesting that cell therapy addresses the wound-healing deficit not merely by accelerating closure but by fundamentally ameliorating the pathological limb microcirculation at its root (61, 62).

Prespecified subgroup analyses in this study identified three key influencing factors—cell type, treatment protocol, and patient baseline characteristics—providing a foundation for stratified clinical management. Regarding cell type selection, our findings suggest that autologous BMMSCs may represent a potentially favorable subtype, although this warrants further confirmation. They demonstrated superior outcomes across three core endpoints: ulcer healing rate (OR = 8.33, *P* < 0.01), amputation reduction (OR = 0.14, *P* < 0.05), and ulcer area reduction (MD = -1.85, *P* < 0.05). This aligns with basic research by Fu et al. (63), which indicates that autologous BMMSCs possess enhanced immunocompatibility, homing capacity, and paracrine activity. Their secreted exosomes can accelerate wound repair by modulating macrophage M2 polarization and inhibiting the NF-κB inflammatory pathway. In contrast, UC-MSCs/PL-MSCs did not demonstrate a clear statistical benefit for outcomes such as amputation rate (OR = 4.33, *P*>0.05) and claudication score (MD = 0.44, *P* < 0.01), potentially due to allogeneic immune reactions and variable cell viability, though further head-to-head trials are needed to confirm these disparities (64, 65).

Our analysis indicates a potential dose-dependent relationship in the treatment response. While higher and ultra-high doses demonstrated the strongest theoretical efficacy—particularly in reducing amputation risk (OR = 0.11 to 0.17) and improving transcutaneous oxygen pressure (TcPO_2_)—the intermediate dose group showed greater statistical robustness, with narrower confidence intervals and consistent effectiveness across multiple secondary outcomes, such as a significant reduction in ulcer area (MD = −2.78, *P* < 0.01). Therefore, rather than recommending a single fixed dose, we propose an “intermediate-to-high dose range” (approximately 1×10^7^ to 1.2×10^9^ cells) as a pragmatic therapeutic window. This range balances the efficacy observed at higher doses with the statistical stability, feasibility, and cost-effectiveness associated with intermediate dosing. Regarding the route, intramuscular injection consistently yielded favorable results for ulcer healing rate (OR = 6.79, *P* < 0.01) and ABI improvement (MD = 0.15, *P* < 0.01). Intra-arterial infusion, limited by vascular anatomy and more invasive, appeared less effective. Intramuscular injection directly delivers stem cells to ischemic muscle tissue, facilitating microvascular repair and is particularly suitable for refractory patients with poor vascular status.

Subgroup analysis of patient baseline characteristics further revealed a trend suggesting that patients with a shorter diabetes duration (<10 years) might derive more significant benefit in neovascularization rate (OR = 131.21, *P* < 0.01) and ABI improvement (MD = 0.19, *P* < 0.01). In contrast, patients with a longer disease duration (>15 years) showed prominent improvement in TcPO_2_ (MD = 28.16, *P* < 0.01), possibly because their more severe ischemia-hypoxia microenvironment allows the paracrine effects of stem cells to become more evident (66). Geographic subgroup analysis indicated more pronounced efficacy in Asian and African regions (healing time MD = -14.68 and -42.00, respectively), whereas the North American subgroup showed no clear benefit. This may be related to a higher comorbidity burden (e.g., obesity, cardiovascular disease) and differences in treatment adherence in North American populations, although these associations require validation in multi-regional studies.

This meta-analysis predominantly included data from Asian studies. This distribution pattern likely reflects regional disparities in clinical practice patterns. In Western healthcare systems, standard care often incorporates aggressive revascularization procedures, which may result in a higher baseline healing rate and could potentially obscure the incremental therapeutic value of cell-based interventions. Conversely, in settings where access to revascularization is limited, the benefits of cell therapy may be more pronounced. From a biological perspective, the pro-angiogenic pathways through which cell therapy exerts its effects are conserved across ethnic groups. Therefore, the core therapeutic efficacy of this approach in improving perfusion is likely generalizable to a global population.

To further explore the observed heterogeneity, meta-regression analysis in this study confirmed that diabetes duration and cell dose are significant factors influencing ulcer healing and perfusion outcomes, respectively, suggesting the importance of patient stratification and dose optimization in clinical practice. However, substantial residual heterogeneity persists in some analyses. This likely originates from methodological variations across trials, such as differences in the assessment of cell viability, injection techniques, and the consistency of background standard care among medical centers. These difficult-to-quantify factors reflect the inherent complexity in translating cell therapy into clinical practice and highlight the urgent need to establish a standardized reporting framework in future research.

This study possesses notable methodological strengths. By incorporating 32 RCTs and evaluating nine key outcomes, it provides more comprehensive evidence than previous analyses. The use of Baujat plots to identify heterogeneity sources, combined with subgroup analyses, clarified moderating factors such as cell dose, administration route, and ulcer area, substantially reducing within-subgroup heterogeneity. Bias assessment indicated that the majority of studies had complete reporting and data, with only a few outcomes having a potential moderate risk of bias. Sensitivity analysis confirmed the stability of the pooled results, with no single study dominating the conclusions, supporting high evidence reliability.

## 6 Limitations and future research directions

This study has several limitations that warrant careful consideration. First, the absence of double-blinding or allocation concealment in some included trials may have introduced performance and detection biases, potentially overestimating treatment effects on subjective outcomes such as resting pain and pain-free walking distance. Second, the evidence is predominantly derived from Asian populations; this geographical imbalance limits the generalizability of findings to Western cohorts, and regional differences may act as confounding factors in subgroup analyses. Third, although subgroup analyses (e.g., by cell type or dosage) indicated statistical significance, these findings are observational and based on a limited number of studies accompanied by wide confidence intervals; thus, they should be interpreted as exploratory and hypothesis-generating rather than definitive. Fourth, the relatively short follow-up duration in current studies necessitates further validation of the intervention’s long-term durability and safety. Fifth, despite our efforts to explore sources of variation, residual heterogeneity persisted in some efficacy outcomes, likely reflecting methodological diversity in cell preparation and background standard care protocols. Furthermore, funnel plot asymmetry suggests potential publication bias, indicating that smaller trials with negative results may remain unpublished. Finally, due to the scarcity of reported adverse events—particularly cardiovascular or renal complications—a robust quantitative meta-analysis of safety outcomes was not feasible.

To address these limitations and better guide clinical practice, future research should prioritize rigorous, high-quality RCTs incorporating strict double-blinding and allocation concealment to minimize methodological bias. It is imperative to expand investigations to non-Asian populations through multi-center international collaborations, thereby validating the generalizability of findings across diverse genetic backgrounds and healthcare systems. Furthermore, future trials should employ head-to-head comparisons to determine optimal cell types and dosages, alongside extended follow-up periods to evaluate the long-term durability and safety of the intervention. Finally, establishing standardized and systematic protocols for adverse event reporting is crucial to mitigate publication bias and facilitate a comprehensive safety assessment.

## 7 Conclusion

This systematic review and meta-analysis suggests that cell therapy, particularly utilizing autologous BMMSCs administered via intramuscular injection, effectively promotes the healing of DFUs and reduces the risk of amputation. The treatment also demonstrates promising efficacy across multiple dimensions, including improving limb perfusion (ABI and TcPO_2_), alleviating pain, and shortening healing time. Furthermore, exploratory subgroup analyses identified potential characteristics of patients who may derive greater benefit, including those with refractory ulcers characterized by large size (≥10 cm²), short diabetes duration (<10 years), or long-term non-healing (>200 days). The core mechanism of action appears to be closely associated with promoting functional neovascularization and ameliorating the local ischemic-hypoxic microenvironment. However, given the limitations in current evidence quality, these findings warrant further validation through large-scale, standardized RCTs.

## Data Availability

The original contributions presented in the study are included in the article/**Supplementary Material**. Further inquiries can be directed to the corresponding author.
