# Supplementary material for: Bone marrow-derived mesenchymal stem cells combined with intramuscular injection promote the healing of diabetic foot ulcers: a systematic review and meta-analysis
Source: Front Endocrinol (Lausanne). 2026 Mar 10;17:1763071. doi: 10.3389/fendo.2026.1763071 (PMC13008641; doi:10.3389/fendo.2026.1763071)
Supplement: Supplementary file 1 [file DataSheet1.docx]

Supplementary Materials

## Table S1 Literature Search Strategy

| Pubmed: 707 | ((((((((((((((((((((stem cell[MeSH Terms]) OR (Cells, Stem[Title/Abstract])) OR (Cell, Stem[Title/Abstract])) OR (Stem Cell[Title/Abstract])) OR (Mother Cells[Title/Abstract])) OR (Cell, Mother[Title/Abstract])) OR (Cells, Mother[Title/Abstract])) OR (Mother Cell[Title/Abstract])) OR (Progenitor Cells[Title/Abstract])) OR (Cell, Progenitor[Title/Abstract])) OR (Cells, Progenitor[Title/Abstract])) OR (Progenitor Cell[Title/Abstract])) OR (Colony-Forming Unit[Title/Abstract])) OR (Colony Forming Unit[Title/Abstract])) OR (Colony-Forming Units[Title/Abstract])) OR (Colony Forming Units[Title/Abstract])) OR (((((((bone marrow[MeSH Terms]) OR (Marrow, Bone[Title/Abstract])) OR (Red Marrow[Title/Abstract])) OR (Marrow, Red[Title/Abstract])) OR (Yellow Marrow[Title/Abstract])) OR (Marrow, Yellow[Title/Abstract])) OR (Marrow[Title/Abstract]))) OR (((((((((Leukocytes, Mononuclear[MeSH Terms]) OR (Mononuclear Leukocyte[Title/Abstract])) OR (Leukocyte, Mononuclear[Title/Abstract])) OR (Mononuclear Leukocytes[Title/Abstract])) OR (Peripheral Blood Mononuclear Cells[Title/Abstract])) OR (Peripheral Blood Mononuclear Cell[Title/Abstract])) OR (Peripheral Blood Human Mononuclear Cells[Title/Abstract])) OR (PBMC Peripheral Blood Mononuclear Cells[Title/Abstract])))) OR (((((((((Adipocytes[MeSH Terms]) OR (Adipocyte[Title/Abstract])) OR (Fat Cells[Title/Abstract])) OR (Cell, Fat[Title/Abstract])) OR (Cells, Fat[Title/Abstract])) OR (Fat Cell[Title/Abstract])) OR (Lipocytes[Title/Abstract])) OR (Lipocyte[Title/Abstract])))) OR (((((((Cell- and Tissue-Based Therapy[MeSH Terms]) OR (Cell and Tissue Based Therapy[Title/Abstract])) OR (Cell Therapy[Title/Abstract])) OR (Therapy, Cell[Title/Abstract])) OR (Tissue Therapy[Title/Abstract])) OR (Therapy, Tissue[Title/Abstract])))) AND ((((((Diabetic Foot[MeSH Terms]) OR (Foot, Diabetic[Title/Abstract])) OR (Diabetic Feet[Title/Abstract])) OR (Feet, Diabetic[Title/Abstract])) OR (Foot Ulcer, Diabetic[Title/Abstract]))) |
| --- | --- |
| Embase: 2005 | #1: 'stem cell'/exp OR 'stem cell' OR 'cells, stem':ti,ab,kw OR 'cell, stem':ti,ab,kw OR 'stem cell':ti,ab,kw OR 'mother cells':ti,ab,kw OR 'cell, mother':ti,ab,kw OR 'cells, mother':ti,ab,kw OR 'mother cell':ti,ab,kw OR 'progenitor cells':ti,ab,kw OR 'cell, progenitor':ti,ab,kw OR 'cells, progenitor':ti,ab,kw OR 'progenitor cell':ti,ab,kw OR 'colony-forming unit':ti,ab,kw OR 'colony forming unit':ti,ab,kw OR 'colony-forming units':ti,ab,kw OR 'colony forming units':ti,ab,kw  #2:'bone marrow'/exp OR 'bone marrow' OR 'marrow, bone':ti,ab,kw OR 'red marrow':ti,ab,kw OR 'marrow, red':ti,ab,kw OR 'yellow marrow':ti,ab,kw OR 'marrow, yellow':ti,ab,kw OR marrow:ti,ab,kw  #3: 'leukocytes, mononuclear'/exp OR 'leukocytes, mononuclear' OR 'mononuclear leukocyte':ti,ab,kw OR 'leukocyte, mononuclear':ti,ab,kw OR 'mononuclear leukocytes':ti,ab,kw OR 'peripheral blood mononuclear cells':ti,ab,kw OR 'peripheral blood mononuclear cell':ti,ab,kw OR 'peripheral blood human mononuclear cells':ti,ab,kw OR 'pbmc peripheral blood mononuclear cells':ti,ab,kw  #4: 'adipocyte'/exp OR 'adipocyte' OR 'fat cells':ti,ab,kw OR 'cell, fat':ti,ab,kw OR 'cells, fat':ti,ab,kw OR 'fat cell':ti,ab,kw OR lipocytes:ti,ab,kw OR lipocyte:ti,ab,kw OR 'adipose-derived stem cell':ti,ab,kw OR 'adipose stem cell':ti,ab,kw OR 'adipose tissue':ti,ab,kw OR 'stromal vascular fraction':ti,ab,kw  #5: 'cell therapy'/exp OR 'cell therapy' OR 'cell and tissue based therapy':ti,ab,kw OR 'cell therapy':ti,ab,kw OR 'therapy, cell':ti,ab,kw OR 'tissue therapy':ti,ab,kw OR 'therapy, tissue':ti,ab,kw  #6: 'diabetic foot'/exp OR 'diabetic foot' OR 'foot, diabetic':ti,ab,kw OR 'diabetic feet':ti,ab,kw OR 'feet, diabetic':ti,ab,kw OR 'foot ulcer, diabetic':ti,ab,kw  #7: #1 OR #2 OR #3 OR #4 OR #5  #6 AND #7 |
| Cochrane of library: 740 | #1: MeSH descriptor: [Stem Cells] explode all trees  #2: (Cells, Stem):ti,ab,kw OR (Cell, Stem):ti,ab,kw OR (Cells, Mother):ti,ab,kw OR (Mother Cells):ti,ab,kw OR (Progenitor Cell):ti,ab,kw OR (Mother Cell):ti,ab,kw OR (Cells, Progenitor):ti,ab,kw OR (Cell, Mother):ti,ab,kw OR (Progenitor Cells):ti,ab,kw OR (Cell, Progenitor):ti,ab,kw OR (Stem Cell):ti,ab,kw OR (Colony-Forming Units):ti,ab,kw OR (Colony Forming Unit):ti,ab,kw OR (Colony Forming Units):ti,ab,kw OR (Colony-Forming Unit):ti,ab,kw  #3: MeSH descriptor: [Bone Marrow] explode all trees  #4: (Marrow, Bone):ti,ab,kw OR (Marrow, Red):ti,ab,kw OR (Red Marrow):ti,ab,kw OR (Marrow):ti,ab,kw OR (Marrow, Yellow):ti,ab,kw OR (Yellow Marrow):ti,ab,kw  #5: MeSH descriptor: [Leukocytes, Mononuclear] explode all trees  #6: (Leukocyte, Mononuclear):ti,ab,kw OR (Mononuclear Leukocyte):ti,ab,kw OR (Mononuclear Leukocytes):ti,ab,kw OR (Peripheral Blood Mononuclear Cell):ti,ab,kw OR (Peripheral Blood Mononuclear Cells):ti,ab,kw OR (Peripheral Blood Human Mononuclear Cells):ti,ab,kw OR (PBMC Peripheral Blood Mononuclear Cells):ti,ab,kw  #7: MeSH descriptor: [Adipocytes] explode all trees  #8: (Adipocyte):ti,ab,kw OR (Lipocyte):ti,ab,kw OR (Fat Cell):ti,ab,kw OR (Fat Cells):ti,ab,kw OR (Cell, Fat):ti,ab,kw OR (Lipocytes):ti,ab,kw OR (Cells, Fat):ti,ab,kw  #9: MeSH descriptor: [Cell- and Tissue-Based Therapy] explode all trees  #10: (Cell and Tissue Based Therapy):ti,ab,kw OR (Cell Therapy):ti,ab,kw OR (Therapy, Cell):ti,ab,kw OR (Therapy, Tissue):ti,ab,kw OR (Tissue Therapy):ti,ab,kw  #11: MeSH descriptor: [Diabetic Foot] explode all trees  #12: (Feet, Diabetic):ti,ab,kw OR (Foot, Diabetic):ti,ab,kw OR (Diabetic Feet):ti,ab,kw OR (Foot Ulcer, Diabetic):ti,ab,kw  #13: #11 OR #12  #14: #1 OR #2 OR #3 OR #4 OR #5 OR #6 OR #7 OR #8 OR #9 OR #10  #15: #13 AND #14 |
| Web of science: 2854 | (Stem Cells OR Stem Cell OR Progenitor Cells OR Progenitor Cell OR Colony-Forming Unit OR Colony Forming Unit OR Colony-Forming Units OR Colony Forming Units OR Bone Marrow OR Red Marrow OR Yellow Marrow OR Marrow OR Mononuclear Leukocytes OR Peripheral Blood Mononuclear Cells OR Peripheral Blood Mononuclear Cell OR PBMC OR Adipocytes OR Adipocyte OR Fat Cells OR Fat Cell OR Lipocytes OR Lipocyte OR Adipose-derived stem cells OR Adipose stem cells OR Cell- and Tissue-Based Therapy OR Cell and Tissue Based Therapy OR Cell Therapy OR Tissue Therapy OR Cellular therapy OR Cell-based therapy OR Cell transplantation) AND (Diabetic Foot OR Diabetic Feet OR Diabetic foot ulcer OR Diabetic foot ulcers) |
| CNKI: 1094 | Stem Cells OR Bone Marrow OR Leukocytes, Mononuclear OR Adipocytes OR Cell- and Tissue-Based Therapy AND Diabetic Foot |

Table S1 Literature Search Strategy

## Table S2 Supplementary Basic Information of Included Literature

| Ref. | Study (Author, Year) | Design | Baseline ulcer area (cm²) T | Baseline ulcer area (cm²) C | Gender (M/F) T | Gender (M/F) C | Diabetes Duration (y) T | Diabetes Duration (y) C |
| --- | --- | --- | --- | --- | --- | --- | --- | --- |
| [26] | Ozturk et al. (2012) | RCT | - | - | 16/4 | 13/7 | 13.5 ± 8.5 | 12.8 ± 9.8 |
| [48] | Rakowska et al. (2023) | Non-RCT | 2.72 ± 2.85 | 2.68 ± 1.58 | 20/3 | 17/6 | 16.1 ± 8.6 | 19.7 ± 10.7 |
| [19] | Dash et al. (2009) | RCT | 7.26 ± 1.41 | - | - | - | - | - |
| [23] | Dubsky et al. (2013) | Non-RCT | 5.2 ± 1.6 | 5.9 ± 2 | 15/2 | 17/5 | 23.1 ± 15.2 | 19.8 ± 9 |
| [23] | Dubsky et al. (2013) | Non-RCT | 5.5 ± 1.7 | 5.9 ± 2 | 9/2 | 17/5 | 21.5 ± 9.4 | 19.8 ± 9 |
| [35] | Raposio et al. (2016) | RCT | 25.18 ± 5.6 | 11.24 ± 2.6 | 11/5 | 10/14 | - | - |
| [36] | Tanios et al. (2021) | RCT | 6.28 (Mean) | 5.63 (Mean) | 29/21 | 24/26 | - | - |
| [32] | Uzun et al. (2021) | RCT | 23.5 ± 5.6 | 25.8 ± 5.4 | 6/4 | 6/4 | 13.4 ± 3.5 | 14.5 ± 3.4 |
| [49] | Tan et al. (2017) | RCT | - | - | 72/28 | - | 11.2 ± 9.4 | - |
| [43] | Wang et al. (2024) | RCT | 9.67 ± 4.86 | 10.17 ± 5.19 | - | - | - | - |
| [29] | Qin et al. (2016) | RCT | - | - | 17/11 | 15/10 | 12.8 ± 7.2 | 13.1 ± 4.6 |
| [20] | Han et al. (2010) | RCT | 4.3 ± 2.1 | 4.0 ± 2.1 | 15/11 | 14/12 | - | - |
| [28] | He et al. (2014) | RCT | 3-72 | 6-42 | 30/20 | 24/26 | - | - |
| [25] | Kirana et al. (2012) | RCT | 9.6 ± 4.2 | - | 9/3 | - | 20.9 ± 4.3 | - |
| [25] | Kirana et al. (2012) | RCT | 7.7 ± 2.7 | - | 8/2 | - | 20.5 ± 3.9 | - |
| [33] | Moon et al. (2019) | RCT | 2.0 ± 0.9 | 2.8 ± 2.0 | 14/8 | 13/4 | 17.1 ± 10.7 | 19.9 ± 9.8 |
| [27] | Mohammadzadeh et al. (2013) | RCT | 14.2 ± 4.1 | 15.8 ± 17.0 | - | - | 16.5 ± 8.7 | 14.2 ± 8.5 |
| [21] | Lu et al. (2011) | RCT | 4.3 ± 3.1 | 4.5 ± 2.3 | 11/8 | - | 9.8 ± 5.0 | - |
| [21] | Lu et al. (2011) | RCT | 4.2 ± 2.9 | 4.5 ± 2.3 | 11/7 | - | 10.3 ± 5.6 | - |
| [38] | Debin et al. (2008) | RCT | 4.16 ± 0.95 | 3.81 ± 1.05 | 11/11 | 15/8 | 8.95 ± 4.94 | 10.30 ± 4.08 |
| [24] | Dubský et al. (2014) | Retrospective | 4.3 ± 1.6 | 4.1 ± 2 | 25/6 | 18/5 | 22.2 ± 14.7 | 20.3 ± 8.9 |
| [47] | Smith et al. (2020) | RCT | 3.10 ± 1.30 | 6.40 ± 5.90 | 6/0 | 4/2 | - | - |
| [44] | Liu et al. (2023) | RCT | 5.98 ± 0.93 | 6.13 ± 1.04 | 34/16 | 32/18 | 14.58 ± 4.19 | 13.57 ± 4.16 |
| [45] | Liu et al. (2021) | RCT | 15.03 ± 2.41 | 14.98 ± 2.53 | 31/23 | 29/22 | 9.35 ± 1.58 | 9.42 ± 1.60 |
| [39] | Sui et al. (2020) | RCT | - | - | 25/18 | 26/17 | 8.42 ± 2.61 | 8.34 ± 2.56 |
| [18] | Huang et al. (2005) | RCT | 2.71 ± 1.32 | 2.39 ± 1.15 | 9/5 | - | 12.9 ± 8.9 | - |
| [22] | Jain et al. (2011) | RCT | - | - | 17/8 | 15/9 | - | - |
| [37] | Pollak et al. (2025) | RCT | 1.72 ± 1.90 | 1.91 ± 2.18 | 34/10 | 37/8 | - | - |
| [37] | Pollak et al. (2025) | RCT | 2.07 ± 2.21 | 1.91 ± 2.18 | 38/9 | 37/8 | - | - |
| [37] | Pollak et al. (2025) | RCT | 2.17 ± 2.01 | 1.91 ± 2.18 | 18/5 | 37/8 | - | - |
| [31] | Lonardi et al. (2019) | RCT | - | - | 45/12 | 41/16 | - | - |
| [34] | Meamar et al. (2021) | RCT | 11.1 ± 5.8 | 8.6 ± 5.5 | 18/10 | - | - | - |
| [34] | Meamar et al. (2021) | RCT | 11.2 ± 5.6 | 8.6 ± 5.5 | 18/10 | - | - | - |
| [42] | Wang et al. (2024) | RCT | 7.5-48.64 | 7.5-48.64 | 28/15 | 25/18 | 10.89 ± 1.21 | 10.53 ± 1.15 |
| [41] | Li et al. (2014) | RCT | - | - | 17/13 | 15/11 | 7.8 ± 5.2 | 8.5 ± 4.3 |
| [30] | Zhang et al. (2016) | Non-RCT | - | - | 12/15 | 15/14 | - | - |
| [40] | Zhao et al. (2018) | RCT | - | - | 31/49 | - | - | - |
| [46] | Li et al. (2021) | RCT | - | - | 14/14 | 15/13 | - | - |

**Notes:** Data are presented as Mean ± Standard Deviation (SD), Mean (Range), or Number, unless otherwise indicated. Abbreviations: Ref., Reference number; T, Treatment group; C, Control group; M, Male; F, Female; y, years; RCT, Randomized Controlled Trial; Non-RCT, Non-Randomized Controlled Trial; Retrospective, Retrospective Comparative Study.

## Table S3 Supplementary Basic Information of Included Literature

| Ref. | Study (Author, Year) | Duration of Ulcer (days) | Cell Number / Dose | Control Group Treatment |
| --- | --- | --- | --- | --- |
| [26] | Ozturk et al. (2012) | - | 2×10⁶ cells per leg | Conventional treatment (vasodilators, anticoagulant/antiplatelet agents, antibiotics, blood glucose control, statins, wound care; hyperbaric oxygen therapy for ulcer patients) |
| [48] | Rakowska et al. (2023) | 593.58 ± 946.68 | 2.5×10⁶ cells / 1mL fibrin gel; 1mL for wounds ≤12cm², proportional increase for wounds >12cm² | Topical application of fibrin gel alone |
| [19] | Dash et al. (2009) | - | 10⁶ cells/cm² | Standard wound care (dressing changes, debridement) |
| [23] | Dubsky et al. (2013) | - | 2.2×10⁹ cells per leg | Conservative treatment (therapeutic shoes, orthoses, antibiotics, etc.) |
| [23] | Dubsky et al. (2013) | - | 2.4×10¹⁰ cells per leg | Conservative treatment (therapeutic shoes, orthoses, antibiotics, etc.) |
| [35] | Raposio et al. (2016) | 809.89 ± 194.51 | 5×10⁵ ASCs isolated from 80mL adipose tissue | Standard wound care (disinfection, debridement if necessary, advanced dressings + bandages, once weekly) |
| [36] | Tanios et al. (2021) | 329.84 ± 262.64 | - | Ulcer debridement + conventional dressing changes (povidone-iodine ointment), once every 3 weeks for 3 cycles |
| [32] | Uzun et al. (2021) | 55.2 ± 20.8 | Mean 6×10⁶ cells per patient | Standard diabetic wound care (blood glucose control, debridement of necrotic tissue, offloading, dressing changes, foot care education) |
| [49] | Tan et al. (2017) | - | Collected at peak CD34⁺ cell count; ~60mL cell suspension; 1.0-2.0mL per injection site, 3cm×3cm spacing | Percutaneous transluminal angioplasty alone (balloon dilation + stenting if necessary) |
| [43] | Wang et al. (2024) | - | CD34⁺ cell concentration: 12×10⁶ cells/mL | Conventional treatment (debridement, dressing application + basic therapy) |
| [29] | Qin et al. (2016) | - | 4.8–8.6×10⁷ cells per leg | Percutaneous transluminal angioplasty alone + standard care (antiplatelet therapy, glucose/lipid control, wound debridement and dressing changes) |
| [20] | Han et al. (2010) | - | 4.0×10⁶ ~ 8.0×10⁶ cells per ulcer | Application of fibrinogen + thrombin alone; other interventions (debridement, offloading, blood glucose control, dressing change frequency) consistent with the treatment group |
| [28] | He et al. (2014) | - | 5.8–8.2×10⁷ cells per leg | Conservative treatment |
| [25] | Kirana et al. (2012) | 166.6 ± 58.1 | 3×10⁸ cells per leg | Standard wound care (debridement, offloading, antibiotic therapy) |
| [25] | Kirana et al. (2012) | 86.1 ± 14.0 | 8×10⁷ cells per leg | Standard wound care (debridement, offloading, antibiotic therapy, etc.) |
| [33] | Moon et al. (2019) | 203.95 ± 334.84 | ~1×10⁶ cells per sheet | Polyurethane film (primary dressing) + polyurethane foam dressing + standard care (debridement, offloading, blood glucose control) |
| [27] | Mohammadzadeh et al. (2013) | 152.20 ± 118.72 | 9–12×10⁸ cells per leg | Sterile phosphate-buffered saline (PBS) injection + standard care (dressing changes, debridement, antibiotic therapy, offloading) |
| [21] | Lu et al. (2011) | 40 ± 23 | 9.6 ± 1.1 × 10⁸ cells per leg | Normal saline |
| [21] | Lu et al. (2011) | 44 ± 22 | 9.3 ± 1.1 × 10⁸ cells per leg | Normal saline |
| [38] | Debin et al. (2008) | - | 7.32×10⁸ - 5.61×10⁹ cells per patient | Conventional treatment (glucose/blood pressure/lipid control, debridement, offloading, antibiotics) |
| [24] | Dubský et al. (2014) | - | CD34⁺ cells: minimum concentration 2×10⁴/mL; total 40-70mL | Conservative treatment (standardized protocol including therapeutic shoes, orthoses, total contact casting for offloading, antibiotic therapy for infected ulcers, etc.) |
| [47] | Smith et al. (2020) | - | Mean harvested volume: 17.5mL; infiltration volume: 2mL/cm² wound area | Standard podiatric care (debridement, dressing changes, offloading) |
| [44] | Liu et al. (2023) | - | 200-250mL bone marrow separated into 40-50mL cell suspension | Conventional treatment (glucose control, vasodilation, anti-infection, lipid/blood pressure control, debridement and dressing changes + intravenous ozagrel sodium) |
| [45] | Liu et al. (2021) | 806.65 ± 135.05 | Mononuclear cell count: (4-9)×10⁸/L; 200mL bone marrow separated into 50mL suspension | Comprehensive conventional medical treatment (glucose control, neurotrophy, microcirculation improvement, debridement and dressing changes) |
| [39] | Sui et al. (2020) | - | 0.5-1×10⁶ cells/kg | Conventional treatment (glucose/blood pressure/lipid control, debridement, anti-infection) |
| [18] | Huang et al. (2005) | - | 3×10⁹ cells per leg | Intravenous prostaglandin E1 (90-200μg/day) |
| [22] | Jain et al. (2011) | - | 5mL per leg | Wound debridement |
| [37] | Pollak et al. (2025) | - | 10×10⁶ cells/dose, 2 doses (Days 1 and 8) | Intramuscular placebo (normal saline) + standard DFU care |
| [37] | Pollak et al. (2025) | - | 3×10⁶ cells/dose, 2 doses (Days 1 and 8) | Intramuscular placebo (normal saline) + standard DFU care |
| [37] | Pollak et al. (2025) | - | 30×10⁶ cells/dose, 2 doses (Days 1 and 8) | Intramuscular placebo (normal saline) + standard DFU care |
| [31] | Lonardi et al. (2019) | - | 10-30mL per leg | Standard care (wound cleansing, povidone-iodine petrolatum gauze dressing, rest and offloading of the affected limb) |
| [34] | Meamar et al. (2021) | 98 ± 12.1 | Seeding density: 1×10⁶ cells/cm² | Standard wound care |
| [34] | Meamar et al. (2021) | 98 ± 12.1 | Seeding density: 1×10⁶ cells/cm² | Standard wound care |
| [42] | Wang et al. (2024) | 132.41 ± 18.57 | Day 0: 1.0×10⁷ cells/4mL; Days 3, 6: 1.0×10⁷ cells/6mL; Days 15, 18: 2.0×10⁷ cells/8mL | Vacuum sealing drainage (VSD) + conventional treatment (glucose control, microcirculation improvement, debridement and dressing changes) |
| [41] | Li et al. (2014) | - | (1.8-2.3)×10⁷ cells per patient | Conventional treatment (glucose/blood pressure/lipid control + alprostadil for microcirculation improvement, 2-week treatment course) |
| [30] | Zhang et al. (2016) | - | ≥1×10⁷ cells per leg | Infra-aortic revascularization + normal saline infusion without cells (containing human albumin + heparin sodium) + standard care |
| [40] | Zhao et al. (2018) | ≤365 | 1×10⁶ cells per injection site | Traditional dressing changes after debridement, once every 3 days; skin grafting/flap transfer if necessary |
| [46] | Li et al. (2021) | - | 200-250mL bone marrow separated into 40-50mL cell suspension | Conventional treatment (glucose control, anti-infection, neurotrophy, vasodilation, debridement and dressing changes) |

**Notes:** Data for Duration of Ulcer are presented as Mean ± Standard Deviation (SD), Mean, or Range (e.g., ≤365), as reported in the original studies. Abbreviations: Ref., Reference number; ASCs, Adipose-derived stem cells; PBS, Phosphate-buffered saline; VSD, Vacuum sealing drainage.

## Table S4 Meta Regression

| Outcome | Source of Heterogeneity | Level (Contrast) | Coefficient | SE | 95% CI | P-value |
| --- | --- | --- | --- | --- | --- | --- |
| ABI (MD) | Route | IA (vs. Combined) | -0.14 | 0.08 | -0.31 to 0.04 | 0.112 |
|  |  | IM (vs. Combined) | -0.04 | 0.07 | -0.19 to 0.12 | 0.6146 |
|  |  | SC (vs. Combined) | -0.18 | 0.07 | -0.33 to -0.02 | 0.0305 |
|  | Cell Type | BMMNC (vs. BMMSC) | -0.05 | 0.1 | -0.27 to 0.18 | 0.6275 |
|  |  | PBMNC (vs. BMMSC) | -0.12 | 0.07 | -0.28 to 0.04 | 0.1172 |
|  |  | UC-MSC (vs. BMMSC) | -0.03 | 0.09 | -0.25 to 0.18 | 0.7259 |
|  | Ulcer Area | ≥10 cm² (vs. <10 cm²) | -0.08 | 0.13 | -0.37 to 0.21 | 0.5449 |
|  |  | <10 cm² (vs. ≥10 cm²) | 0.08 | 0.06 | -0.05 to 0.21 | 0.1887 |
|  | Diabetes Duration | 10–15 years (vs. Ref) | 0 | 0.07 | -0.16 to 0.17 | 0.9462 |
|  |  | >15 years (vs. Ref) | 0.07 | 0.12 | -0.20 to 0.35 | 0.5455 |
|  |  | <10 years (vs. Ref) | 0.13 | 0.1 | -0.10 to 0.37 | 0.2268 |
|  | Cell Dose | High (vs. High+Ultra) | 0.02 | 0.1 | -0.21 to 0.25 | 0.8387 |
|  |  | Low (vs. High+Ultra) | 0.22 | 0.16 | -0.18 to 0.61 | 0.2312 |
|  |  | Medium (vs. High+Ultra) | -0.07 | 0.09 | -0.30 to 0.16 | 0.4878 |
|  |  | Ultra-High (vs. High+Ultra) | -0.01 | 0.27 | -0.66 to 0.64 | 0.9711 |
|  |  | Unclear (vs. High+Ultra) | -0.11 | 0.09 | -0.33 to 0.11 | 0.272 |
|  | Ulcer Duration | ≤200 days (vs. >200 days) | 0.05 | 0.06 | -0.09 to 0.19 | 0.4398 |
|  | Follow-up | <10 mo (vs. ≥10 mo) | 0.04 | 0.07 | -0.13 to 0.20 | 0.6246 |
| Amputation (LogOR) | Region | Europe (vs. Asia) | -0.05 | 0.42 | -0.95 to 0.85 | 0.9033 |
|  |  | North America (vs. Asia) | 2.65 | 0.78 | 0.99 to 4.31 | 0.0038 |
|  | Cell Type | BMMSC (vs. ASC) | -1.58 | 0.75 | -3.19 to 0.04 | 0.0555 |
|  |  | BMMNC (vs. ASC) | -0.98 | 0.59 | -2.24 to 0.29 | 0.1194 |
|  |  | PBMNC (vs. ASC) | -1.15 | 0.61 | -2.45 to 0.15 | 0.0791 |
|  |  | UC-MSC (vs. ASC) | 1.67 | 0.73 | 0.11 to 3.23 | 0.0374 |
|  | Follow-up | <10 mo (vs. ≥10 mo) | -0.71 | 0.5 | -1.77 to 0.34 | 0.1728 |
|  | Diabetes Duration | 10–15 years (vs. Ref) | 0.33 | 0.65 | -1.06 to 1.71 | 0.6246 |
|  |  | >15 years (vs. Ref) | -0.57 | 0.62 | -1.88 to 0.75 | 0.3713 |
|  |  | <10 years (vs. Ref) | -1.45 | 1.07 | -3.74 to 0.84 | 0.1961 |
|  | Cell Dose | High (vs. High+Ultra) | 0.53 | 1.4 | -2.51 to 3.56 | 0.7126 |
|  |  | Low (vs. High+Ultra) | 1.83 | 1.37 | -1.13 to 4.79 | 0.2045 |
|  |  | Medium (vs. High+Ultra) | 1.77 | 1.41 | -1.27 to 4.81 | 0.2305 |
|  |  | Ultra-High (vs. High+Ultra) | 0.09 | 1.37 | -2.86 to 3.04 | 0.9494 |
|  |  | Unclear (vs. High+Ultra) | 0.81 | 1.37 | -2.15 to 3.78 | 0.5639 |
|  | Route | IA (vs. Combined) | 0.21 | 1.82 | -3.68 to 4.11 | 0.9075 |
|  |  | IM (vs. Combined) | 1.28 | 1.08 | -1.04 to 3.61 | 0.2561 |
|  |  | SC (vs. Combined) | 1.84 | 1.42 | -1.21 to 4.89 | 0.2173 |
|  |  | Topical (vs. Combined) | 2.32 | 2.21 | -2.43 to 7.07 | 0.3124 |
|  | Ulcer Area | ≥10 cm² (vs. Ref) | -0.75 | 1.07 | -3.03 to 1.52 | 0.4927 |
|  |  | <10 cm² (vs. Ref) | -0.59 | 0.69 | -2.04 to 0.87 | 0.4041 |
|  | Ulcer Duration | >200 days (vs. Ref) | 1.05 | 1.98 | -3.16 to 5.25 | 0.6049 |
|  |  | ≤200 days (vs. Ref) | -0.28 | 0.63 | -1.61 to 1.05 | 0.6626 |
| Claudication (MD) | Ulcer Duration | >200 days (vs. Ref) | -1.23 | 0.12 | -1.73 to -0.73 | 0.0089 |
|  | Ulcer Area | ≥10 cm² (vs. Ref) | -1.25 | 0.02 | -1.55 to -0.94 | 0.0123 |
|  |  | <10 cm² (vs. Ref) | -1.11 | 0.16 | -3.16 to 0.95 | 0.0925 |
|  | Cell Type | PBMNC (vs. BMMSC) | 0.14 | 0.16 | -1.91 to 2.19 | 0.5446 |
|  |  | UC-MSC (vs. BMMSC) | 1.25 | 0.02 | 0.94 to 1.55 | 0.0123 |
|  | Diabetes Duration | 10–15 years (vs. Ref) | 0 | 0.27 | -3.47 to 3.47 | 0.9967 |
|  |  | <10 years (vs. Ref) | -1.23 | 0.23 | -4.19 to 1.73 | 0.1194 |
|  | Route | IM (vs. Combined) | -1.02 | 1.04 | -14.19 to 12.15 | 0.5053 |
|  |  | SC (vs. Combined) | -1.09 | 6.02 | -77.55 to 75.37 | 0.8859 |
|  | Cell Dose | Medium (vs. Low) | -1.02 | 1.04 | -14.19 to 12.15 | 0.5053 |
|  |  | Unclear (vs. Low) | -1.09 | 6.02 | -77.55 to 75.37 | 0.8859 |
| Neovascularization (LogOR) | Diabetes Duration | 10–15 years (vs. Ref) | 1.51 | 0.39 | -3.47 to 6.50 | 0.1616 |
|  |  | >15 years (vs. Ref) | -0.44 | 0.26 | -3.79 to 2.91 | 0.3434 |
|  |  | <10 years (vs. Ref) | 3.41 | 0.4 | -1.72 to 8.55 | 0.075 |
|  | Ulcer Area | <10 cm² (vs. Ref) | 1.98 | 1.02 | -0.84 to 4.80 | 0.123 |
|  | Ulcer Duration | ≤200 days (vs. Ref) | 2.27 | 1.24 | -1.19 to 5.72 | 0.1427 |
|  | Cell Type | BMMNC (vs. BMMSC) | 0 | 2.07 | -6.58 to 6.59 | 0.9984 |
|  |  | PBMNC (vs. BMMSC) | -1.55 | 1.44 | -6.14 to 3.04 | 0.3608 |
|  | Cell Dose | High (vs. High+Ultra) | 1.74 | 1.59 | -5.12 to 8.60 | 0.3885 |
|  |  | Low (vs. High+Ultra) | -1.32 | 1.39 | -7.30 to 4.67 | 0.4439 |
|  |  | Ultra-High (vs. High+Ultra) | 0.36 | 1.68 | -6.88 to 7.60 | 0.8499 |
|  | Route | IM (vs. Combined) | -0.35 | 1.37 | -4.14 to 3.44 | 0.8105 |
|  | Region | Europe (vs. Asia) | -0.35 | 2.7 | -7.84 to 7.14 | 0.9032 |
|  | Follow-up | <10 mo (vs. ≥10 mo) | 0.35 | 2.7 | -7.14 to 7.84 | 0.9032 |
| PFWD (MD) | Route | IM (vs. Combined) | -123.13 | 26.35 | -206.98 to -39.27 | 0.0185 |
|  |  | SC (vs. Combined) | 107.36 | 25.31 | 26.81 to 187.90 | 0.024 |
|  | Cell Dose | Low (vs. High+Ultra) | -134 | 58.1 | -872.26 to 604.26 | 0.2605 |
|  |  | Medium (vs. High+Ultra) | -114.73 | 39.9 | -621.69 to 392.22 | 0.2131 |
|  |  | Ultra-High (vs. High+Ultra) | 61.8 | 114.17 | -1388.82 to 1512.42 | 0.6841 |
|  |  | Unclear (vs. High+Ultra) | 113.9 | 38.23 | -371.81 to 599.61 | 0.2061 |
|  | Cell Type | PBMNC (vs. BMMSC) | 101.78 | 117.23 | -271.31 to 474.87 | 0.4492 |
|  |  | UC-MSC (vs. BMMSC) | -118.27 | 122.87 | -509.29 to 272.76 | 0.4068 |
|  | Diabetes Duration | 10–15 years (vs. Ref) | -4.15 | 843.82 | -3634.82 to 3626.52 | 0.9965 |
|  |  | >15 years (vs. Ref) | -54.79 | 911.45 | -3976.43 to 3866.85 | 0.9575 |
|  | Ulcer Area | <10 cm² (vs. Ref) | -37.19 | 245.98 | -720.14 to 645.77 | 0.8872 |
| Resting Pain (MD) | Route | IM (vs. Combined) | -1.07 | 0.37 | -1.95 to -0.20 | 0.0231 |
|  |  | SC (vs. Combined) | -0.64 | 0.89 | -2.74 to 1.46 | 0.4959 |
|  | Ulcer Area | ≥10 cm² (vs. Ref) | -0.72 | 0.39 | -1.64 to 0.21 | 0.1087 |
|  |  | <10 cm² (vs. Ref) | -1.26 | 0.46 | -2.35 to -0.18 | 0.0286 |
|  | Cell Type | BMMNC (vs. BMMSC) | -0.59 | 0.76 | -2.45 to 1.28 | 0.4714 |
|  |  | PBMNC (vs. BMMSC) | -0.06 | 0.81 | -2.04 to 1.92 | 0.9419 |
|  |  | UC-MSC (vs. BMMSC) | 0.91 | 0.4 | -0.07 to 1.90 | 0.0636 |
|  | Cell Dose | High (vs. High+Ultra) | -0.11 | 0.79 | -2.32 to 2.09 | 0.894 |
|  |  | Low (vs. High+Ultra) | 1.7 | 0.74 | -0.35 to 3.75 | 0.0824 |
|  |  | Medium (vs. High+Ultra) | 0.61 | 0.72 | -1.39 to 2.60 | 0.4472 |
|  |  | Ultra-High (vs. High+Ultra) | -0.46 | 1.59 | -4.87 to 3.95 | 0.7864 |
|  |  | Unclear (vs. High+Ultra) | 0.59 | 0.85 | -1.75 to 2.94 | 0.5211 |
|  | Ulcer Duration | >200 days (vs. Ref) | -0.46 | 0.43 | -1.48 to 0.55 | 0.3175 |
|  |  | ≤200 days (vs. Ref) | -1.24 | 0.67 | -2.82 to 0.35 | 0.1074 |
|  | Diabetes Duration | 10–15 years (vs. Ref) | 0.96 | 0.69 | -0.83 to 2.75 | 0.2258 |
|  |  | >15 years (vs. Ref) | -0.45 | 1.29 | -3.77 to 2.86 | 0.7407 |
|  |  | <10 years (vs. Ref) | 0.1 | 0.66 | -1.59 to 1.79 | 0.8843 |
| TcPO₂ (MD) | Cell Dose | Low (vs. High) | -15.55 | 3.49 | -24.08 to -7.02 | 0.0043 |
|  |  | Medium (vs. High) | -17.3 | 2.94 | -24.51 to -10.10 | 0.0011 |
|  |  | Ultra-High (vs. High) | 7.64 | 4.5 | -3.38 to 18.66 | 0.1408 |
|  |  | Unclear (vs. High) | -13.82 | 2.95 | -21.05 to -6.60 | 0.0034 |
|  | Ulcer Area | <10 cm² (vs. Ref) | 14.16 | 4.35 | 4.31 to 24.01 | 0.01 |
|  | Diabetes Duration | 10–15 years (vs. Ref) | 3.41 | 2.33 | -2.60 to 9.41 | 0.2045 |
|  |  | >15 years (vs. Ref) | 26.01 | 6.93 | 8.20 to 43.82 | 0.0132 |
|  |  | <10 years (vs. Ref) | 21.45 | 7.98 | 0.93 to 41.97 | 0.0435 |
|  | Region | Europe (vs. Asia) | 22.93 | 8.14 | 4.52 to 41.34 | 0.0201 |
|  | Cell Type | BMMSC (vs. ASC) | 19.78 | 10.02 | -4.73 to 44.29 | 0.0957 |
|  |  | BMMNC (vs. ASC) | 18.62 | 8.24 | -1.54 to 38.79 | 0.0646 |
|  |  | PBMNC (vs. ASC) | 2.65 | 4.83 | -9.16 to 14.46 | 0.6029 |
|  |  | UC-MSC (vs. ASC) | -0.57 | 4.89 | -12.52 to 11.39 | 0.9115 |
|  | Ulcer Duration | >200 days (vs. Ref) | -0.98 | 7.02 | -17.17 to 15.22 | 0.8926 |
|  |  | ≤200 days (vs. Ref) | 15.25 | 8.23 | -3.73 to 34.22 | 0.101 |
|  | Route | IM (vs. IA) | 2.2 | 5.79 | -11.49 to 15.90 | 0.7148 |
|  |  | SC (vs. IA) | 3.46 | 5.78 | -10.20 to 17.13 | 0.5678 |
|  |  | Topical (vs. IA) | -0.66 | 15.69 | -37.77 to 36.45 | 0.9676 |
|  | Follow-up | <10 mo (vs. ≥10 mo) | 2.8 | 4.96 | -8.42 to 14.01 | 0.5861 |
| Ulcer Area Reduction (MD) | Ulcer Area | <10 cm² (vs. ≥10 cm²) | 1.86 | 0.08 | 1.61 to 2.11 | <0.001 |
|  | Ulcer Duration | >200 days (vs. Ref) | -1.86 | 0.07 | -2.18 to -1.54 | 0.0016 |
|  |  | ≤200 days (vs. Ref) | -3.47 | 1.36 | -9.31 to 2.37 | 0.1251 |
|  | Cell Dose | Low (vs. High) | 5.96 | 0.45 | 0.19 to 11.74 | 0.0484 |
|  |  | Medium (vs. High) | 6.88 | 0.43 | 1.46 to 12.30 | 0.0395 |
|  |  | Unclear (vs. High) | 8.74 | 0.43 | 3.32 to 14.16 | 0.0311 |
|  | Cell Type | PBMNC (vs. BMMSC) | -7.37 | 70.51 | -310.75 to 296.01 | 0.9263 |
|  |  | UC-MSC (vs. BMMSC) | -1.4 | 25.61 | -111.58 to 108.77 | 0.9613 |
|  | Follow-up | <10 mo (vs. ≥10 mo) | 1.4 | 20.96 | -65.32 to 68.12 | 0.9509 |
|  | Route | Topical (vs. IM) | -1.4 | 20.96 | -68.12 to 65.32 | 0.9509 |
|  | Diabetes Duration | <10 years (vs. 10–15) | 1.25 | 29.71 | -93.30 to 95.80 | 0.9692 |
| Ulcer Healing Rate (LogOR) | Diabetes Duration | 10–15 years (vs. Ref) | 0.52 | 0.55 | -0.62 to 1.66 | 0.3566 |
|  |  | >15 years (vs. Ref) | 1.3 | 0.43 | 0.40 to 2.20 | 0.0063 |
|  |  | <10 years (vs. Ref) | 0.31 | 0.54 | -0.80 to 1.43 | 0.568 |
|  | Region | Asia (vs. Africa) | -0.78 | 0.77 | -2.38 to 0.81 | 0.3216 |
|  |  | Europe (vs. Africa) | -0.4 | 0.81 | -2.08 to 1.28 | 0.6251 |
|  |  | North America (vs. Africa) | -1.77 | 0.83 | -3.48 to -0.06 | 0.0435 |
|  | Cell Type | BM-DC (vs. ASC) | -0.73 | 0.88 | -2.55 to 1.10 | 0.4181 |
|  |  | BMMSC (vs. ASC) | -0.11 | 0.54 | -1.23 to 1.02 | 0.8475 |
|  |  | BMMNC (vs. ASC) | 1.15 | 0.73 | -0.37 to 2.66 | 0.1301 |
|  |  | PBMNC (vs. ASC) | 0.47 | 0.78 | -1.14 to 2.09 | 0.5487 |
|  |  | UC-MSC (vs. ASC) | -0.46 | 0.43 | -1.35 to 0.43 | 0.2976 |
|  | Follow-up | <10 mo (vs. ≥10 mo) | 0.43 | 0.37 | -0.33 to 1.20 | 0.2559 |
|  | Route | IM (vs. Combined) | -0.07 | 0.51 | -1.12 to 0.99 | 0.8974 |
|  |  | SC (vs. Combined) | -0.16 | 0.57 | -1.34 to 1.01 | 0.7747 |
|  |  | Topical (vs. Combined) | 0.73 | 0.87 | -1.06 to 2.52 | 0.4074 |
|  | Cell Dose | High (vs. High+Ultra) | 1.39 | 1.76 | -2.26 to 5.04 | 0.4366 |
|  |  | Low (vs. High+Ultra) | -0.81 | 1.15 | -3.18 to 1.57 | 0.4897 |
|  |  | Medium (vs. High+Ultra) | -0.88 | 1.13 | -3.23 to 1.47 | 0.4441 |
|  |  | Ultra-High (vs. High+Ultra) | -0.09 | 1.31 | -2.80 to 2.62 | 0.9477 |
|  |  | Unclear (vs. High+Ultra) | -0.44 | 1.13 | -2.77 to 1.90 | 0.702 |
|  | Ulcer Duration | >200 days (vs. Ref) | 0.16 | 0.54 | -0.95 to 1.26 | 0.7731 |
|  |  | ≤200 days (vs. Ref) | 0.35 | 0.59 | -0.88 to 1.57 | 0.5642 |
|  | Ulcer Area | ≥10 cm² (vs. Ref) | 0.29 | 0.52 | -0.79 to 1.37 | 0.5865 |
|  |  | <10 cm² (vs. Ref) | 0.01 | 0.42 | -0.85 to 0.86 | 0.9829 |
| Healing Time (MD) | Route | SC (vs. IM) | 23.06 | 3.89 | 12.27 to 33.85 | 0.004 |
|  |  | Topical (vs. IM) | 18.43 | 7.09 | -1.27 to 38.13 | 0.0602 |
|  | Ulcer Duration | >200 days (vs. Ref) | 13.47 | 8.28 | -9.52 to 36.46 | 0.1792 |
|  |  | ≤200 days (vs. Ref) | 23.56 | 4.41 | 11.33 to 35.80 | 0.0059 |
|  | Diabetes Duration | 10–15 years (vs. Ref) | -18.53 | 44.9 | -143.19 to 106.14 | 0.701 |
|  |  | >15 years (vs. Ref) | -21.84 | 5.66 | -37.55 to -6.12 | 0.0182 |
|  | Cell Dose | Medium (vs. Low) | -10.82 | 21.38 | -70.19 to 48.54 | 0.6393 |
|  |  | Unclear (vs. Low) | -21.09 | 57.47 | -180.64 to 138.46 | 0.7322 |
|  | Follow-up | <10 mo (vs. ≥10 mo) | -10.2 | 21.4 | -65.21 to 44.81 | 0.6538 |
|  | Cell Type | UC-MSC (vs. ASC) | -8.1 | 18.24 | -54.98 to 38.78 | 0.6754 |
|  | Region | Asia (vs. Africa) | 21.26 | 63.02 | -153.71 to 196.24 | 0.7528 |
|  |  | Europe (vs. Africa) | 42 | 127.05 | -310.75 to 394.75 | 0.7576 |
|  | Ulcer Area | ≥10 cm² (vs. Ref) | -21.48 | 110.13 | -327.25 to 284.28 | 0.8548 |
|  |  | <10 cm² (vs. Ref) | -13.35 | 111.84 | -323.87 to 297.17 | 0.9107 |

**Abbreviations:** ABI: Ankle-Brachial Index; MD: Mean Difference; LogOR: Log Odds Ratio; SE: Standard Error; CI: Confidence Interval; Ref: Reference group for comparison; IA: Intra-arterial Injection; IM: Intramuscular Injection; SC: Subcutaneous Injection; ASC: Adipose-Derived Stem Cells; BM-DC: Autologous Bone Marrow-Derived Dendritic Cells; BMMNC: Autologous Bone Marrow Mononuclear Cells; BMMSC: Autologous Bone Marrow Mesenchymal Stem Cells; PBMNC: Autologous Peripheral Blood Mononuclear Cells; UC-MSC: Umbilical Cord/Placental Mesenchymal Stem Cells; PFWD: Pain-Free Walking Distance; mo: months

## Fig S1


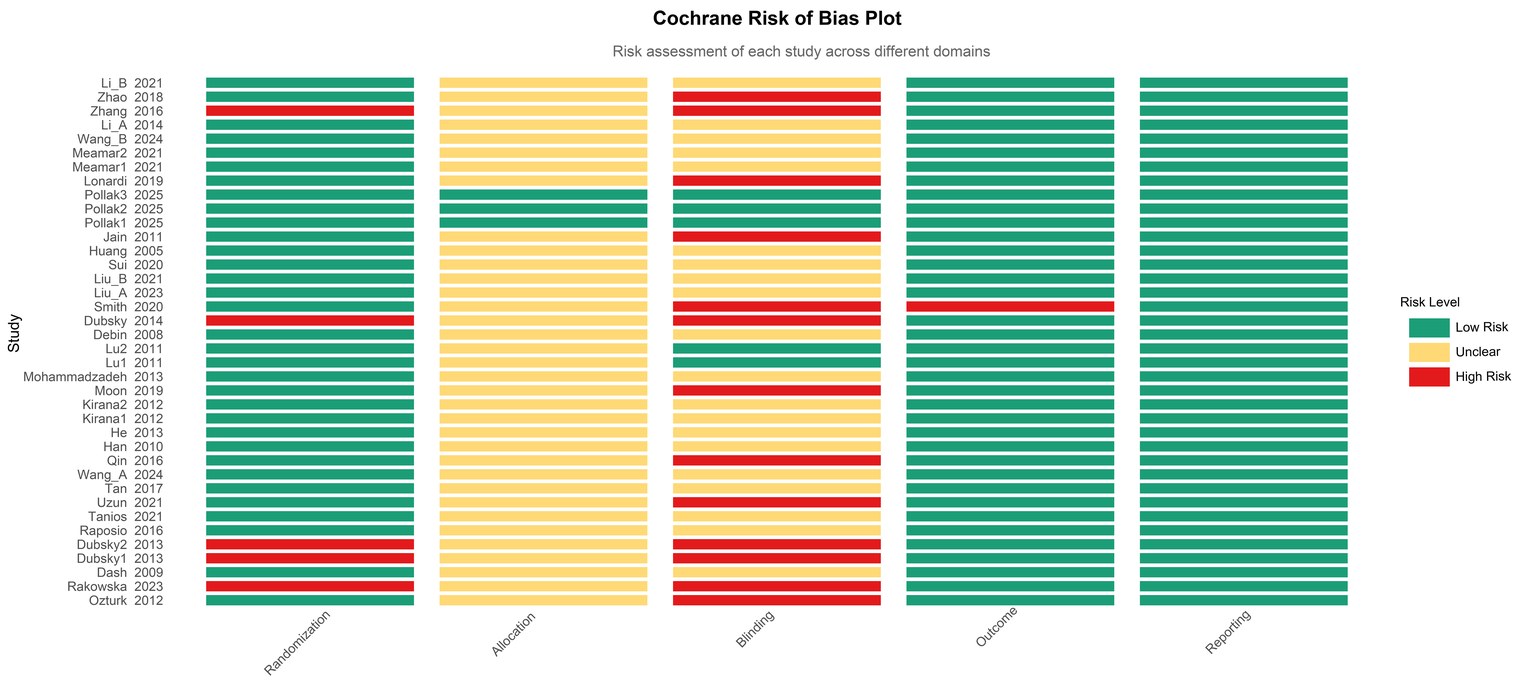


Fig S1 Quality assessment heatmap

## Fig S2


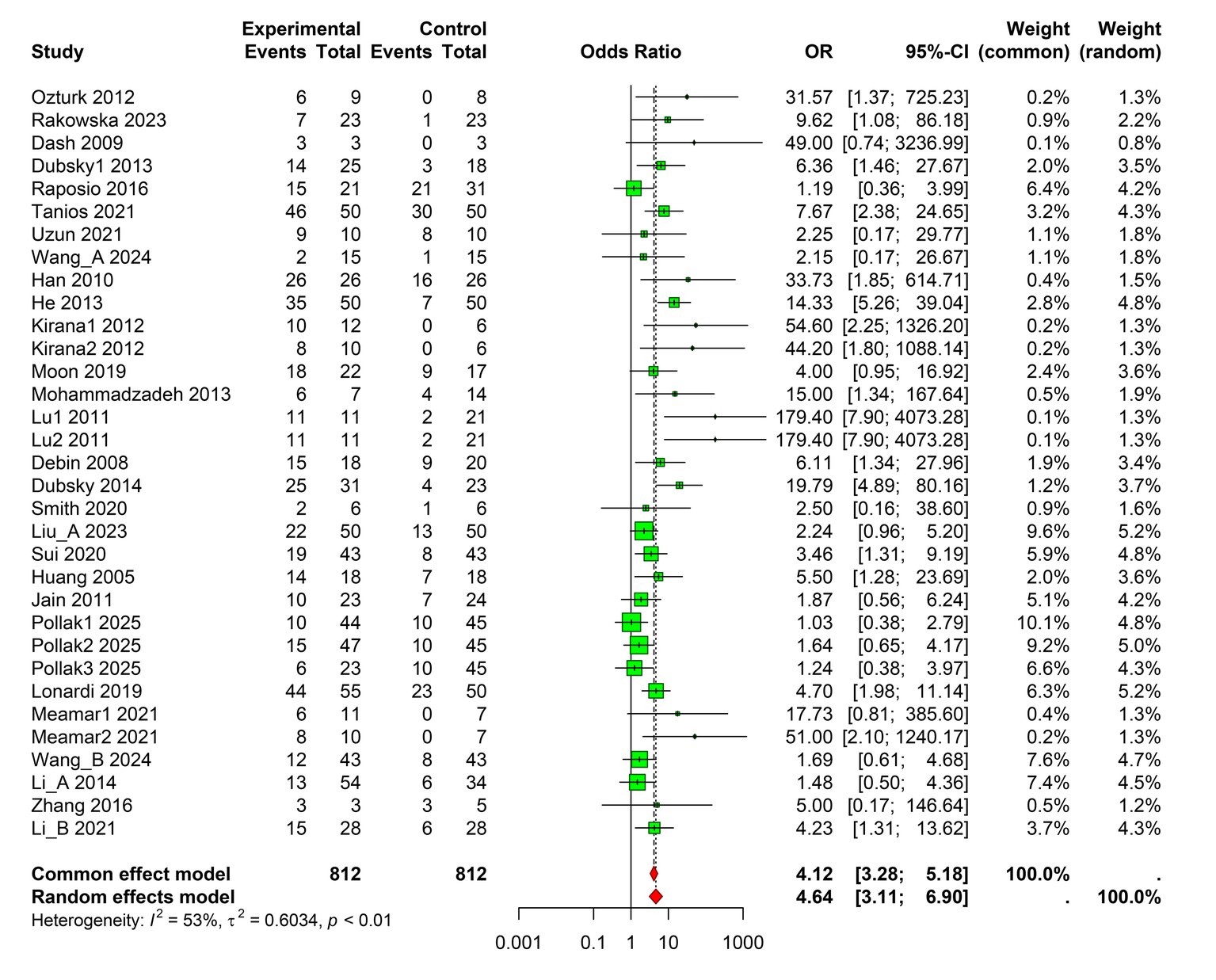


Fig S2 Overall forest plot of ulcer healing rate, OR: Odds Ratio, CI: Confidence Interval

## Fig S3


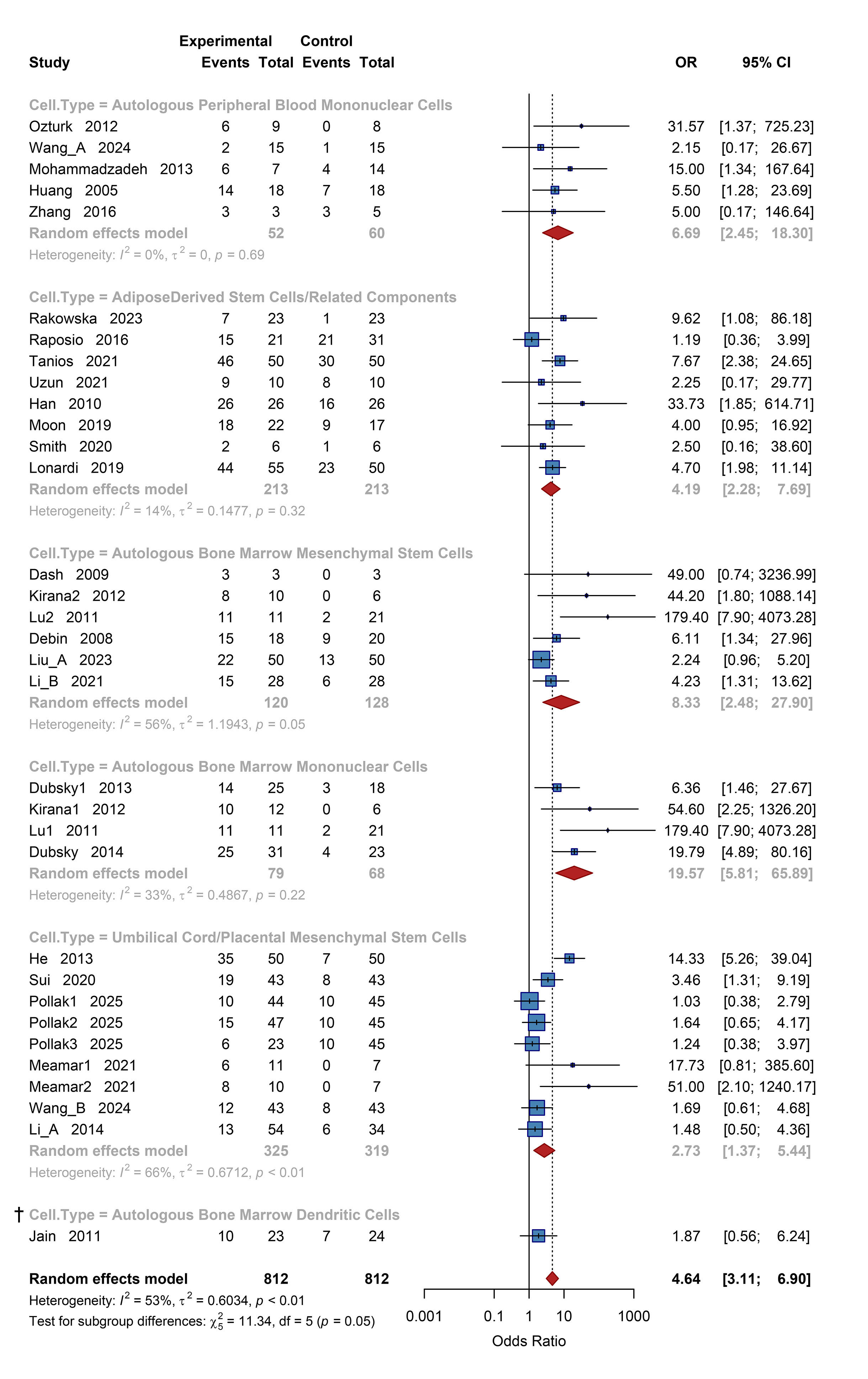


Fig S3 Subgroup analysis of ulcer healing rate by cell type, OR: Odds Ratio, CI: Confidence Interval, † Results based on sparse data (number of studies k < 3) should be interpreted with caution.

## Fig S4


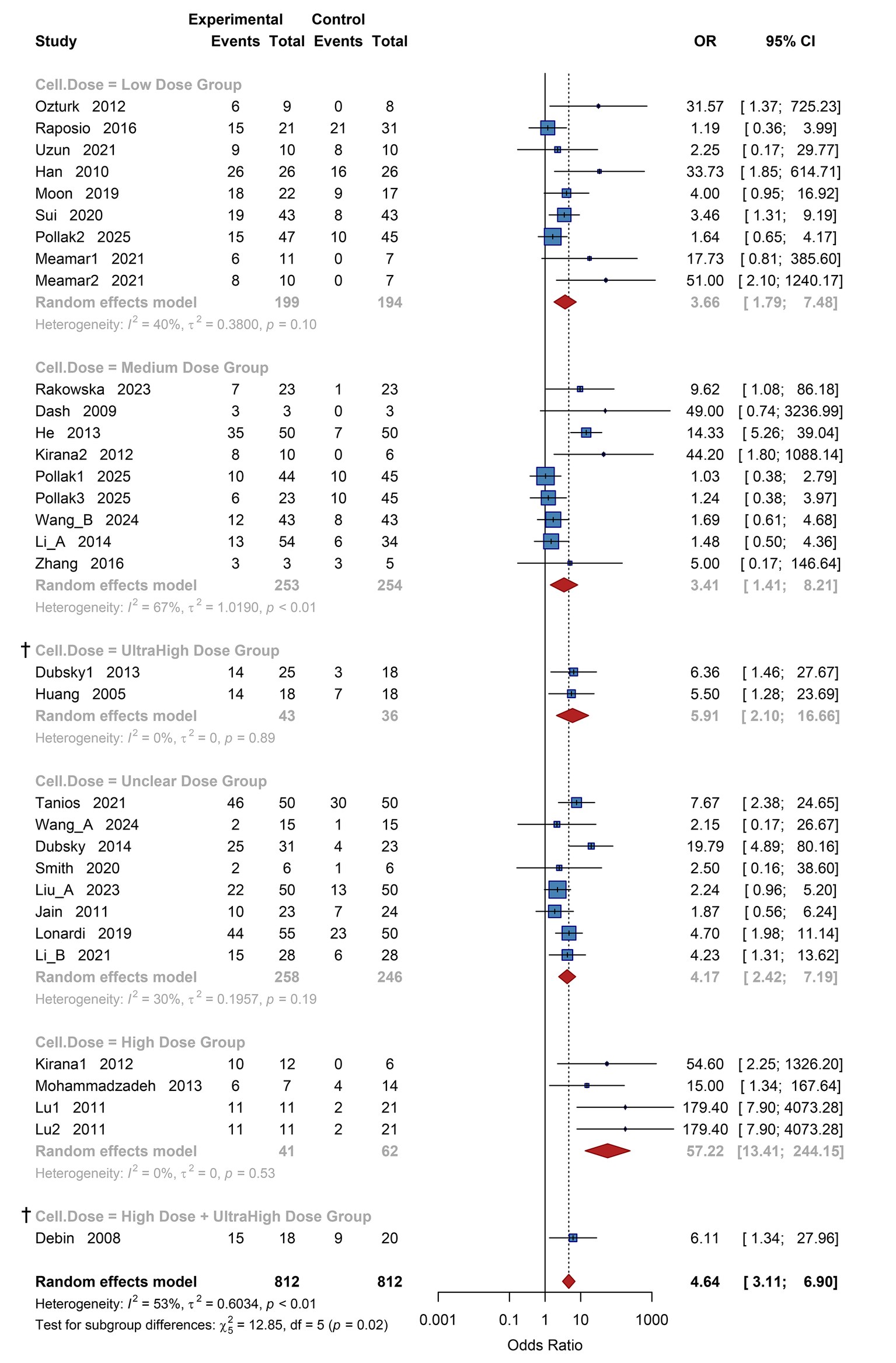


Fig S4 Ulcer healing rate based on subgroup analysis of cell dose, OR: Odds Ratio, CI: Confidence Interval, Low-dose group: 0.5×10⁶ to 8×10⁶ cells, Medium-dose group: 1×10⁷ to 8.6×10⁷ cells, High-dose group: 3×10⁸ to 1.2×10⁹ cells, Ultra-high-dose group: ≥2×10⁹ cells, unclear dose group: No quantifiable total cell count available, † Results based on sparse data (number of studies k < 3) should be interpreted with caution.

## Fig S5


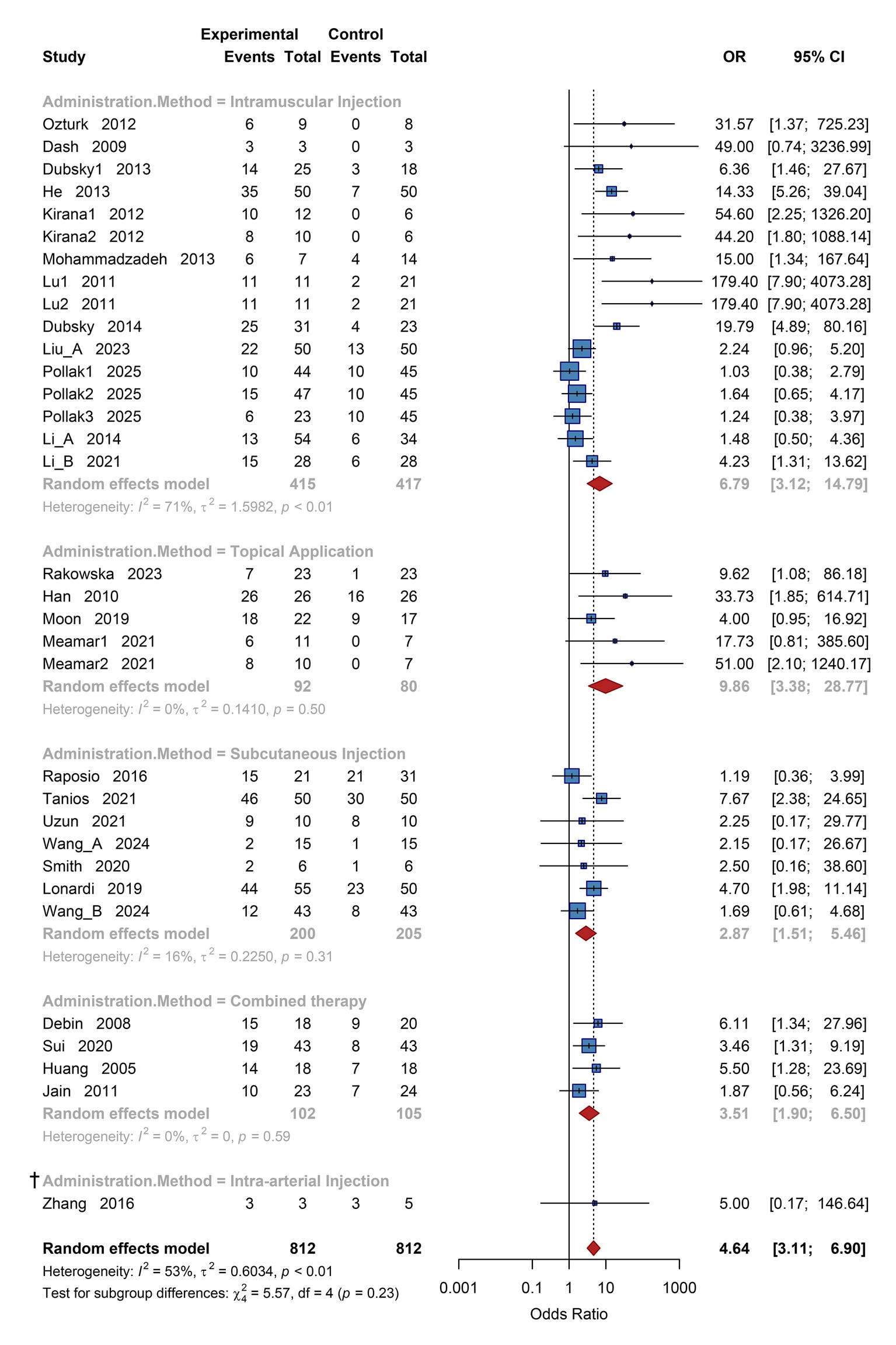


Fig S5 Ulcer healing rate subgroup analysis based on treatment methods, OR: Odds Ratio, CI: Confidence Interval, † Results based on sparse data (number of studies k < 3) should be interpreted with caution.

## Fig S6


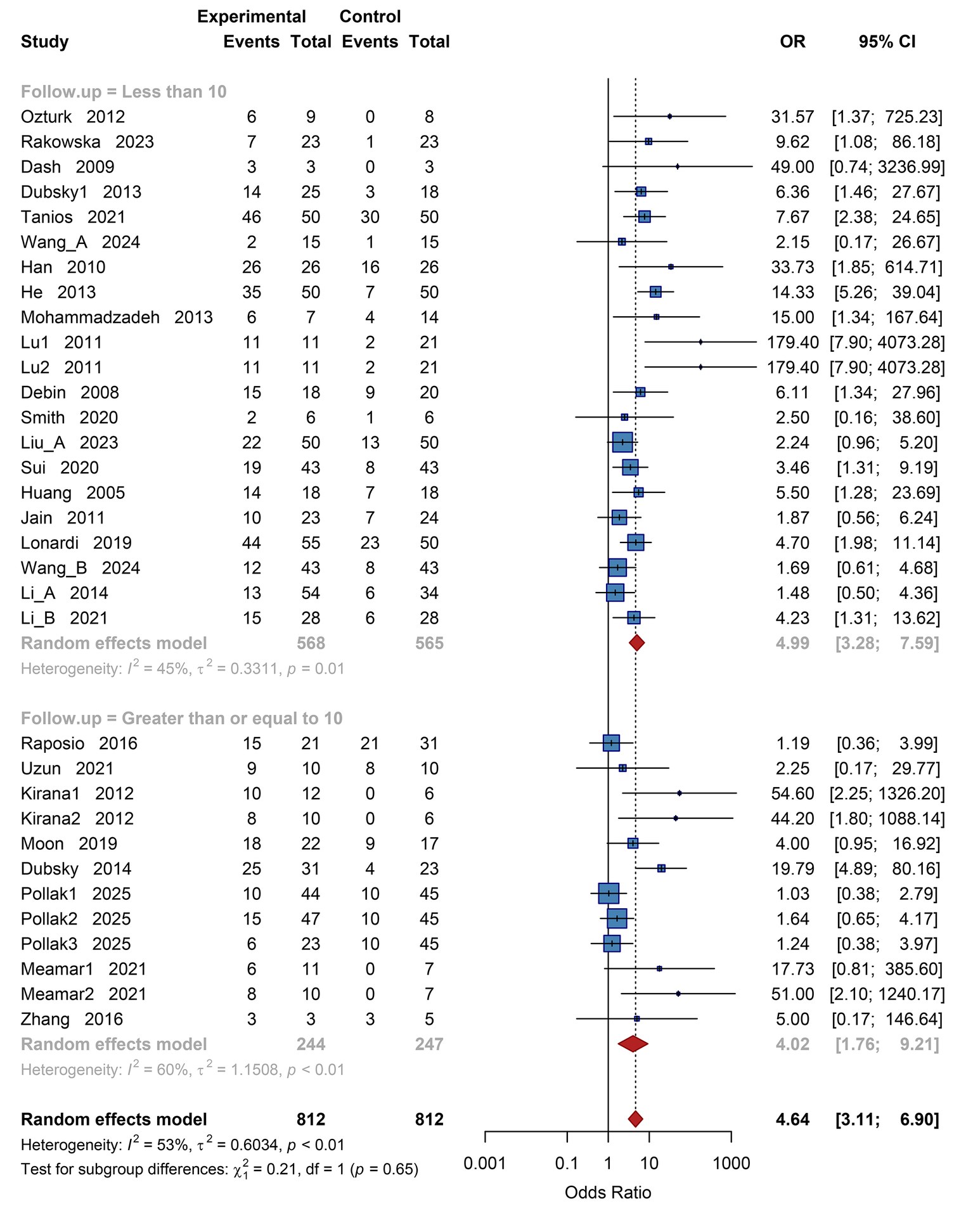


Fig S6 Subgroup analysis of ulcer healing rate over follow-up time, OR: Odds Ratio, CI: Confidence Interval

## Fig S7


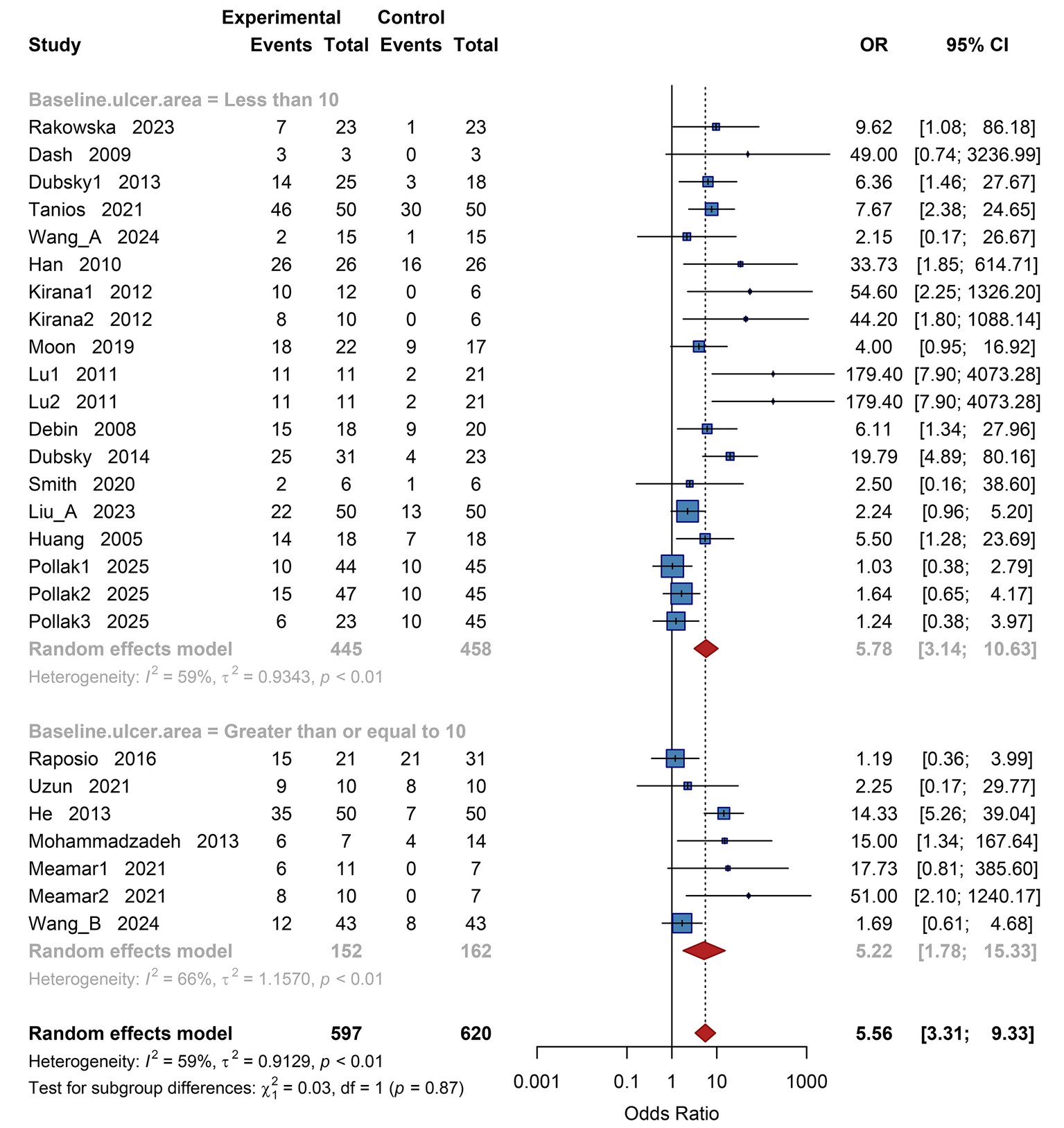


Fig S7 Ulcer healing rate based on subgroup analysis by baseline ulcer size, OR: Odds Ratio, CI: Confidence Interval

## Fig S8


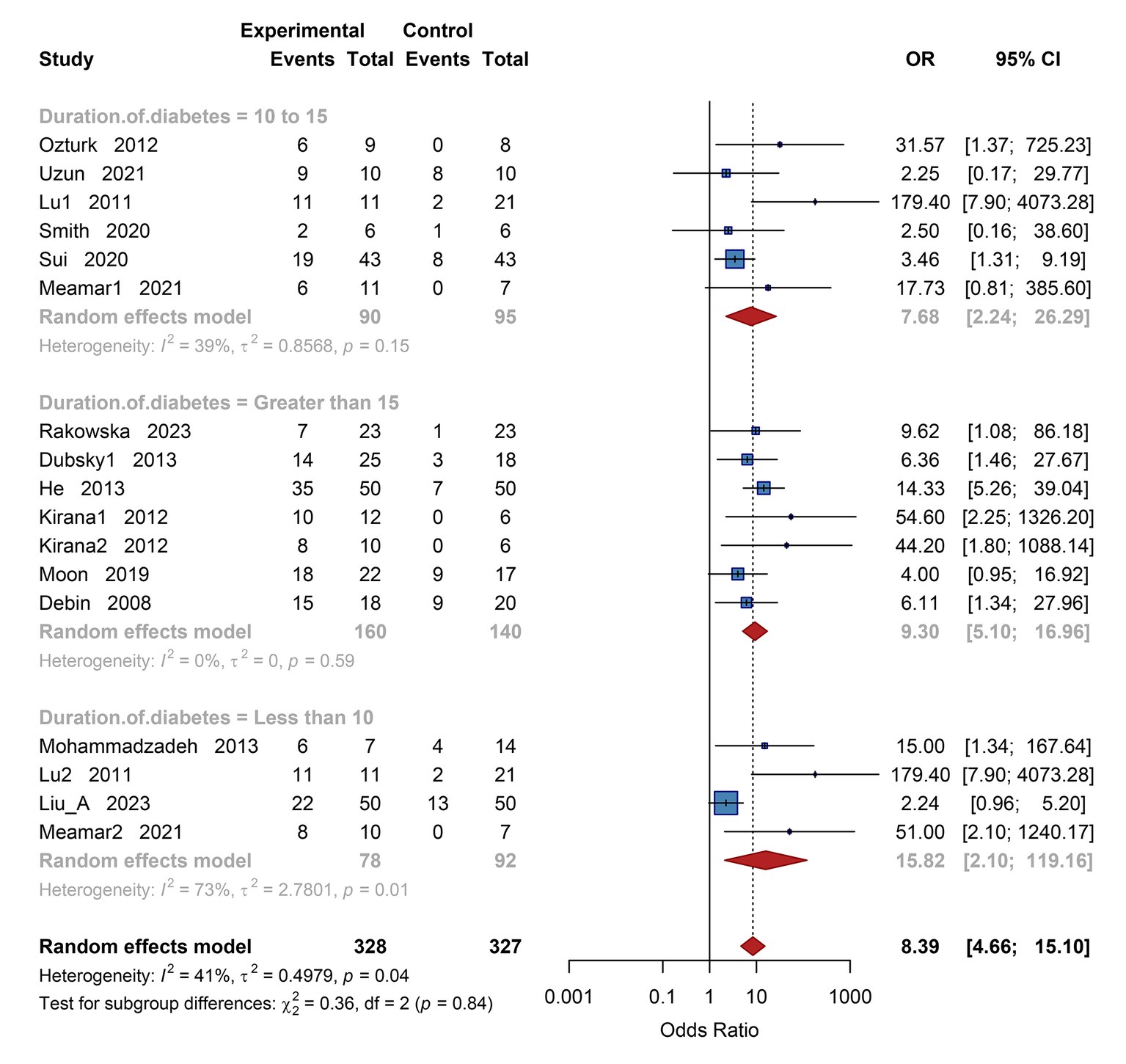


Fig S8 Ulcer healing rate based on subgroup analysis of diabetes duration, OR: Odds Ratio, CI: Confidence Interval

## Fig S9


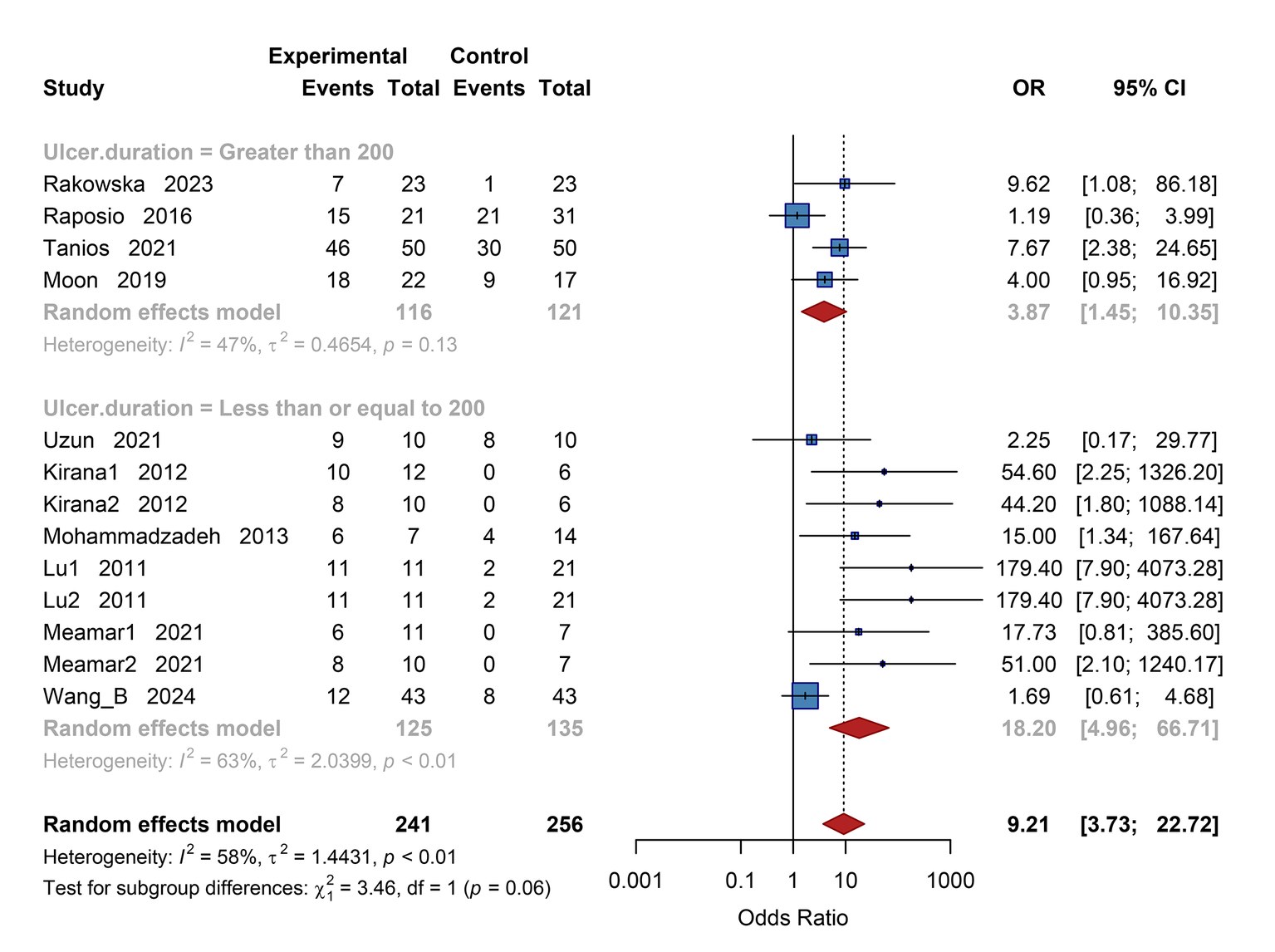


Fig S9 Ulcer healing rate based on subgroup analysis by ulcer duration, OR: Odds Ratio, CI: Confidence Interval

## Fig S10


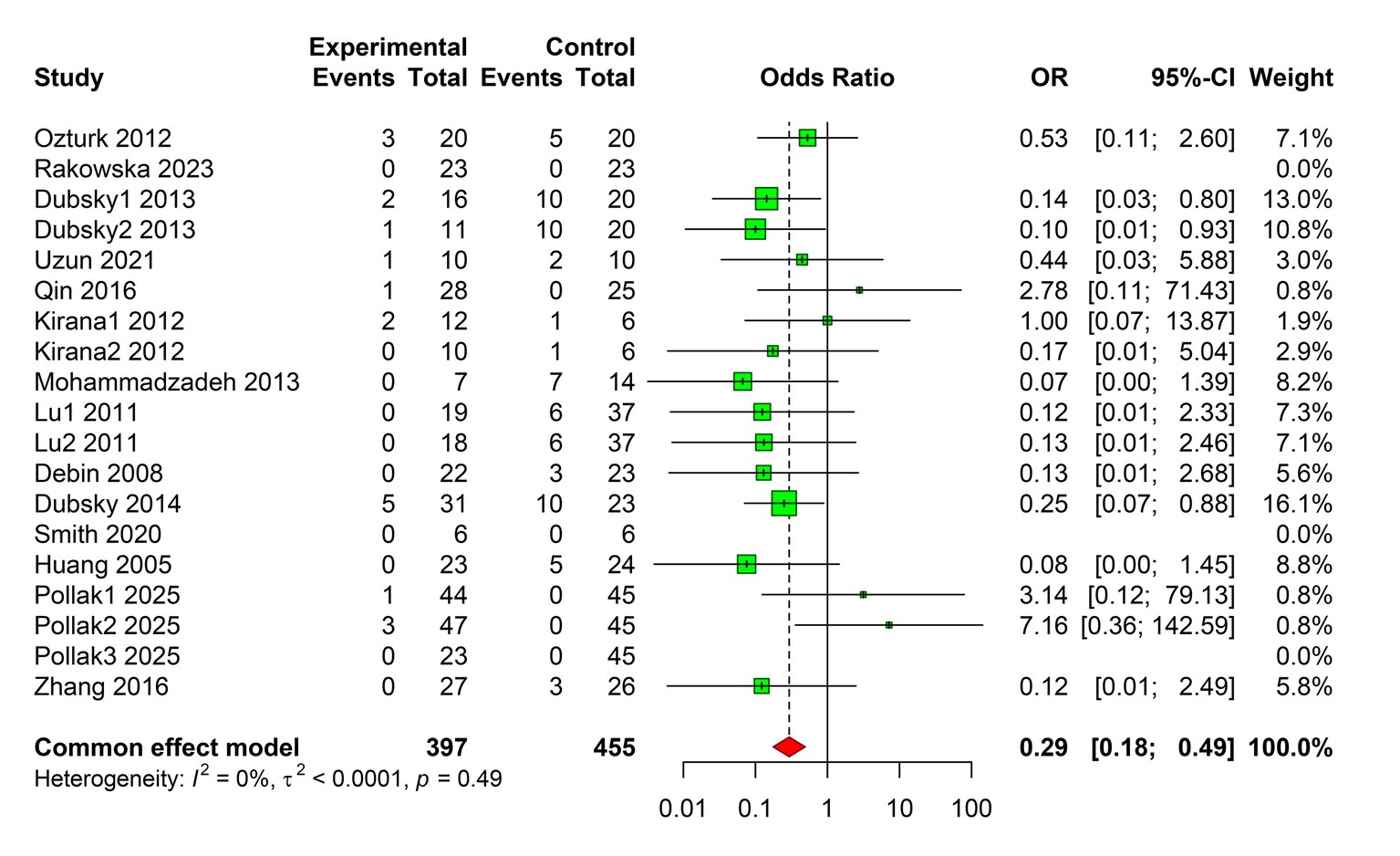


Fig S10 Overall Amputation Rate Forest Plot Analysis, OR: Odds Ratio, CI: Confidence Interval

## Fig S11


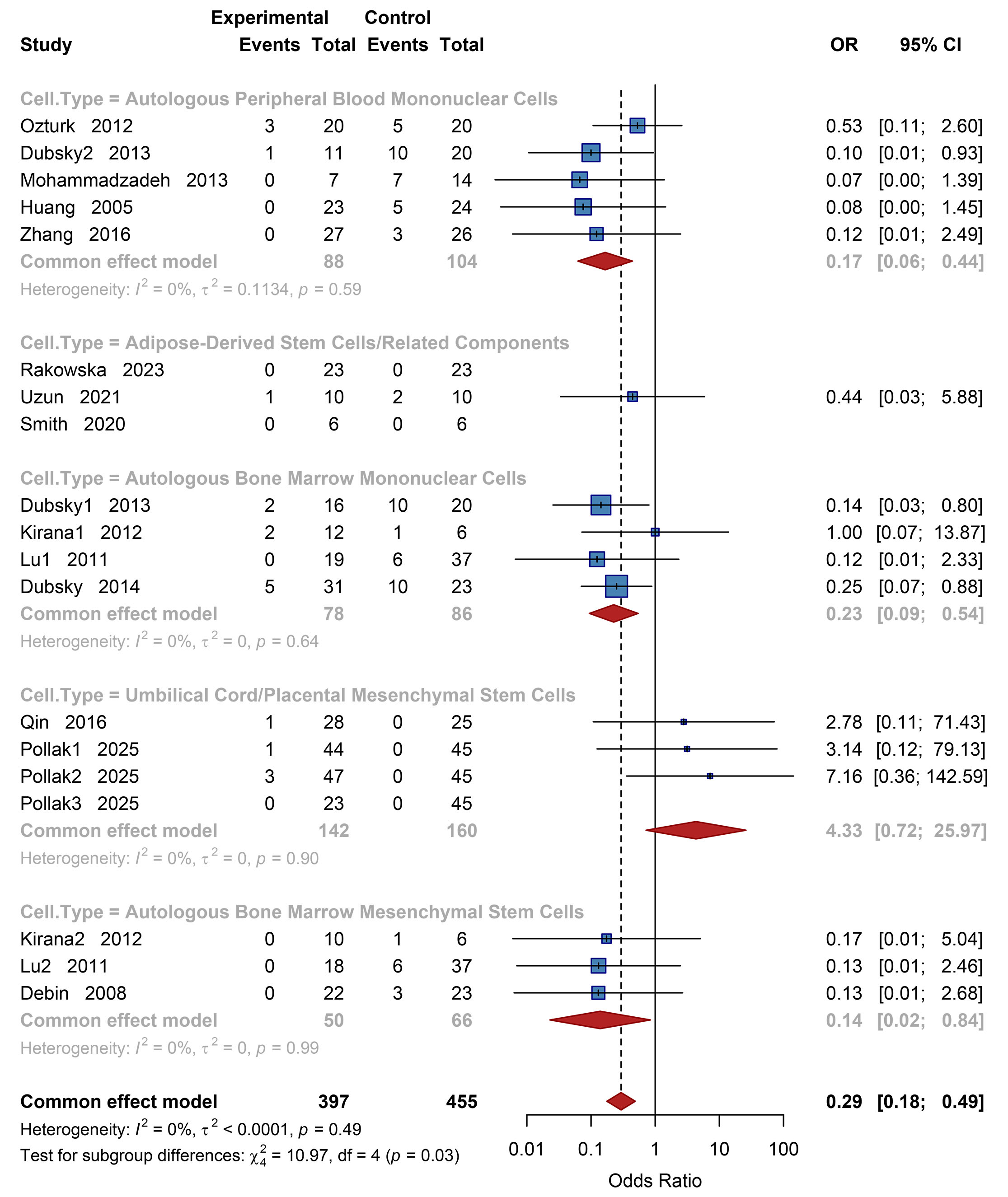


Fig S11 Subgroup Analysis of Amputation Rate by Cell Type, OR: Odds Ratio, CI: Confidence Interval

## Fig S12


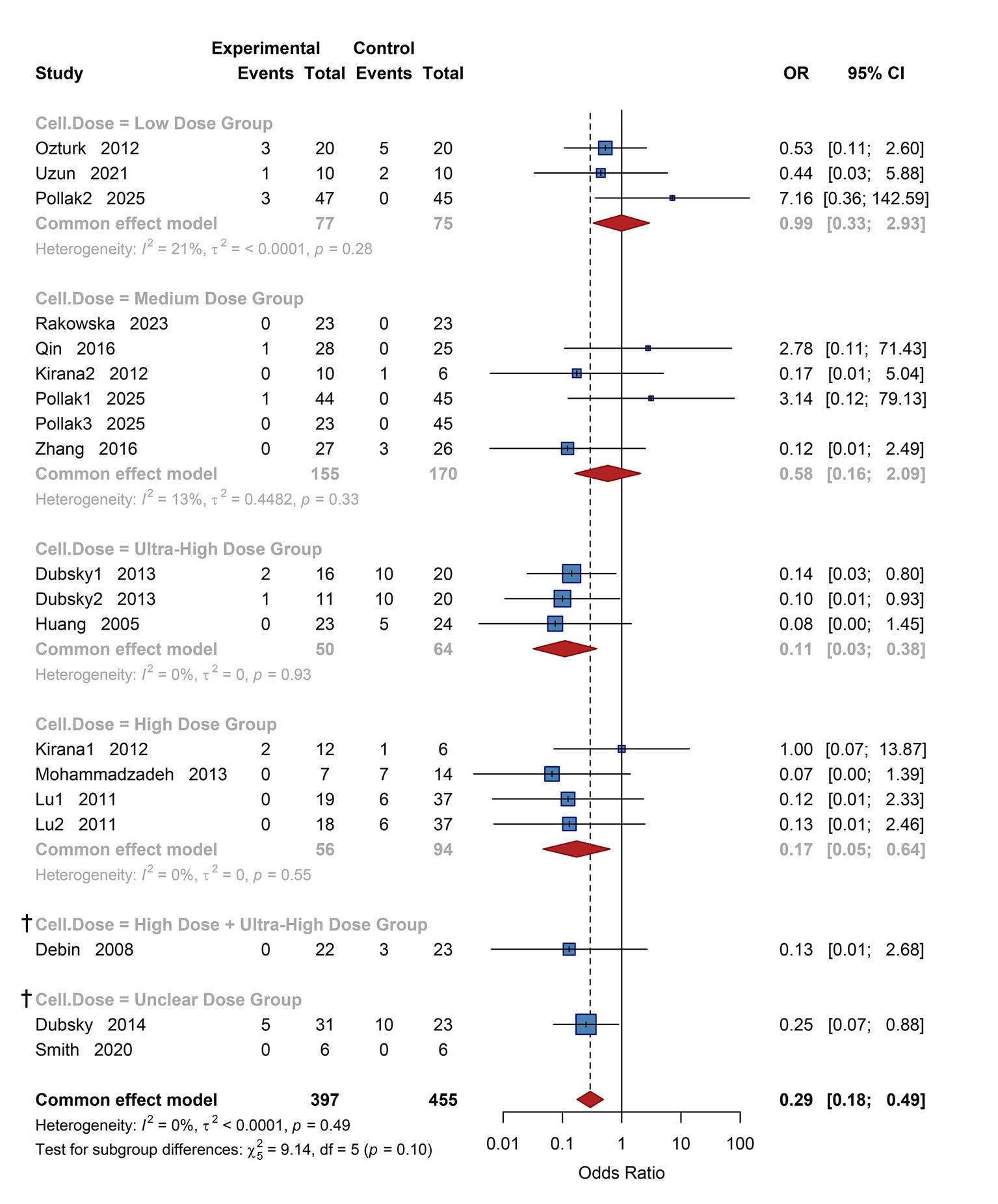


Fig S12 Subgroup Analysis of Amputation Rate by Cell Dose, OR: Odds Ratio, CI: Confidence Interval, Low-dose group: 0.5×10⁶ to 8×10⁶ cells, Medium-dose group: 1×10⁷ to 8.6×10⁷ cells, High-dose group: 3×10⁸ to 1.2×10⁹ cells, Ultra-high-dose group: ≥2×10⁹ cells, unclear dose group: No quantifiable total cell count available, † Results based on sparse data (number of studies k < 3) should be interpreted with caution.

## Fig S13


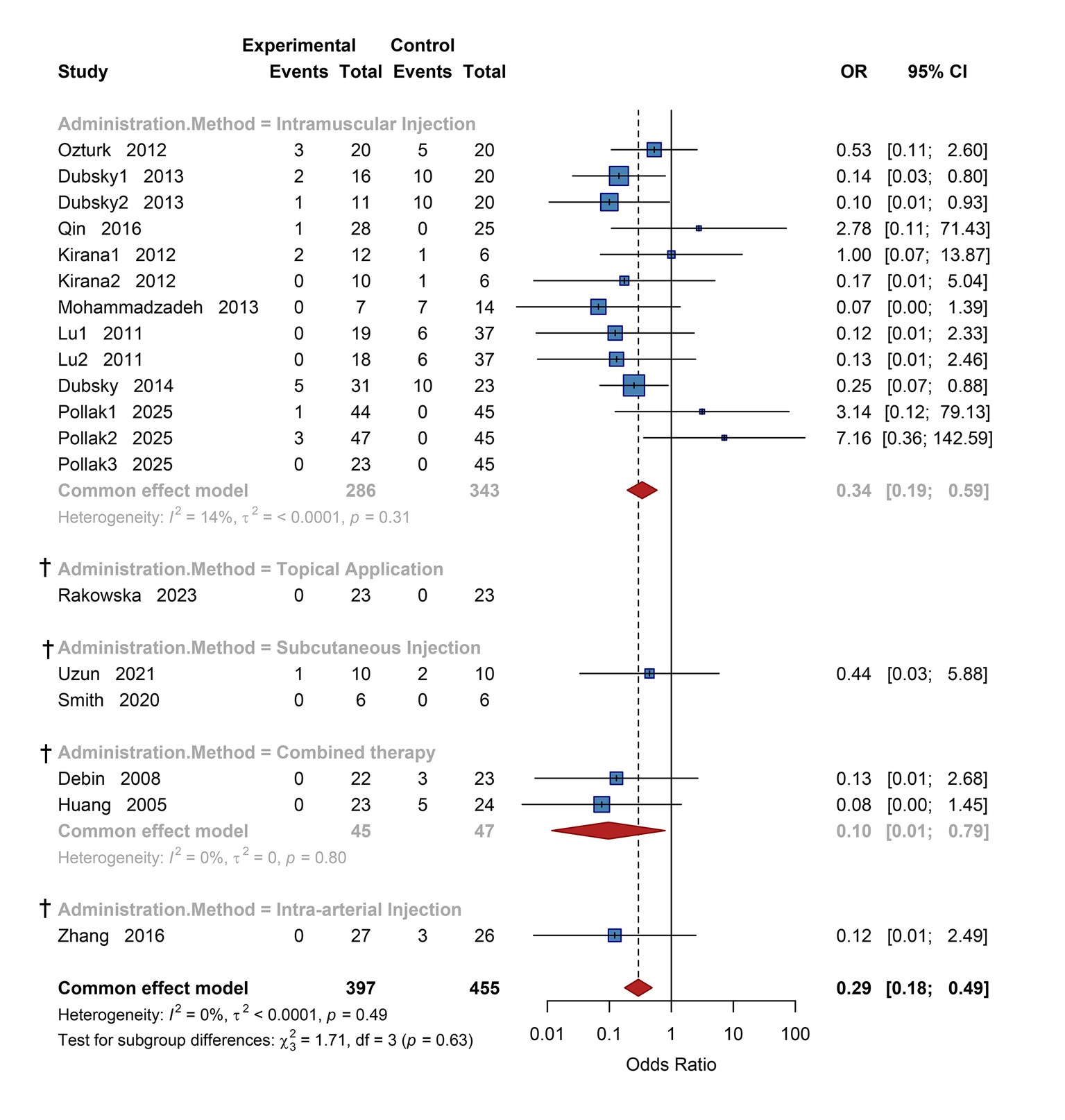


Fig S13 Subgroup Analysis of Amputation Rate by Administration Method, OR: Odds Ratio, CI: Confidence Interval, † Results based on sparse data (number of studies k < 3) should be interpreted with caution.

## Fig S14


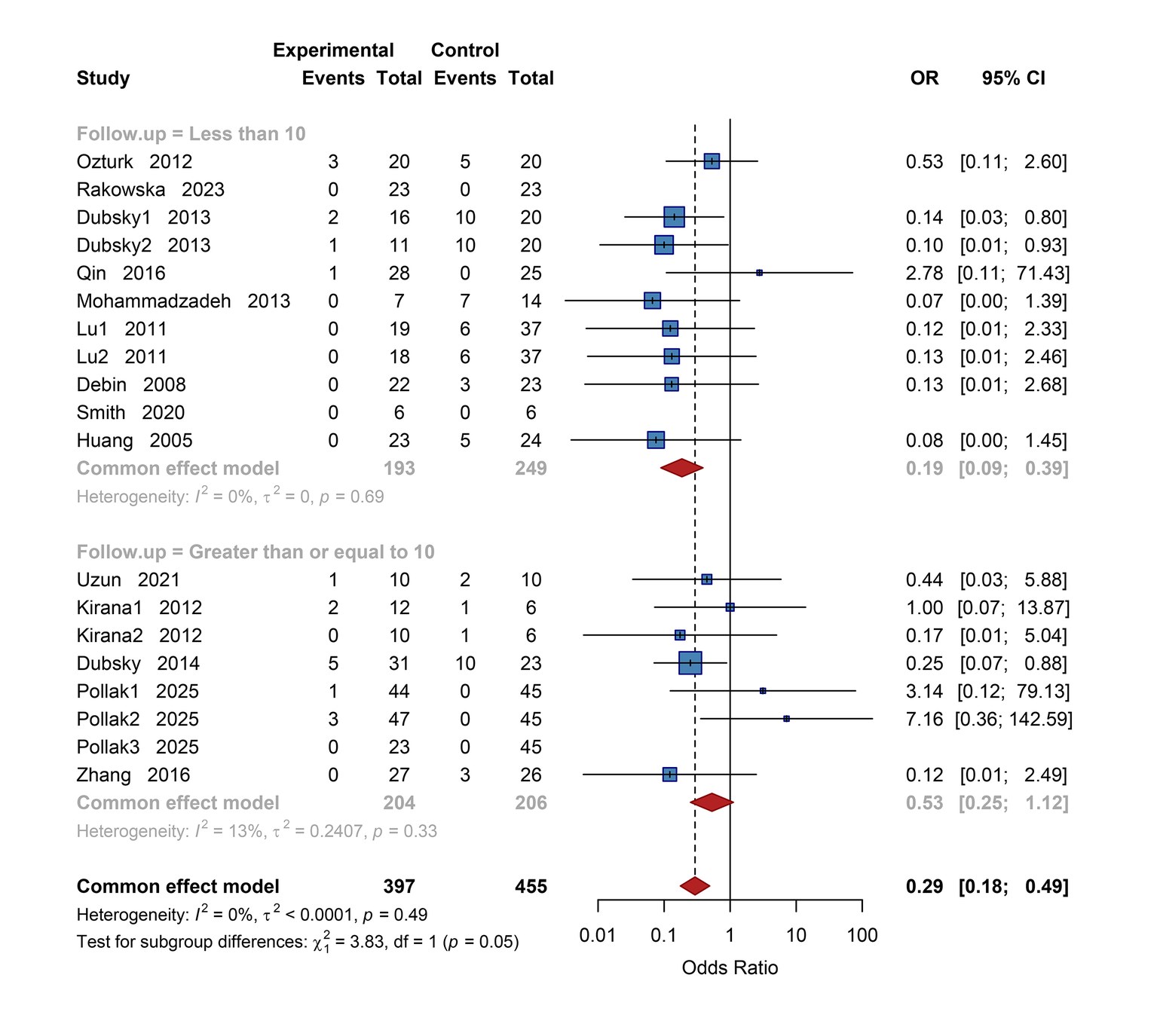


Fig S14 Subgroup Analysis of Amputation Rate by Follow-up Duration, OR: Odds Ratio, CI: Confidence Interval

## Fig S15


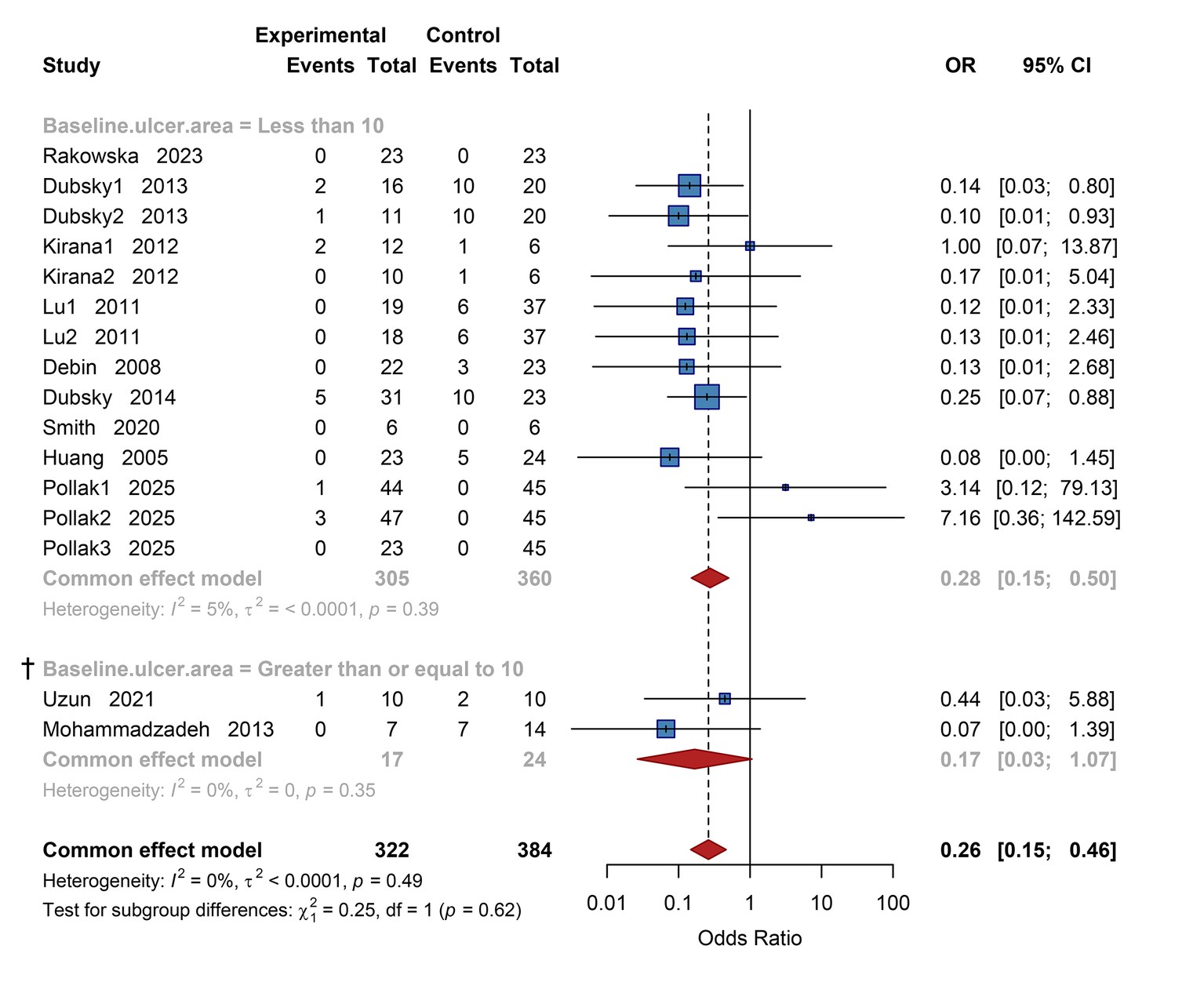


Fig S15 Subgroup Analysis of Amputation Rate by Baseline Ulcer Area, OR: Odds Ratio, CI: Confidence Interval, † Results based on sparse data (number of studies k < 3) should be interpreted with caution.

## Fig S16


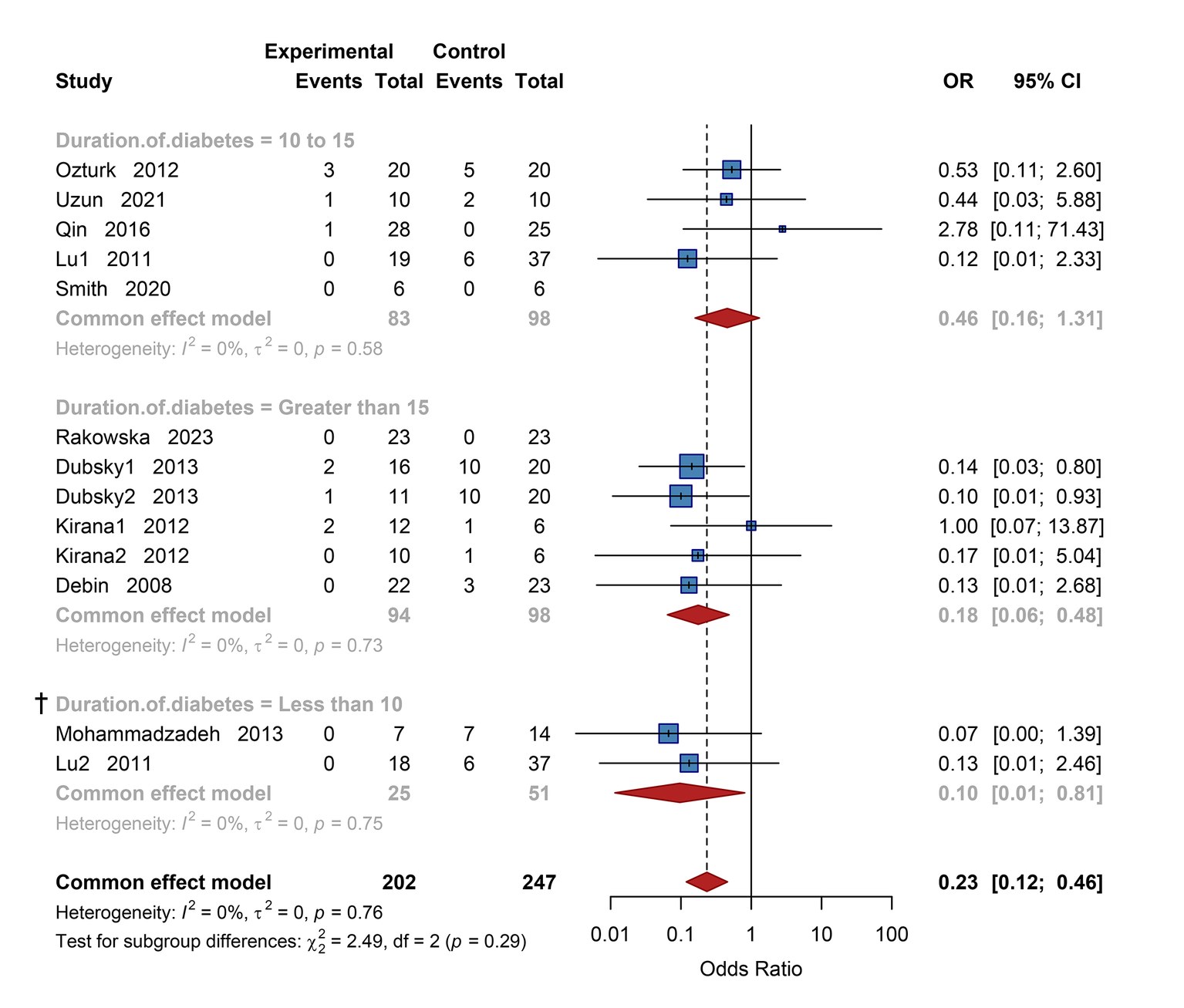


Fig S16 Subgroup Analysis of Amputation Rate by Duration of Diabetes, OR: Odds Ratio, CI: Confidence Interval, † Results based on sparse data (number of studies k < 3) should be interpreted with caution.

## Fig S17


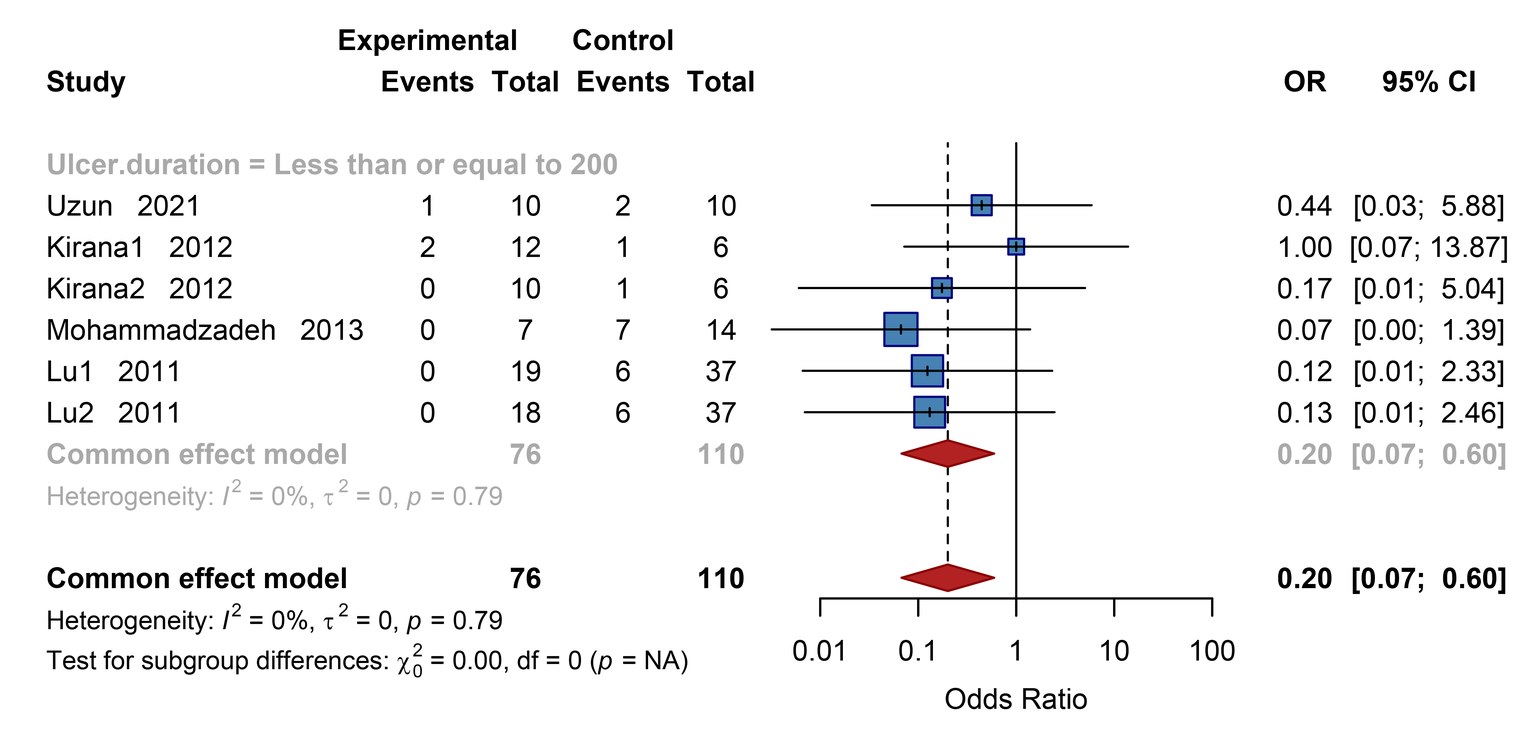


Fig S17 Subgroup Analysis of Amputation Rate by Ulcer Duration, OR: Odds Ratio, CI: Confidence Interval

## Fig S18


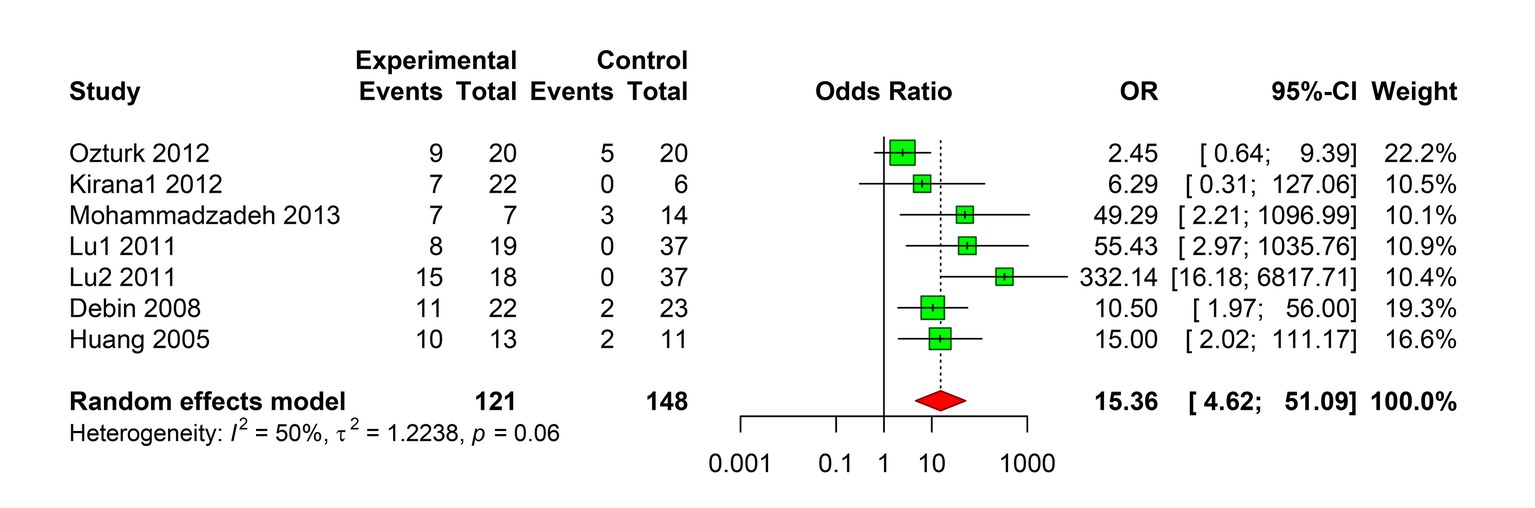


Fig S18 Overall Neovascularization Rate Forest Plot Analysis, OR: Odds Ratio, CI: Confidence Interval

## Fig S19


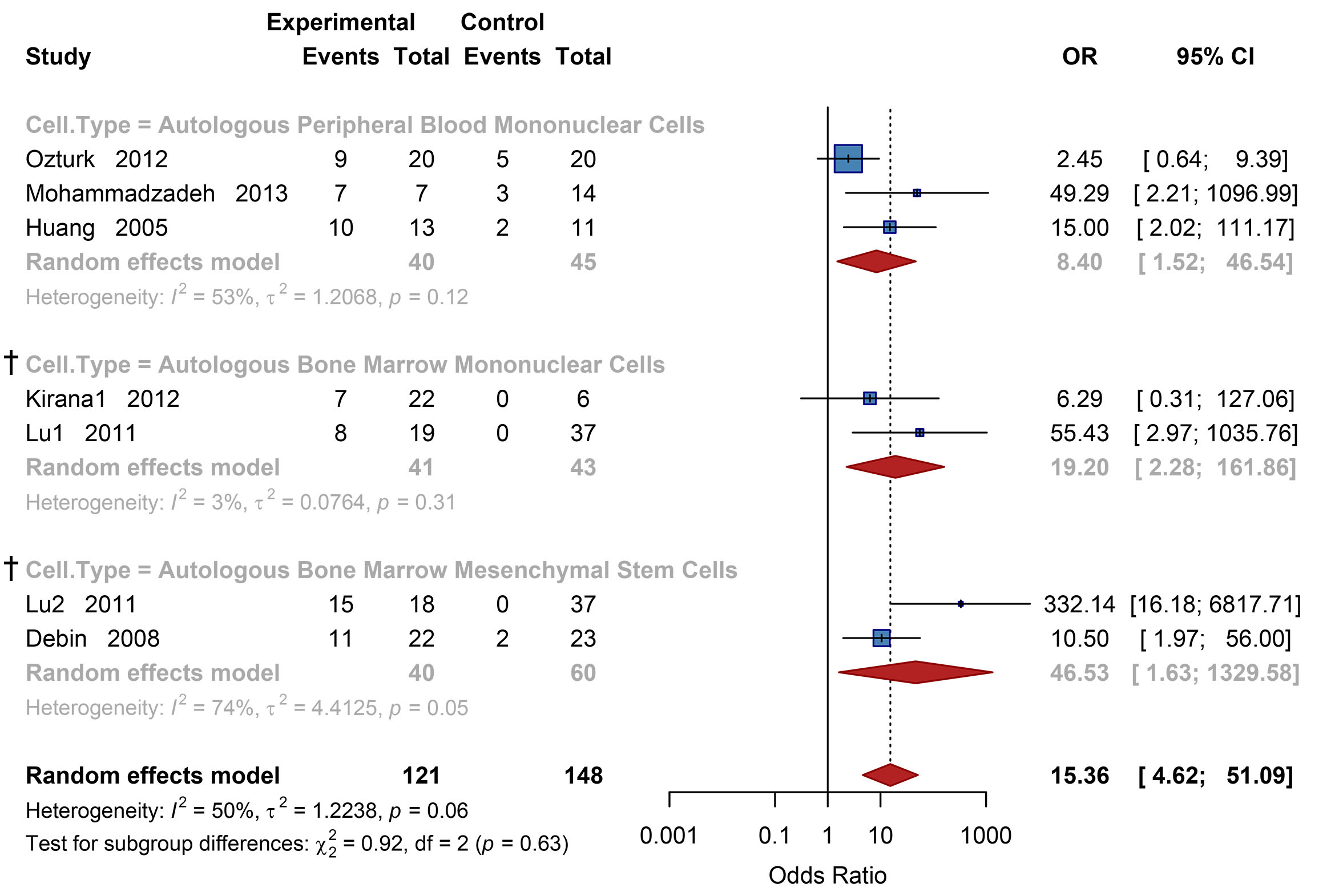


Fig S19 Subgroup Analysis of Neovascularization Rate by Cell Type, OR: Odds Ratio, CI: Confidence Interval, † Results based on sparse data (number of studies k < 3) should be interpreted with caution.

## Fig S20


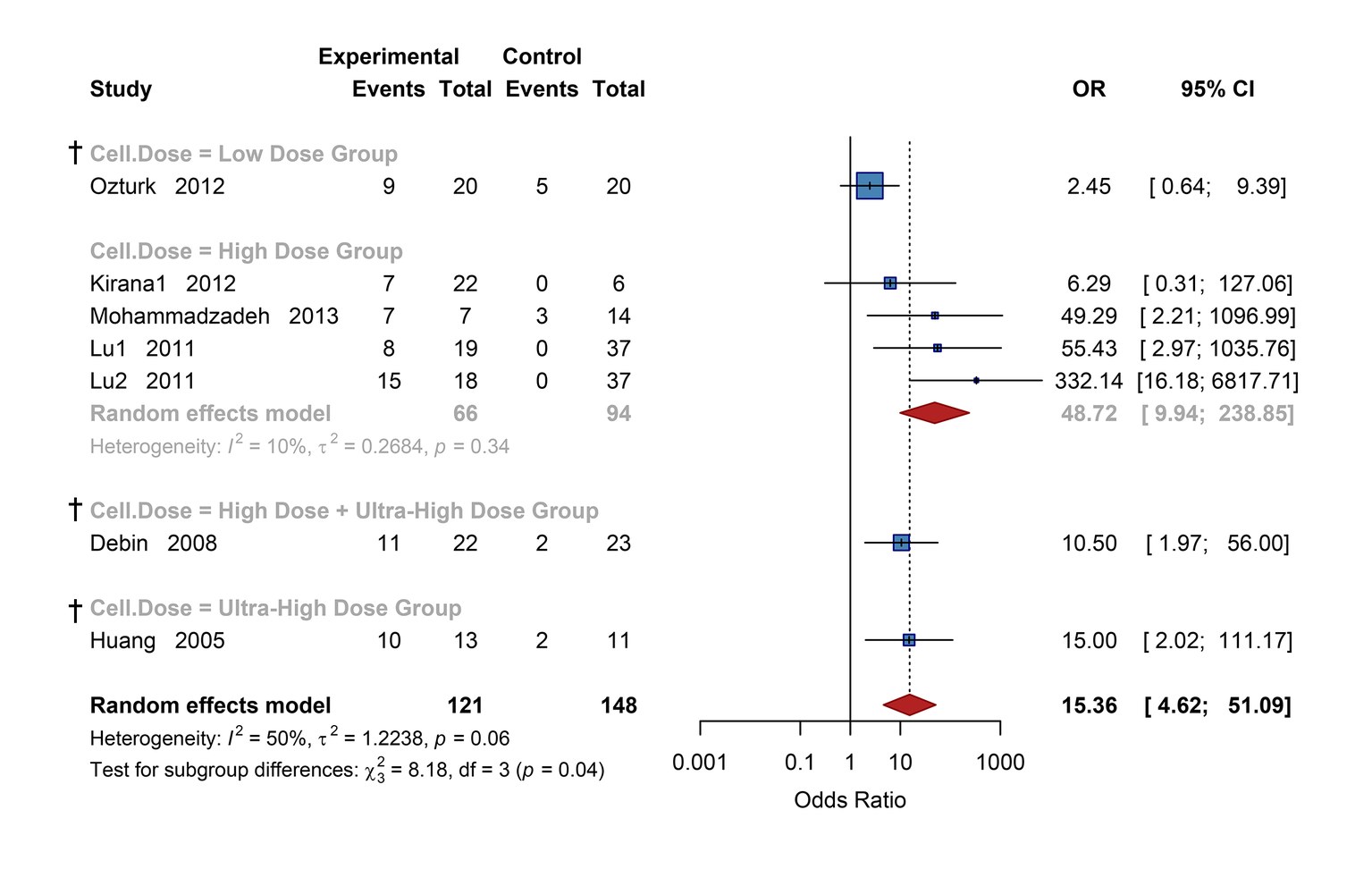


Fig S20 Subgroup Analysis of Neovascularization Rate by Cell Dose, OR: Odds Ratio, CI: Confidence Interval, Low-dose group: 0.5×10⁶ to 8×10⁶ cells, Medium-dose group: 1×10⁷ to 8.6×10⁷ cells, High-dose group: 3×10⁸ to 1.2×10⁹ cells, Ultra-high-dose group: ≥2×10⁹ cells, unclear dose group: No quantifiable total cell count available, † Results based on sparse data (number of studies k < 3) should be interpreted with caution.

## Fig S21


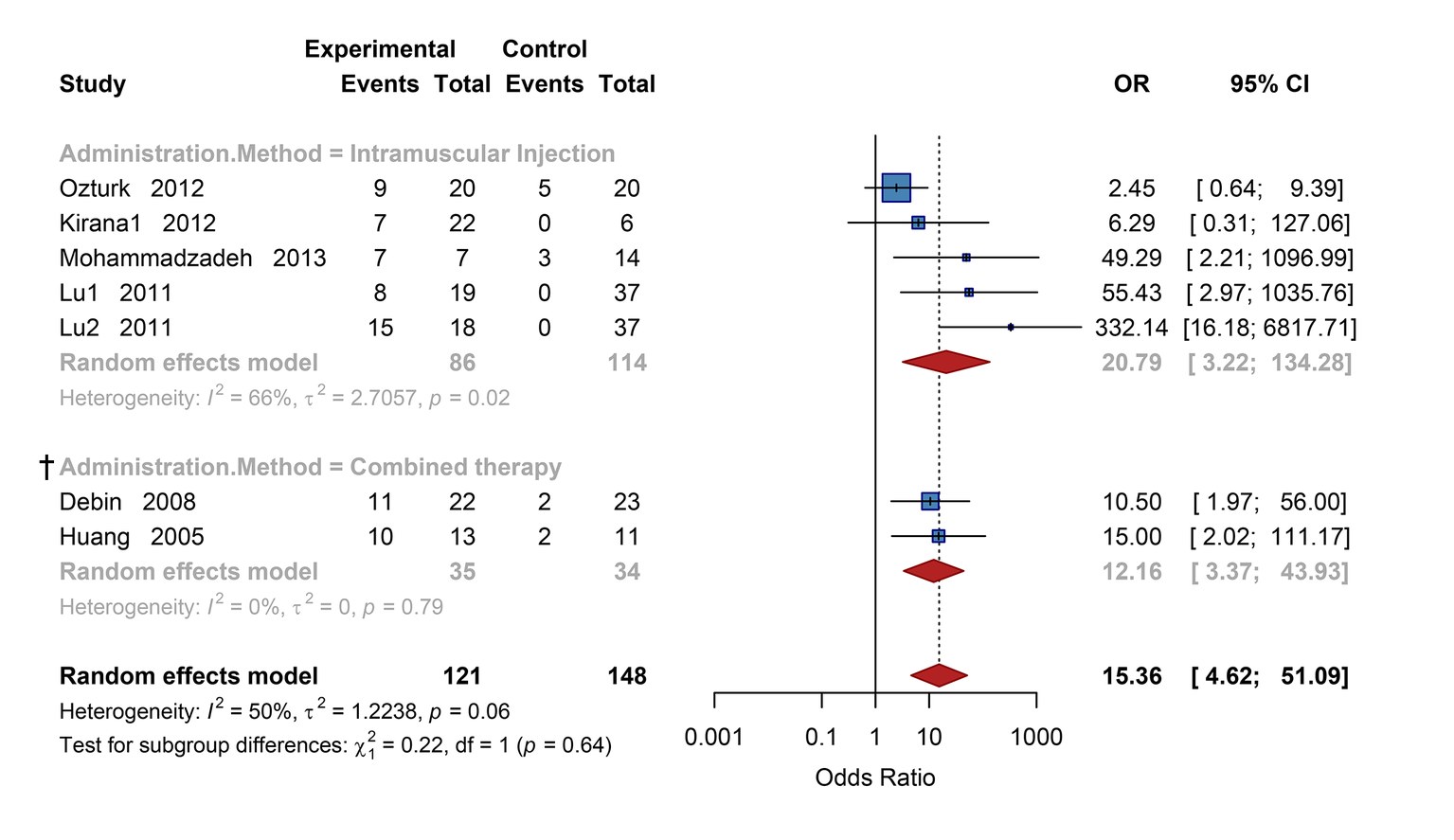


Fig S21 Subgroup Analysis of Neovascularization Rate by Administration Method, OR: Odds Ratio, CI: Confidence Interval, † Results based on sparse data (number of studies k < 3) should be interpreted with caution.

## Fig S22


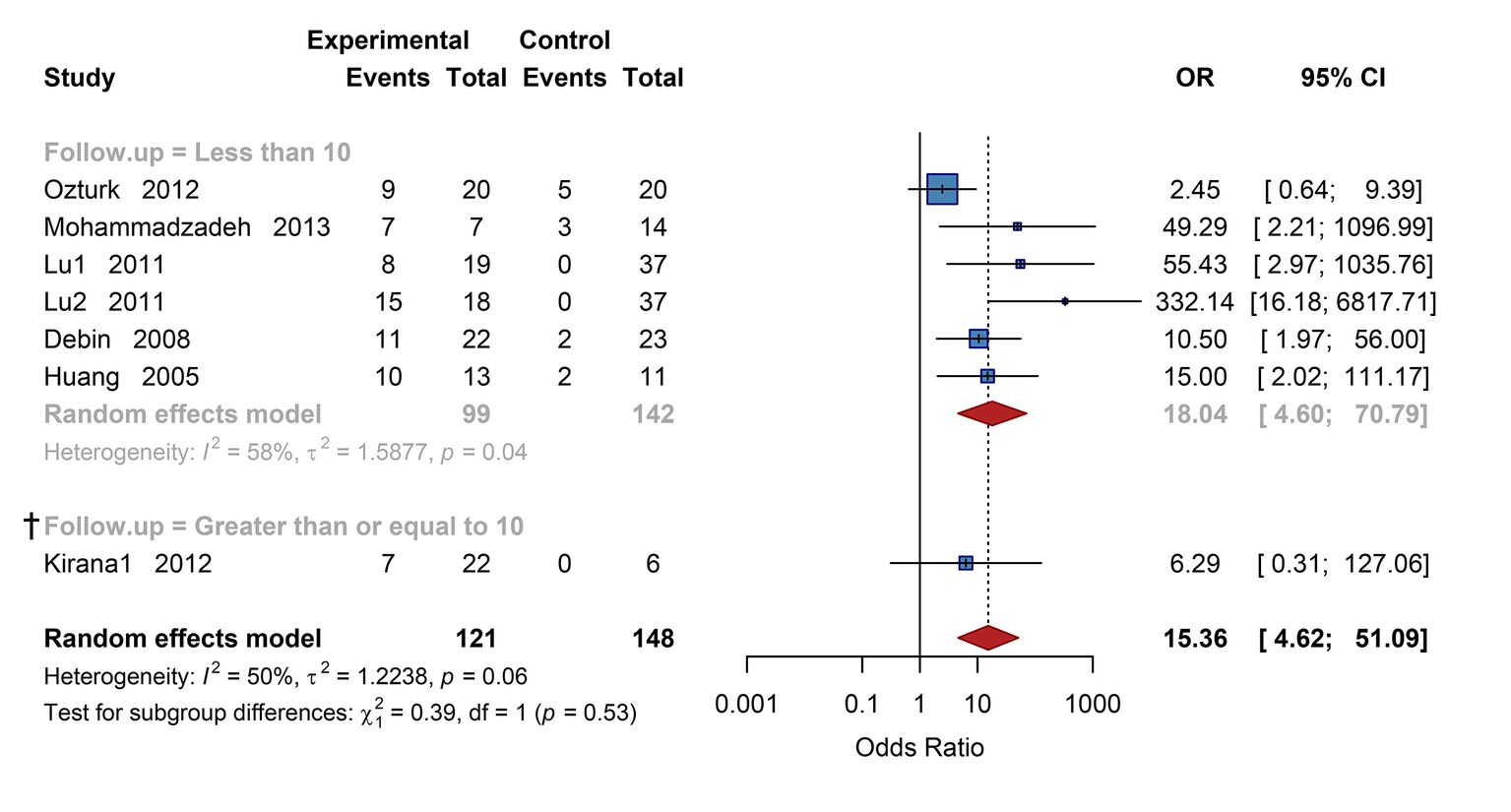


Fig S22 Subgroup Analysis of Neovascularization Rate by Follow-up Duration, OR: Odds Ratio, CI: Confidence Interval, † Results based on sparse data (number of studies k < 3) should be interpreted with caution.

## Fig S23


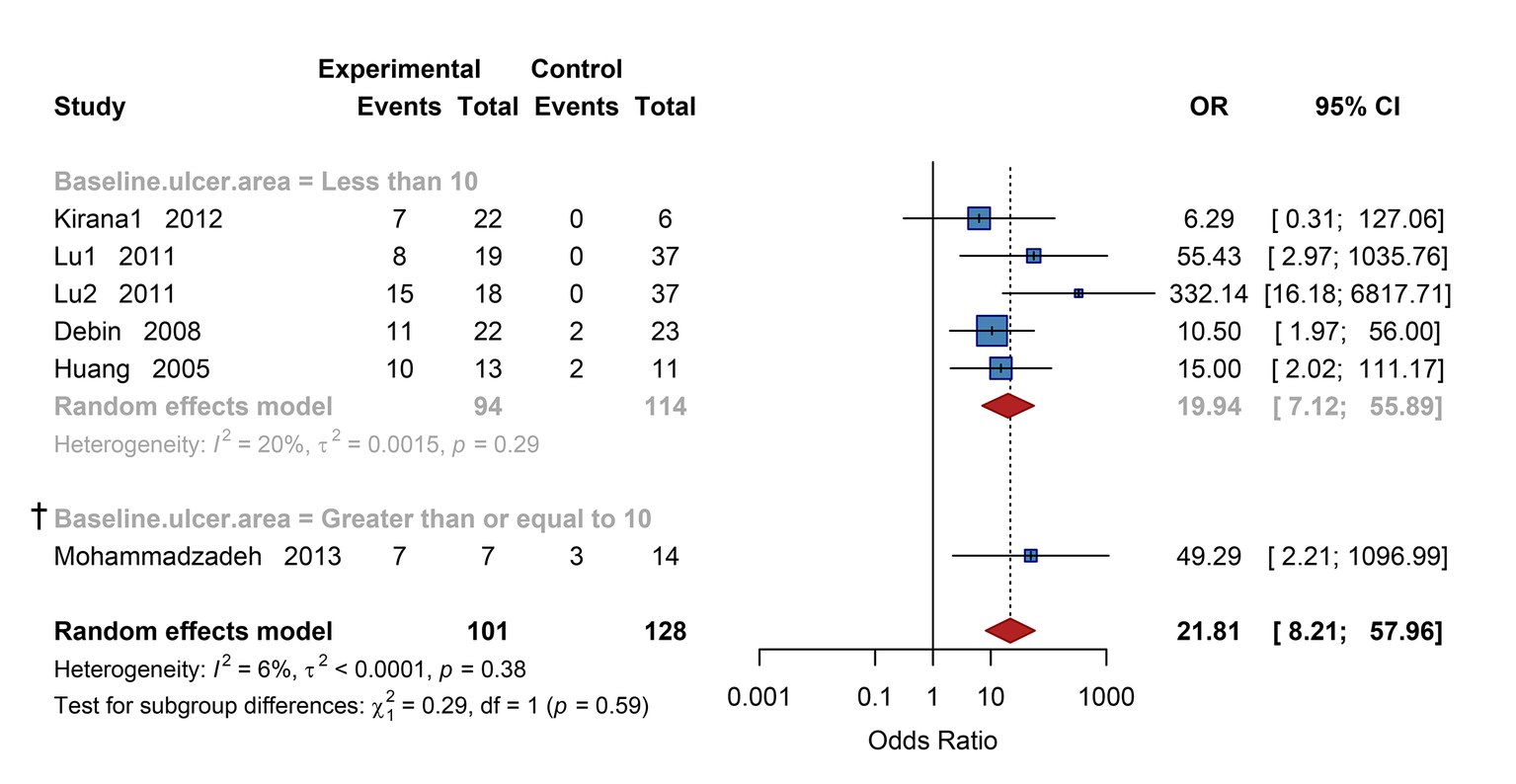


Fig S23 Subgroup Analysis of Neovascularization Rate by Baseline Ulcer Area, OR: Odds Ratio, CI: Confidence Interval, † Results based on sparse data (number of studies k < 3) should be interpreted with caution.

## Fig S24


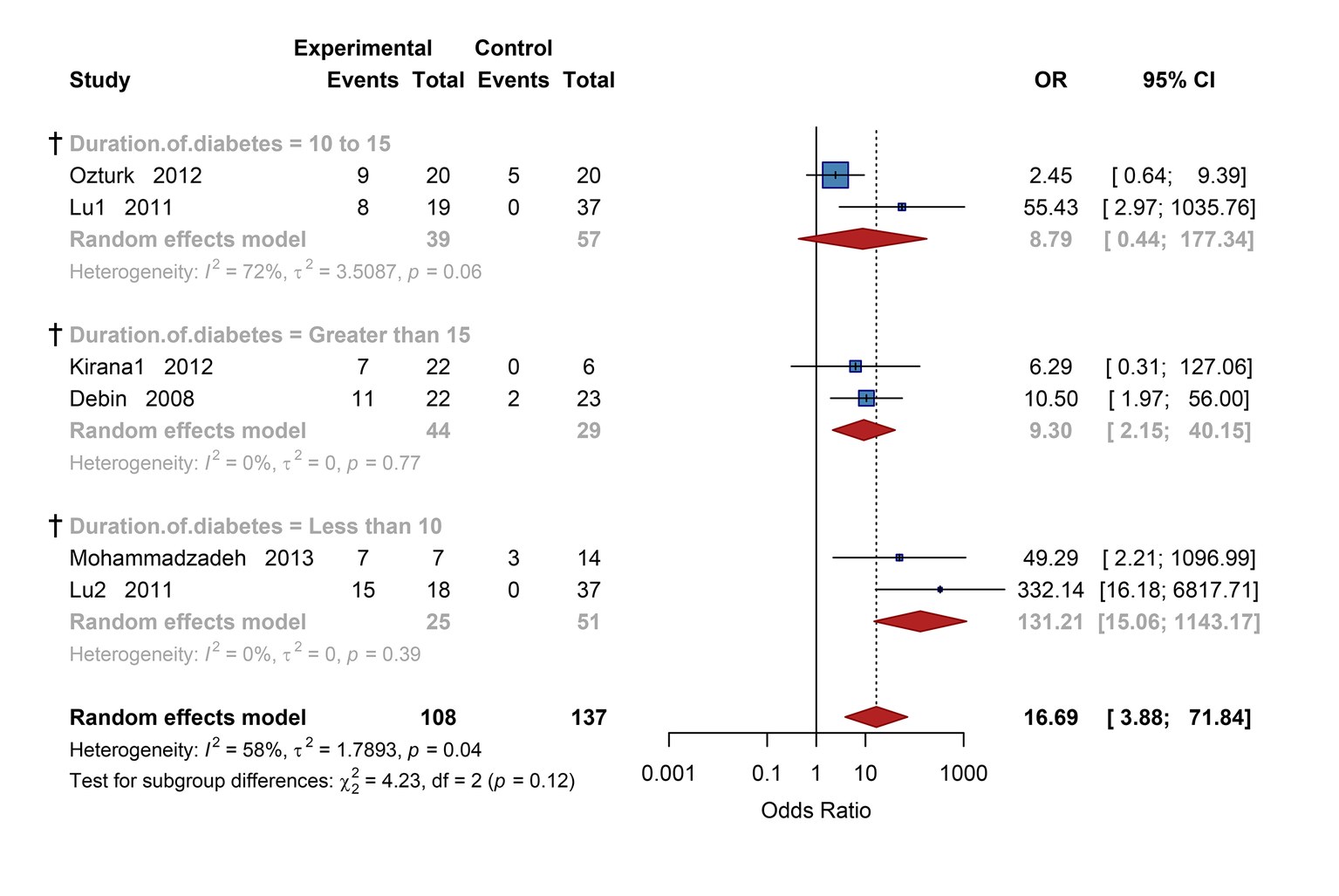


Fig S24 Subgroup Analysis of Neovascularization Rate by Duration of Diabetes, OR: Odds Ratio, CI: Confidence Interval, † Results based on sparse data (number of studies k < 3) should be interpreted with caution.

## Fig S25


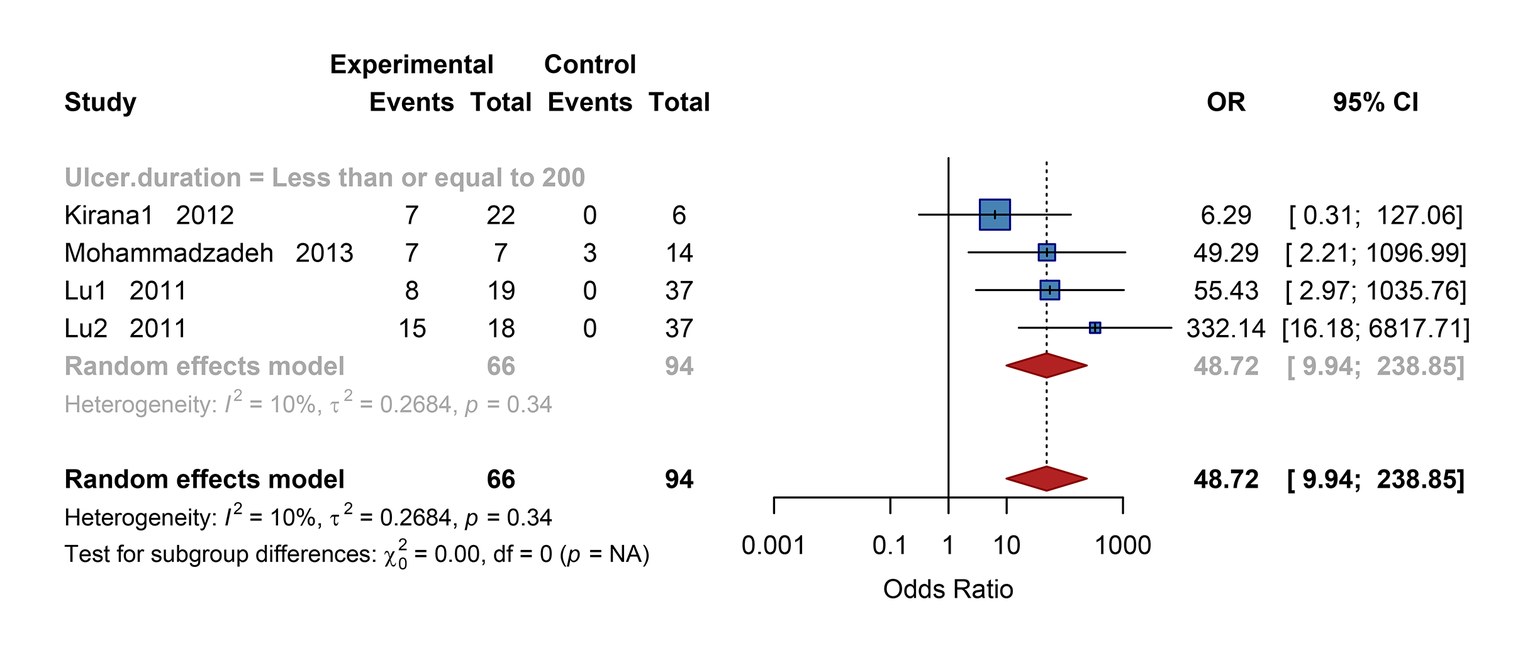


Fig S25 Subgroup Analysis of Neovascularization Rate by Ulcer Duration, OR: Odds Ratio, CI: Confidence Interval

## Fig S26


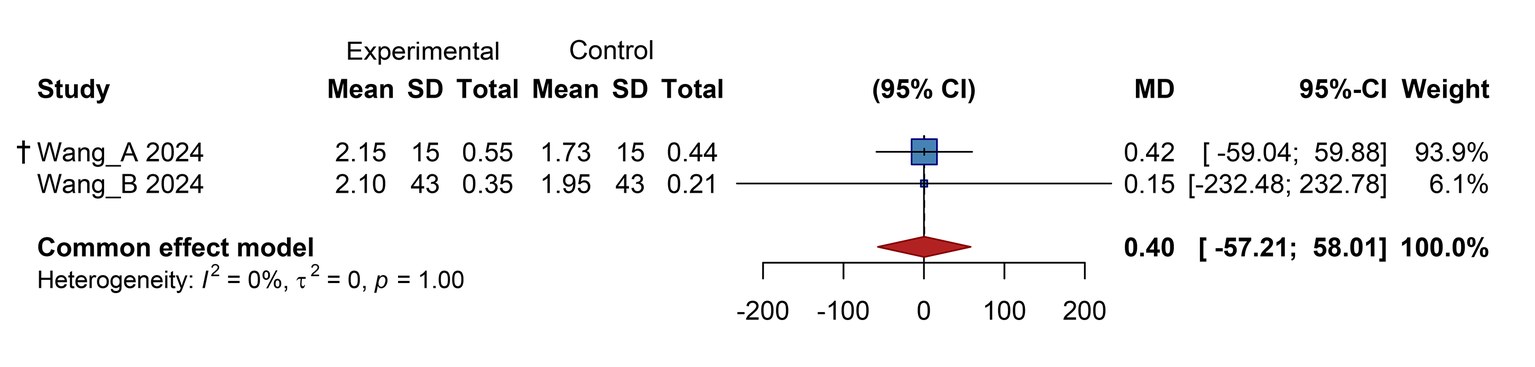


Fig S26 Meta-analysis of neovascularization scores, SD: Standard Deviation, † Results based on sparse data (number of studies k < 3) should be interpreted with caution.

## Fig S27


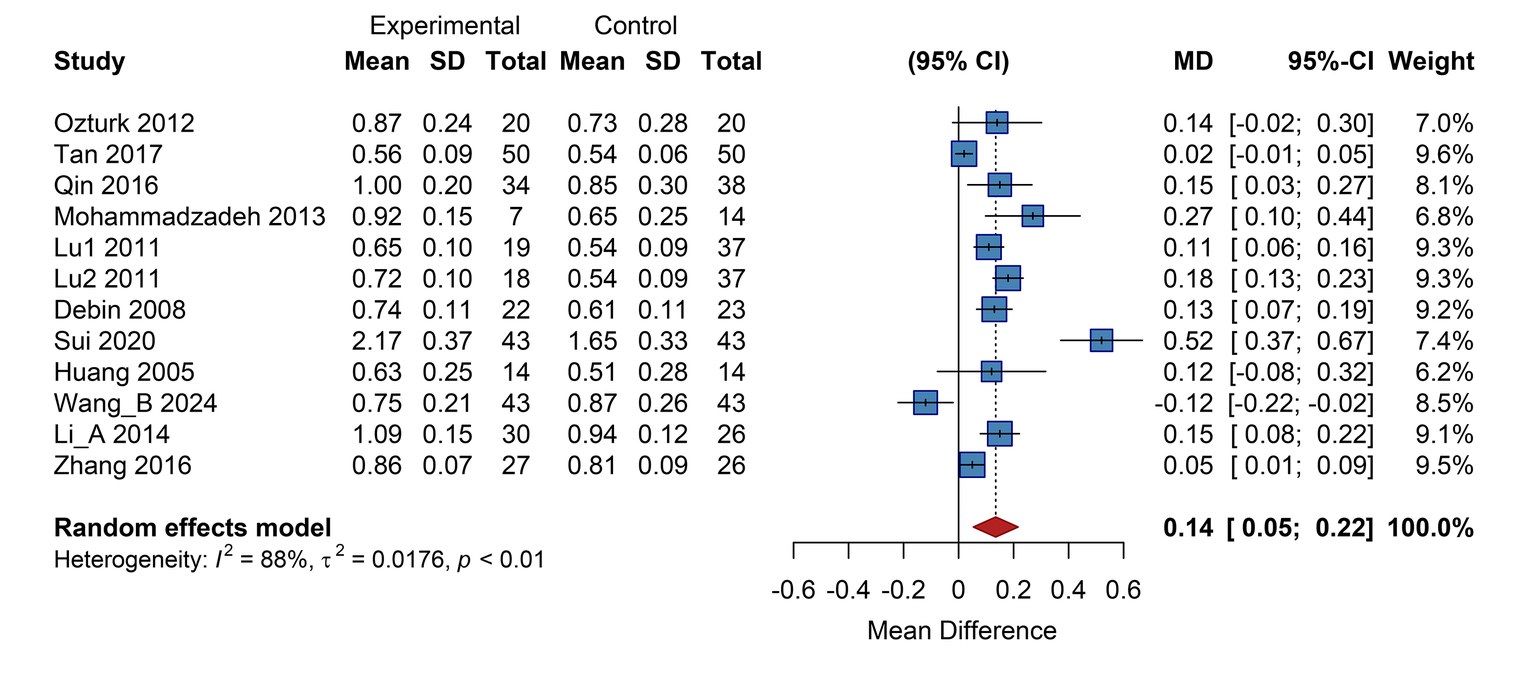


Fig S27 Overall ABI Forest Plot Analysis, SD: Standard Deviation

## Fig S28


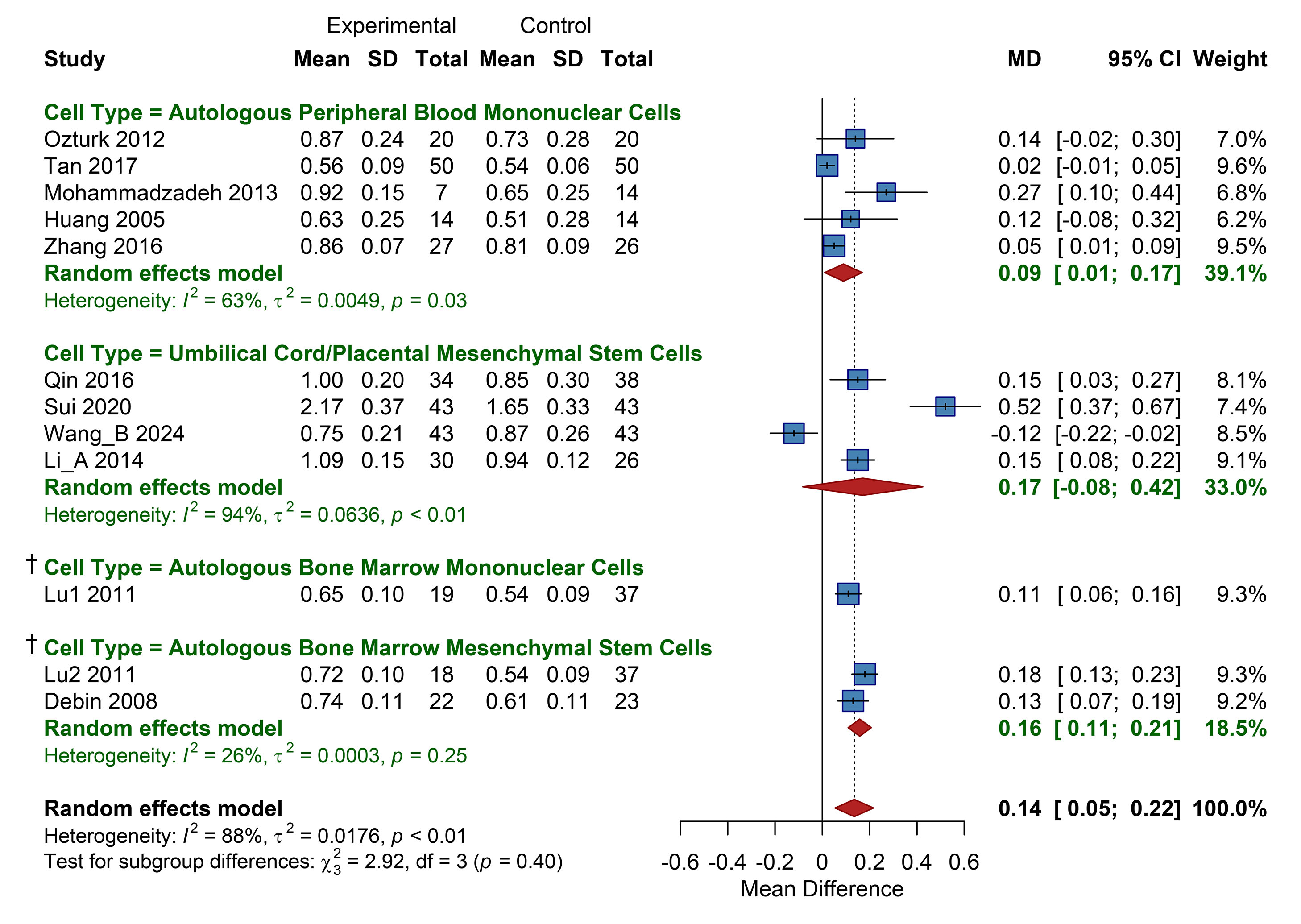


Fig S28 Subgroup Analysis of ABI by Cell Type, SD: Standard Deviation, † Results based on sparse data (number of studies k < 3) should be interpreted with caution.

## Fig S29


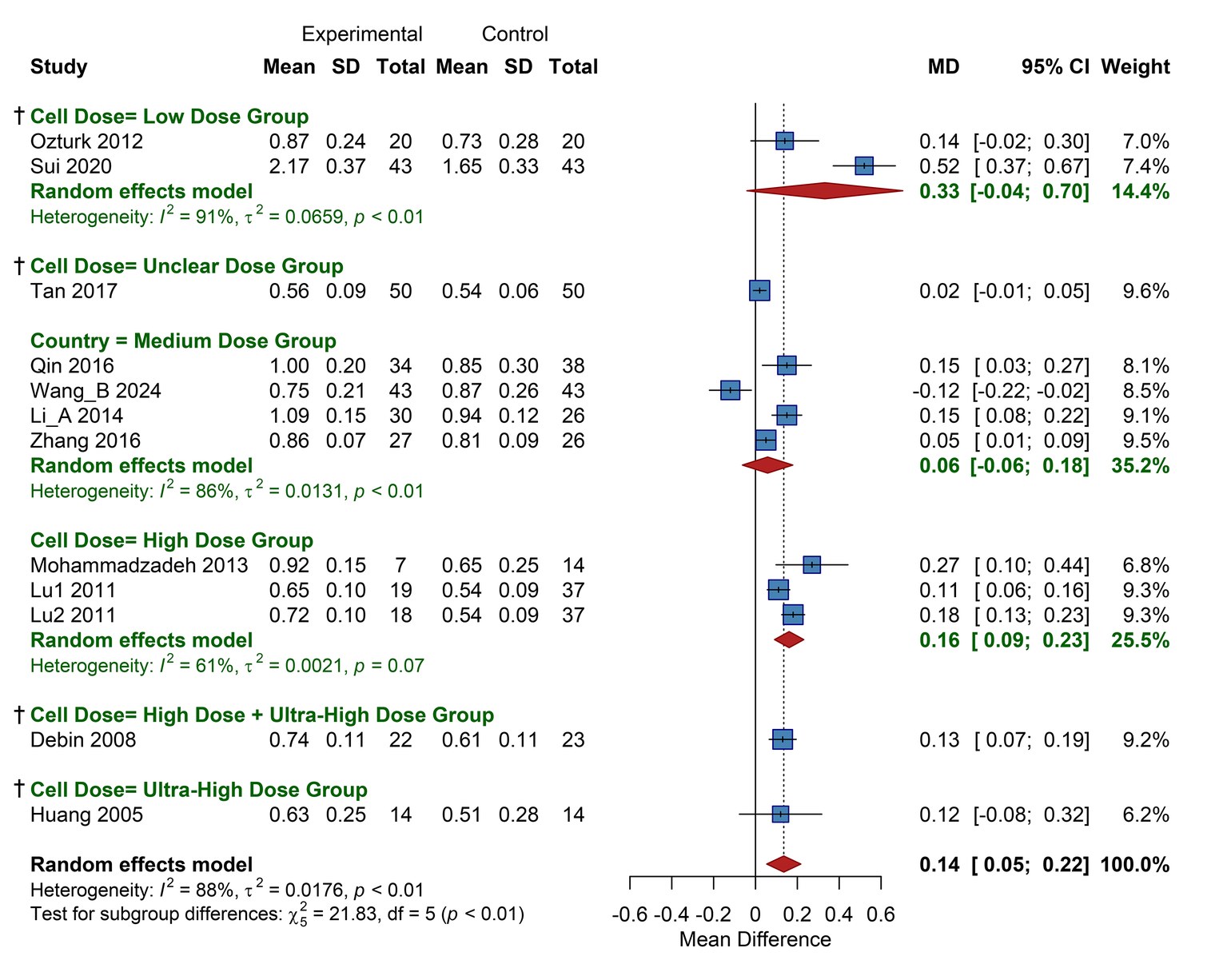


Fig S29 Subgroup Analysis of ABI by Cell Dose, SD: Standard Deviation, † Results based on sparse data (number of studies k < 3) should be interpreted with caution.

## Fig S30


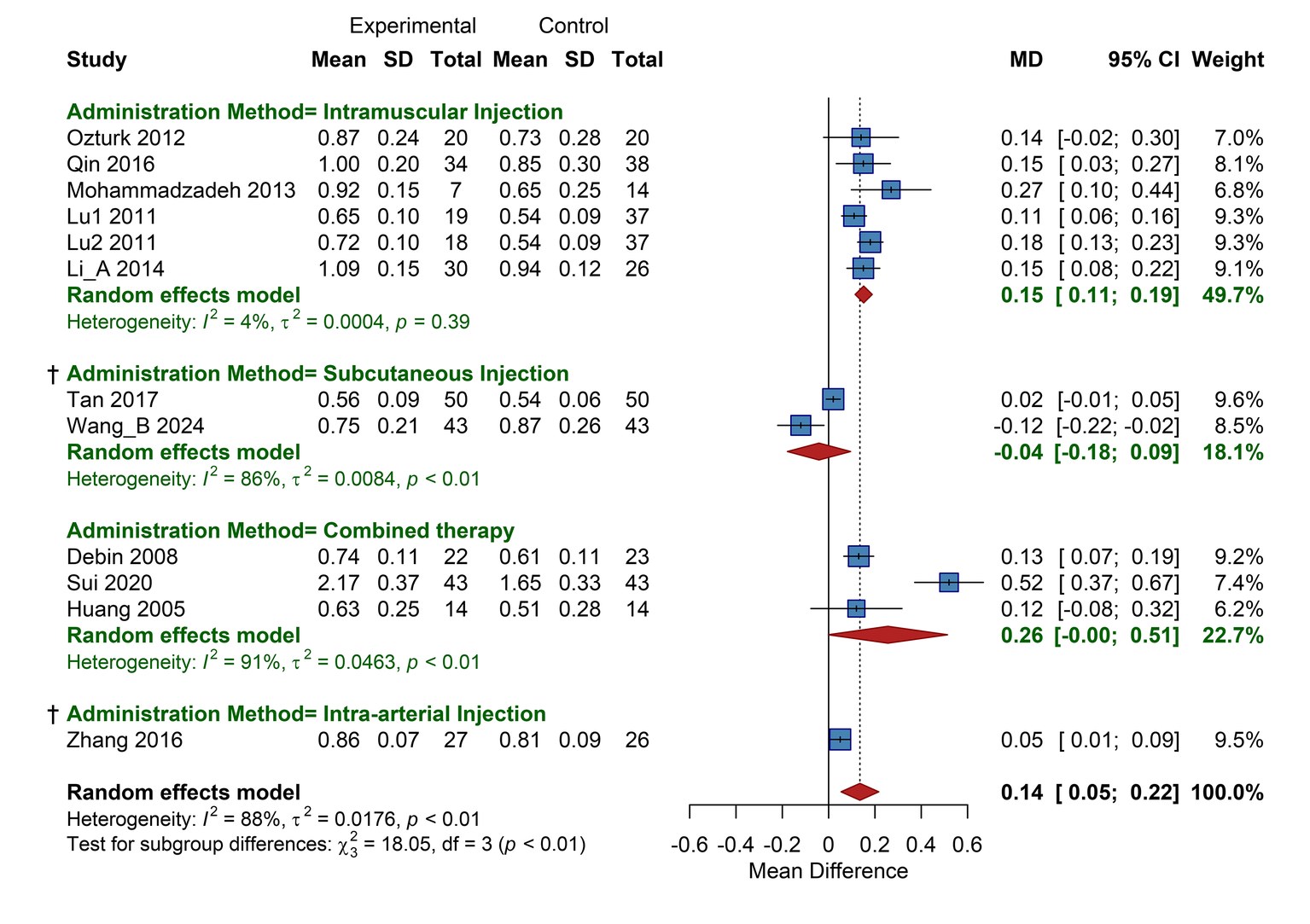


Fig S30 Subgroup Analysis of ABI by Administration Method, SD: Standard Deviation, † Results based on sparse data (number of studies k < 3) should be interpreted with caution.

## Fig S31


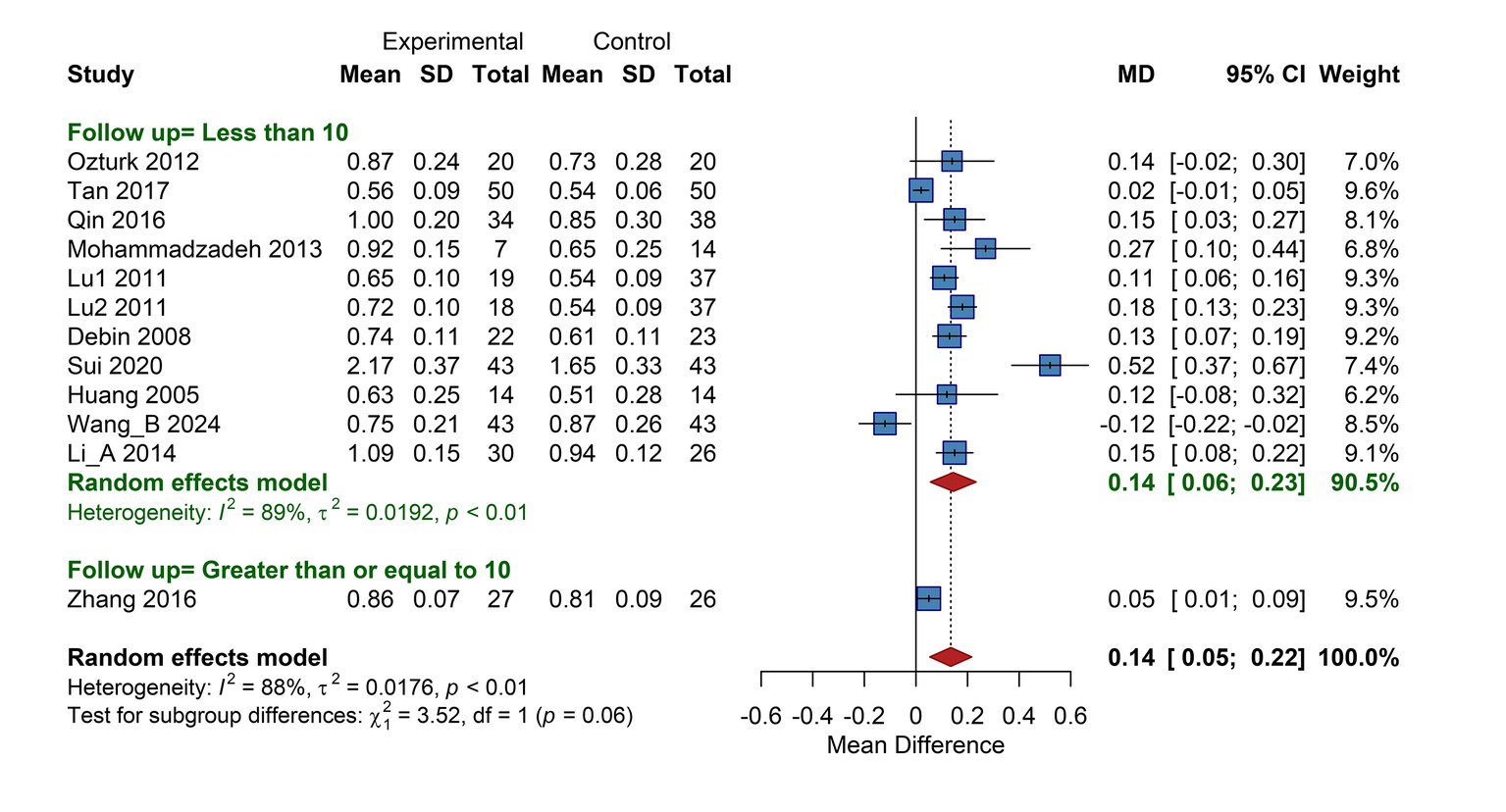


Fig S31 Subgroup Analysis of ABI by Follow-up Duration, SD: Standard Deviation, † Results based on sparse data (number of studies k < 3) should be interpreted with caution.

## Fig S32


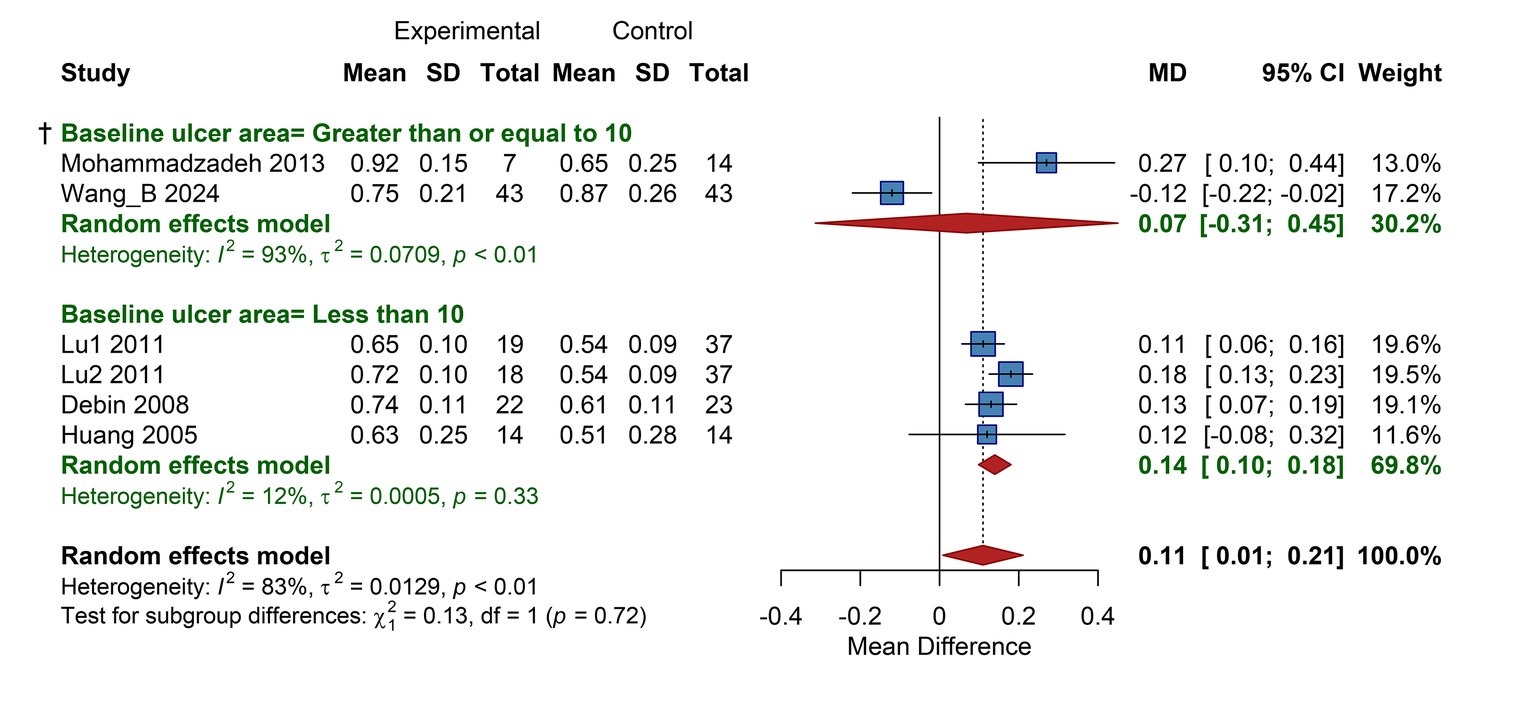


Fig S32 Subgroup Analysis of ABI by Baseline Ulcer Area, SD: Standard Deviation

## Fig S33


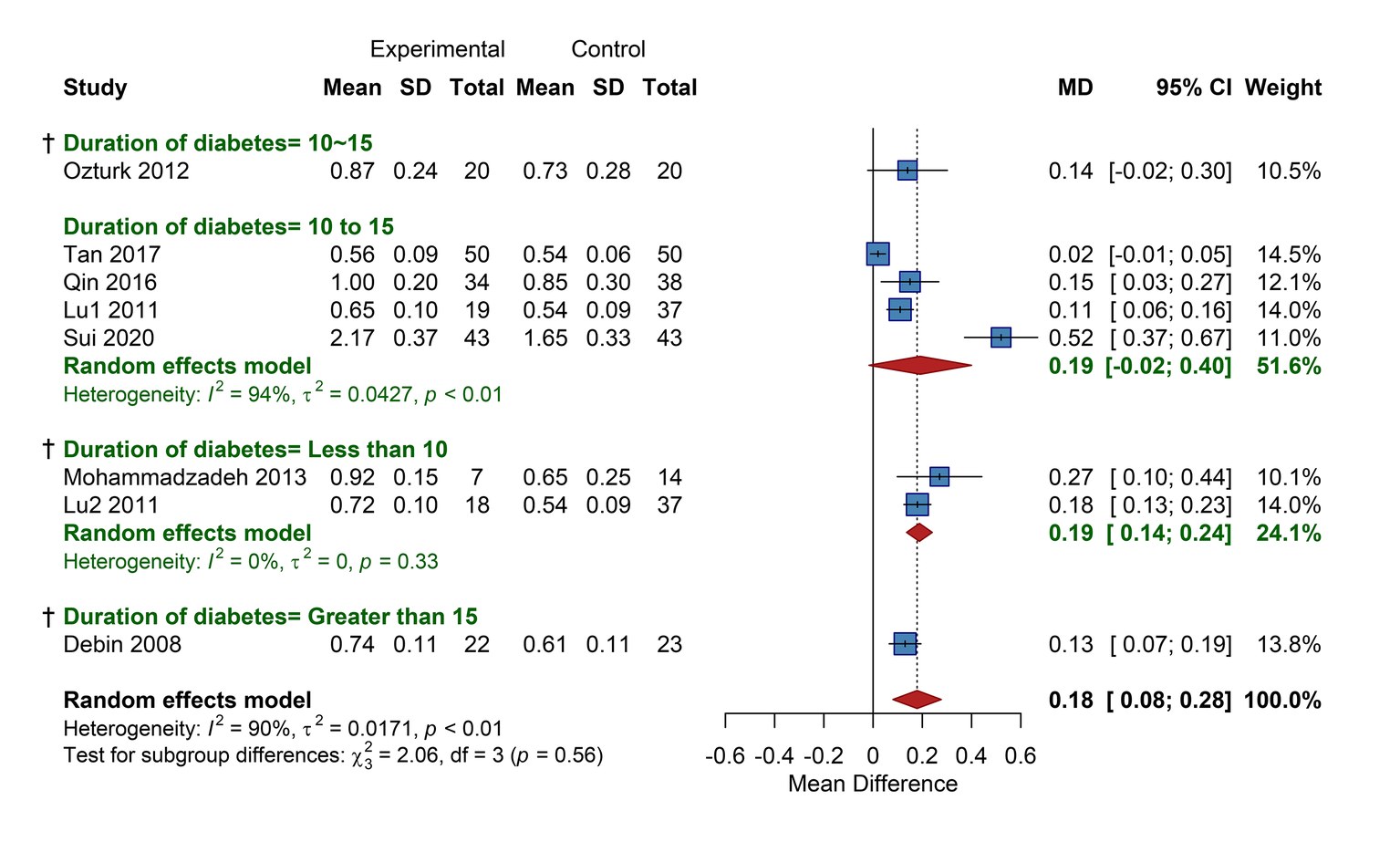


Fig S33 Subgroup Analysis of ABI by Duration of Diabetes, SD: Standard Deviation, † Results based on sparse data (number of studies k < 3) should be interpreted with caution.

## Fig S34


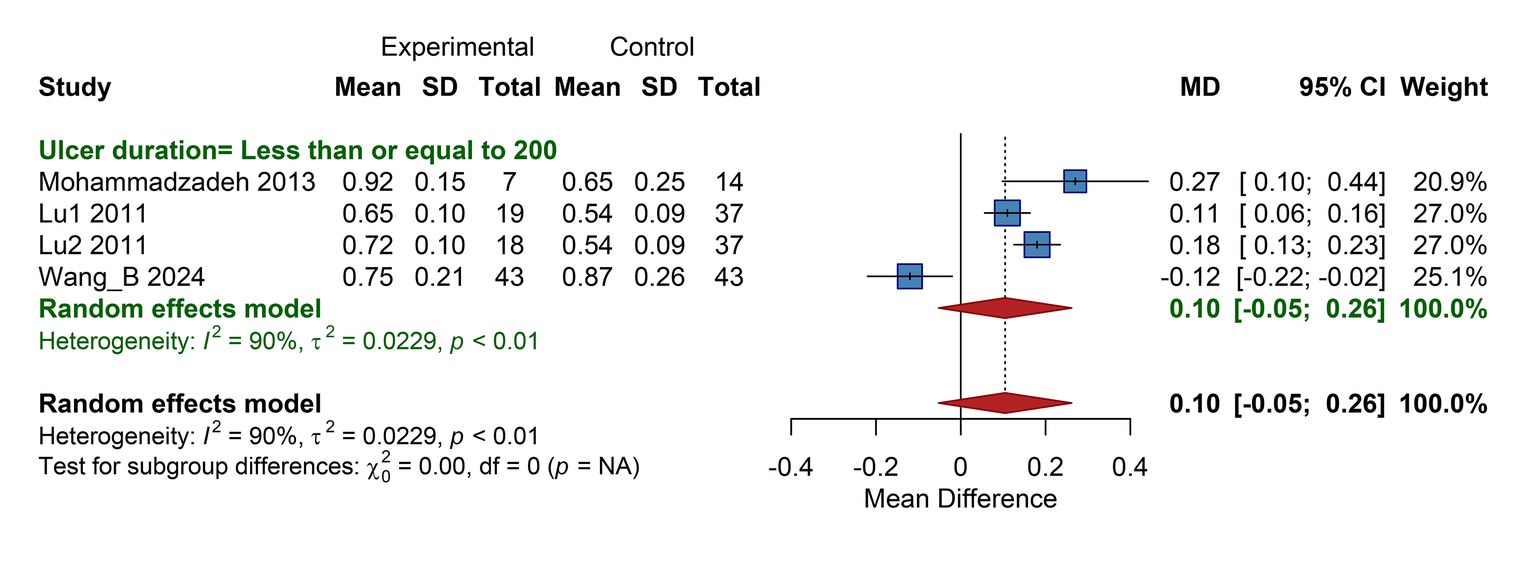


Fig S34 Subgroup Analysis of ABI by Ulcer Duration, SD: Standard Deviation, † Results based on sparse data (number of studies k < 3) should be interpreted with caution.

## Fig S35


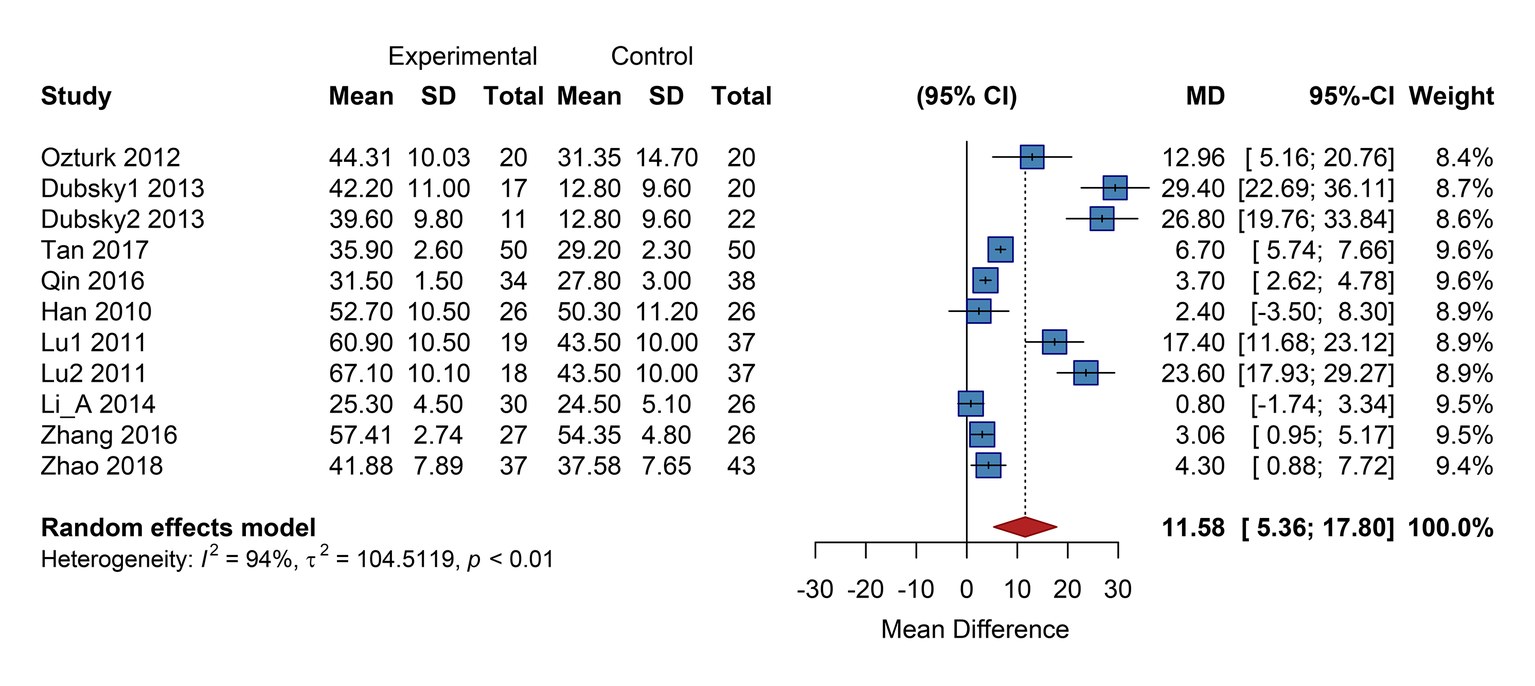


Fig S35 Overall TcPO₂ Forest Plot Analysis, SD: Standard Deviation

## Fig S36


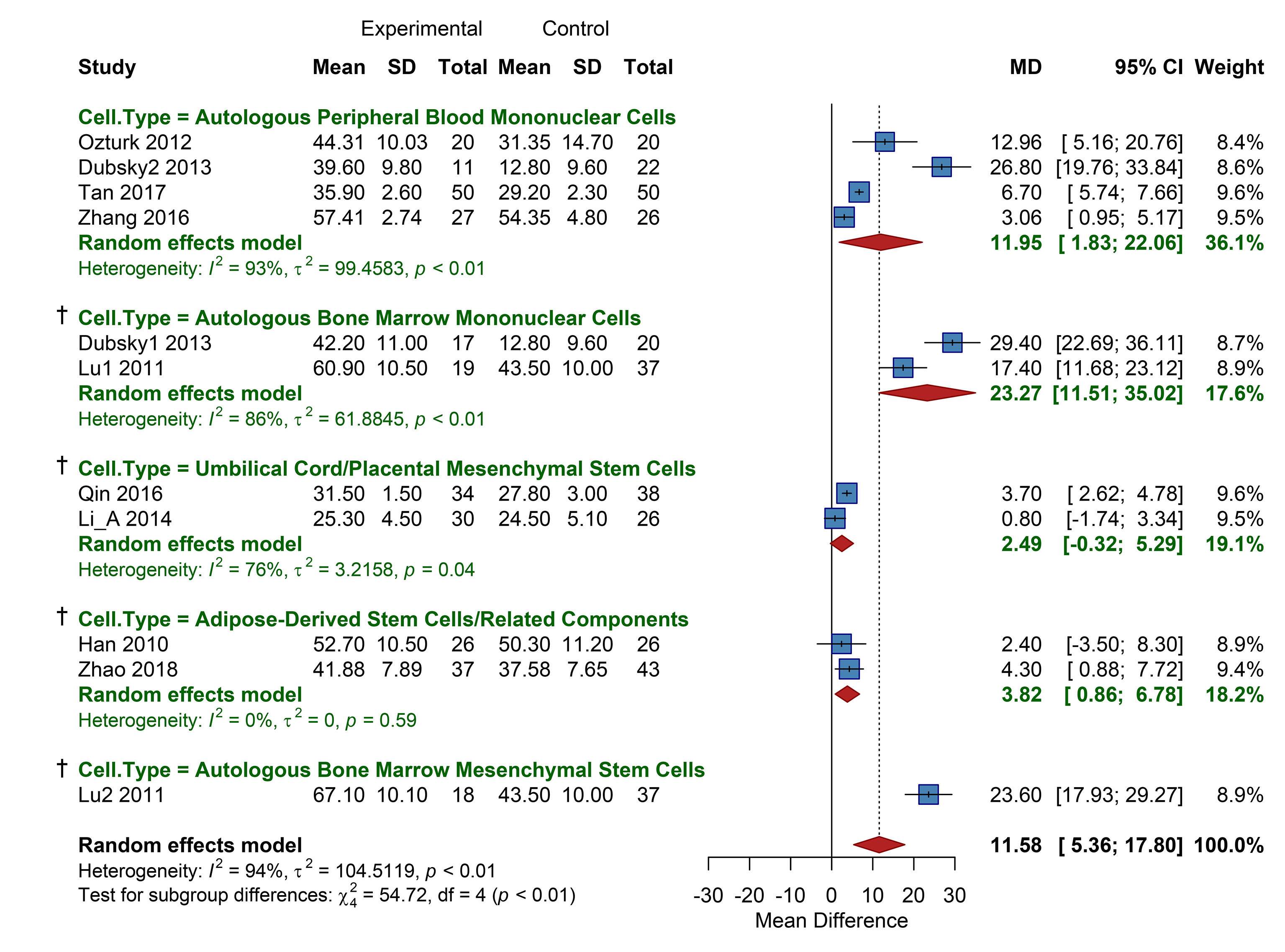


Fig S36 Subgroup Analysis of TcPO₂ by Cell Type, SD: Standard Deviation, † Results based on sparse data (number of studies k < 3) should be interpreted with caution.

## Fig S37


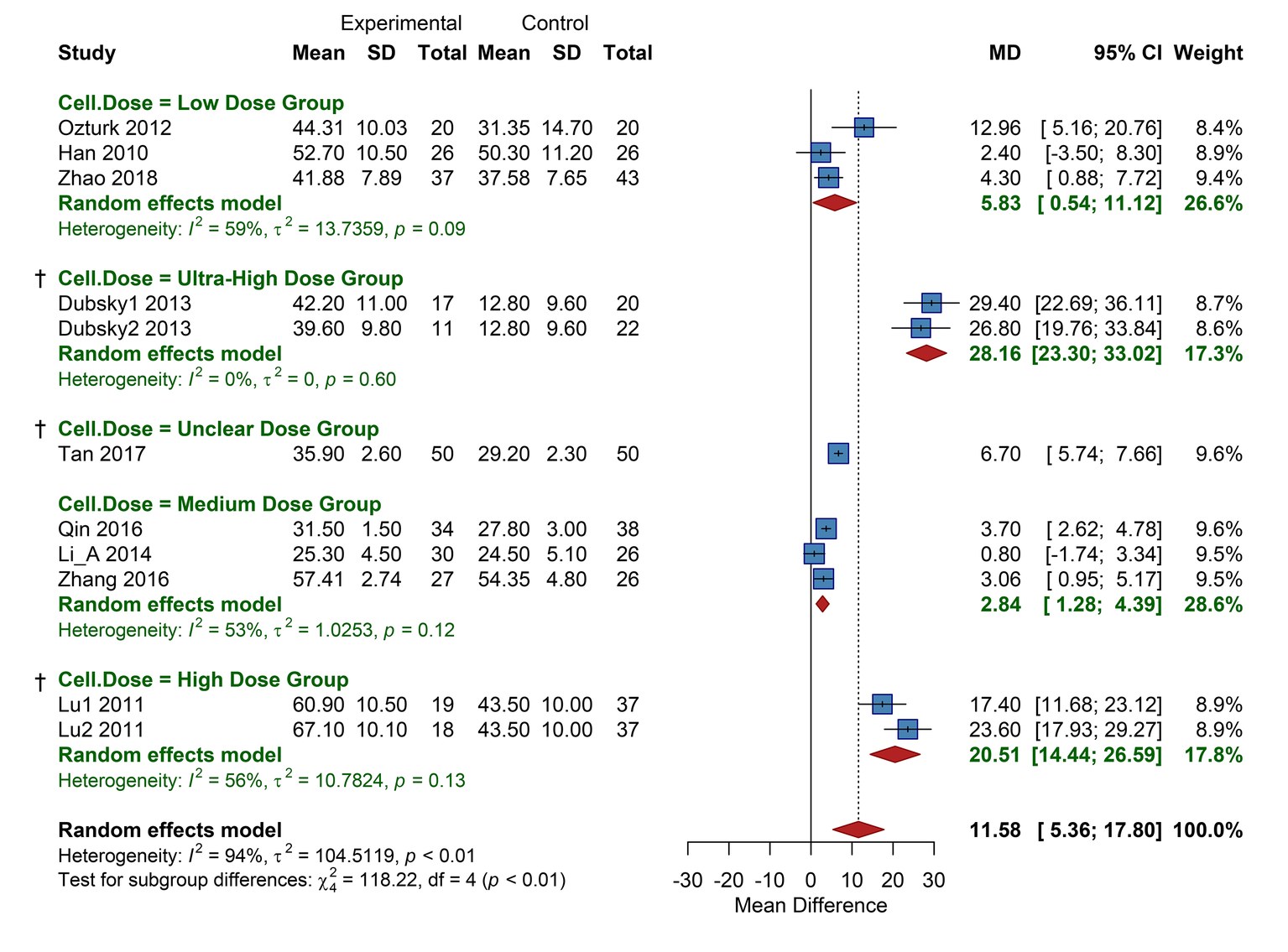


Fig S37 Subgroup Analysis of TcPO₂ by Cell Dose, SD: Standard Deviation, Low-dose group: 0.5×10⁶ to 8×10⁶ cells, Medium-dose group: 1×10⁷ to 8.6×10⁷ cells, High-dose group: 3×10⁸ to 1.2×10⁹ cells, Ultra-high-dose group: ≥2×10⁹ cells, unclear dose group: No quantifiable total cell count available, † Results based on sparse data (number of studies k < 3) should be interpreted with caution.

## Fig S38


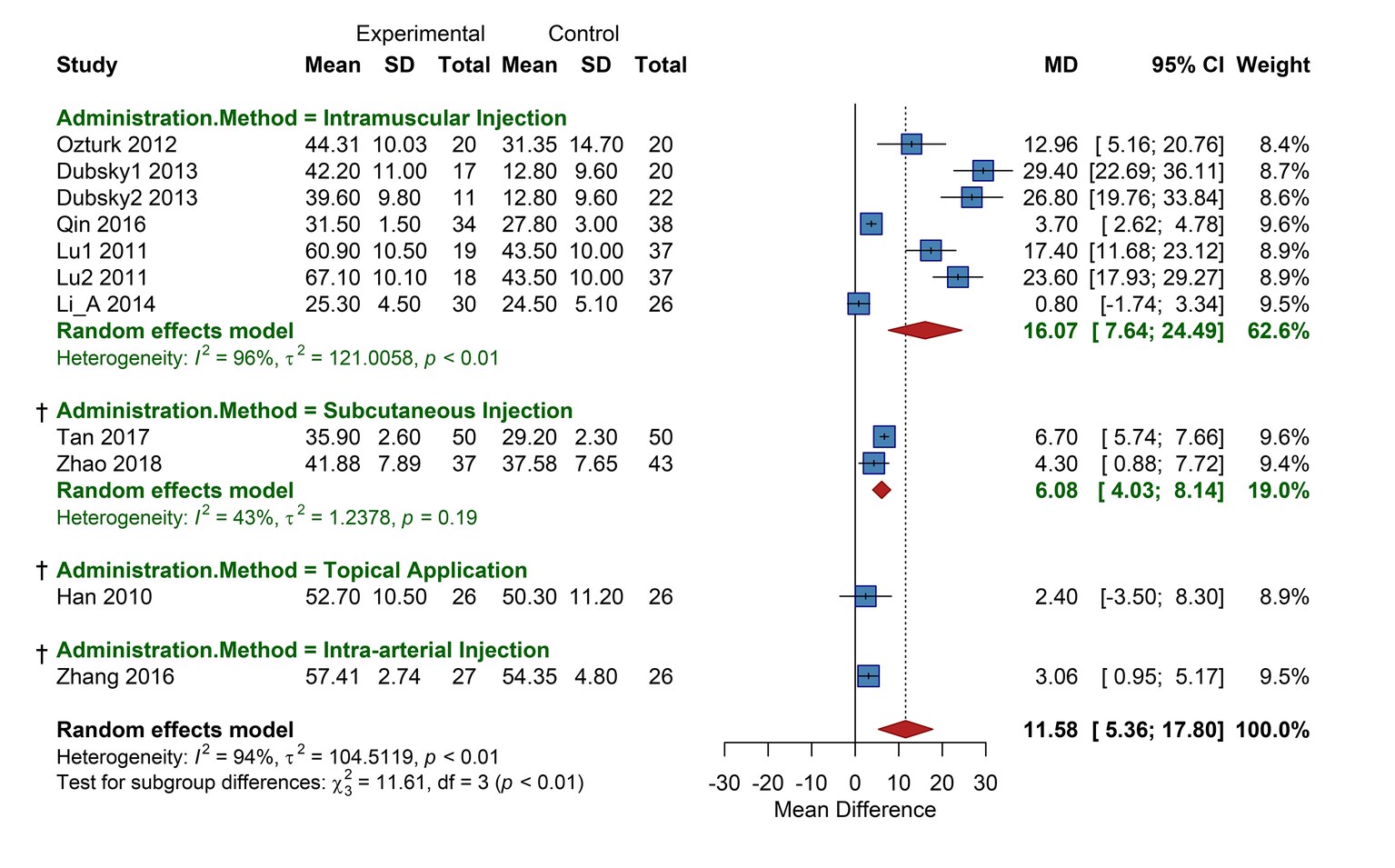


Fig S38 Subgroup Analysis of TcPO₂ by Administration Method, SD: Standard Deviation, † Results based on sparse data (number of studies k < 3) should be interpreted with caution.

## Fig S39


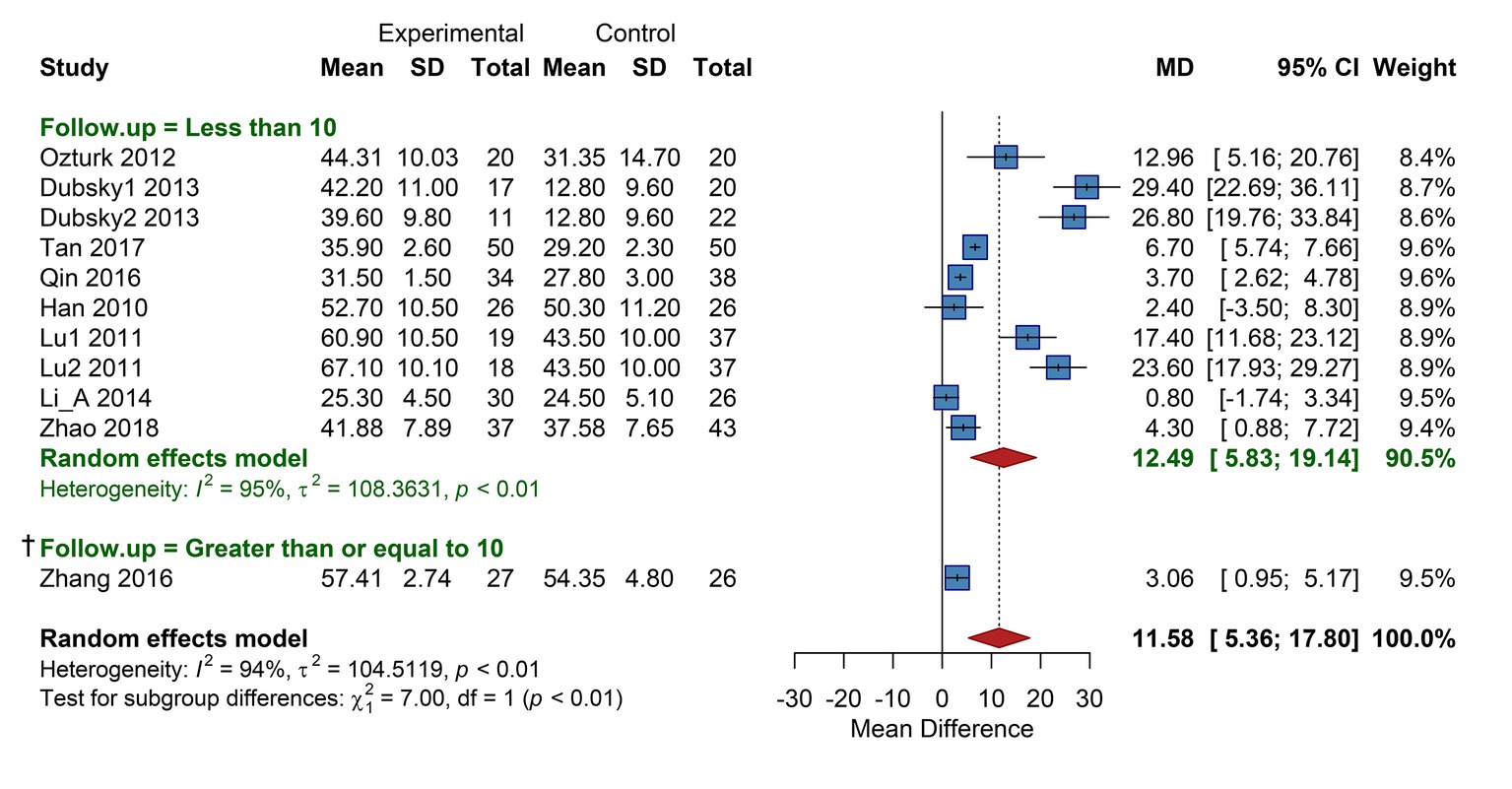


Fig S39 Subgroup Analysis of TcPO₂ by Follow-up Duration, SD: Standard Deviation, † Results based on sparse data (number of studies k < 3) should be interpreted with caution.

## Fig S40


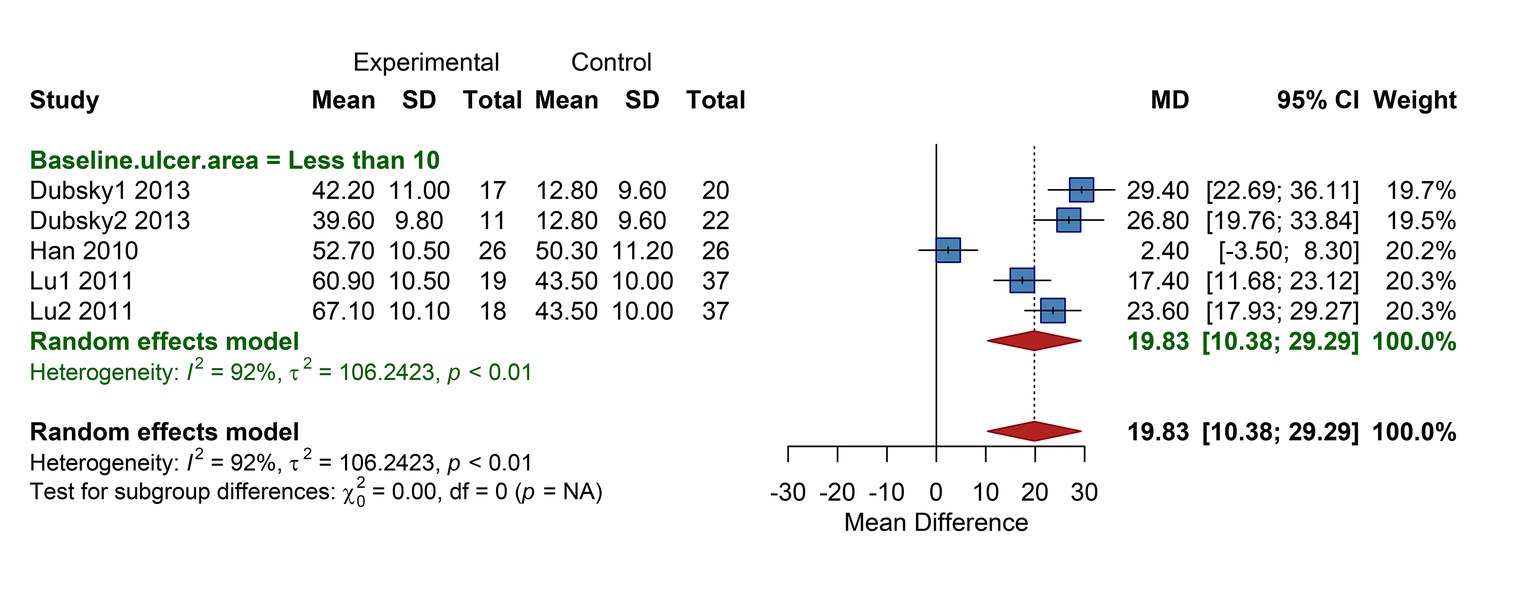


Fig S40 Subgroup Analysis of TcPO₂ by Baseline Ulcer Area, SD: Standard Deviation

## Fig S41


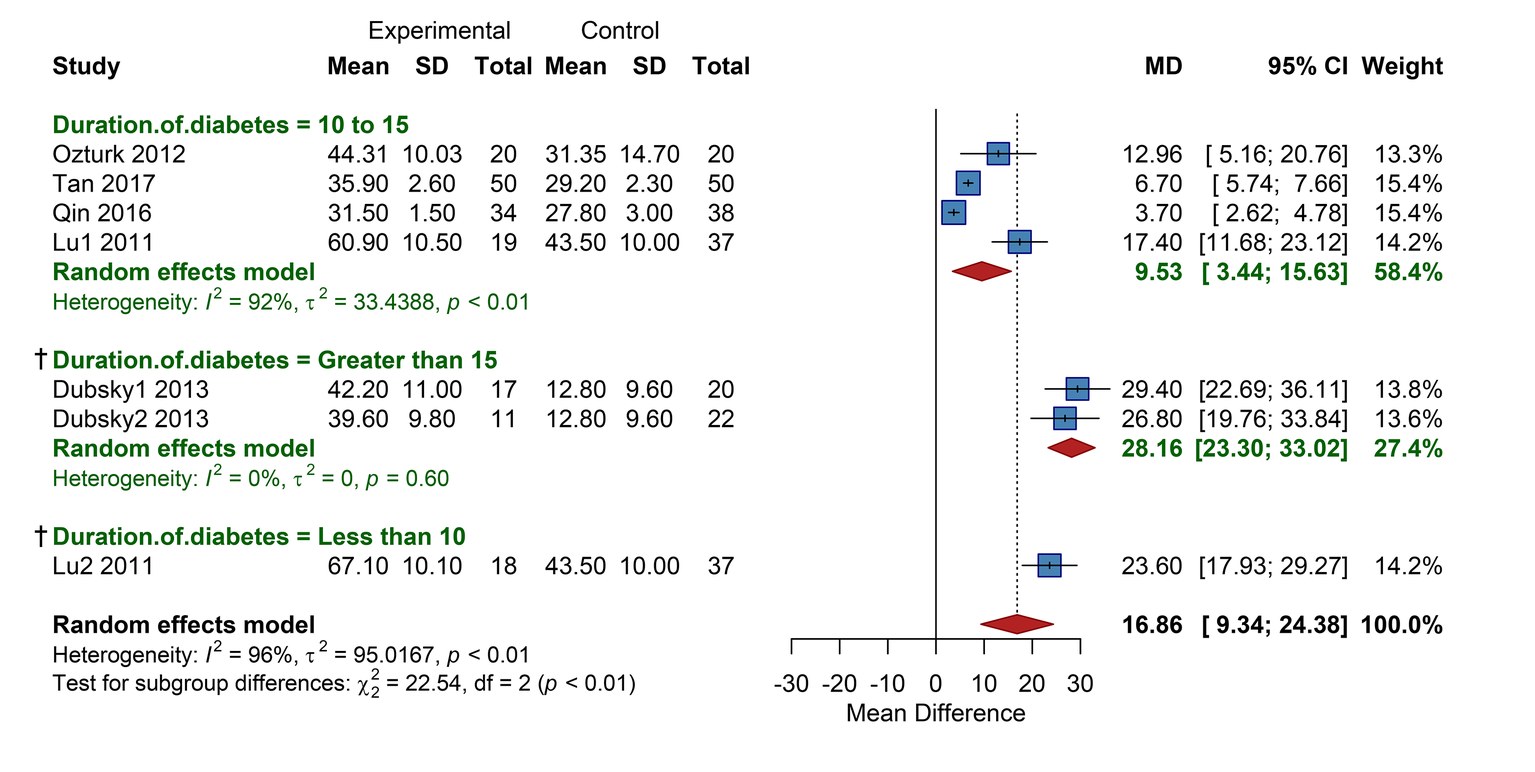


Fig S41 Subgroup Analysis of TcPO₂ by Duration of Diabetes, SD: Standard Deviation, † Results based on sparse data (number of studies k < 3) should be interpreted with caution.

## Fig S42


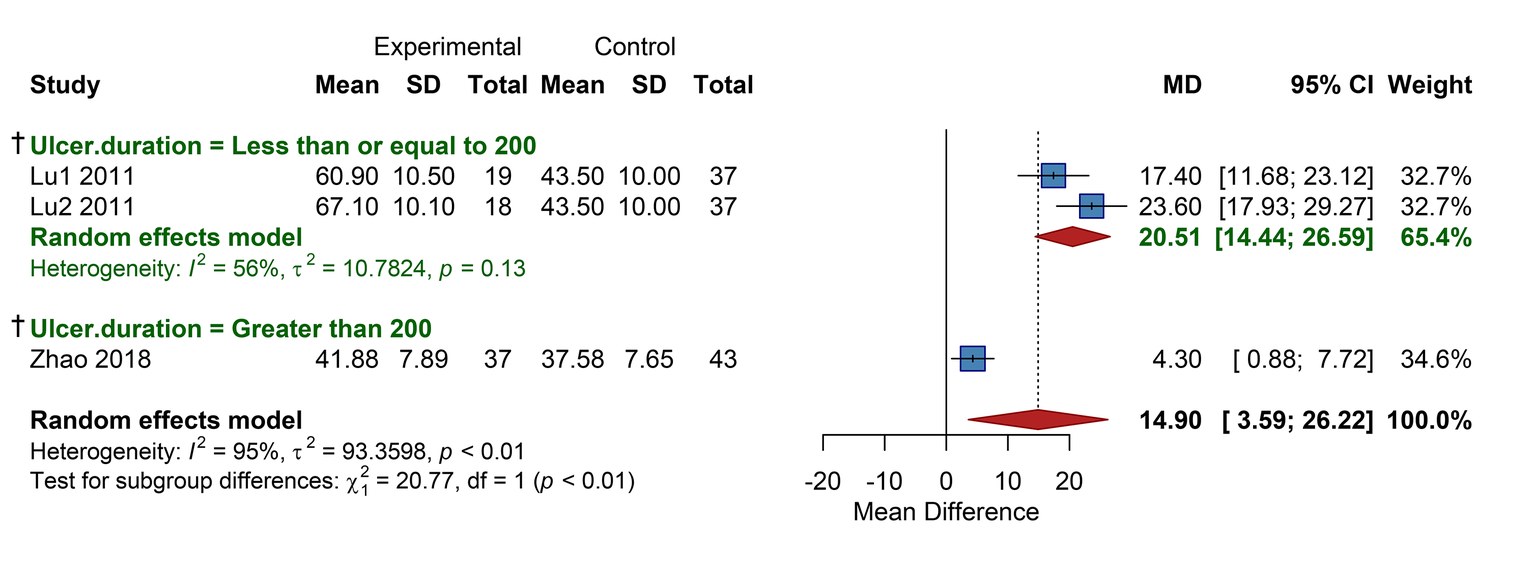


Fig S42 Subgroup Analysis of TcPO₂ by Ulcer Duration, SD: Standard Deviation, † Results based on sparse data (number of studies k < 3) should be interpreted with caution.

## Fig S43


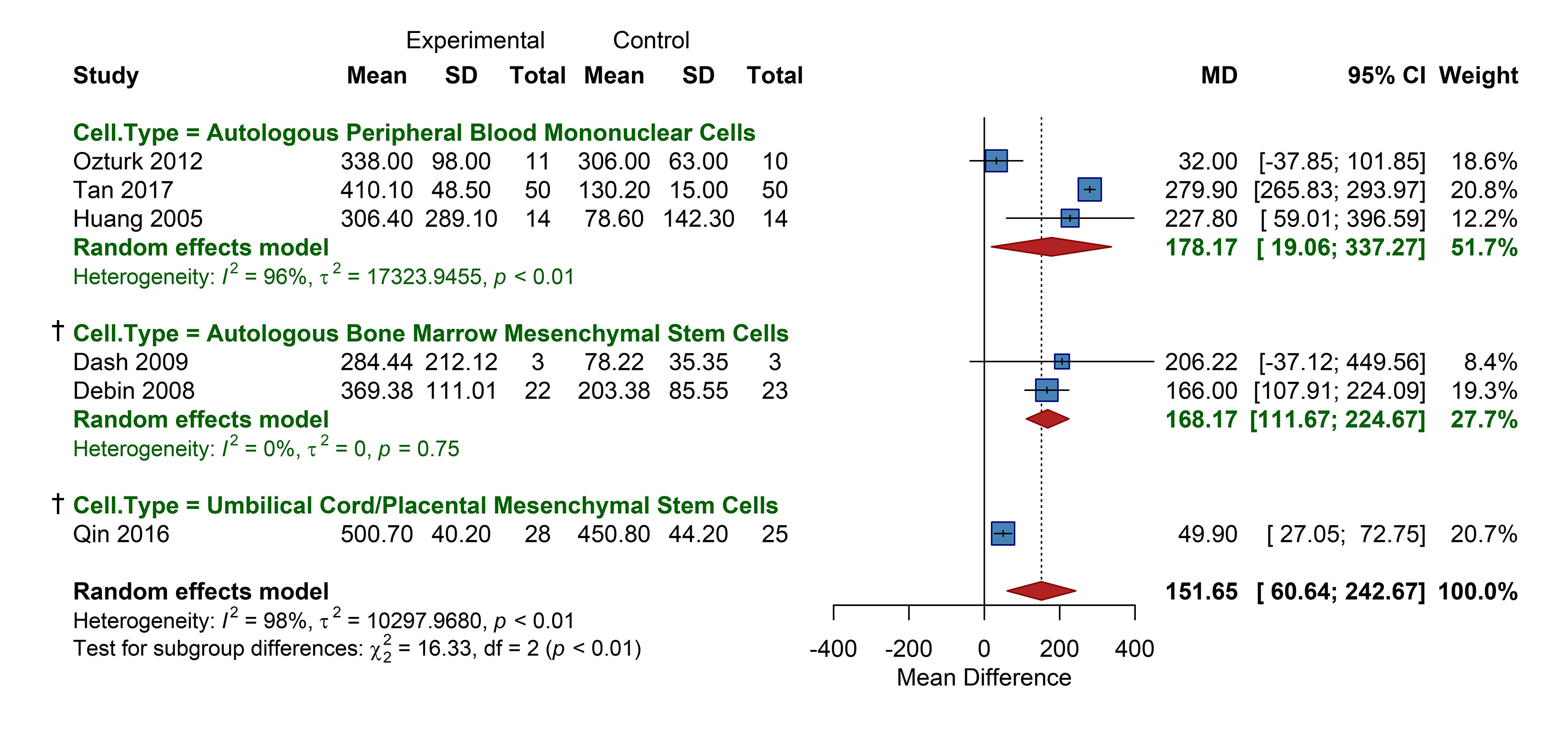


Fig S43 Subgroup Analysis of Pain-free Walking Distance by Cell Type, SD: Standard Deviation, † Results based on sparse data (number of studies k < 3) should be interpreted with caution.

## Fig S44


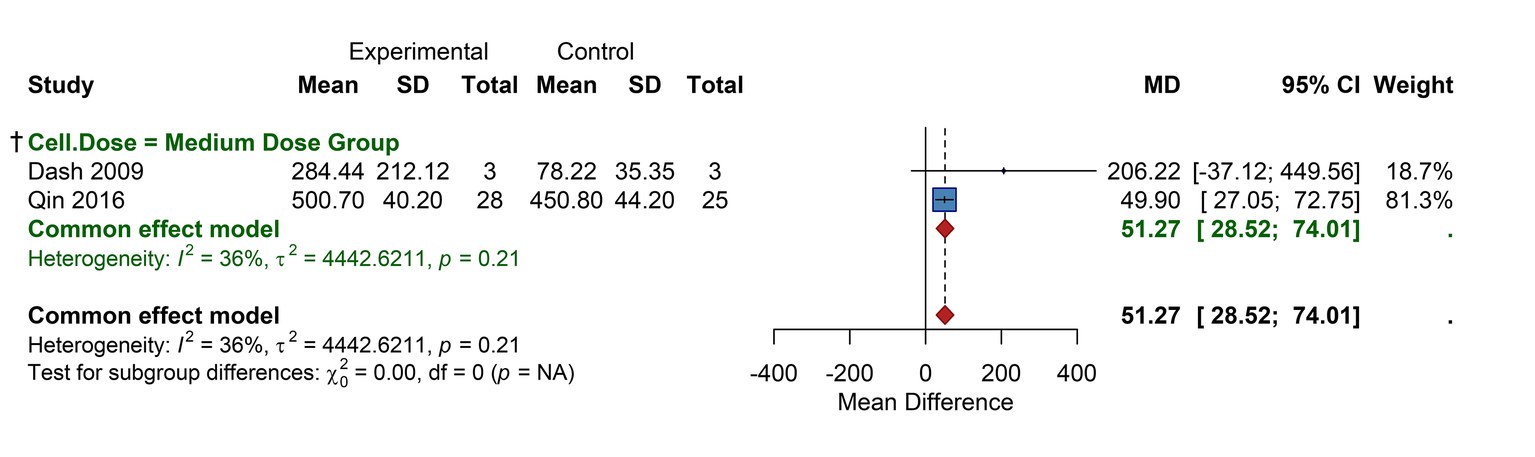


Fig S44 Subgroup Analysis of Pain-free Walking Distance by Cell Dose, SD: Standard Deviation, Low-dose group: 0.5×10⁶ to 8×10⁶ cells, Medium-dose group: 1×10⁷ to 8.6×10⁷ cells, High-dose group: 3×10⁸ to 1.2×10⁹ cells, Ultra-high-dose group: ≥2×10⁹ cells, unclear dose group: No quantifiable total cell count available, † Results based on sparse data (number of studies k < 3) should be interpreted with caution.

## Fig S45


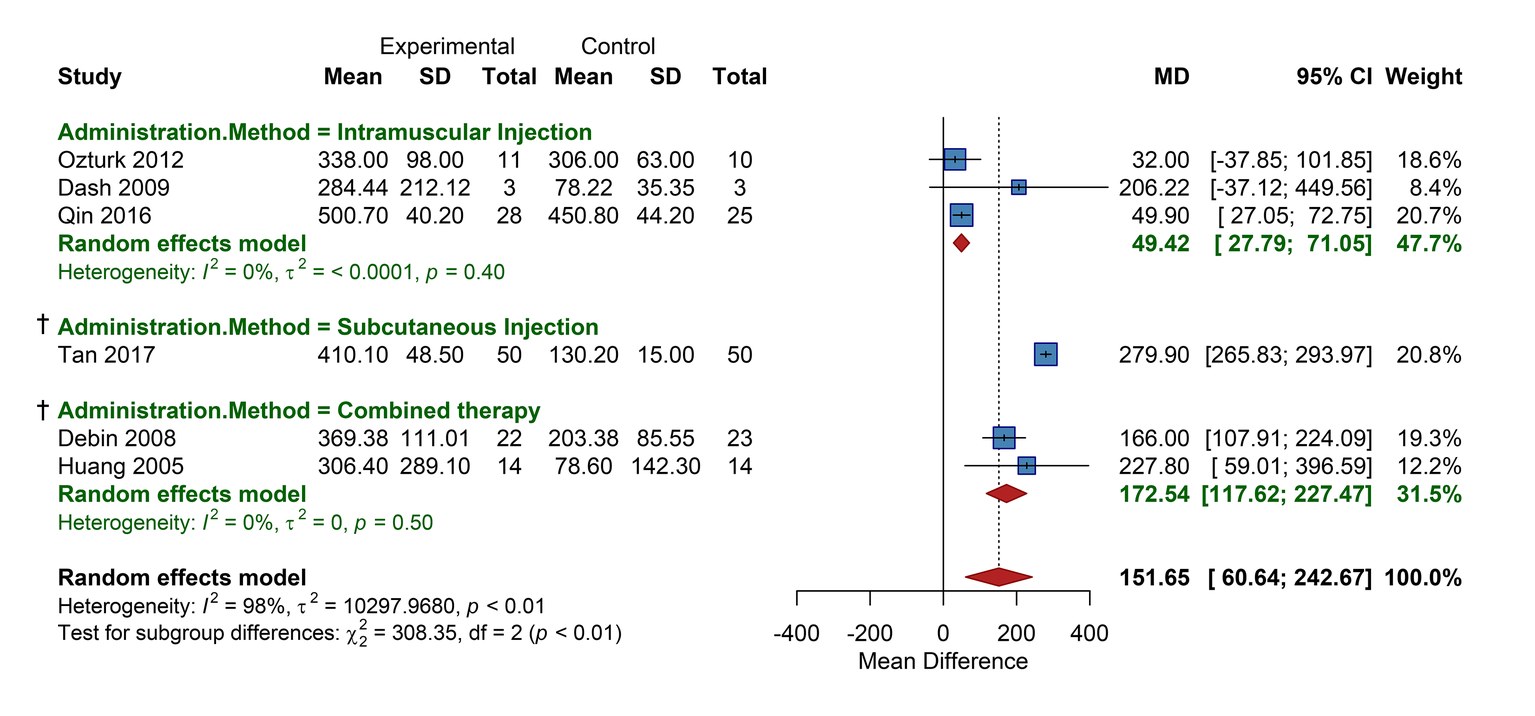


Fig S45 Subgroup Analysis of Pain-free Walking Distance by Administration Method, SD: Standard Deviation, † Results based on sparse data (number of studies k < 3) should be interpreted with caution.

## Fig S46


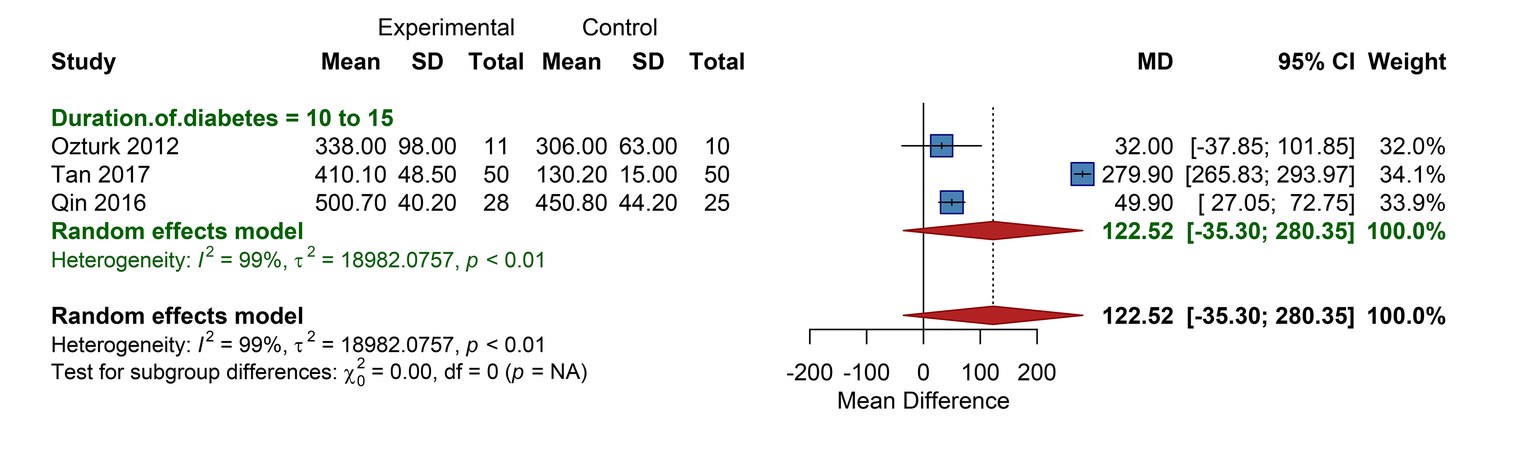


Fig S46 Subgroup Analysis of Pain-free Walking Distance by Duration of Diabetes, SD: Standard Deviation

## Fig S47


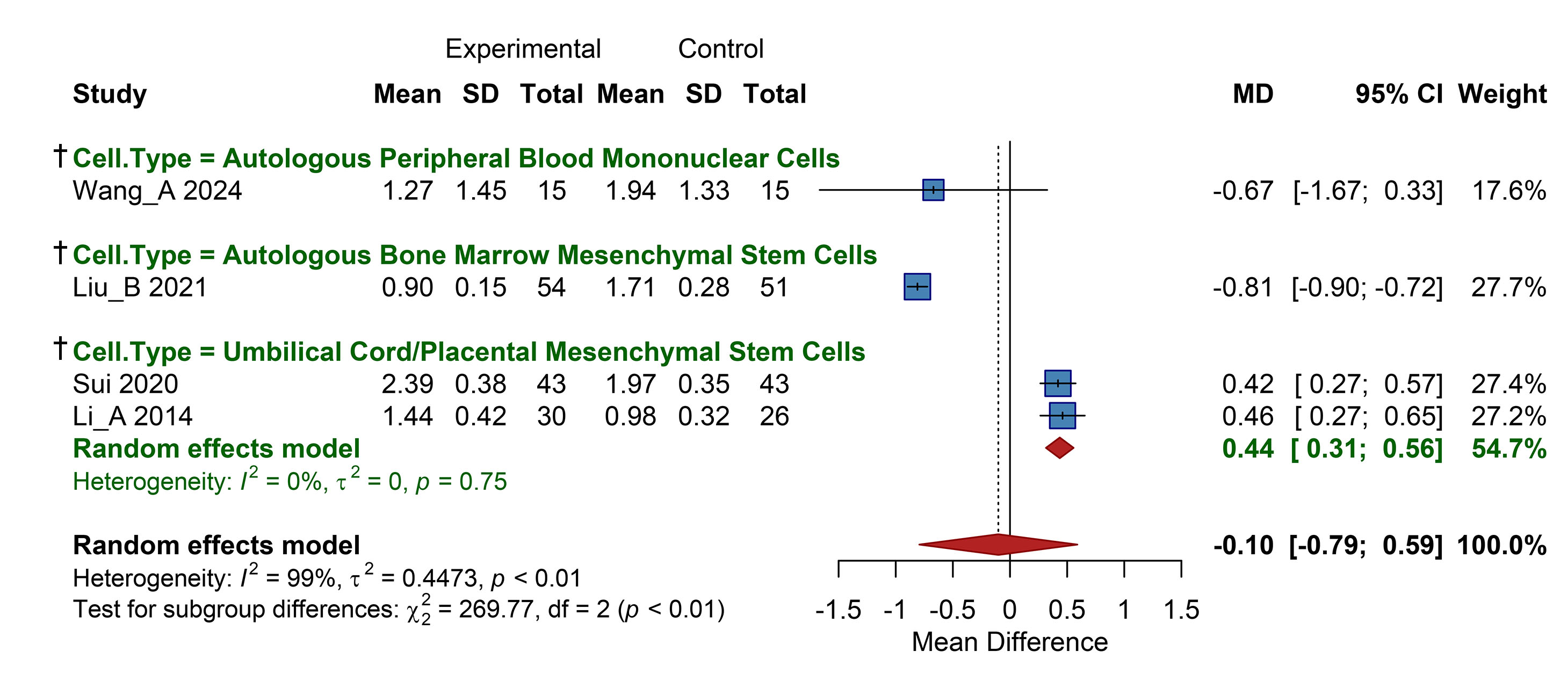


Fig S47 Subgroup Analysis of Pain-free Walking Claudication Score by Cell Type, SD: Standard Deviation, † Results based on sparse data (number of studies k < 3) should be interpreted with caution.

## Fig S48


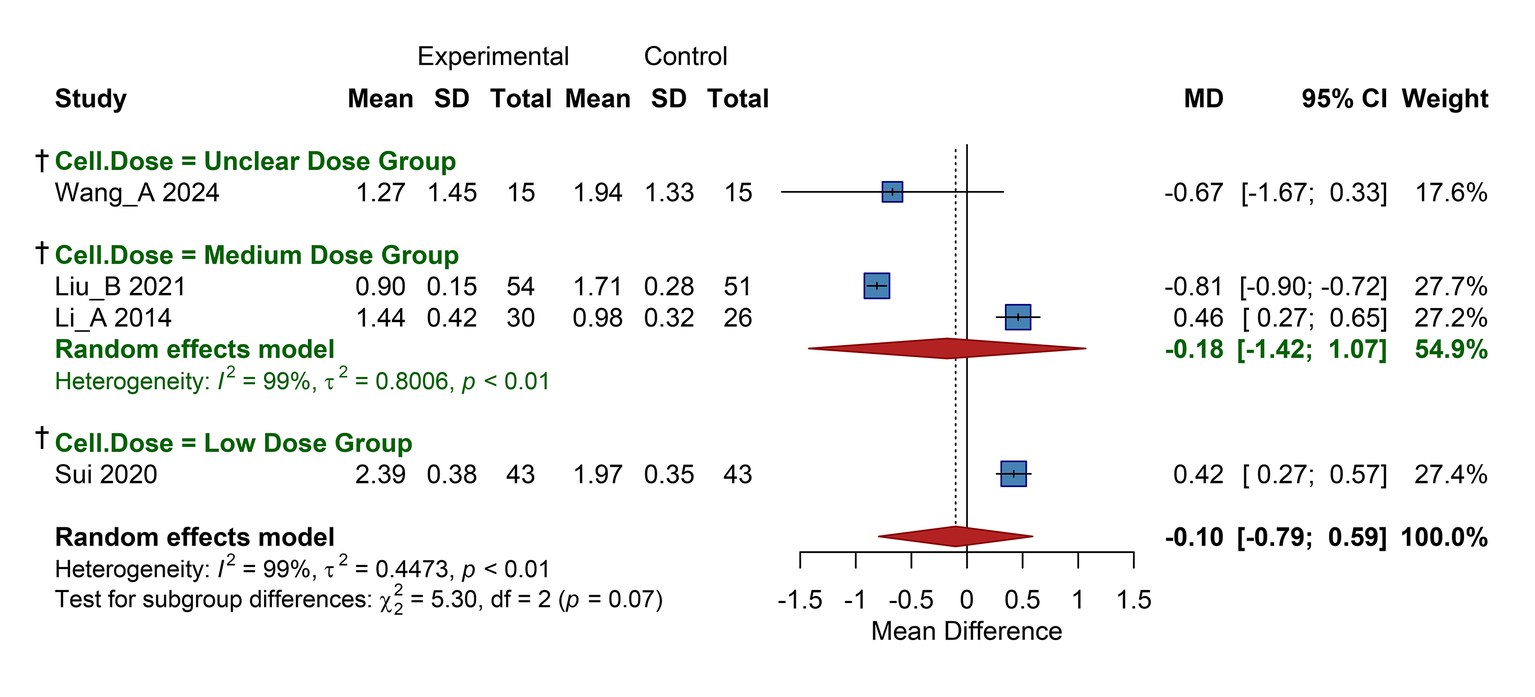


Fig S48 Subgroup Analysis of Pain-free Walking Claudication Score by Cell Dose, SD: Standard Deviation, Low-dose group: 0.5×10⁶ to 8×10⁶ cells, Medium-dose group: 1×10⁷ to 8.6×10⁷ cells, High-dose group: 3×10⁸ to 1.2×10⁹ cells, Ultra-high-dose group: ≥2×10⁹ cells, unclear dose group: No quantifiable total cell count available, † Results based on sparse data (number of studies k < 3) should be interpreted with caution.

## Fig S49


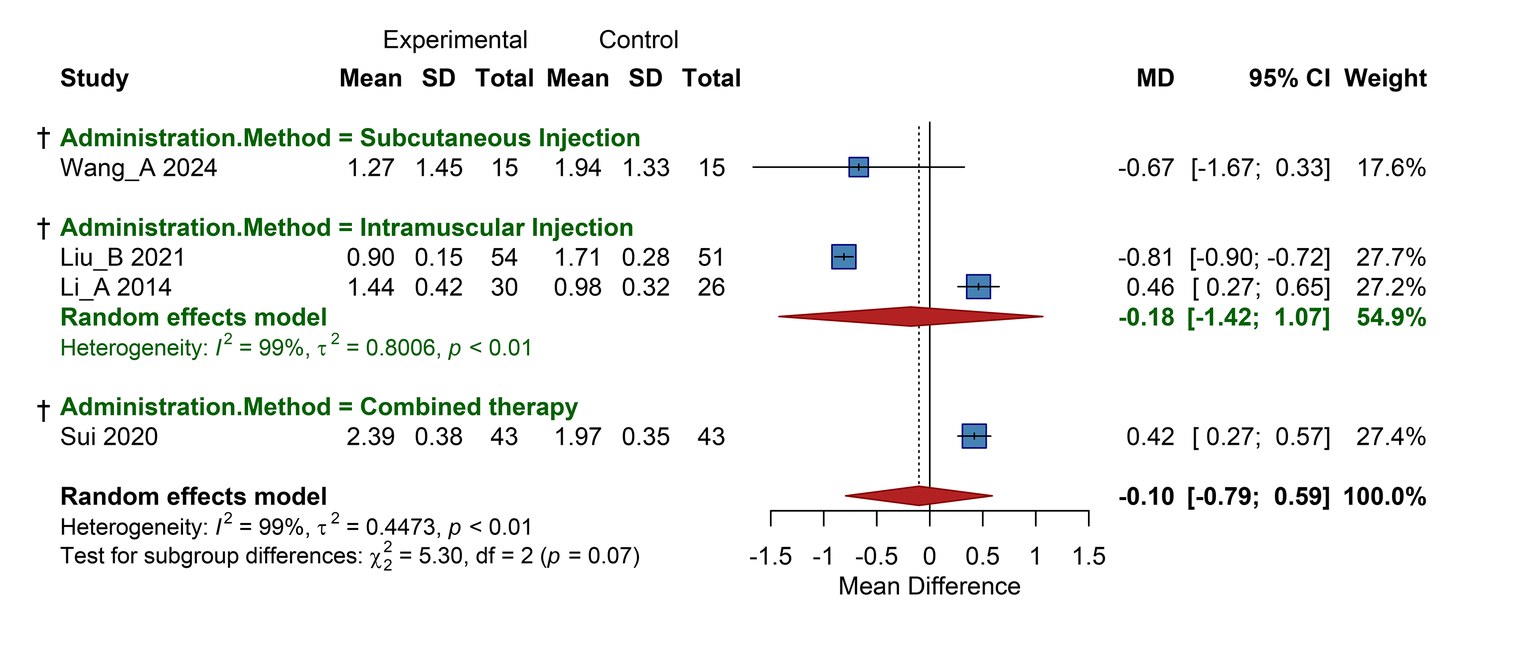


Fig S49 Subgroup Analysis of Pain-free Walking Claudication Score by Administration Method, SD: Standard Deviation, † Results based on sparse data (number of studies k < 3) should be interpreted with caution.

## Fig S50


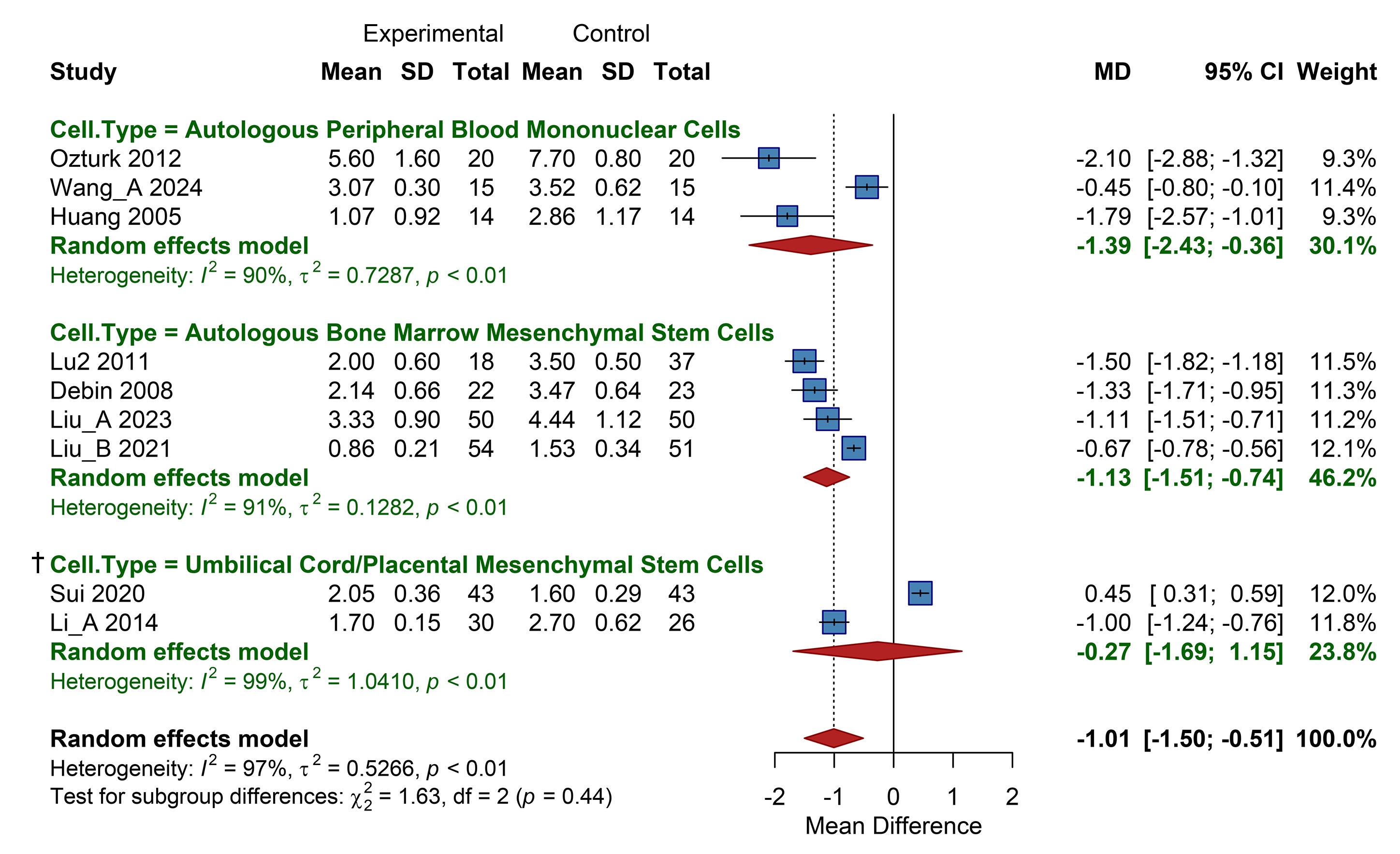


Fig S50 Subgroup Analysis of Resting Pain Score by Cell Type, SD: Standard Deviation, † Results based on sparse data (number of studies k < 3) should be interpreted with caution.

## Fig S51


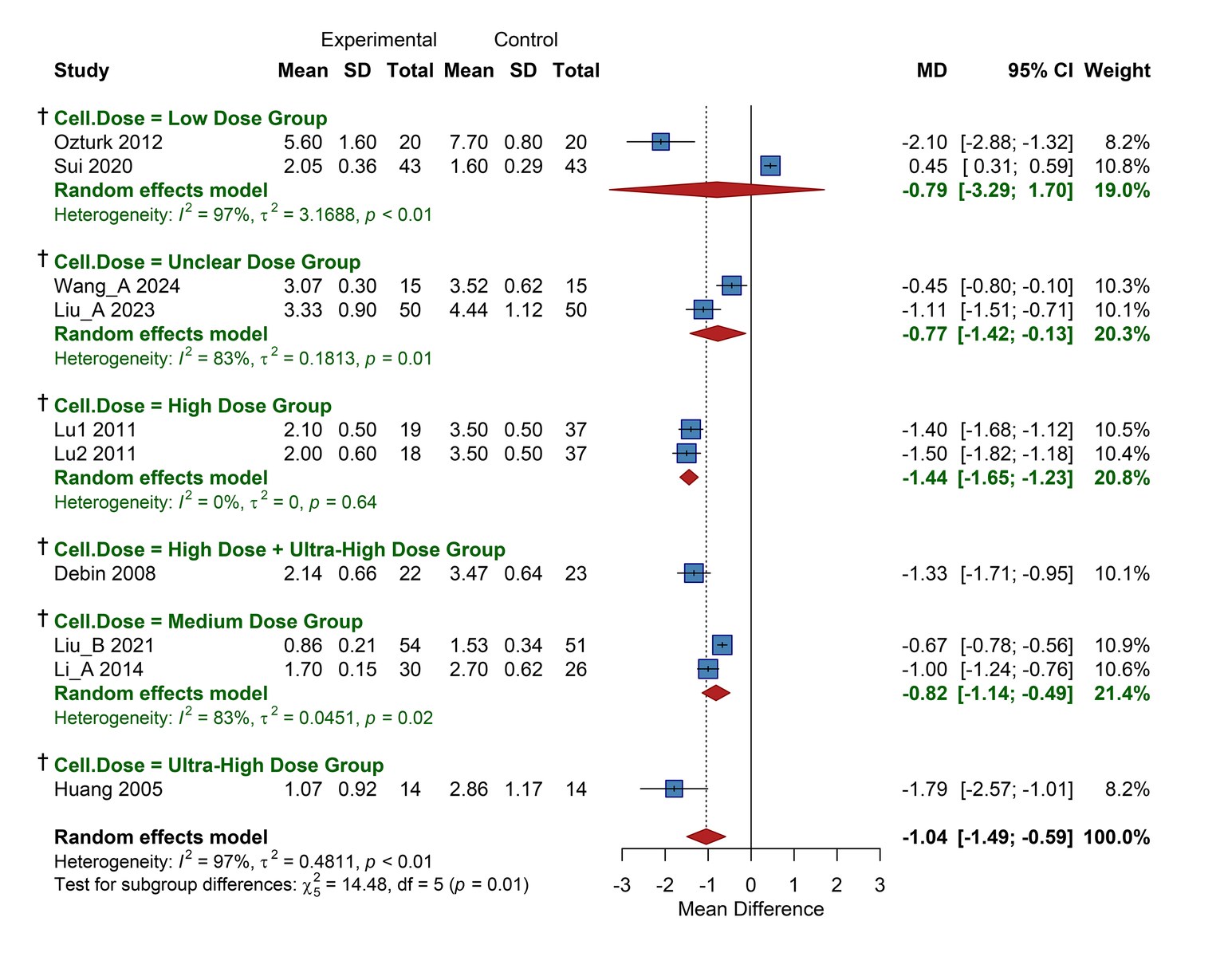


Fig S51 Subgroup Analysis of Resting Pain Score by Cell Dose, SD: Standard Deviation, Low-dose group: 0.5×10⁶ to 8×10⁶ cells, Medium-dose group: 1×10⁷ to 8.6×10⁷ cells, High-dose group: 3×10⁸ to 1.2×10⁹ cells, Ultra-high-dose group: ≥2×10⁹ cells, unclear dose group: No quantifiable total cell count available, † Results based on sparse data (number of studies k < 3) should be interpreted with caution.

## Fig S52


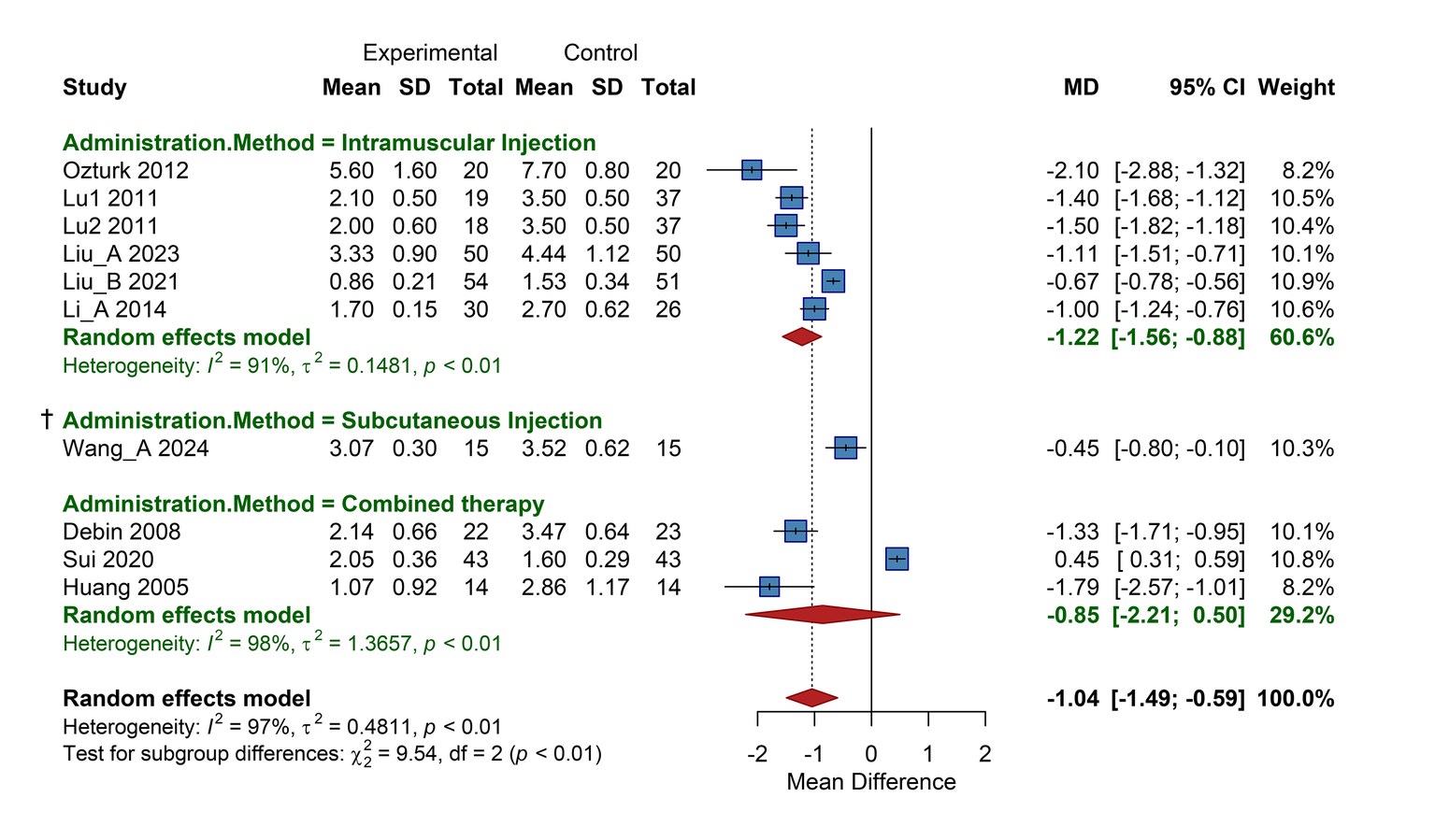


Fig S52 Subgroup Analysis of Resting Pain Score by Administration Method, SD: Standard Deviation, † Results based on sparse data (number of studies k < 3) should be interpreted with caution.

## Fig S53


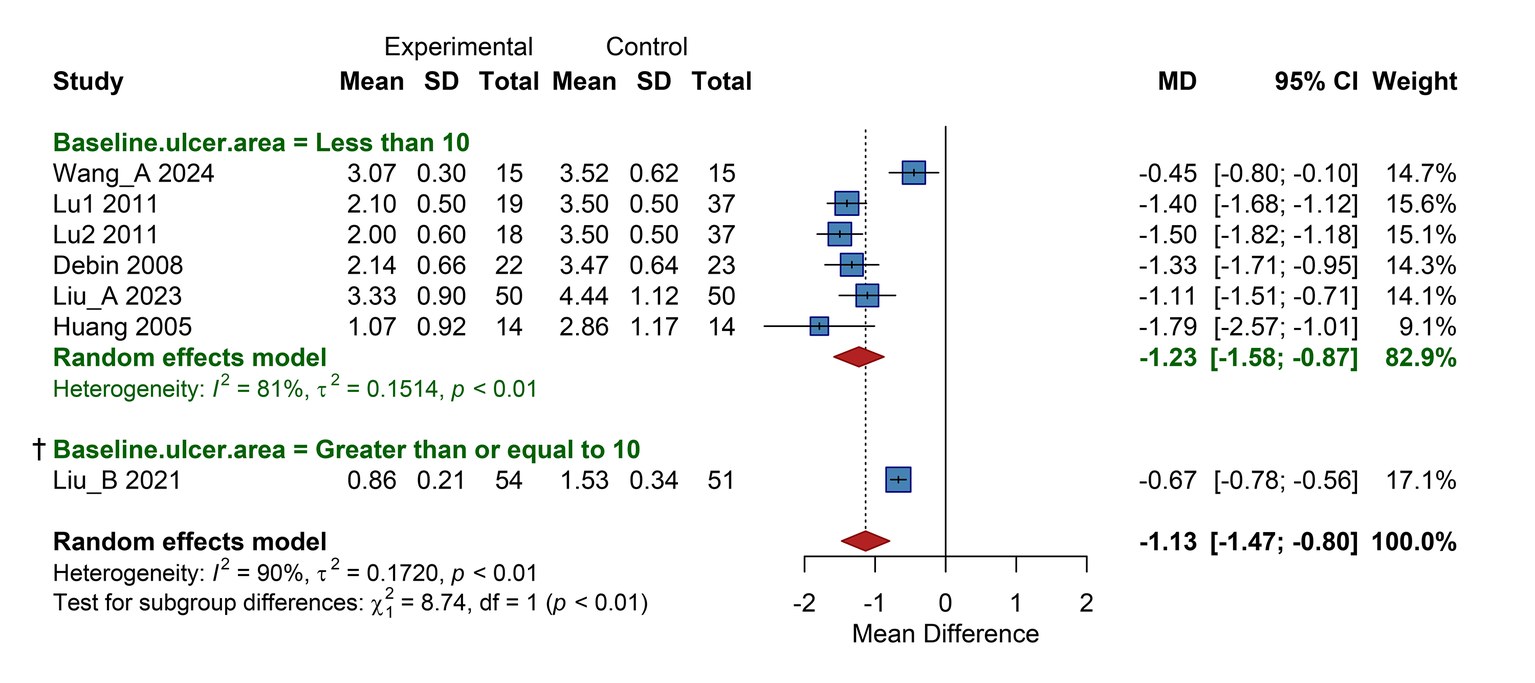


Fig S53 Subgroup Analysis of Resting Pain Score by Baseline Ulcer Area, SD: Standard Deviation, † Results based on sparse data (number of studies k < 3) should be interpreted with caution.

## Fig S54


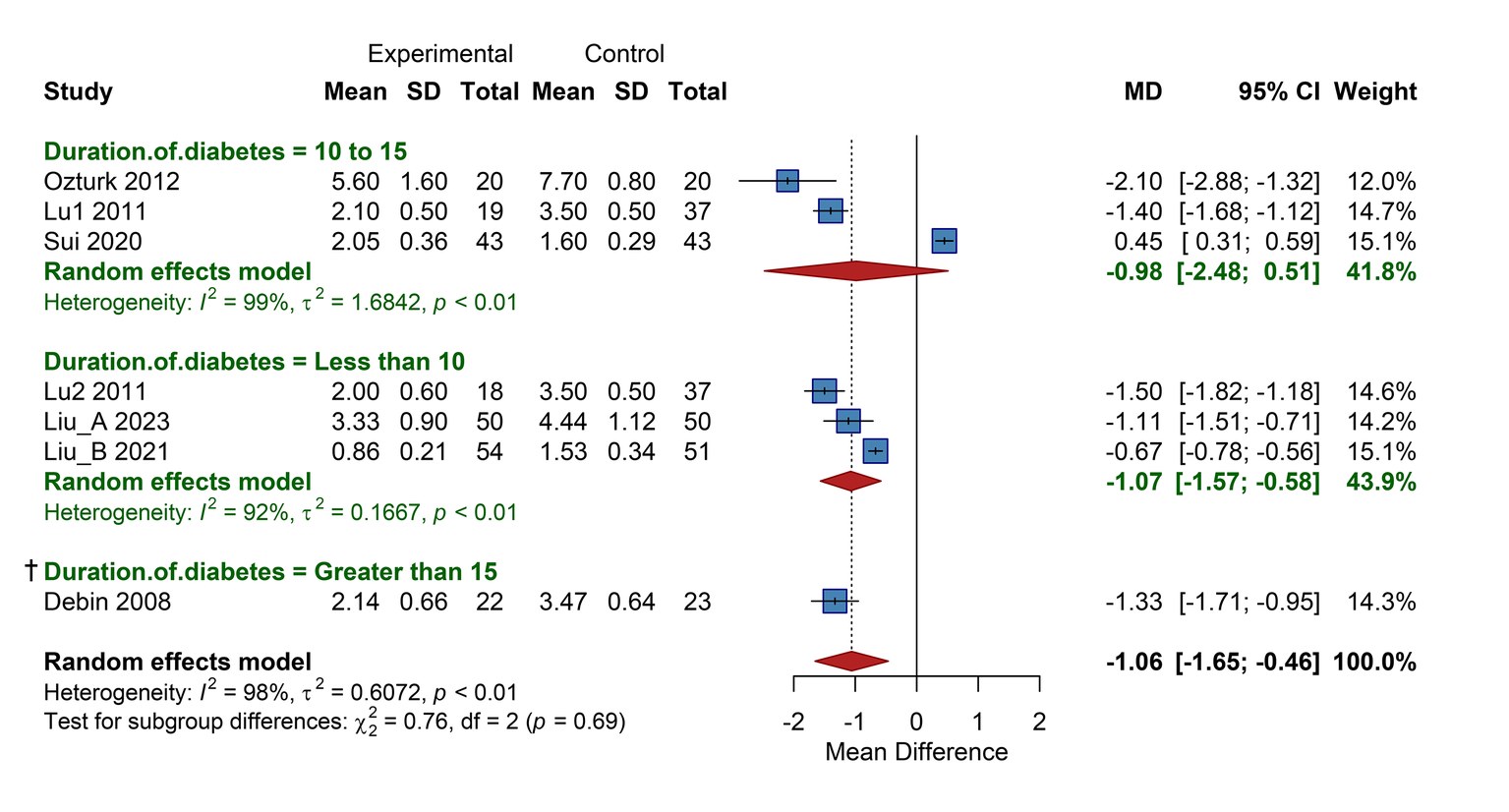


Fig S54 Subgroup Analysis of Resting Pain Score by Duration of Diabetes, SD: Standard Deviation, † Results based on sparse data (number of studies k < 3) should be interpreted with caution.

## Fig S55


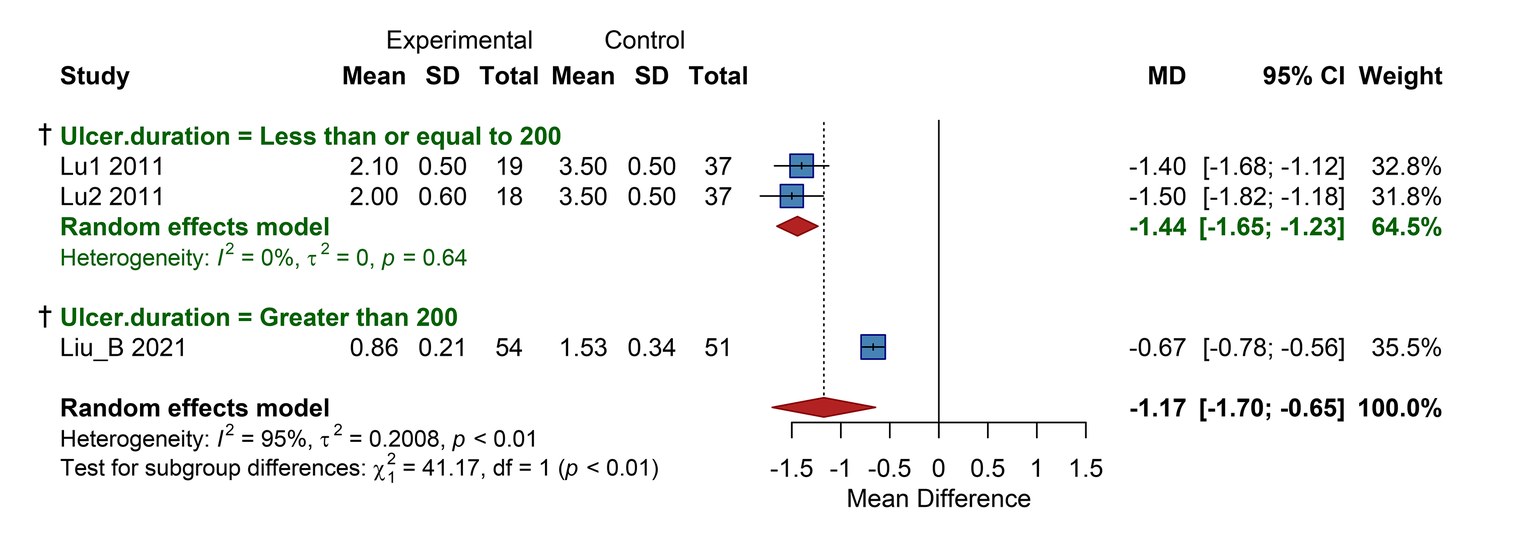


Fig S55 Subgroup Analysis of Resting Pain Score by Ulcer Duration, SD: Standard Deviation, † Results based on sparse data (number of studies k < 3) should be interpreted with caution.

## Fig S56


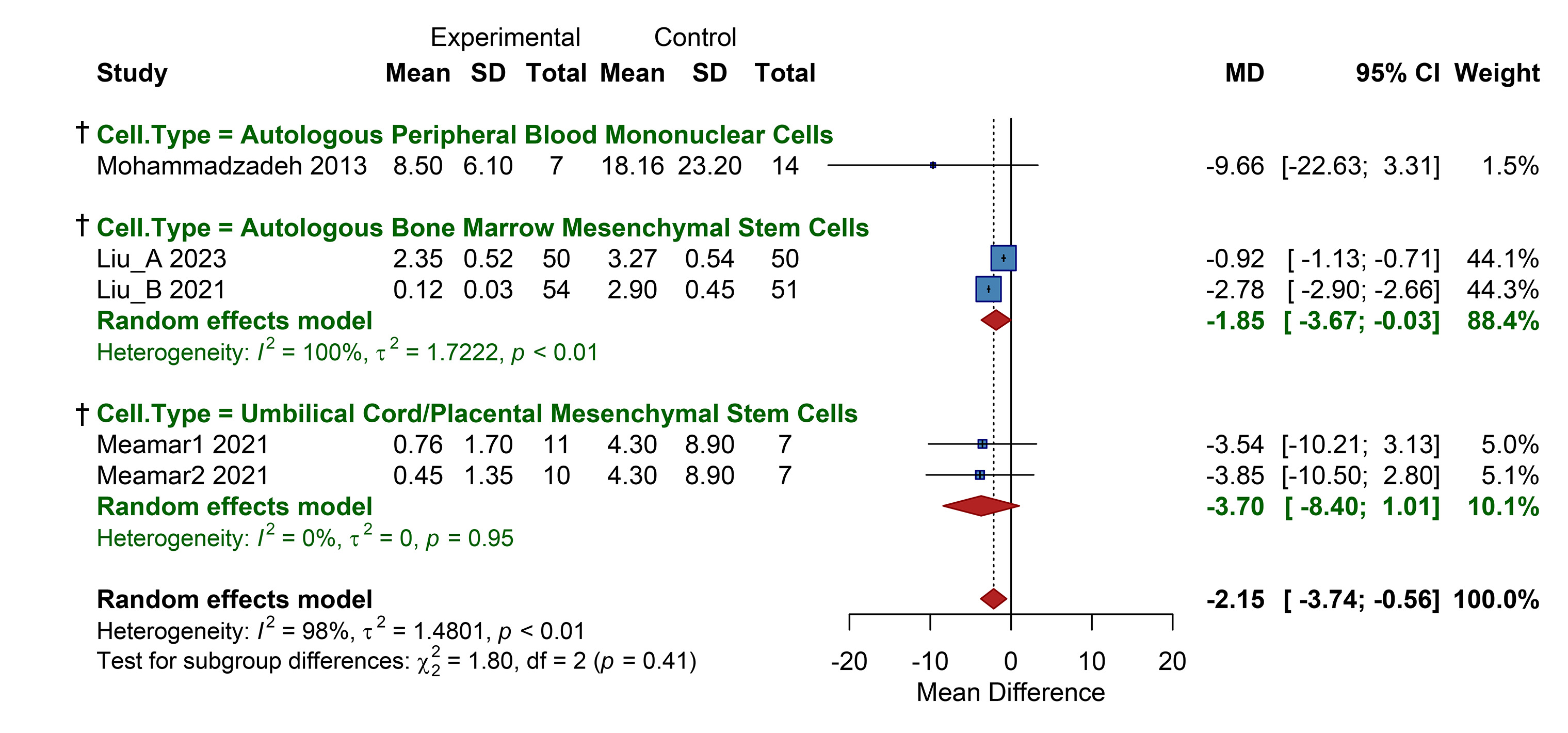


Fig S56 Subgroup Analysis of Ulcer Area by Cell Type, SD: Standard Deviation, † Results based on sparse data (number of studies k < 3) should be interpreted with caution.

## Fig S57


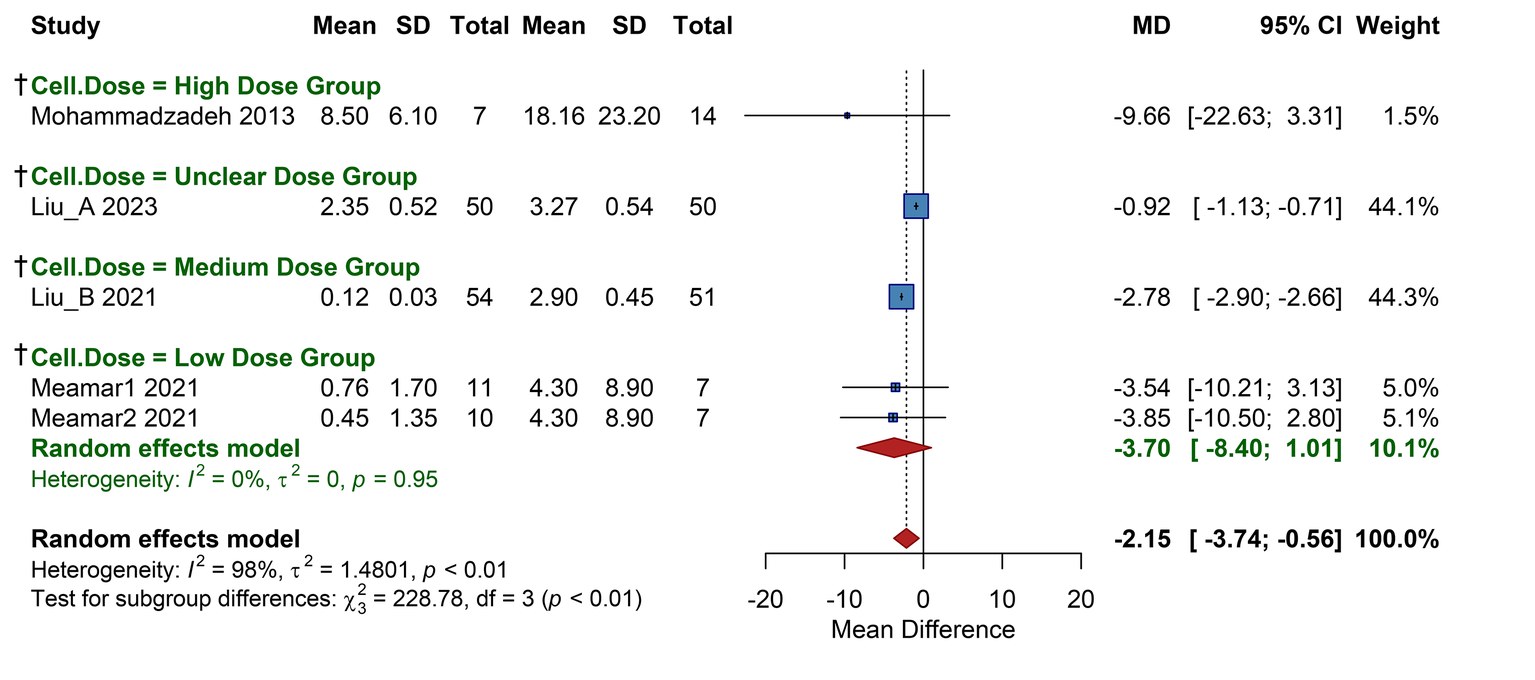


Fig S57 Subgroup Analysis of Ulcer Area by Cell Dose, SD: Standard Deviation, Low-dose group: 0.5×10⁶ to 8×10⁶ cells, Medium-dose group: 1×10⁷ to 8.6×10⁷ cells, High-dose group: 3×10⁸ to 1.2×10⁹ cells, Ultra-high-dose group: ≥2×10⁹ cells, unclear dose group: No quantifiable total cell count available, † Results based on sparse data (number of studies k < 3) should be interpreted with caution.

## Fig S58


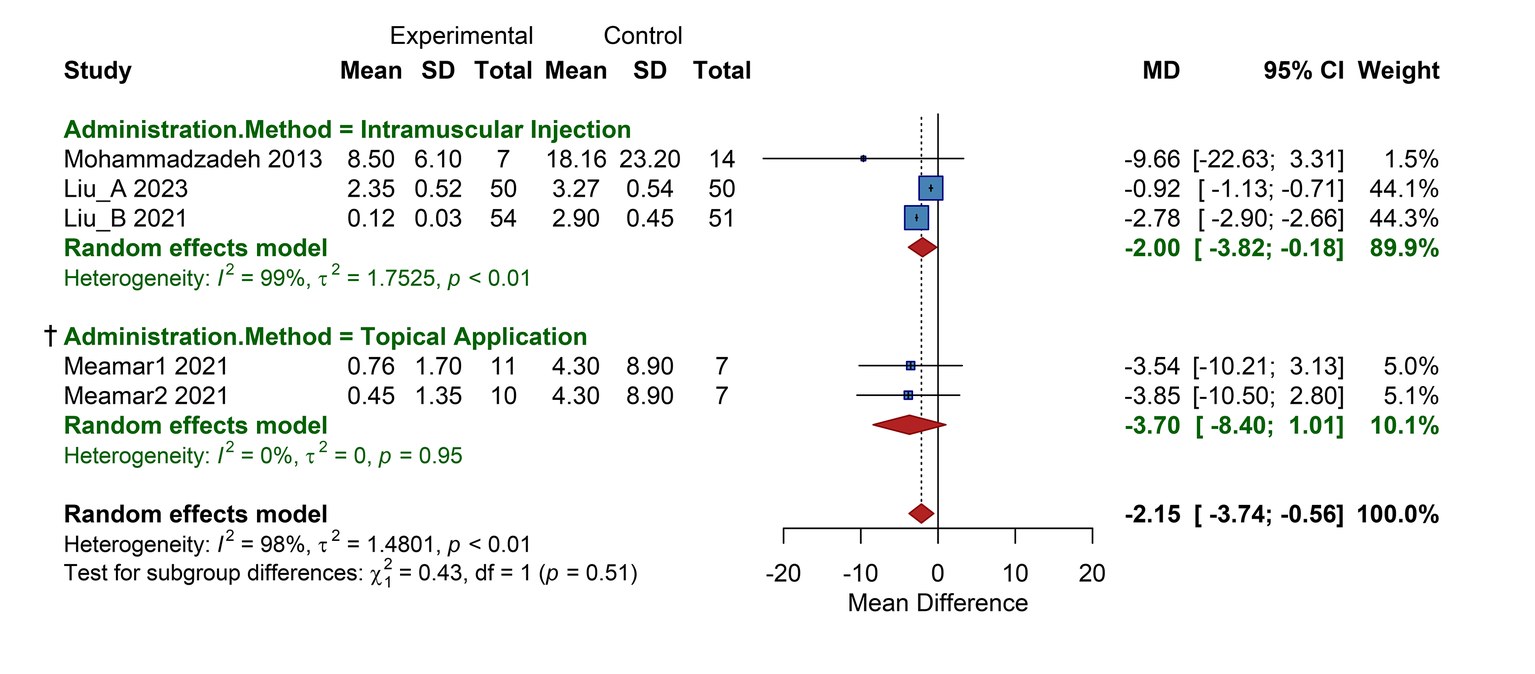


Fig S58 Subgroup Analysis of Ulcer Area by Administration Method, SD: Standard Deviation, † Results based on sparse data (number of studies k < 3) should be interpreted with caution.

## Fig S59


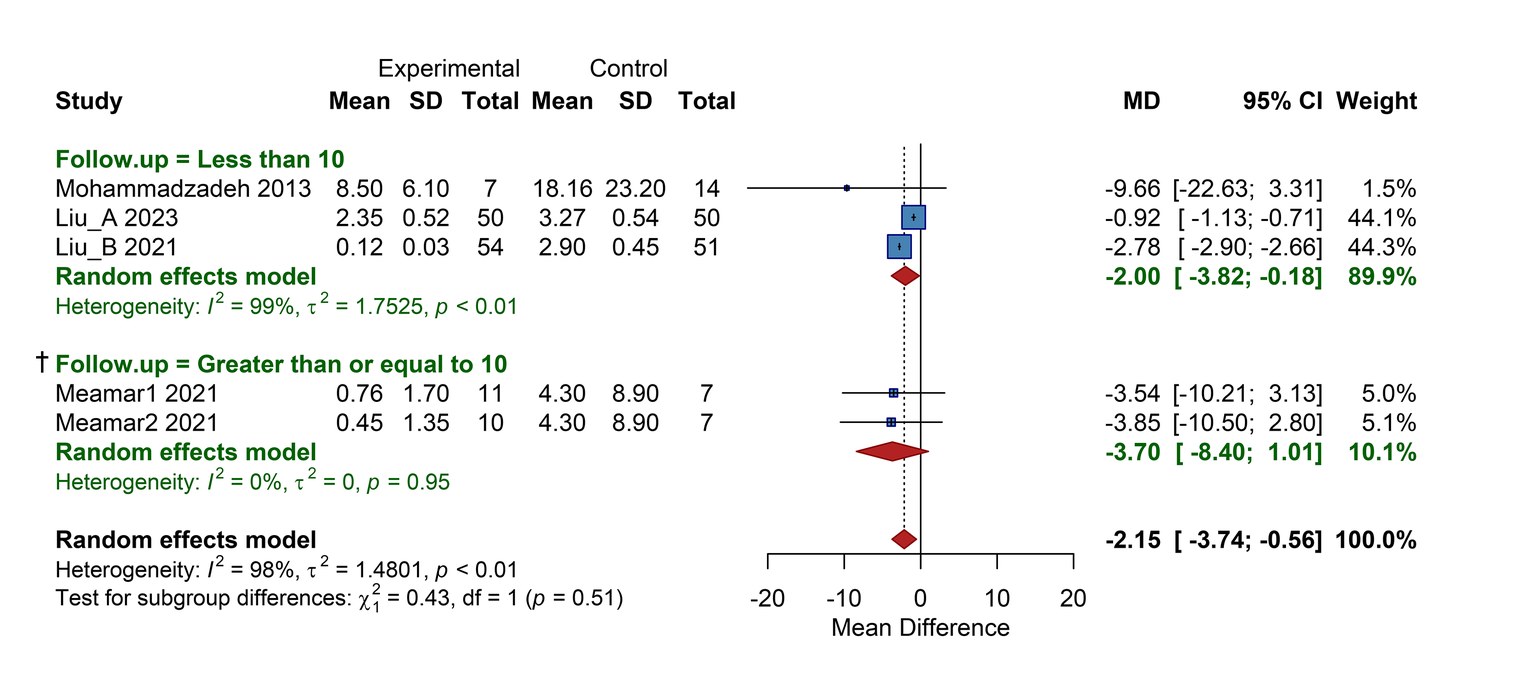


Fig S59 Subgroup Analysis of Ulcer Area by Follow-up Duration, SD: Standard Deviation, † Results based on sparse data (number of studies k < 3) should be interpreted with caution.

## Fig S60


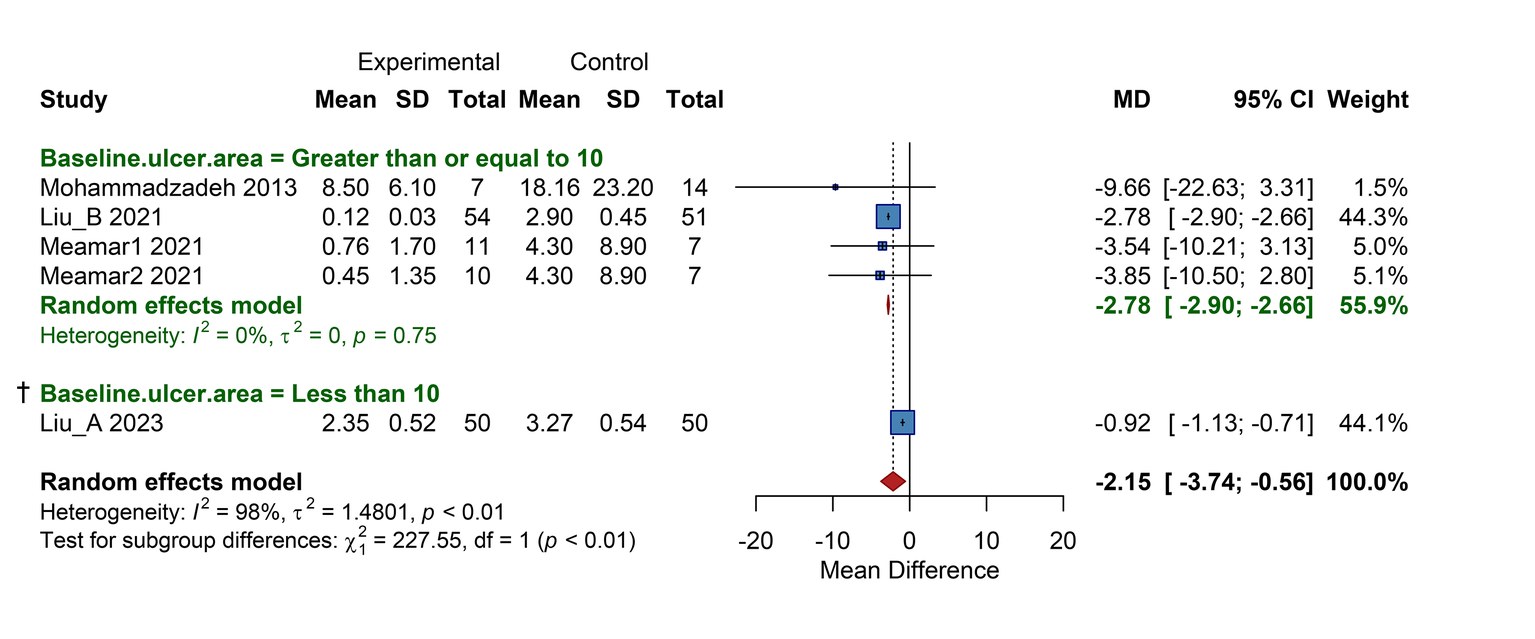


Fig S60 Subgroup Analysis of Ulcer Area by Baseline Ulcer Area, SD: Standard Deviation, † Results based on sparse data (number of studies k < 3) should be interpreted with caution.

## Fig S61


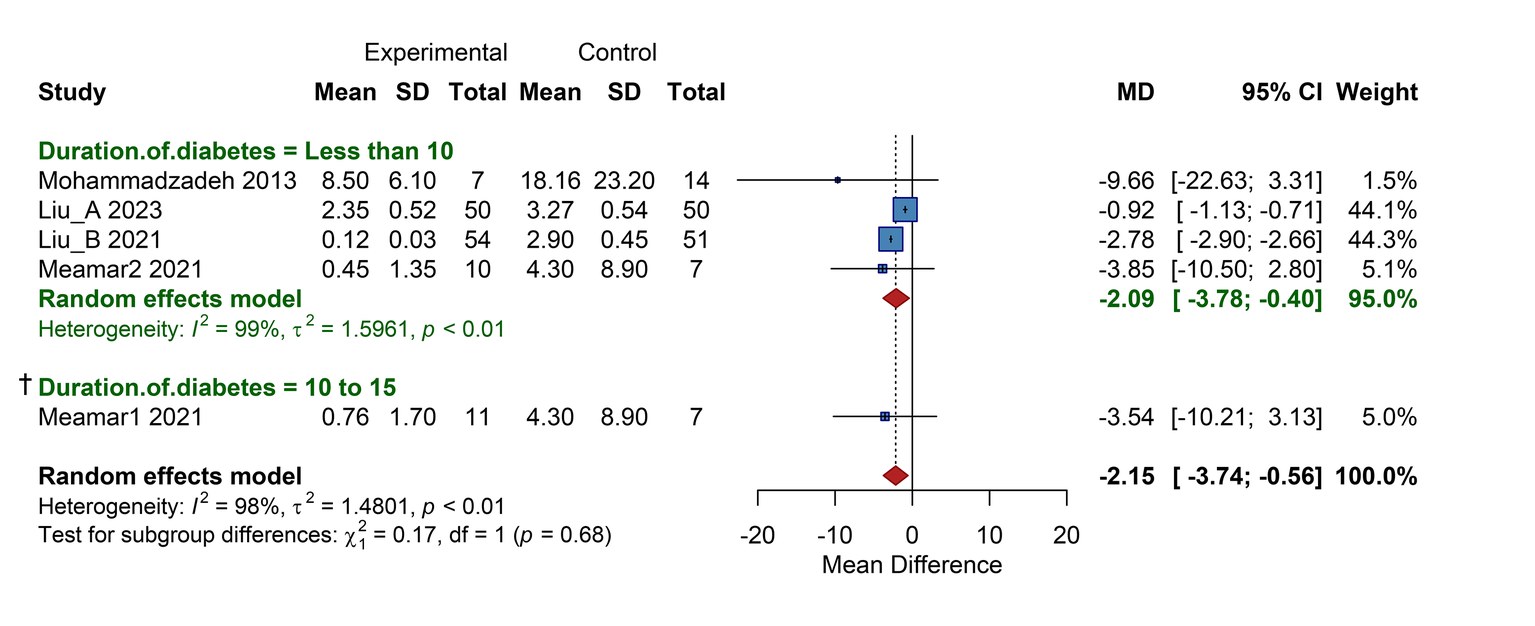


Fig S61 Subgroup Analysis of Ulcer Area by Duration of Diabetes, SD: Standard Deviation, † Results based on sparse data (number of studies k < 3) should be interpreted with caution.

## Fig S62


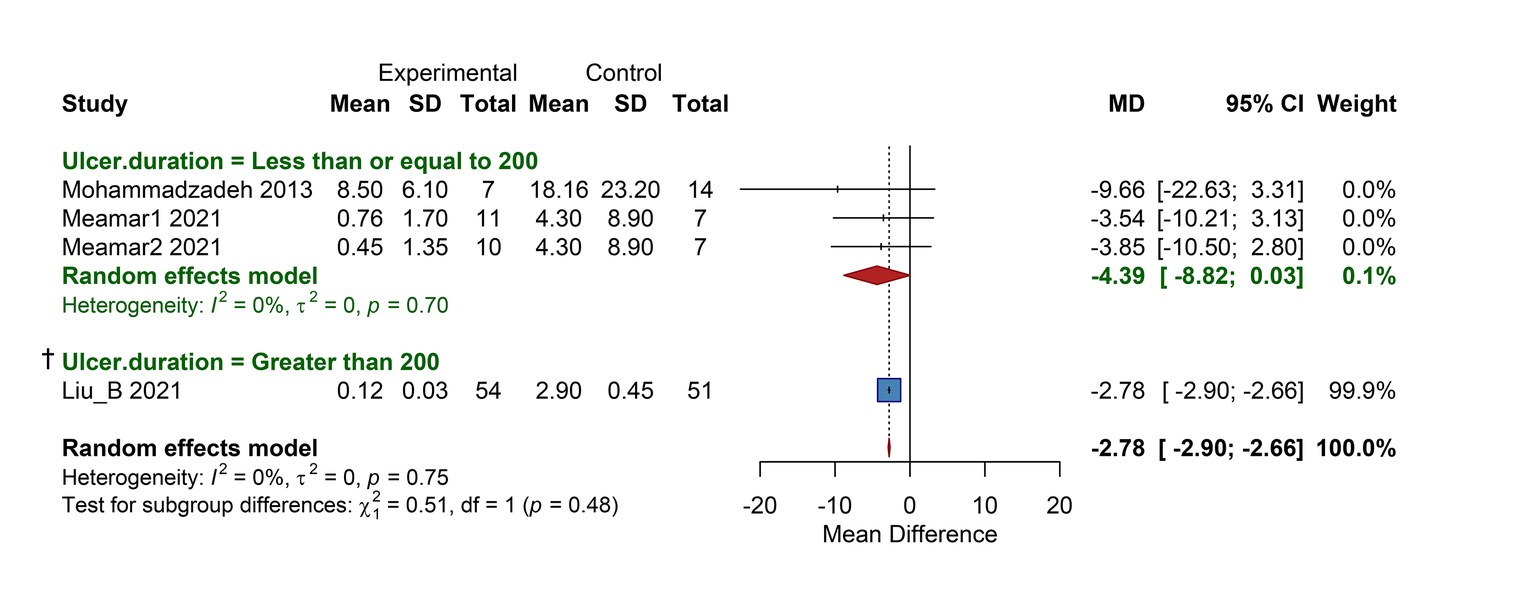


Fig S62 Subgroup Analysis of Ulcer Area by Ulcer Duration, SD: Standard Deviation, † Results based on sparse data (number of studies k < 3) should be interpreted with caution.

## Fig S63


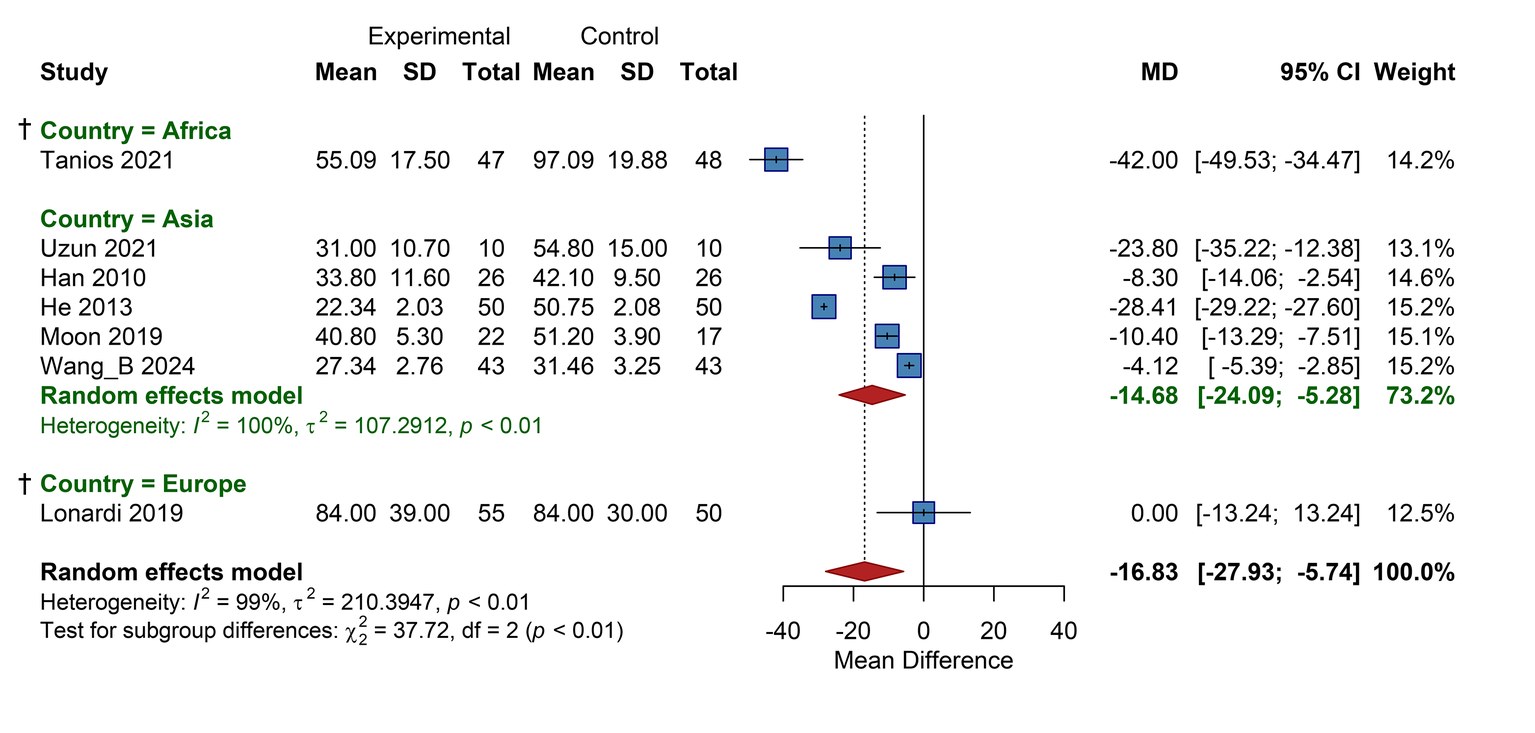


Fig S63 Subgroup Analysis of Ulcer Healing Time by Country, SD: Standard Deviation, † Results based on sparse data (number of studies k < 3) should be interpreted with caution.

## Fig S64


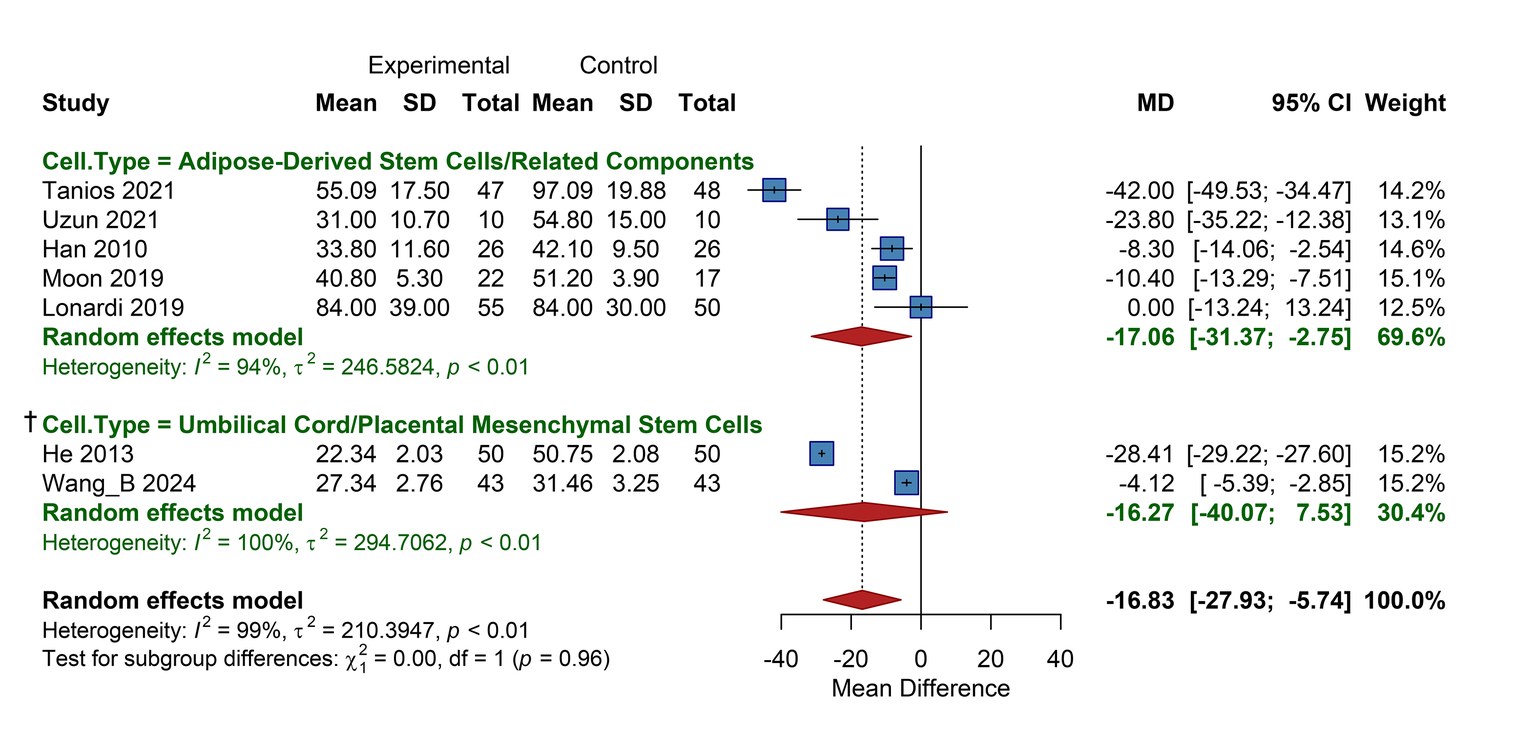


Fig S64 Subgroup Analysis of Ulcer Healing Time by Cell Type, SD: Standard Deviation, † Results based on sparse data (number of studies k < 3) should be interpreted with caution.

## Fig S65


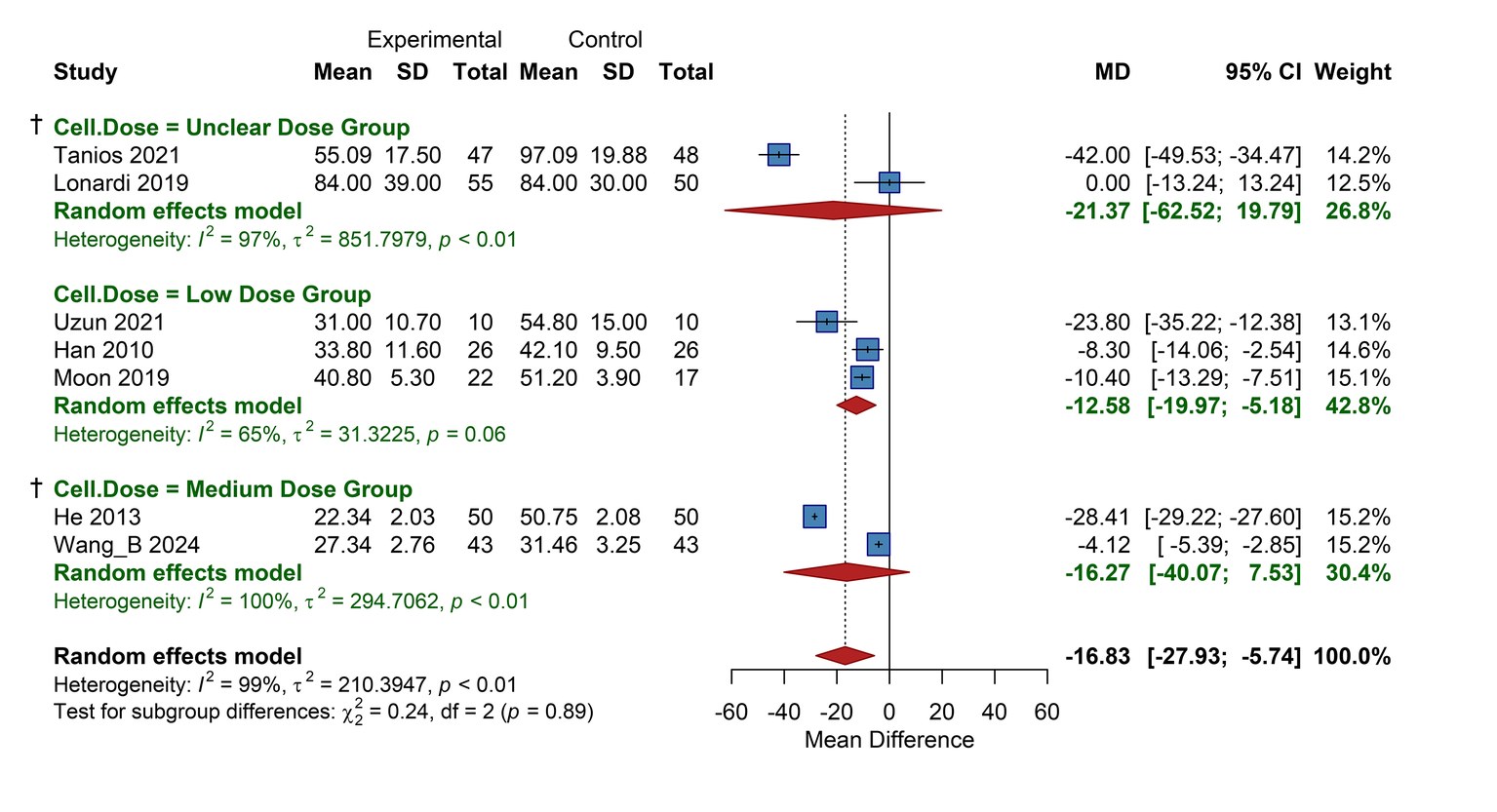


Fig S65 Subgroup Analysis of Ulcer Healing Time by Cell Dose, SD: Standard Deviation, Low-dose group: 0.5×10⁶ to 8×10⁶ cells, Medium-dose group: 1×10⁷ to 8.6×10⁷ cells, High-dose group: 3×10⁸ to 1.2×10⁹ cells, Ultra-high-dose group: ≥2×10⁹ cells, unclear dose group: No quantifiable total cell count available, † Results based on sparse data (number of studies k < 3) should be interpreted with caution.

## Fig S66


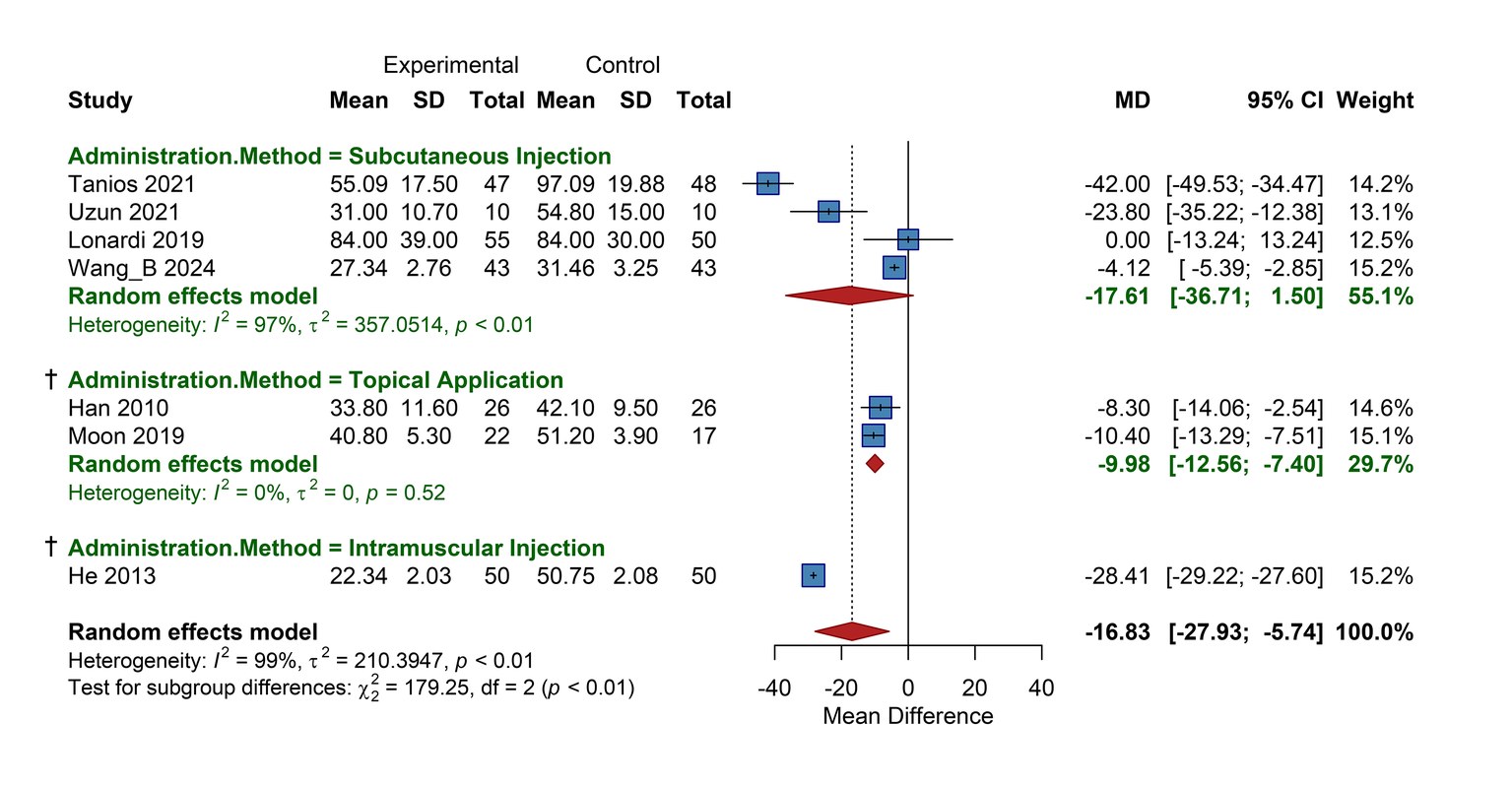


Fig S66 Subgroup Analysis of Ulcer Healing Time by Administration Method, SD: Standard Deviation, † Results based on sparse data (number of studies k < 3) should be interpreted with caution.

## Fig S67


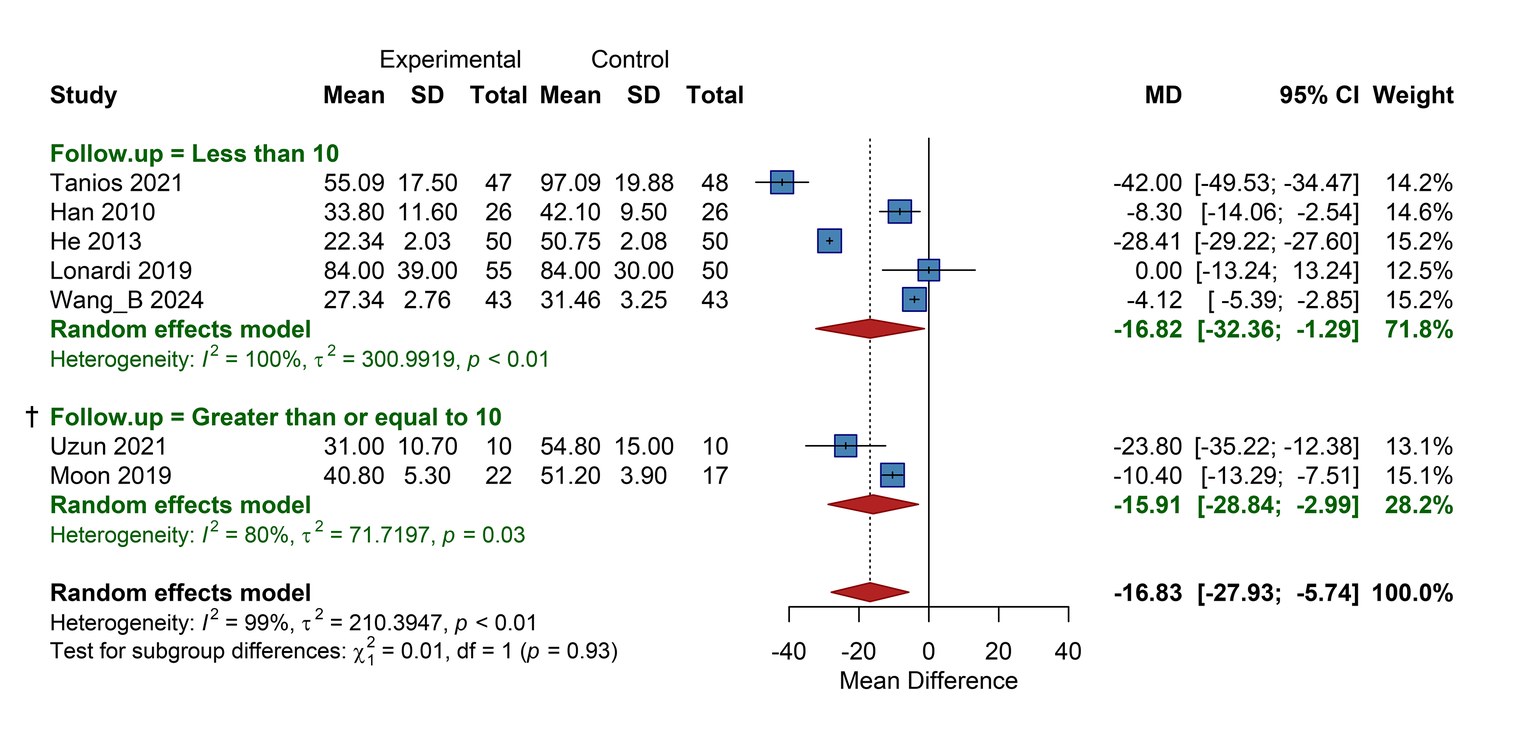


Fig S67 Subgroup Analysis of Ulcer Healing Time by Follow-up Duration, SD: Standard Deviation, † Results based on sparse data (number of studies k < 3) should be interpreted with caution.

## Fig S68


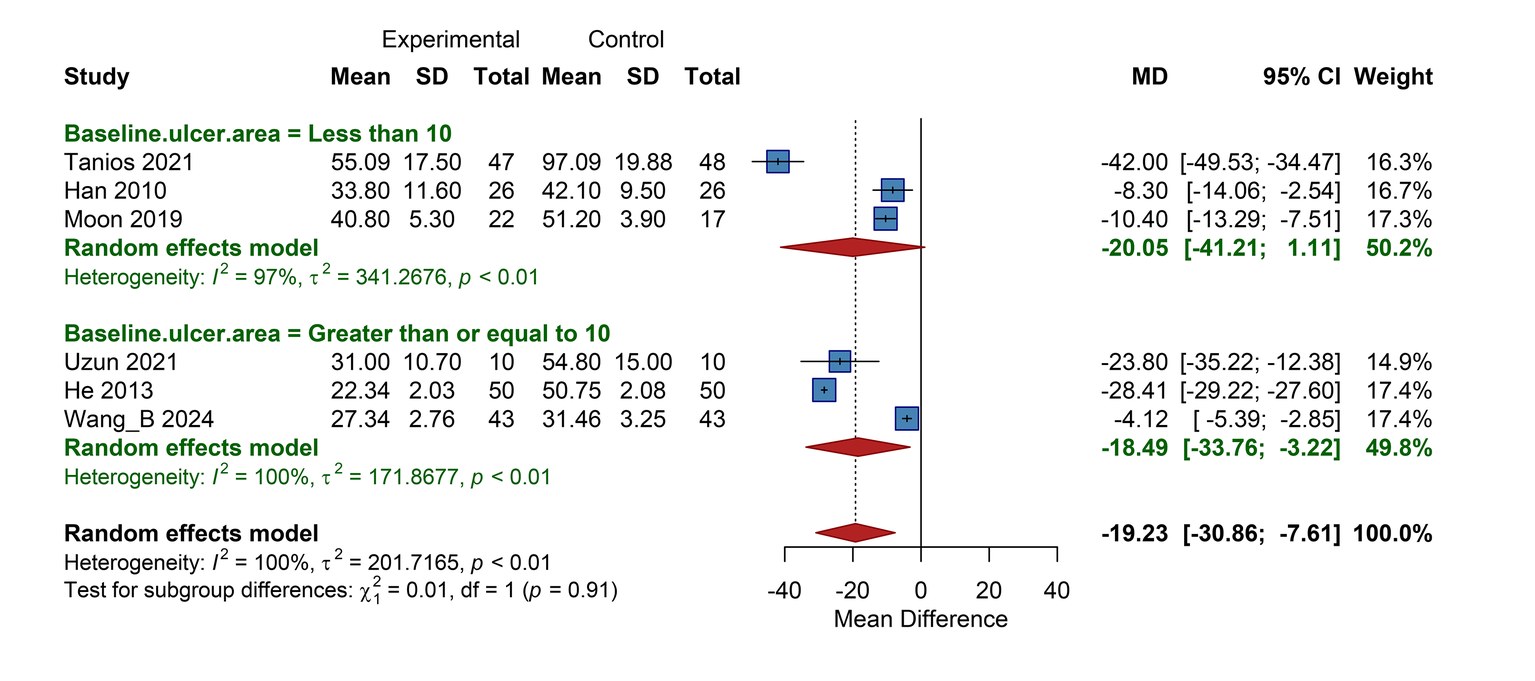


Fig S68 Subgroup Analysis of Ulcer Healing Time by Baseline Ulcer Area, SD: Standard Deviation, † Results based on sparse data (number of studies k < 3) should be interpreted with caution.

## Fig S69


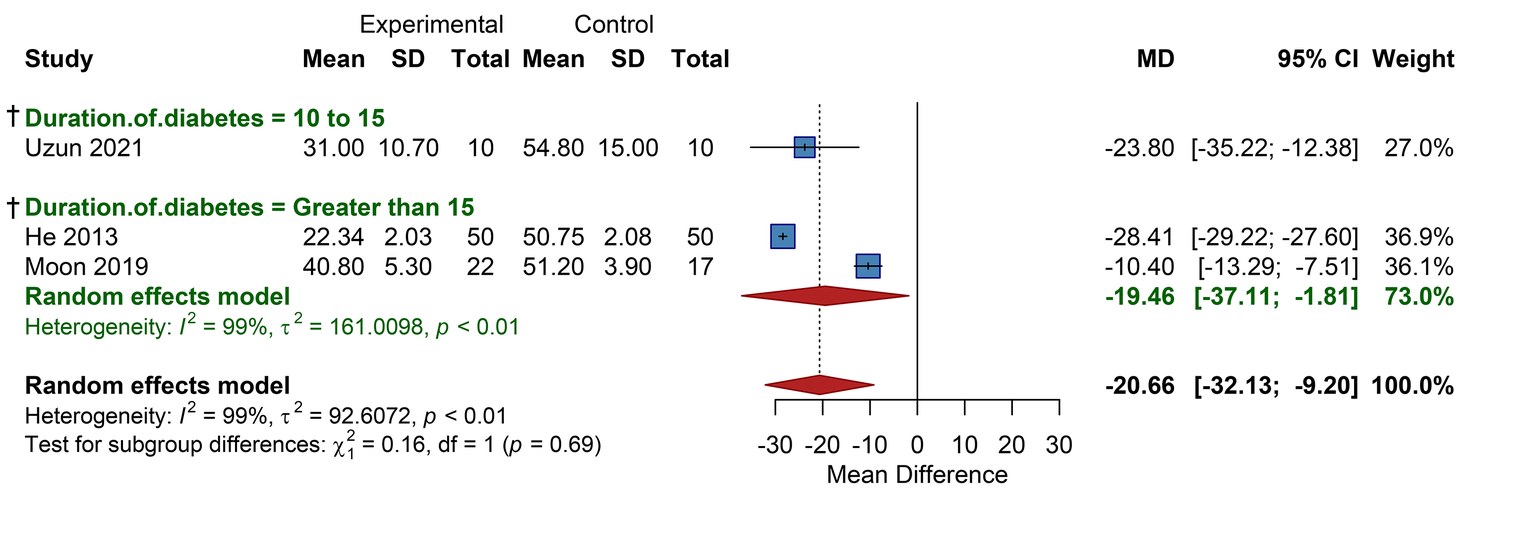


Fig S69 Subgroup Analysis of Ulcer Healing Time by Duration of Diabetes, SD: Standard Deviation, † Results based on sparse data (number of studies k < 3) should be interpreted with caution.

## Fig S70


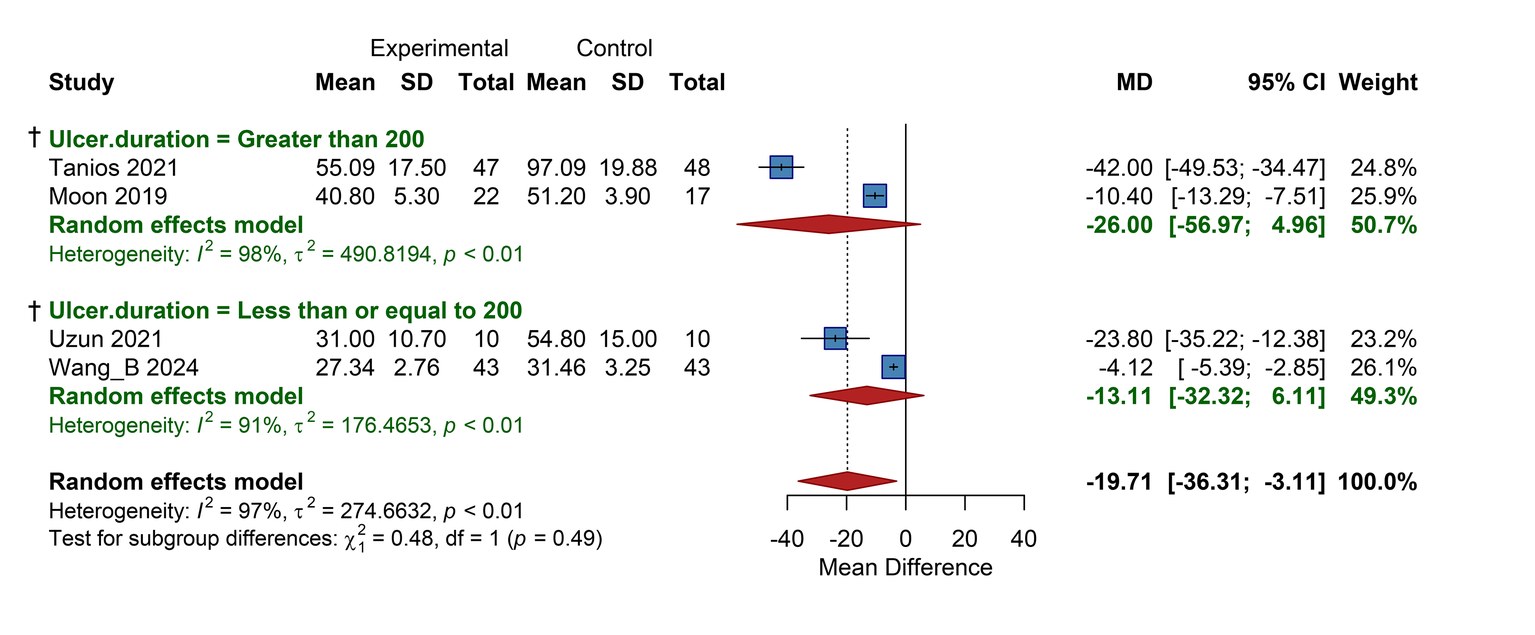


Fig S70 Subgroup Analysis of Ulcer Healing Time by Ulcer Duration, SD: Standard Deviation, † Results based on sparse data (number of studies k < 3) should be interpreted with caution.

## Fig S71


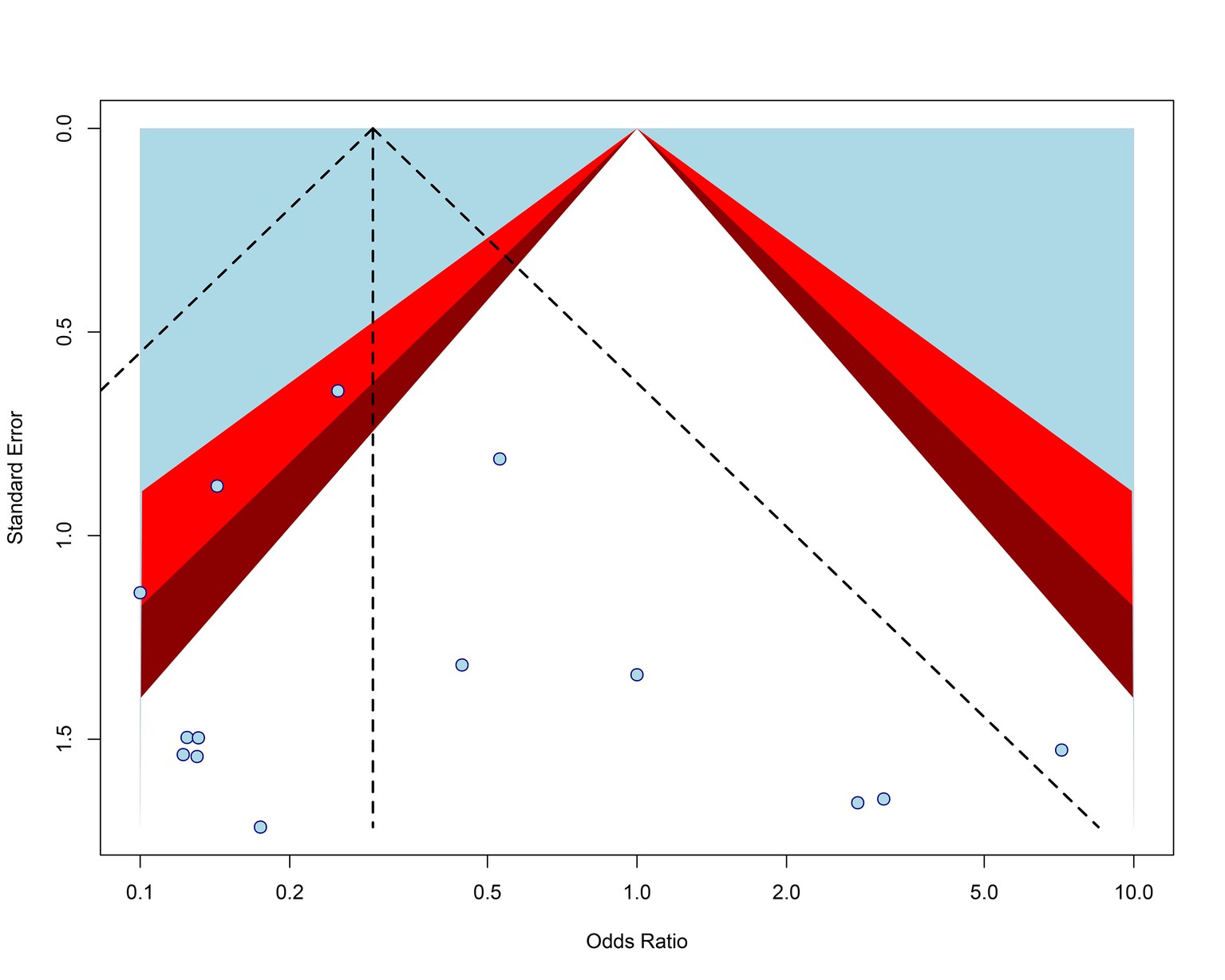


Fig S71 Amputation Rate Funnel Chart

## Fig S72


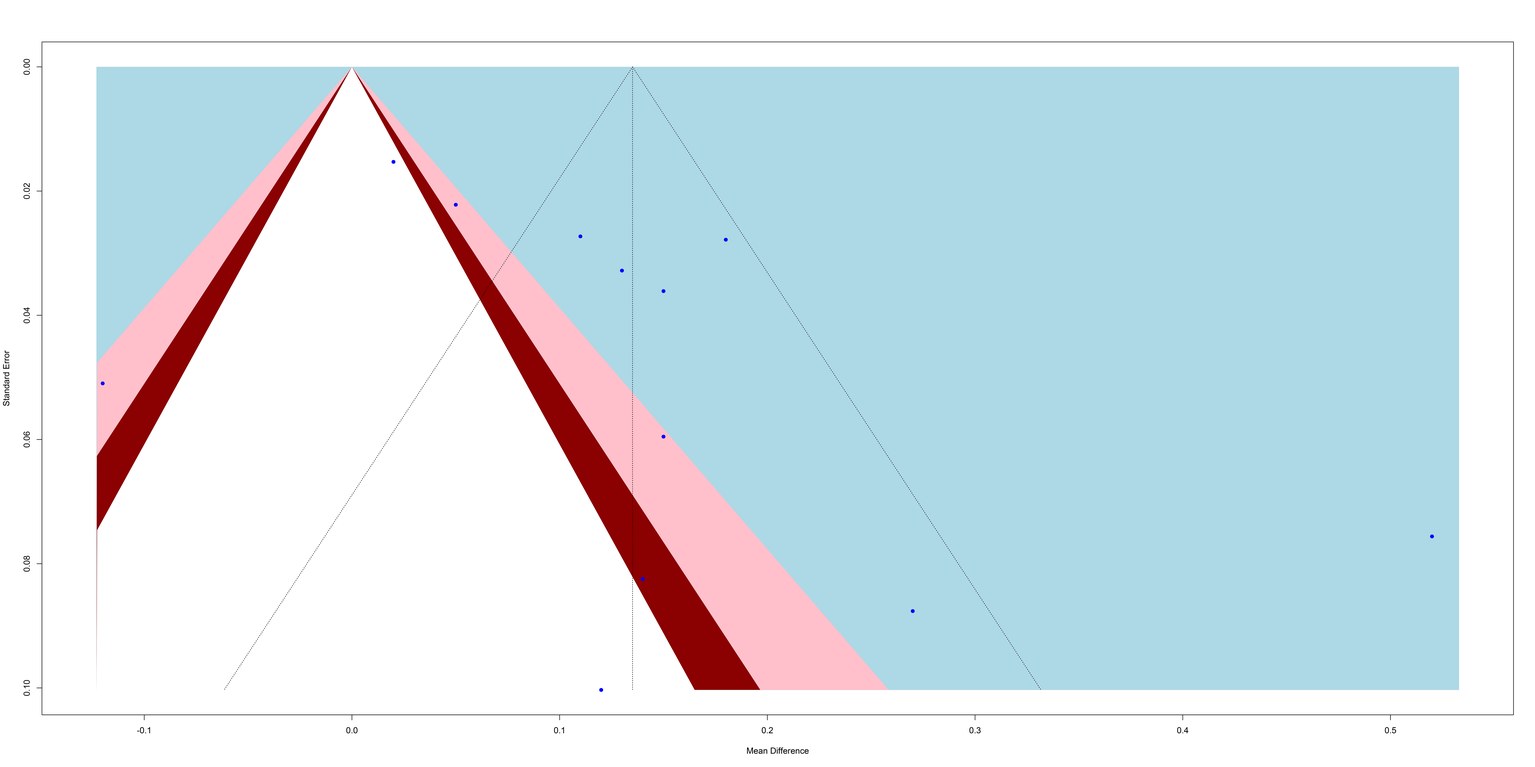


Fig S72 ABI Funnel Chart

## Fig S73


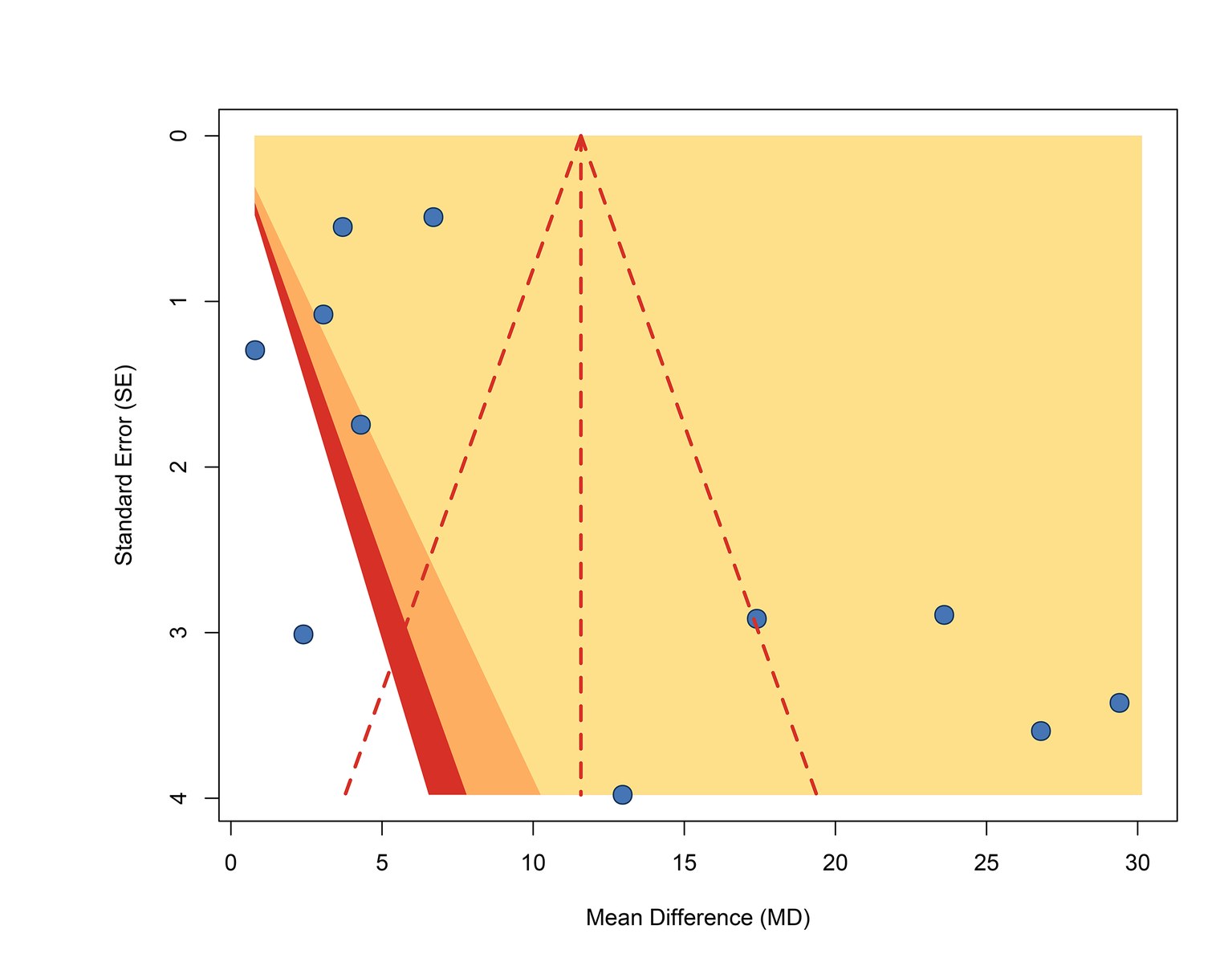


Fig S73 TcPo2 Funnel Chart

## Fig S74


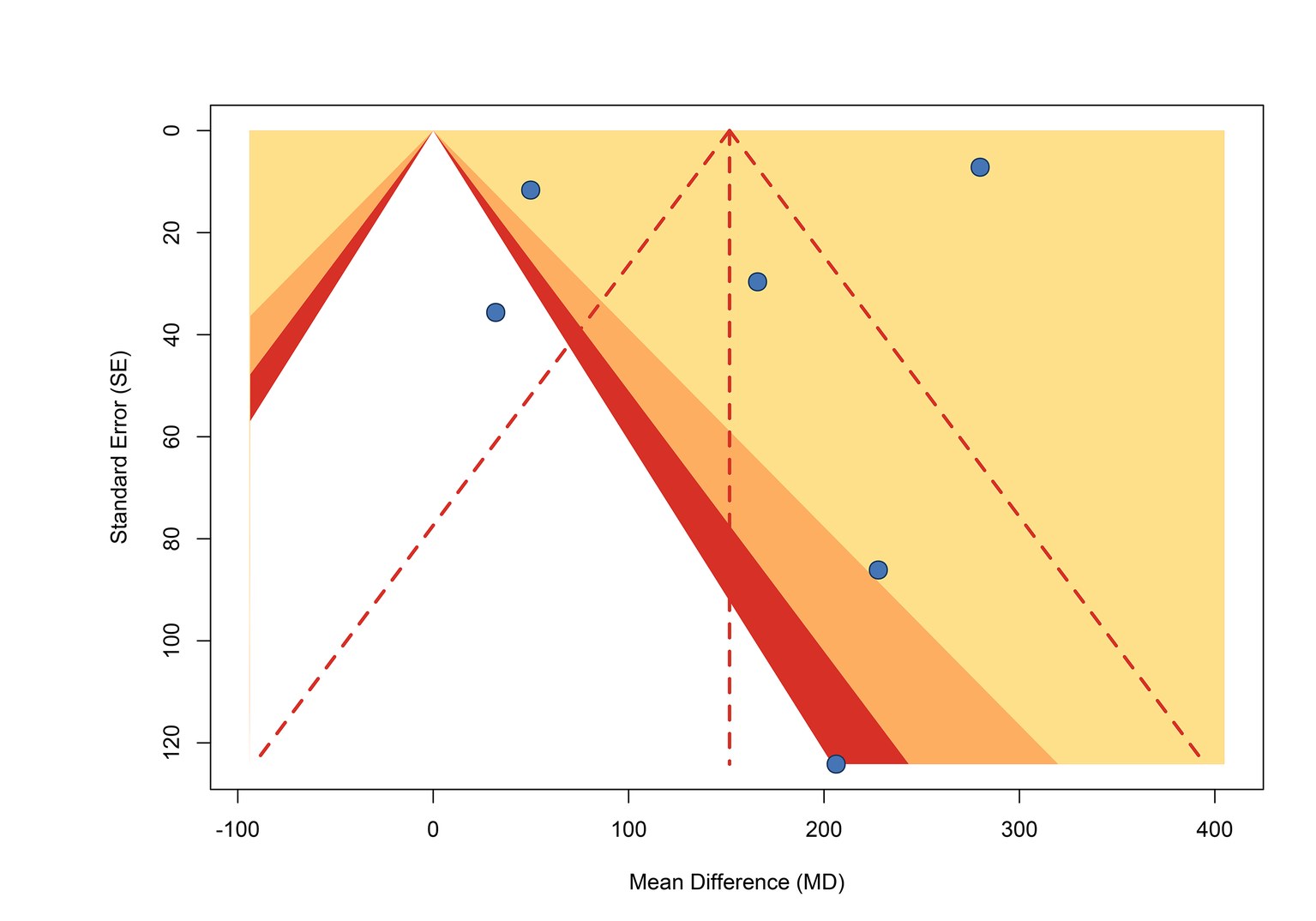


Fig S74 Painless walking distance funnel chart

## Fig S75


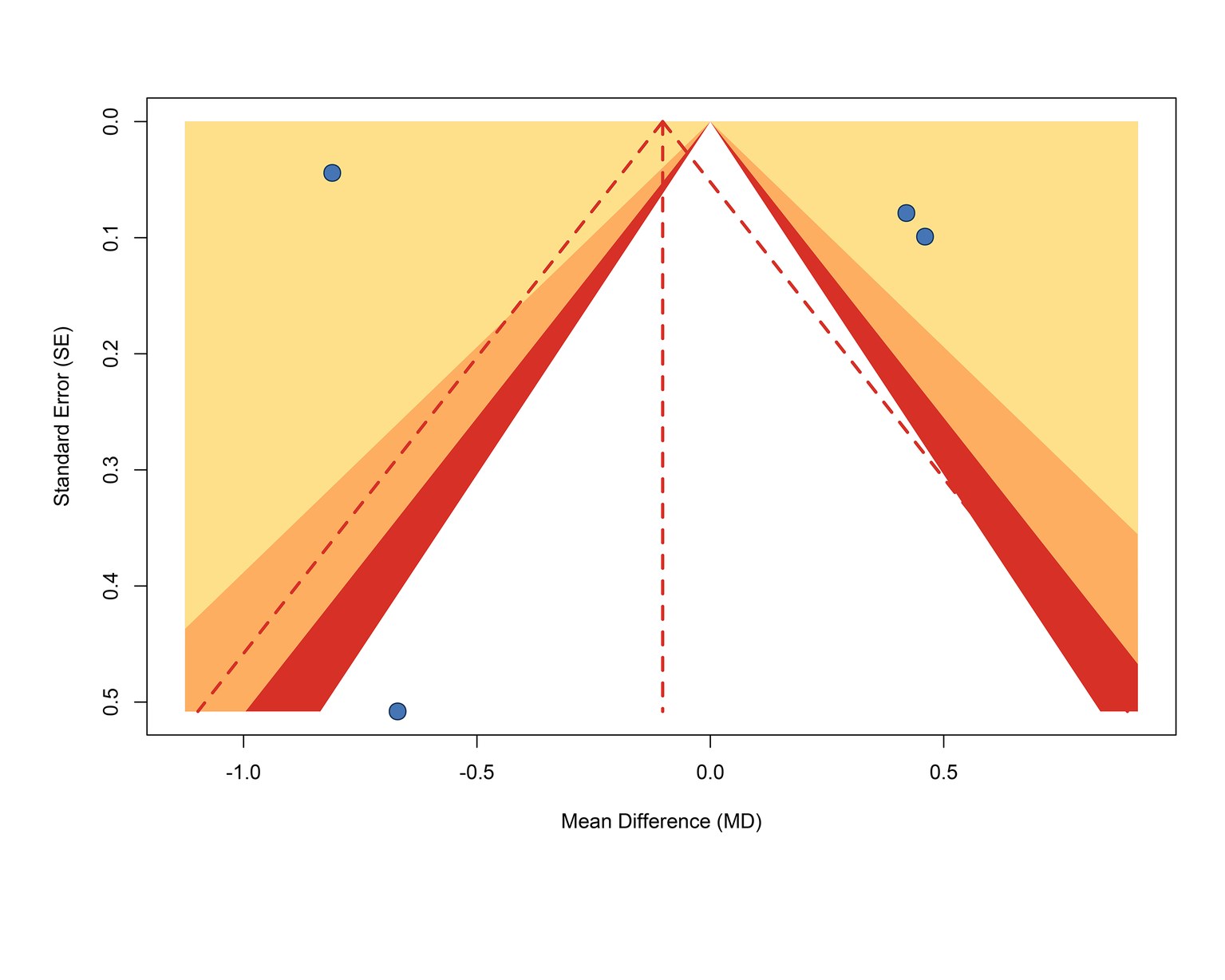


Fig S75 Painless Walking Limping Score Funnel Chart

## Fig S76


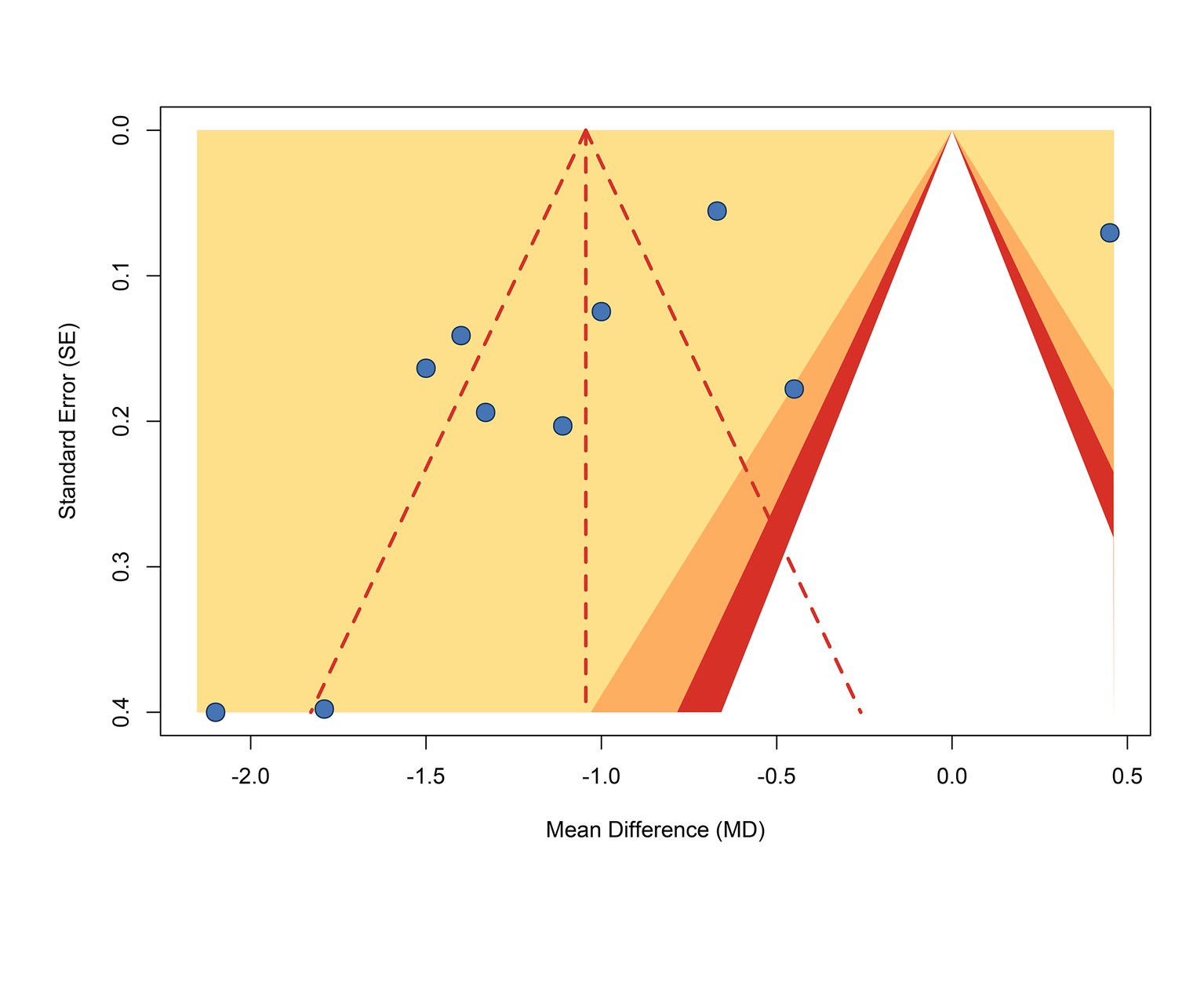


Fig S76 Resting pain score funnel chart

## Fig S77


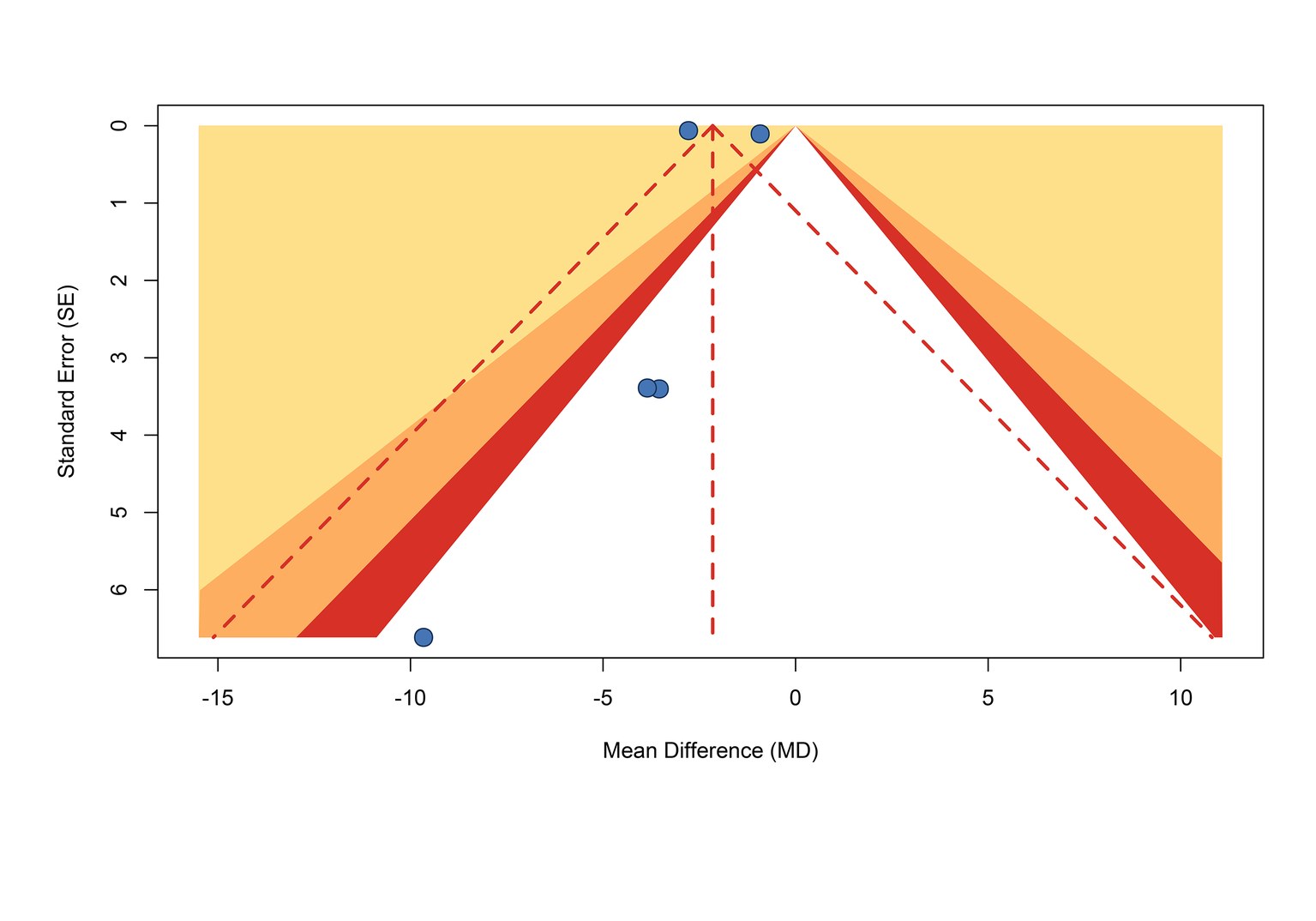


Fig S77 Result ulcer area funnel chart

## Fig S78


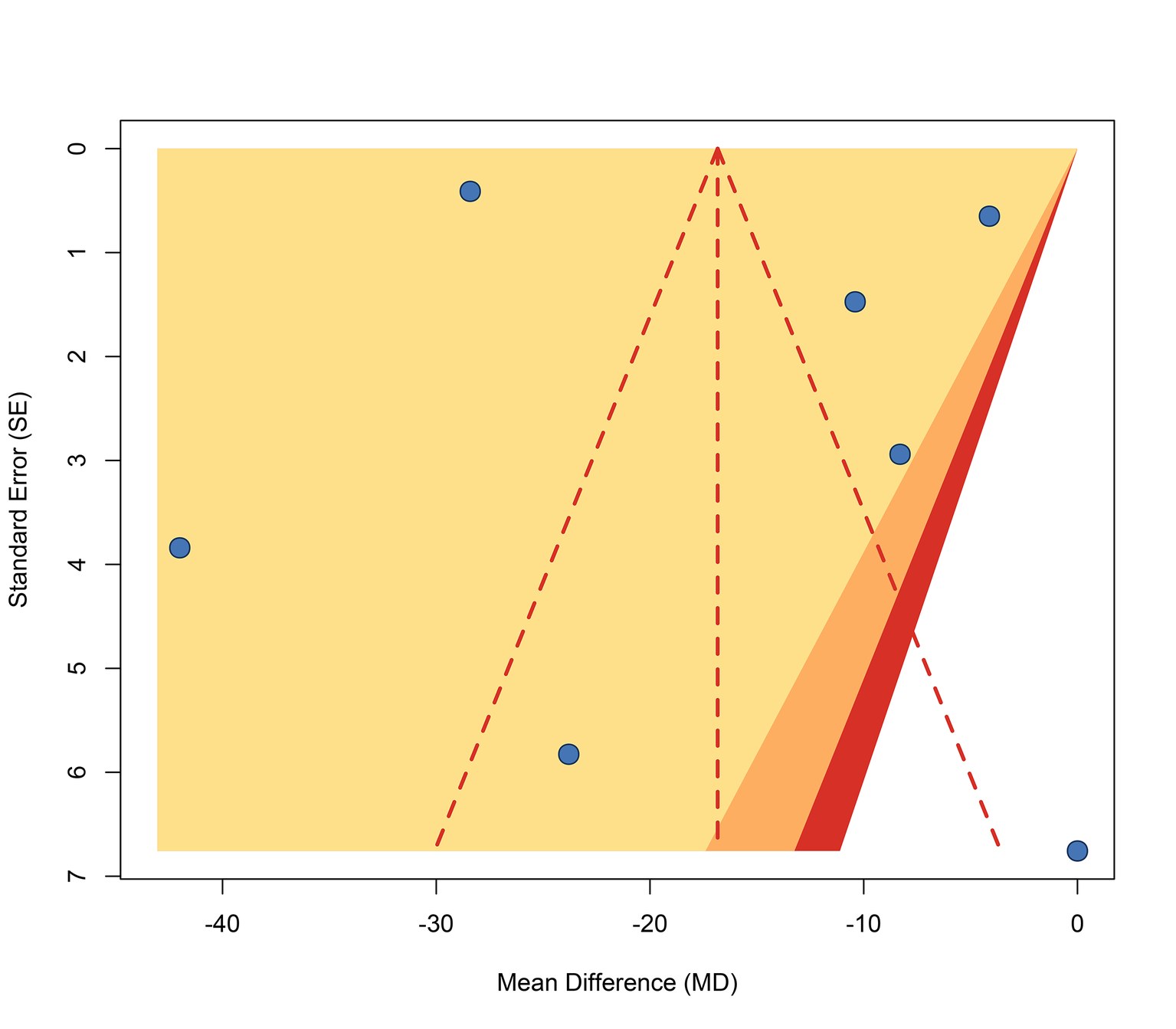


Fig S78 Ulcer Healing Time Funnel Chart

## Fig S79


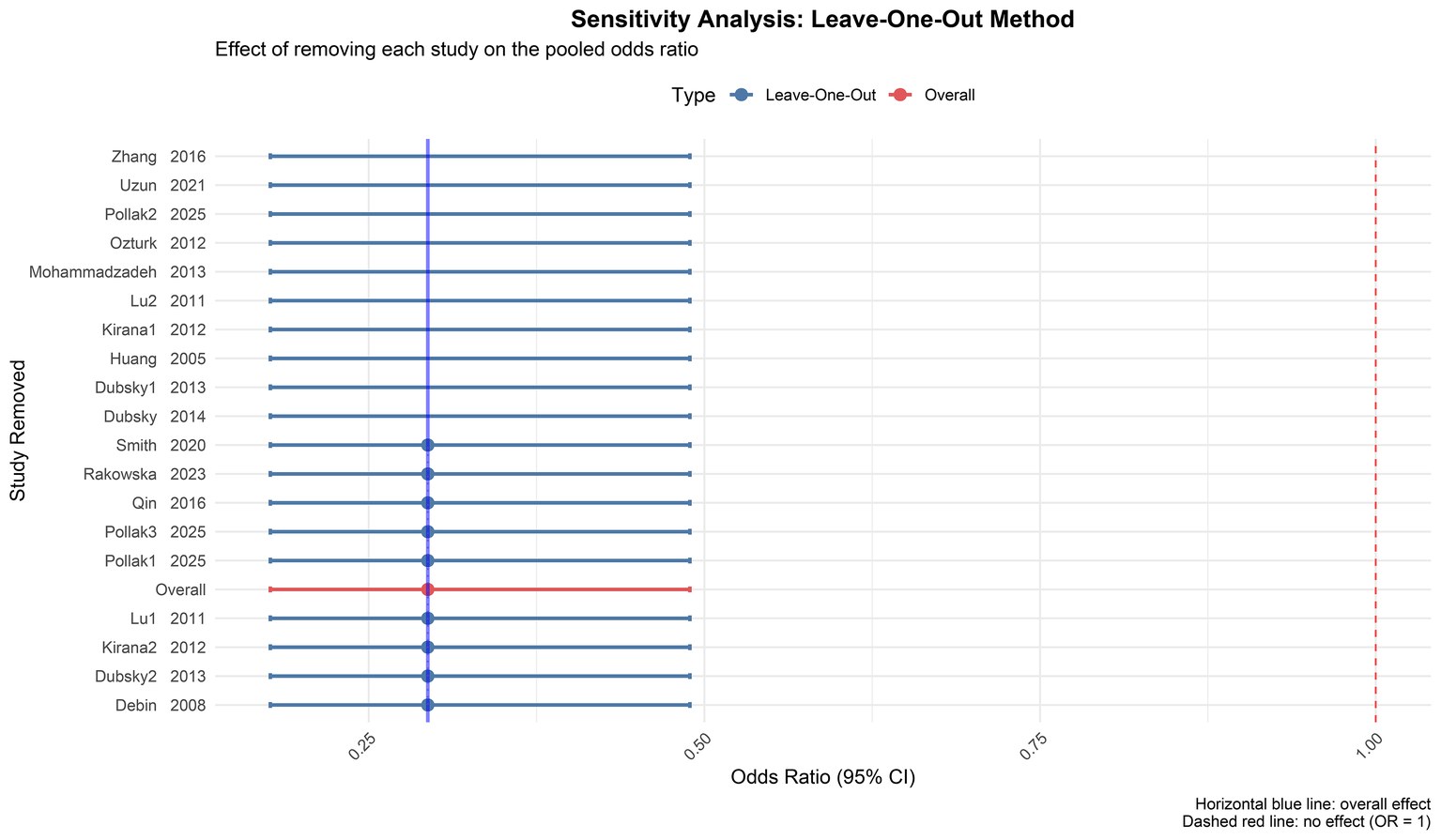


Fig S79 Amputation rate sensitivity analysis

## Fig S80


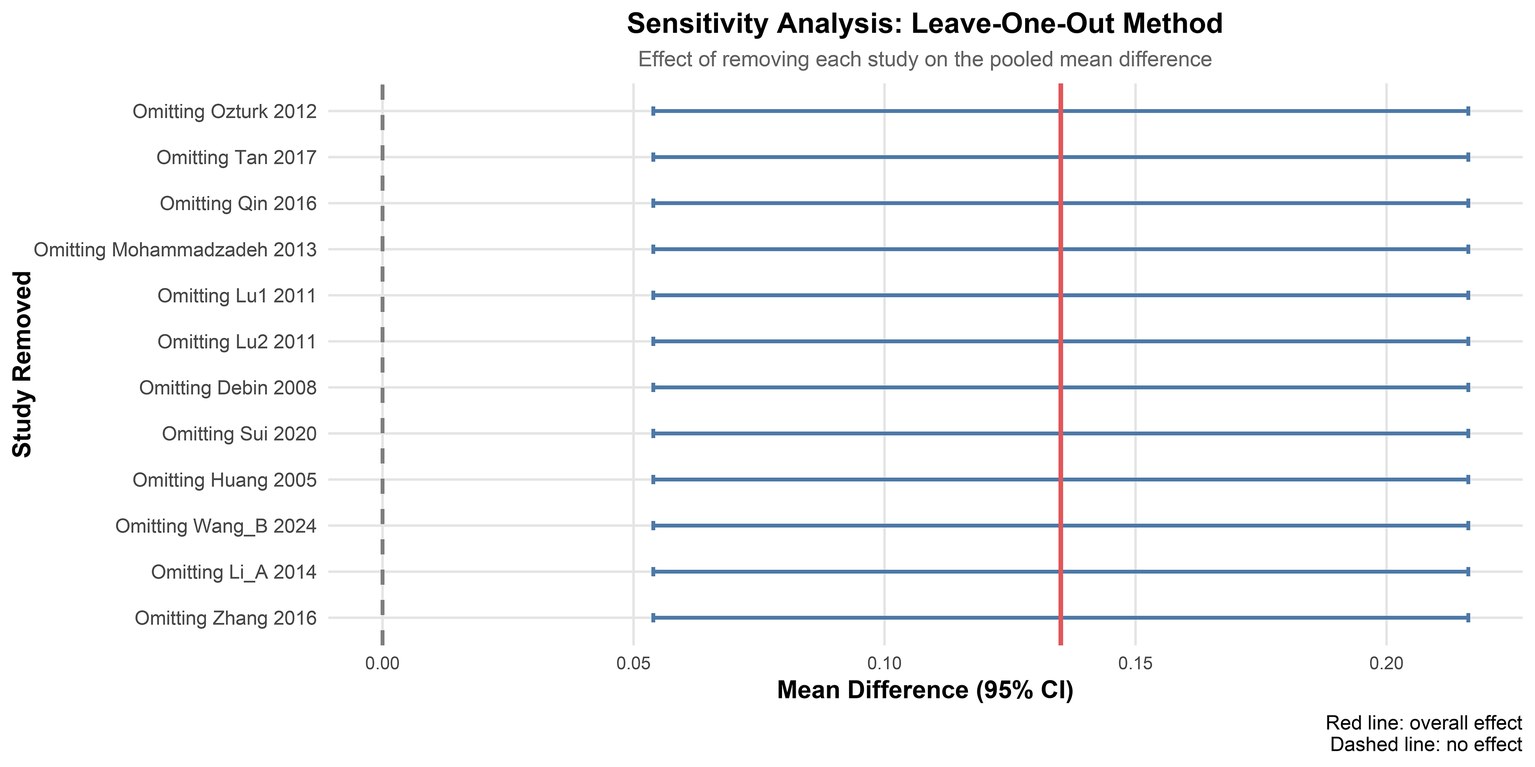


Fig S80 ABI sensitivity analysis

## Fig S81


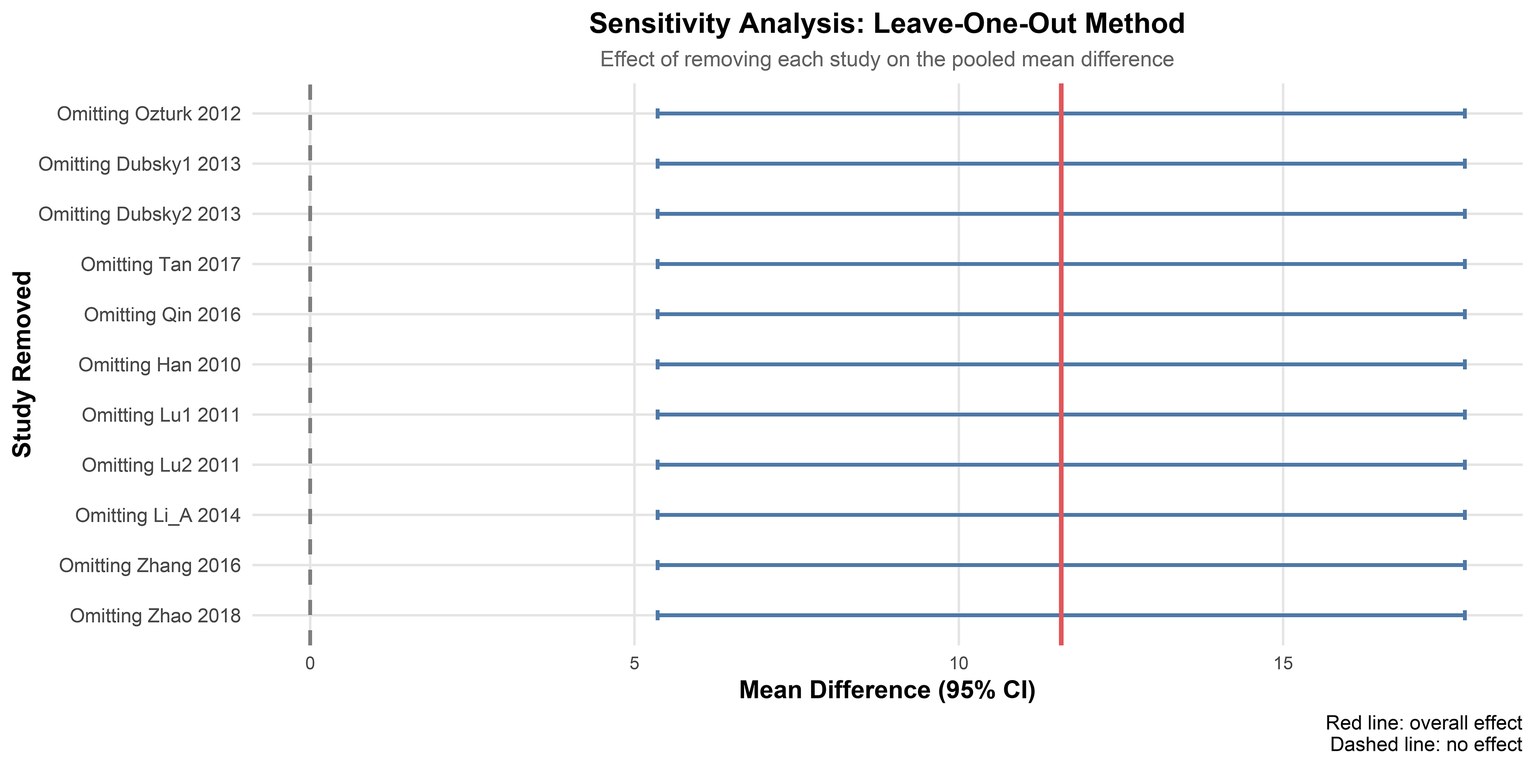


Fig S81 TcPo2 Sensitivity Analysis

## Fig S82


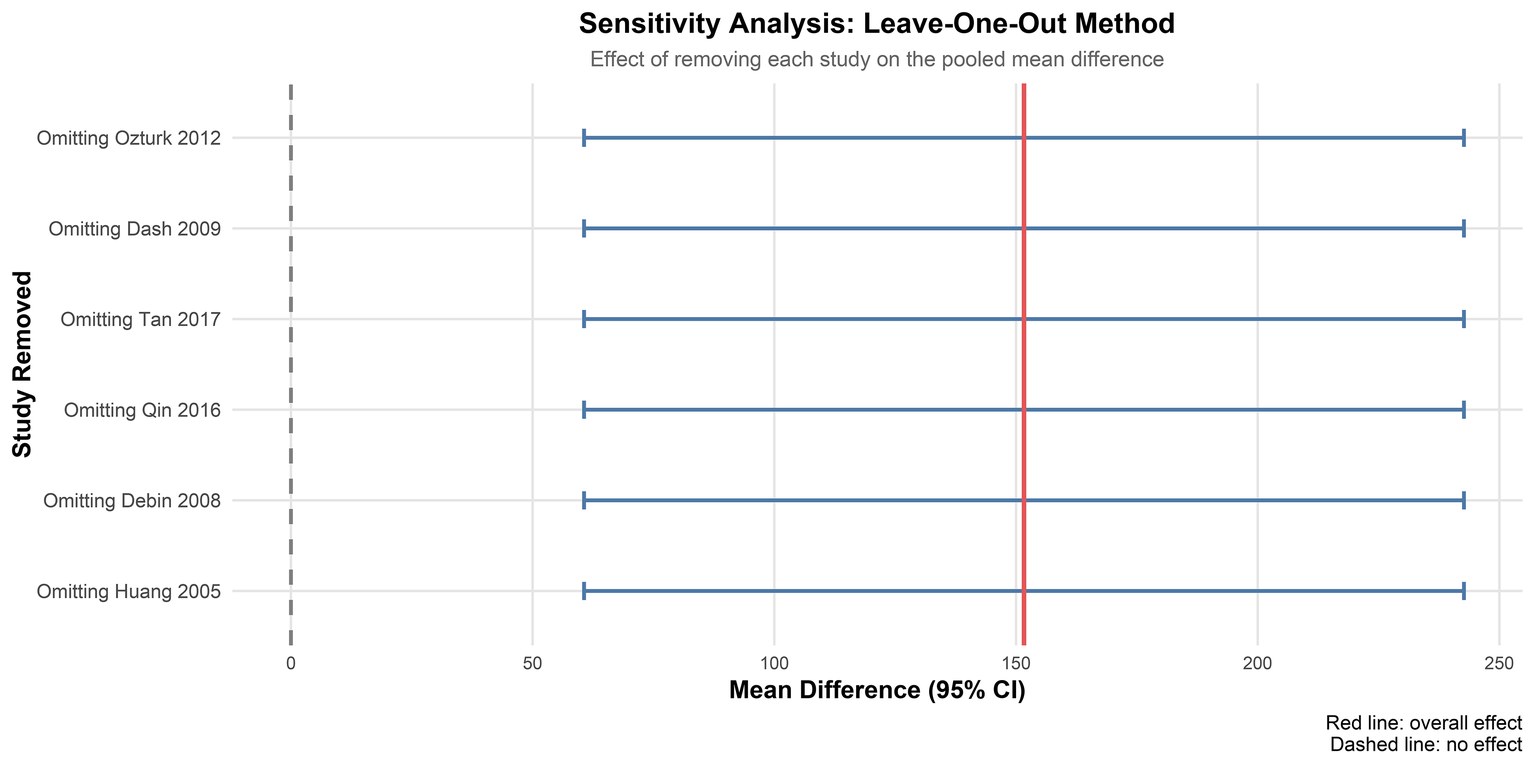


Fig S82 Sensitivity analysis of painless walking distance

## Fig S83


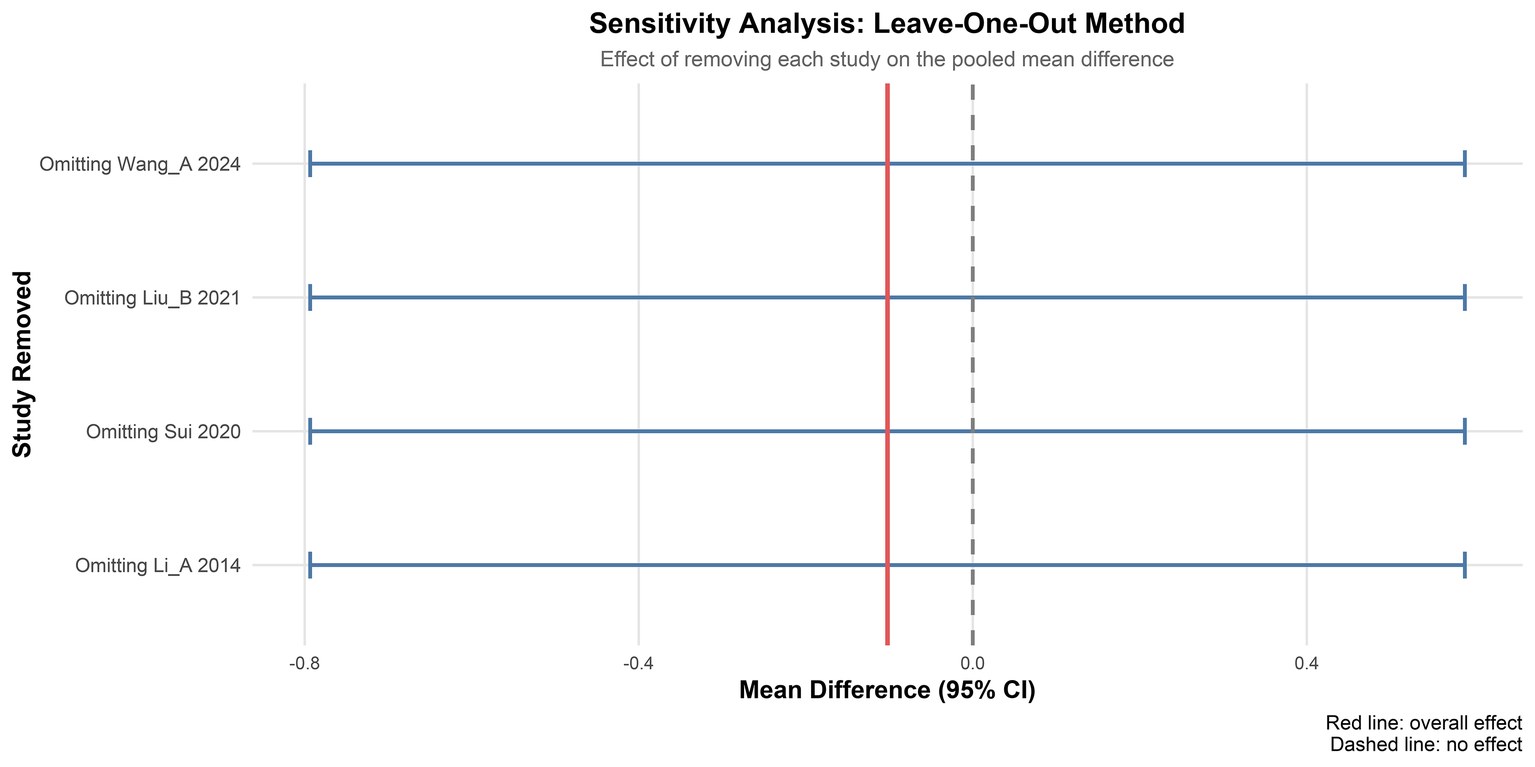


Fig S83 Sensitivity analysis of painless walking limping score

## Fig S84


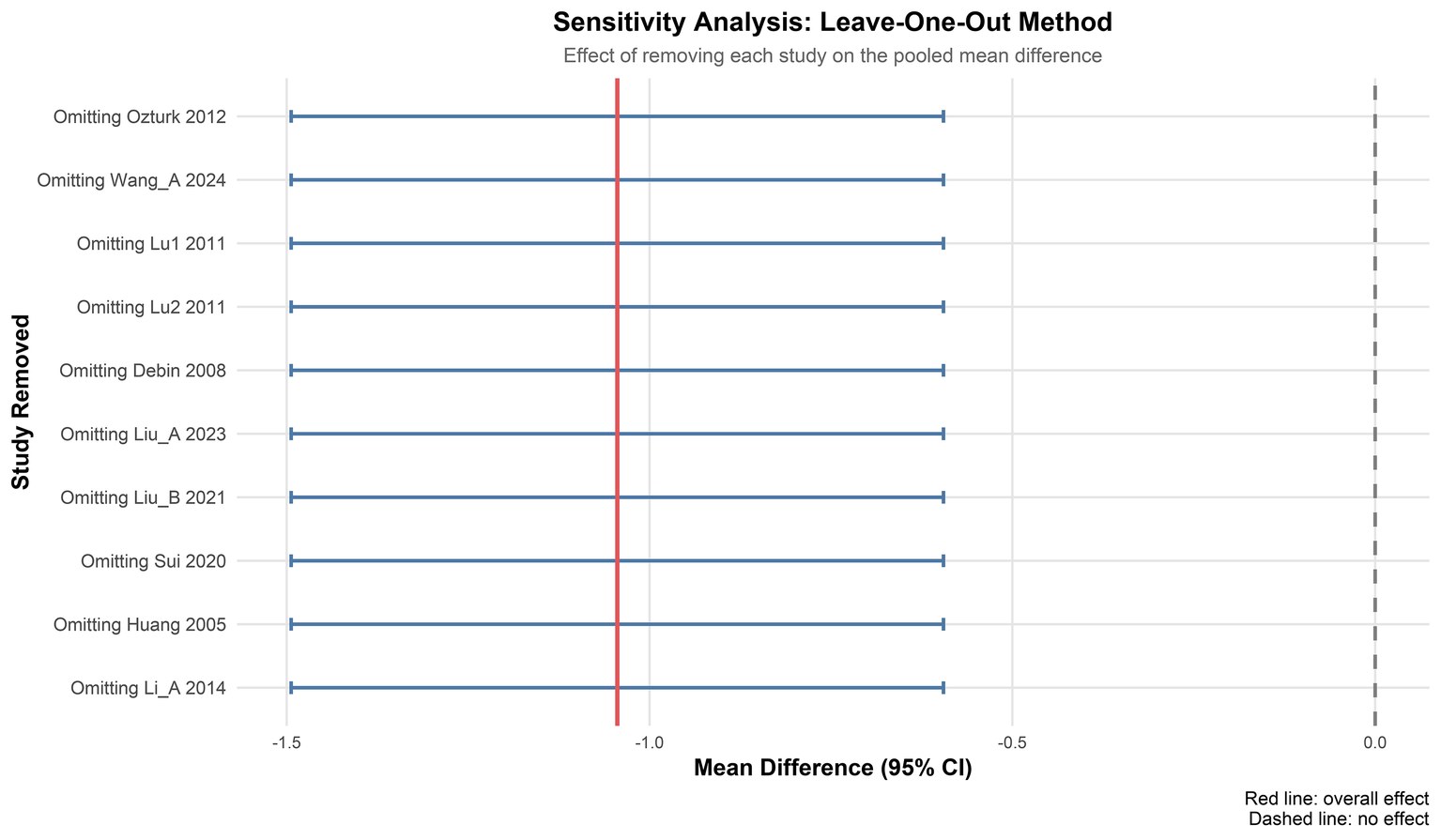


Fig S84 Resting pain score sensitivity analysis

## Fig S85


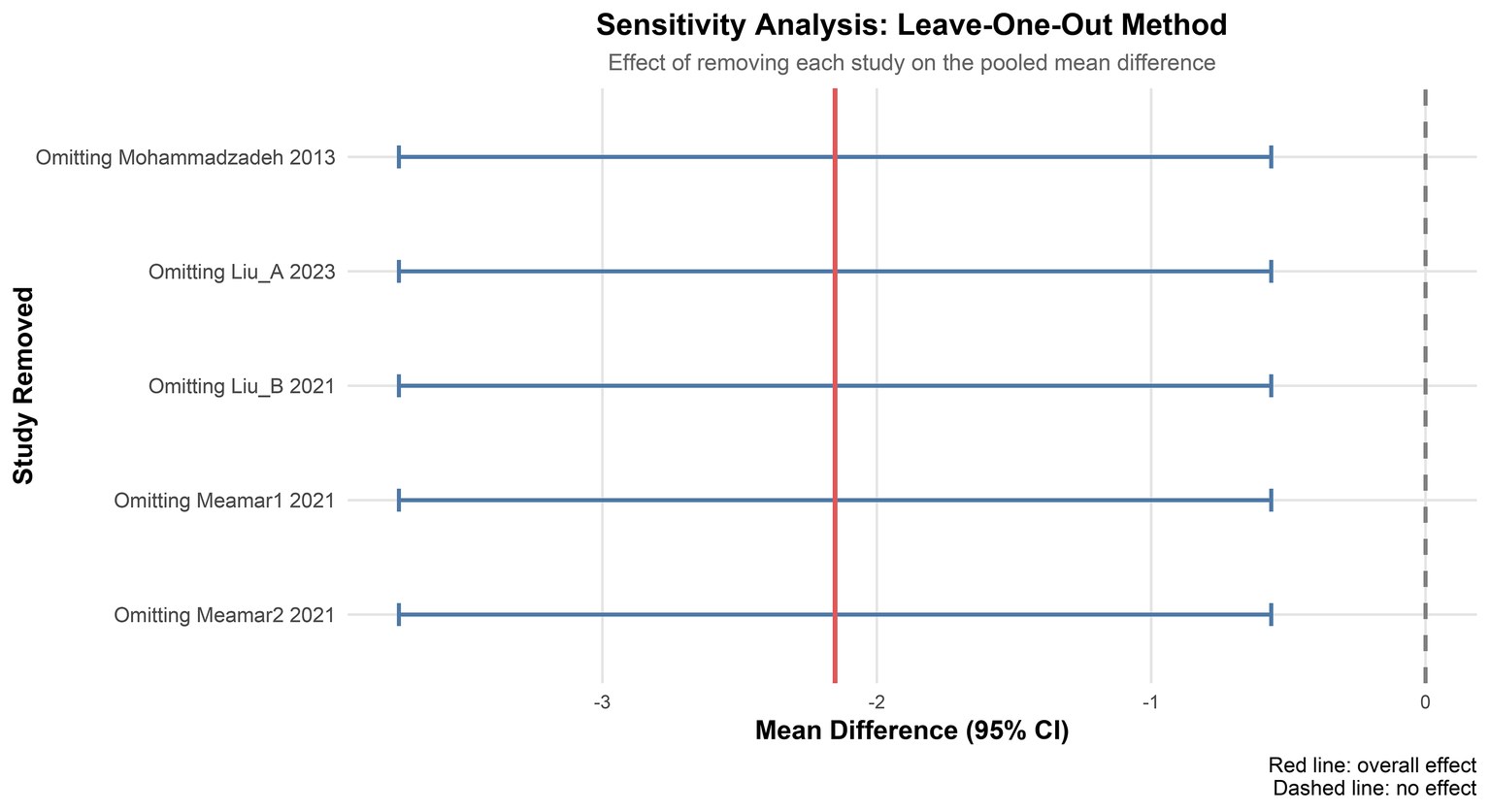


Fig S85 Results of ulcer area sensitivity analysis

## Fig S86


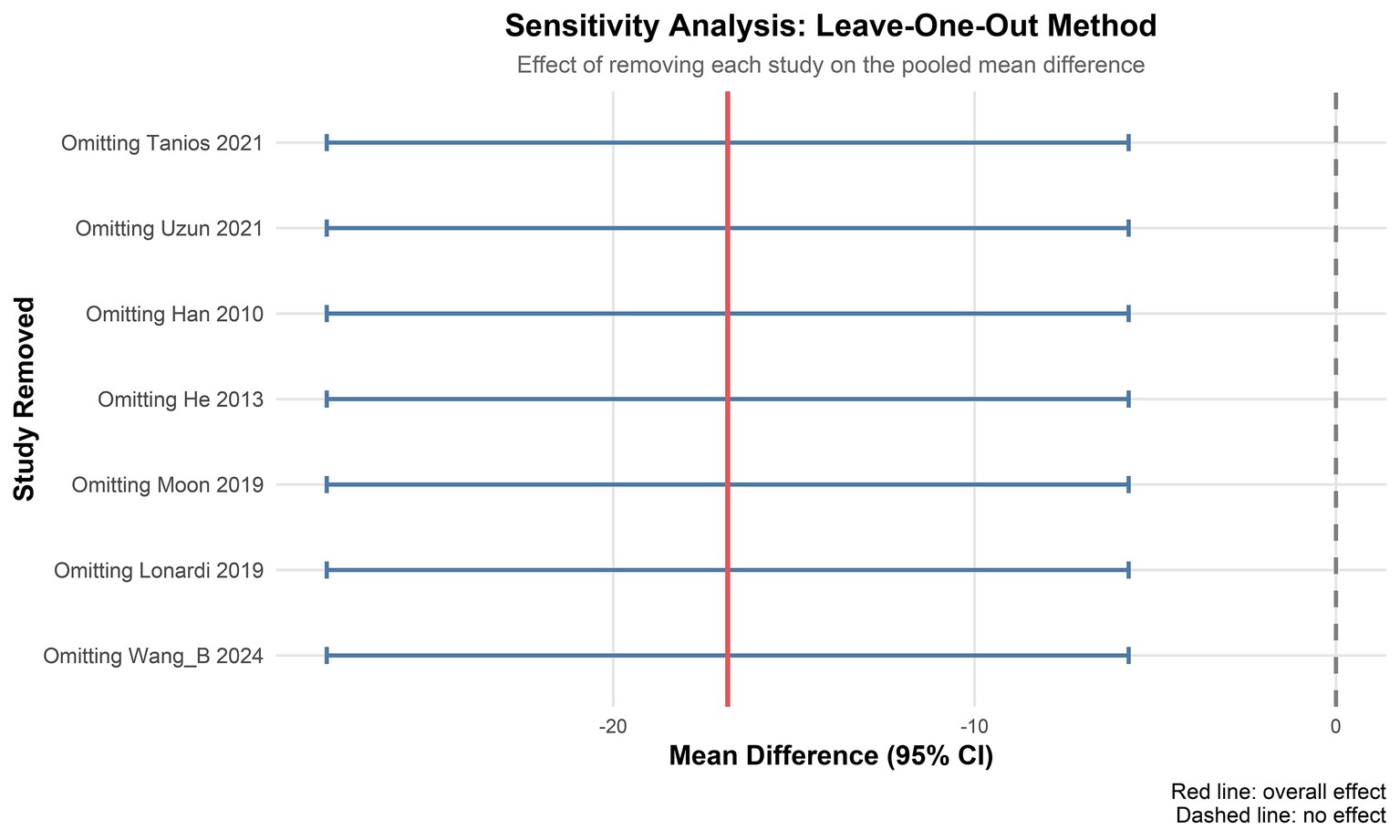


Fig S86 Analysis of Sensitivity of Ulcer Healing Time

## Fig S87


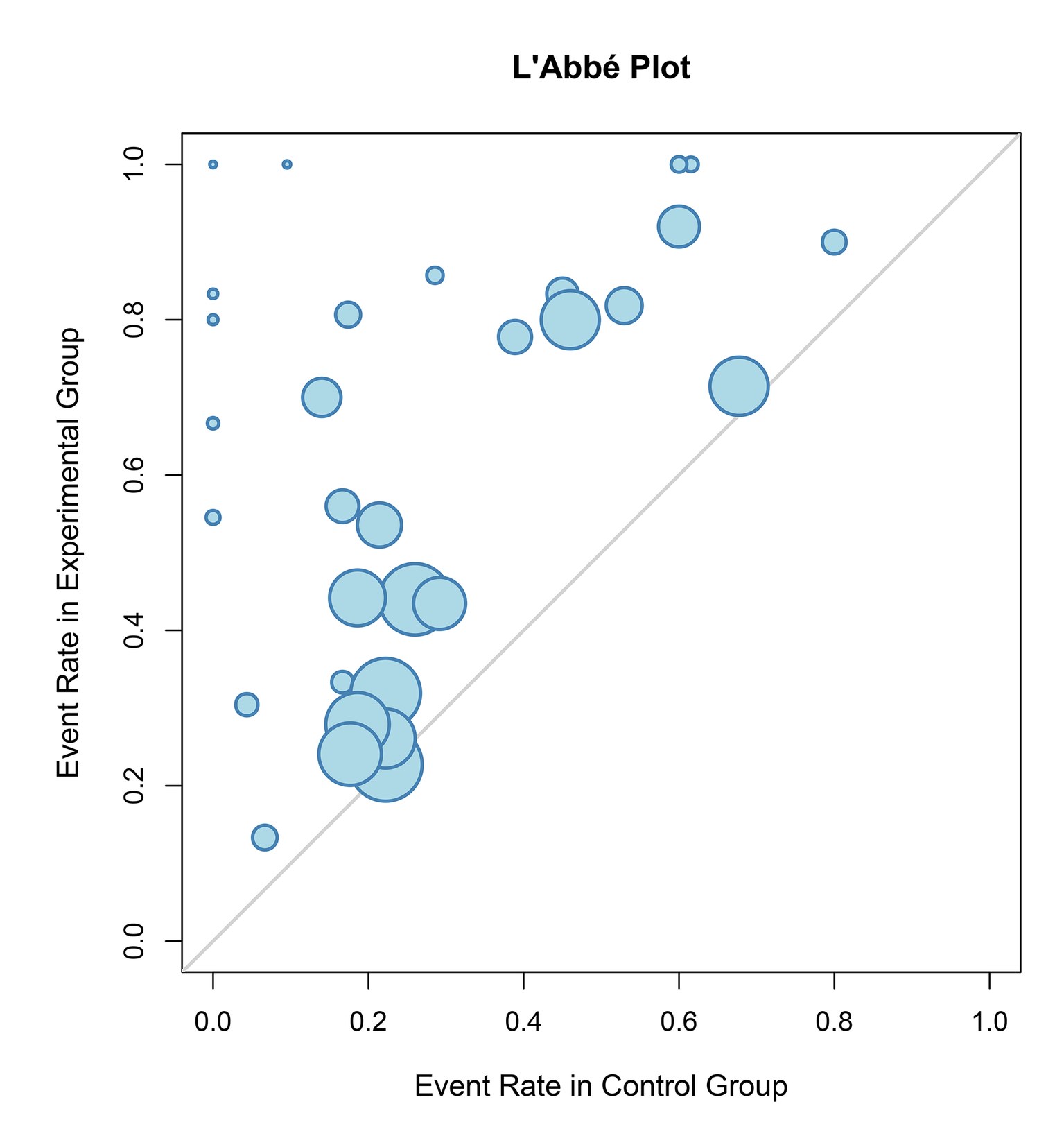


Fig S87 Heterogeneity test of ulcer healing rate

## Fig S88


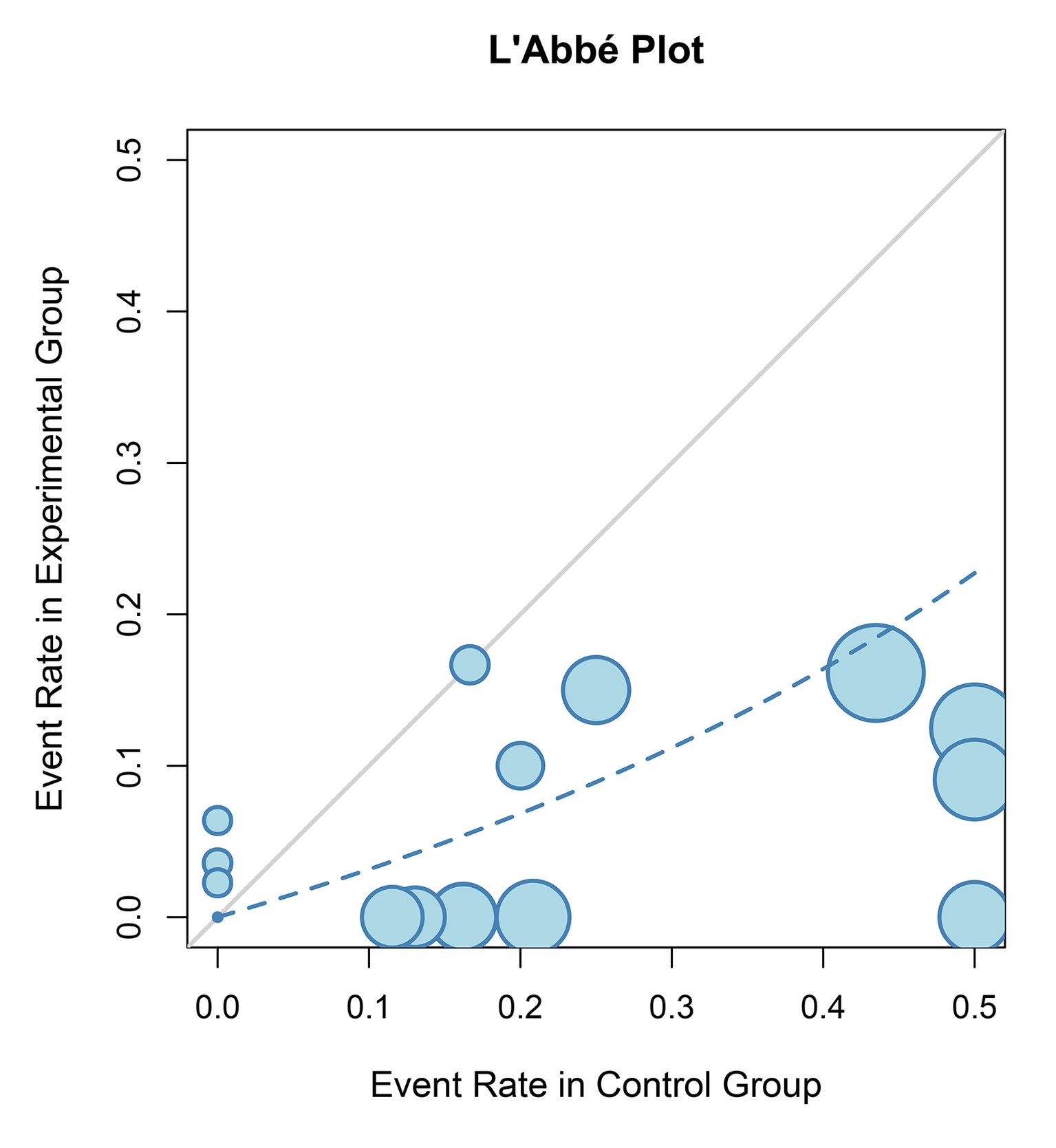


Fig S88 Heterogeneity of amputation rates

## Fig S89


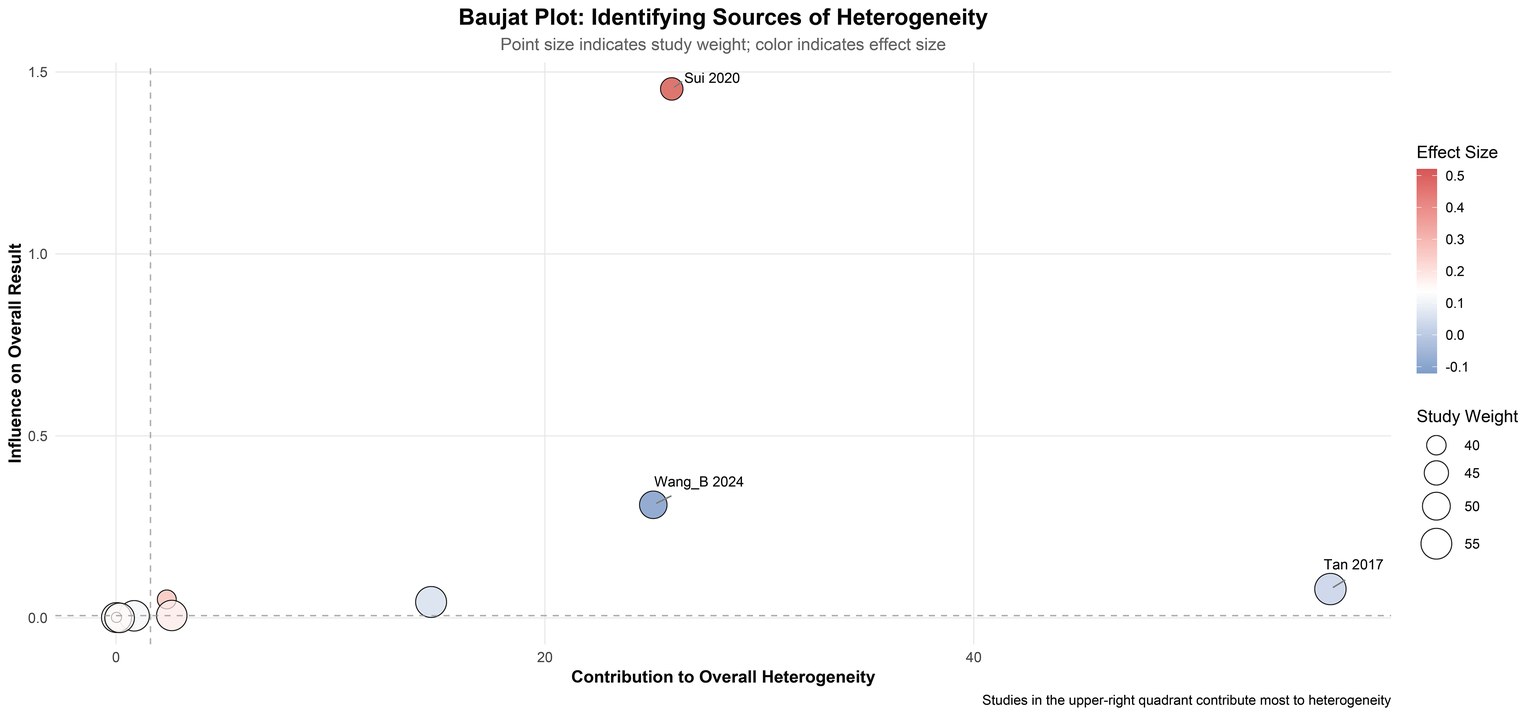


Fig S89 ABI heterogeneity test

## Fig S90


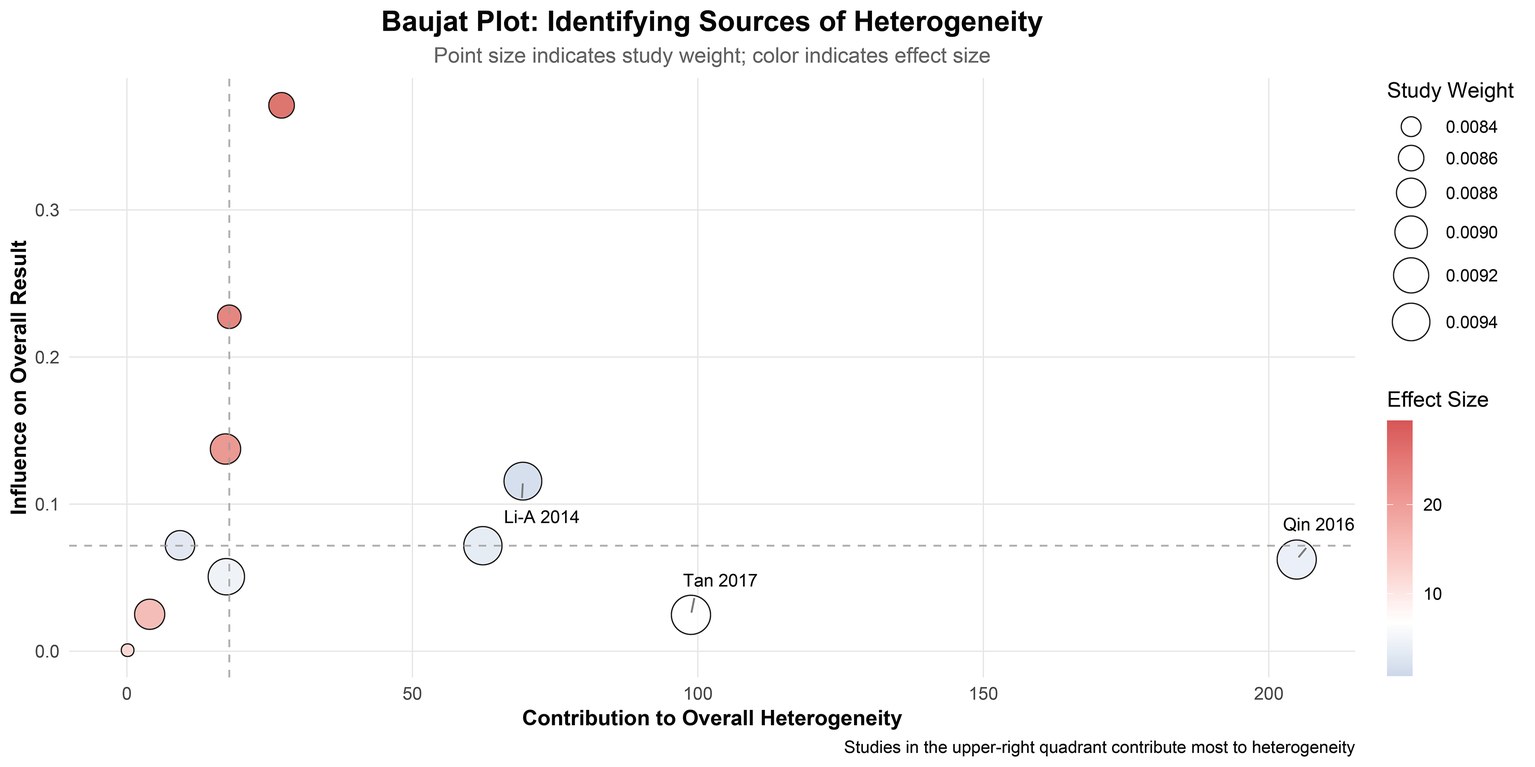


Fig S90 TcPo2 heterogeneity analysis

## Fig S91


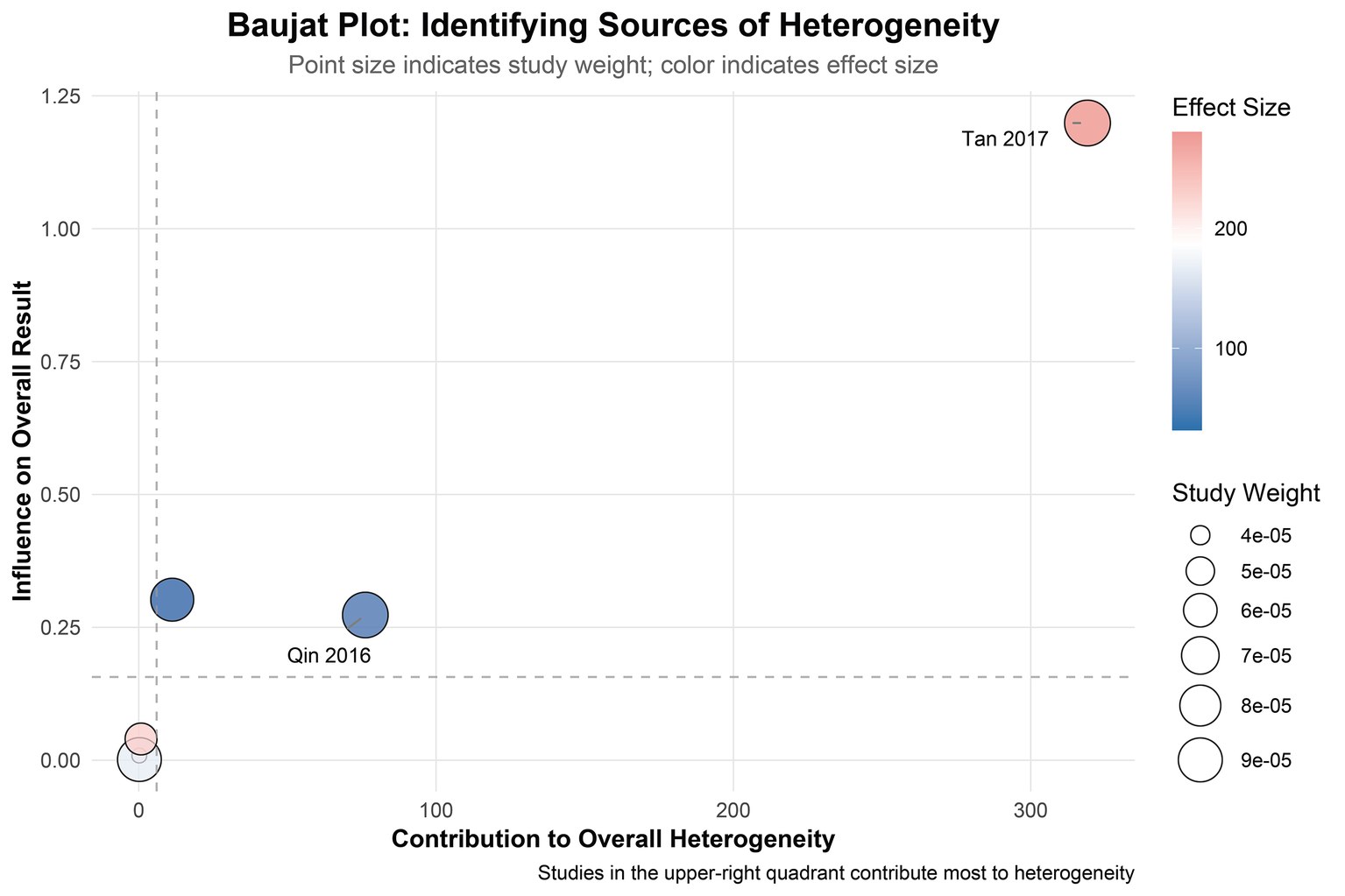


Fig S91 Painless walking distance overall heterogeneity test

## Fig S92


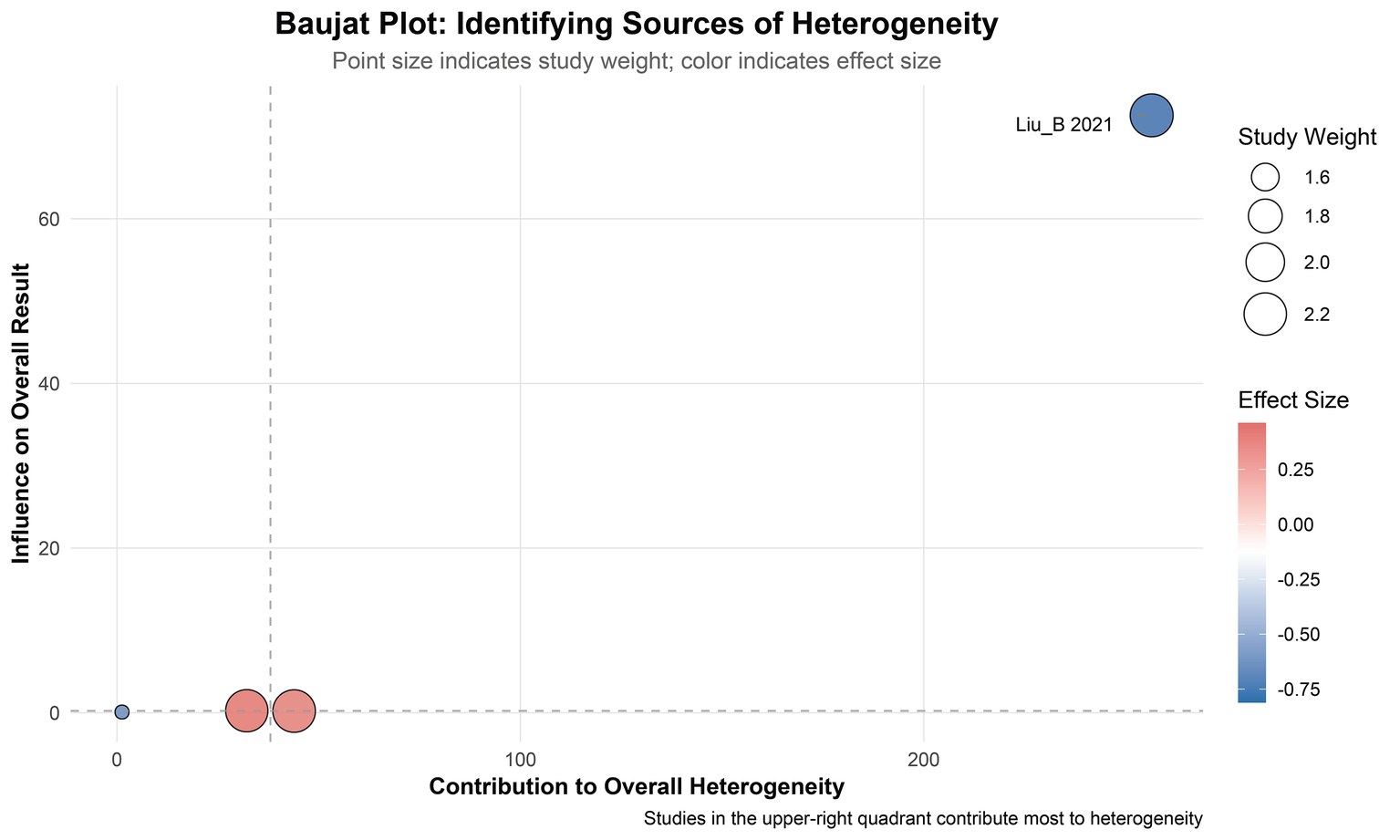


Fig S92 Painless walking limp score Heterogeneity analysis

## Fig S93


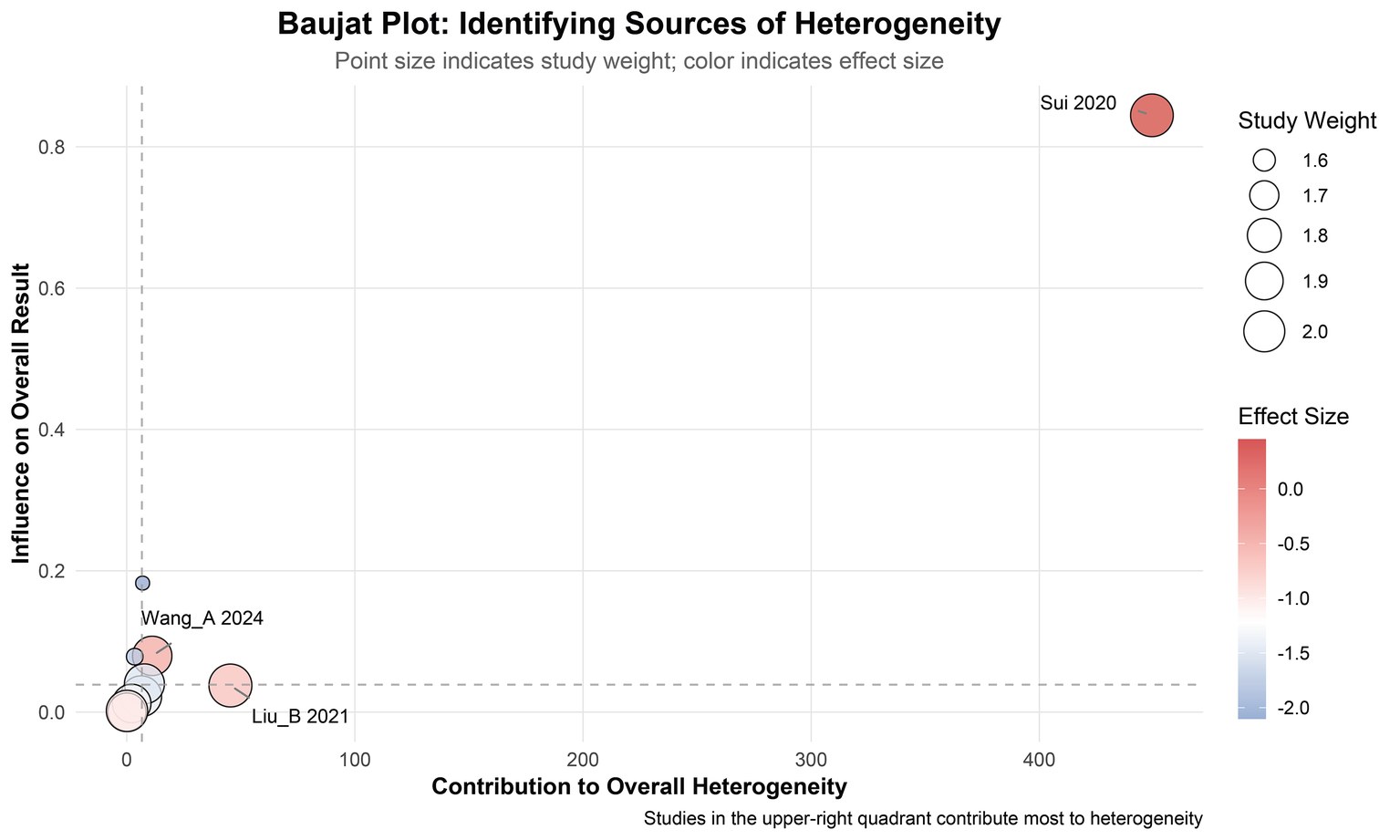


Fig S93 Resting pain score heterogeneity analysis

## Fig S94


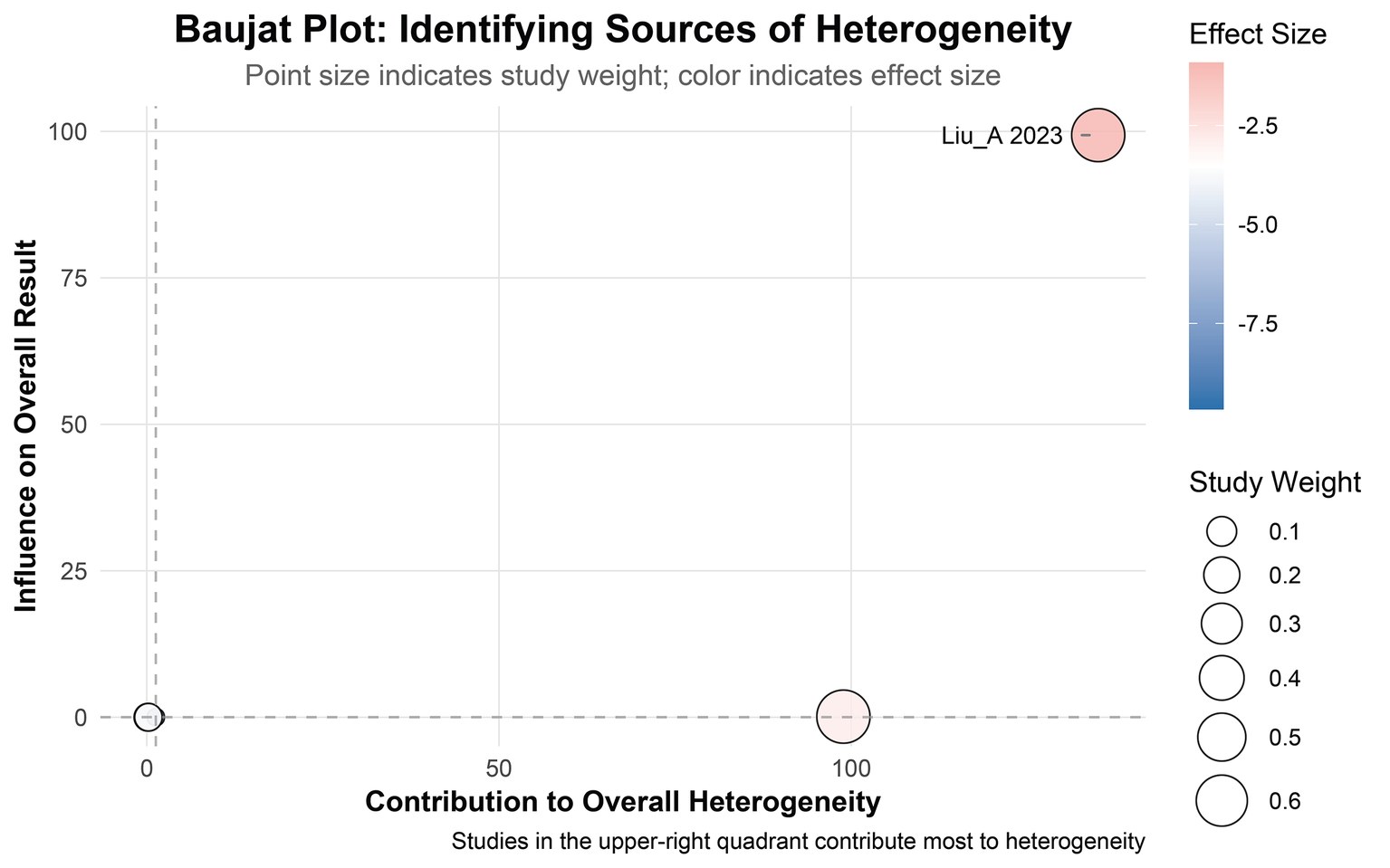


Fig S94 Results of ulcer area heterogeneity analysis

## Fig S95


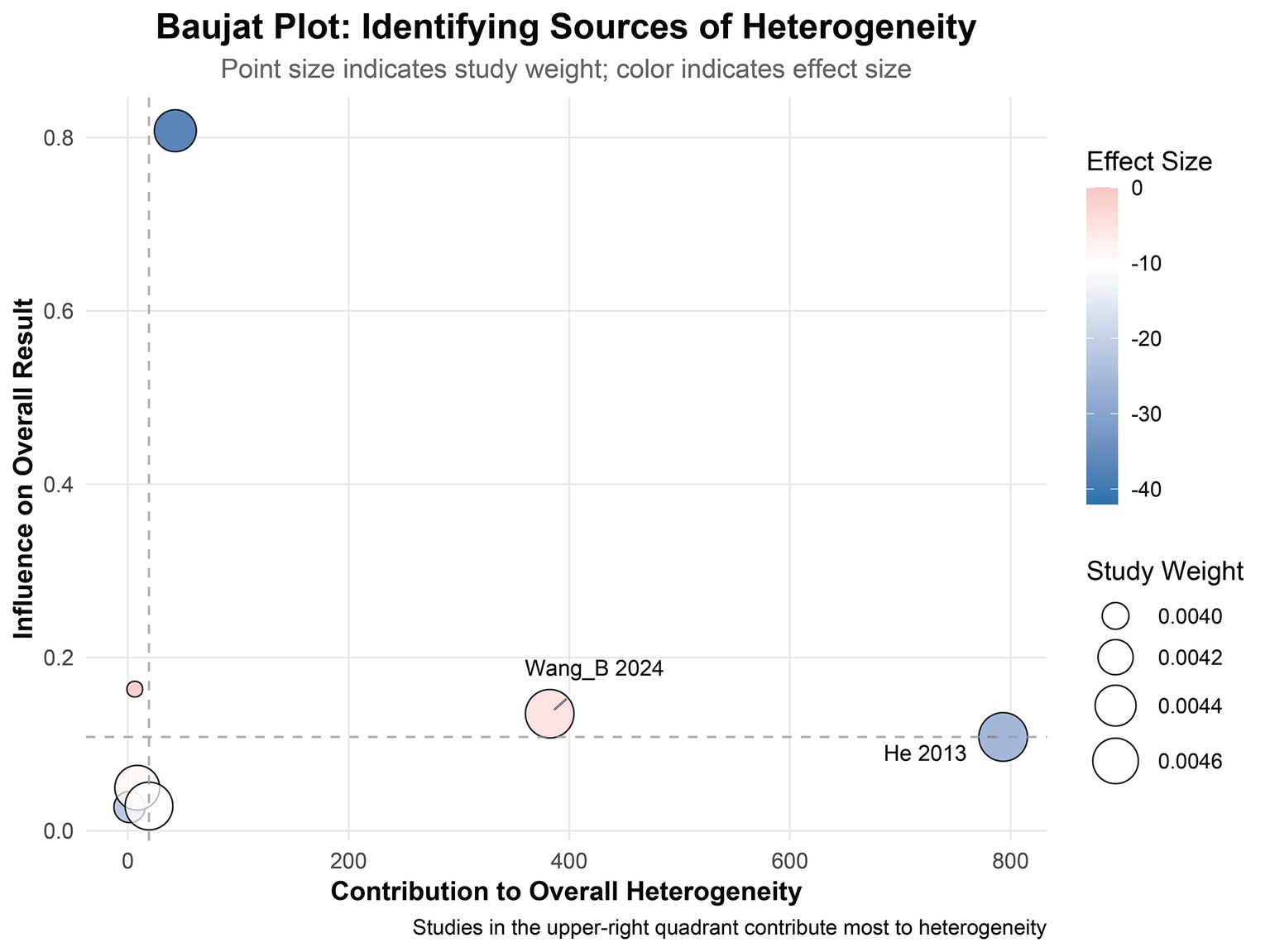


Fig S95 Analysis of Heterogeneity in Ulcer Healing Time
